# Host Genes Related to Paneth Cells and Xenobiotic Metabolism Are Associated with Shifts in Human Ileum-Associated Microbial Composition

**DOI:** 10.1371/journal.pone.0030044

**Published:** 2012-06-13

**Authors:** Tianyi Zhang, Robert A. DeSimone, Xiangmin Jiao, F. James Rohlf, Wei Zhu, Qing Qing Gong, Steven R. Hunt, Themistocles Dassopoulos, Rodney D. Newberry, Erica Sodergren, George Weinstock, Charles E. Robertson, Daniel N. Frank, Ellen Li

**Affiliations:** 1 Department of Applied Mathematics and Statistics, Stony Brook University, Stony Brook, New York, United States of America; 2 Department of Medicine, Stony Brook University, Stony Brook, New York, United States of America; 3 Department of Ecology and Evolution, Stony Brook University, Stony Brook, New York, United States of America; 4 Department of Medicine, Washington University-St. Louis School of Medicine, Saint Louis, Missouri, United States of America; 5 Department of Surgery, Washington University-St. Louis School of Medicine, Saint Louis, Missouri, United States of America; 6 The Genome Institute, Washington University-St. Louis School of Medicine, Saint Louis, Missouri, United States of America; 7 Department of Molecular, Cellular and Developmental Biology, University of Colorado, Boulder, Colorado, United States of America; 8 Department of Medicine, University of Colorado, Aurora, Colorado, United States of America; Charité, Campus Benjamin Franklin, Germany

## Abstract

The aim of this study was to integrate human clinical, genotype, mRNA microarray and 16 S rRNA sequence data collected on 84 subjects with ileal Crohn’s disease, ulcerative colitis or control patients without inflammatory bowel diseases in order to interrogate how host-microbial interactions are perturbed in inflammatory bowel diseases (IBD). Ex-vivo ileal mucosal biopsies were collected from the disease unaffected proximal margin of the ileum resected from patients who were undergoing initial intestinal surgery. Both RNA and DNA were extracted from the mucosal biopsy samples. Patients were genotyped for the three major NOD2 variants (Leufs1007, R702W, and G908R) and the ATG16L1T300A variant. Whole human genome mRNA expression profiles were generated using Agilent microarrays. Microbial composition profiles were determined by 454 pyrosequencing of the V3–V5 hypervariable region of the bacterial 16 S rRNA gene. The results of permutation based multivariate analysis of variance and covariance (MANCOVA) support the hypothesis that host mucosal Paneth cell and xenobiotic metabolism genes play an important role in host microbial interactions.

## Introduction

Inflammatory bowel diseases are complex genetic disorders resulting from the interplay of genetic and environmental factors [1]–[3]. Crohn’s diseases (CD) and ulcerative colitis (UC) represent the two major inflammatory bowel diseases (IBD) phenotypes and are distinguished by different patterns of disease location. The inflammation in CD patients may be located anywhere along the gastrointestinal tract, but in the majority (80%) of CD patients, the terminal ileum is involved. In UC, the inflammation is confined to the colon. Because there is evidence that isolated Crohn’s colitis are associated with genetic factors that are distinct from ileal CD, and the overlap between genetic factors associated with UC and isolated Crohn’s colitis, we have focused our attention on the ileal CD subphenotype as a relatively homogenous category that is distinct from isolated colitis (CD or UC) and non-IBD controls [4]–[6].

Single nucleotide polymorphisms in the NOD2 gene and the ATG16L1 gene have been linked to alterations in innate host immunity, particularly Paneth cell function and with ileal CD phenotype [7]–[14]. We previously reported that increased *CD3D* mRNA expression in disease affected ileum resected from 18 ileal CD patients was associated with NOD2 genotype [15]. We also observed alterations in mRNA gene expression in the disease unaffected proximal margin of resected ileum from 19 ileal CD patients compared to 9 control non-IBD patients, regardless of NOD2 genotype [15]. The microarray dataset has recently been further expanded to include 47 ileal CD, 27 UC and 25 non-IBD control subjects (total = 99).

Culture-independent microbiological technologies coupled with high-throughput DNAsequencing have uncovered alterations in human intestine-associated microbial compositions (“dysbiosis”) in IBD patients compared with controls [16]–[25]. Ileal CD phenotype has been also associated with shifts in intestinal and fecal microbial composition, particularly reduced relative frequency of *Faecalibacterium prausnitzii* [19], [20], [23]. In addition to disease phenotype, exploratory analyses have also associated NOD2 genotype to intestinal associated microbial composition [22]. We have recently completed 16 S rRNA sequence analysis on an independent set of disease-unaffected ileal biopsies collected of 52 ileal CD, 58 colitis and 60 control patients without IBD undergoing initial surgical resection [24]. Of the 170 subjects with microbial composition data and 99 subjects with mRNA expression profiles, 84 subjects had paired microarray and microbial datasets. We report here the results of permutation based MANCOVA of these paired mRNA expression and microbial profiles in 34 ileal CD, 27 UC and 23 non-IBD control patients.

## Results

### Patient Characteristics

As shown in Table 1, 35% of ileal CD patients harbored at least one NOD2 risk allele (NOD2R) compared to 13% of nonIBD control patients, consistent with previous studies [1]–[3]. Only one ileal CD patient was homozygous for the ATG16L1 nonrisk allele. The patients were predominantly Caucasian. The median age of surgery was lower in ileal CD patients than nonIBD control patients. Thirty percent of colitis patients had a concomitant *C. difficile* infection, consistent with the increased incidence of this infection noted previously in IBD patients [26], [27]. Thirty to fifty percent of IBD patients and none of the non-IBD control patients were treated with 5-aminosalicylic acid (5-ASA), steroids, immunomodulators or an anti-TNFα agent. All of the subjects received intravenous antibiotics within one hour of incision [28].

**Table 1:** Distribution of NOD2 composite and ATG16L1 genotype and clinical characteristics of ileal CD, colitis and control non-IBD patients.

Variables	Ileal CD(n = 34)	UC(n = 27)	Control(n = 23)	P-value	FDR
NOD2 genotype				0.11	0.15
NOD2^R^ (R/R + R/NR)	35%	19%	13%		
NOD2^NR^ (NR/NR)	65%	81%	87%		
ATG16L1 genotype				0.23	0.28
ATG16L1^R/R^	41%	41%	43%		
ATG16L1^R/NR^	56%	41%	35%		
ATG16L1^NR/NR^	3%	18%	22%		
Gender (male)	38%	59%	30%	0.095	0.14
Race (Caucasian)	94%	96%	96%	0.92	0.92
**Median age (range) y**	36 (21–59)	43 (17–64)	55 (32–84)	<0.001	<0.001
Current smoker	38%	11%	22%	0.048	0.08
**Positive fecal C. difficile toxin**	0%	30%	0%	<0.001	<0.001
Median BMI (range) kg/m^2^	25 (16–38)	24(18–43)	28(20–38)	0.43	0.47
**5-ASA**	52%	70%	0%	<0.001	<0.001
**Steroids**	55%	74%	0%	<0.001	<0.001
**Immunomodulators**	50%	52%	0%	<0.001	<0.001
**Anti-TNFα biologics**				0.003	0.006
Current (≤8 weeks)	35%	41%	0%		
Past (>8 weeks)	9%	7%	0%		
Never	56%	52%	100%		

The variables shown below are included in the subsequent MANCOVA analyses for 84 patients. Chi-square test for contingency table was used for categorical data and Kruskal-Wallis test was used for age and BMI. Variables that differed significantly (FDR ≤0.05) are **bolded**.

### Comparison of Ileal Mucosal Expression Profiles between Ileal CD, UC and non-IBD Control Subjects

**Figure 1:**
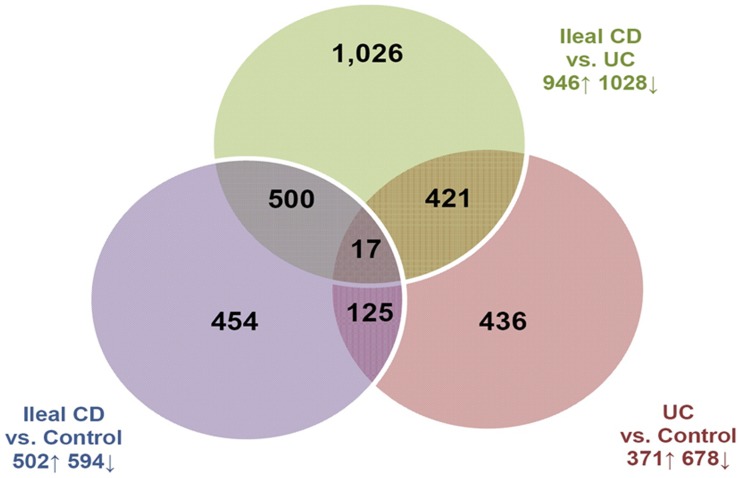
Venn diagram of the union of the gene-probes identified by SAM. Two-class unpaired SAM analyses of ileal CD vs Control samples, UC vs. Control Samples and ileal CD vs. UC samples have been conducted. The number of gene-probes that overlapped between the three separate analyses is shown within the Venn diagram. The total numbers of upregulated and downregulated gene-probes for each individual analysis are shown on the side.

Normalization and pre-processing of the data to filter out undetectable gene-probes resulted in a total of 26,765 gene-probes. Because this number of variables still greatly exceeded the sample size, we sought to further reduce the number of input microarray variables. We reasoned that genes that were differentially expressed between the three disease phenotypes were most likely to be involved in altering host-microbial interactions. Two-class unpaired SAM analysis was used to identify genes differentially expressed (fold change >1.5, FDR <0.05) between ileal CD and Control samples, between UC and Control samples, and between CD and UC samples [29]. The results indicate significant differences in gene expression patterns between all three disease phenotypes (see Table S1) [30], [31]. By taking the union of the candidate genes identified by the three two-class comparisons, the dimensions of the normalized microarray data was reduced from 26,765 to a 2,979 gene-probe set (see Fig. 1).

Hierarchical clustering of the 2,979 gene-probe set was then carried out by using 1-correlation dissimilarities and Ward linkage as previously described [32], [33]. The number of clusters was chosen to be 43, based on inspection of the R2 plot (see Table S2). Hierarchical clustering of the original 26,765 gene-probe set was also carried out using the same algorithms to 265 clusters. This number of clusters was again chosen based on inspection of the R2 plot. In all but four (clusters #14, 20, 31, 36) of the 43 clusters, greater than 40% of the gene-probes were concentrated in two of the 265 clusters obtained by clustering the original 26,765 gene-probe set, indicating that using SAM to reduce the number of probes did not appear to bias clustering. We reasoned that genes, which were highly correlated with each other, would be linked by common biological pathways. Ingenuity Pathway Analysis (IPA) canonical pathways were associated (P<0.01 and at least 4 gene probes) in 12 of 43 clusters (see Table S3). In addition, direct inspection of cluster #24 revealed that this cluster included a number of genes expressed in Paneth cells, such as the α-defensins.

### Permutational based MANCOVA with Stepwise Variable Selection and Gene Cluster Centroids as Independent Variables

For 84 of 99 ileal mucosal samples with microarray profiles, 454 pyrosequencing of the V3, V4, and V5 (V3–V5) hypervariable regions of the 16 S rRNA gene was completed using primers adopted by the Human Microbiome Project [34], [35]. A vector consisting of the relative frequencies of six phyla/subphyla categories (excluding the seventh “other Taxa” category):1.) Actinobacteria, 2.) Bacteroidetes, 3.) Firmicutes. Clostridium Group IV, 4.) Firmicutes. Clostridium Group XIVa, 5.) Firmicutes. Bacilli, 6.) Proteobacteria, was used as the dependent variables. The 43 microarray cluster medians were used as cluster centroids [36], [37], in addition to disease phenotype and the other 12 input variables in the analysis. Using the stepwise variable selection method, permutation based MANCOVA selected disease phenotype, a Paneth cell gene enriched cluster, two xenobiotic metabolism gene enriched clusters, and NOD2 genotype as the independent variable set (see Table 2). Gene-probes included in these three clusters are listed in Table 3. We obtained similar results using the cluster mean or first principle component [36], [38] as the cluster centroids (data not shown). We also obtained similar results when parallel Sanger and 454 V1–V3 16 S sequence datasets were used (data not shown).

**Table 2:** Selected variables associated (P≤0.05) with shifts in ileum associated microbial composition.

Selected Variables	Df	R^2^	P value
Phenotype	2	0.0460	0.037
Paneth cell cluster (Cluster 24)	1	0.0576	0.002
Xenobiotic cluster A (Cluster 13)	1	0.0319	0.024
Xenobiotic cluster B (Cluster 2)	1	0.0291	0.041
NOD2 genotype	1	0.0414	0.011

**Table 3:** Gene list for the Paneth cell gene enriched cluster and the xenobiotic metabolism gene enriched clusters A and B.

Cluster Function	Gene list
Paneth Cell (cluster 24)	*ENPP7, * ***DEFA5*** *, TM4SF20, RGN, MDK, * ***REG3A*** *, BCMO1, BAI2, GPR172B, CA9, ANGPTL4, ASAH2, CEL, NPC1L1, SERPINB5, SERPINA1, NPNT, VNN1, DDO, PRSS2, * ***PLA2G2A*** *, PRSS1, SLC2A12, CCK, CDKN1C, UNC5CL, FBXO2, KLK12, SIGLEC15, CLCA1, RHBG, CCL25, AZGP1, LCT, * ***DEFA6*** *, GCNT1, SLC16A4, UNC93A, LOC100128979, WNT11, VNN1, PEAR1, LOC643201, * ***ITLN2*** *, * ***REG4*** *, LOC100240735, LOC100240735, * ***REG3G*** *, PRSS2*
Xenobiotic Metabolism Cluster A (cluster 13)	*CPO, PRSS7, AATK, HEBP1, * ***ABCG5*** *, * ***CYP2C9*** *, GATM, SLC5A12, * ***GSTA1*** *, MS4A8B, SULT1E1, PTGR1, CYP2C19, CYP2C19, * ***ABCC2*** *, NR0B2, ABCA4, APOC3, CYBRD1, MME, MTTP, GSTA2, UNC93A, SST, ACE2, GSTA3, SOAT2, FBP1, TM4SF5, SLC23A3, EDN2, * ***NR1I3*** *, PDIA2, ENPEP, UGT2B4, C17ORF78, SLC5A11, ANO6, KCNH6, C19ORF77, C21ORF129, MGAM, ABCC2, PDZK1, CYP2C18, CPS1, NQO2, DNASE1, DHDH, OSR2, BST1, PIK3C2G, MEP1B, APOB, RBP2, AADAC, PEPD, MAOB, MAOB, APOA4, REEP6, MEP1A, GSTA5, PHYHIPL, OAT, MEP1A, * ***SULT2A1*** *, MGAT3, MME, GSTA2, TIAM2, LOC285733, EMB, SLC16A10, SLC6A4, EMB, LOC149703, TMEM229A, C19ORF69*
Xenobiotic Metabolism Cluster B (cluster 2)	*NELL2, ACOX2, CYP2J2, * ***SULT1A2*** *, PRR15L, GUCA2A, LGALS2, PCK2, DDC, RNF128, FMO1, FAM82A2, ABAT, SAT2, NAT8, AGXT2, BTNL3, MYO1A, MTHFS, SMPD3, CBS, VIL1, EDN3, ABCG2, MOSC2, G6PC, CDK20, CYP4F3, VIL1, ABHD6, HSD17B11, TRPM6, C9ORF24, KLKB1, TM4SF4, EFNA1, CBR1, ANKRD43, SLC9A3R1, SUSD2, SLC1A7, LINCR, CHP2, SLC17A4, MAF, FAM151A, OAZ1, KAZALD1, APOM, C9ORF40, ANXA13, GUCA2B, GLRX, C6ORF123, EPHX2, CTU2, CES2, PBX1, KAZALD, SP8, SULT1A4, TRPM6, VPS35, NAT8B, CSNK1D, STAU2, PTPRF, PGRMC2, AGPHD1, AGXT2, APOM, IYD, LRAT, LAMA1, ADI1*

The genes selected for further correlation analyses are **bolded.**

See Table S3 for the complete list of all clusters.

To examine correlations between gene transcripts and bacterial taxa at a more granular level, we selected individual gene transcripts within these microarray clusters and individual bacterial genera. The selected transcripts included the alpha defensins, (*DEFA5* and *DEFA6*), which have exhibited altered regulation in ileal Crohn’s disease [10], [39], [40], and included cellular detoxification genes, which have exhibited altered regulation in ulcerative colitis [41]. The bacteria genera selected included the Faecalibacterium and Shigella/Escherichia genera, because the relative frequency of *Faecalibacterium prausnizii* has been reported to be reduced and that of *Escherichia coli* have been reported to be increased in patients with ileal CD. Bacterial genera, previously selected as ulcerative colitis related were also included in this analysis [42].

**Figure 2:**
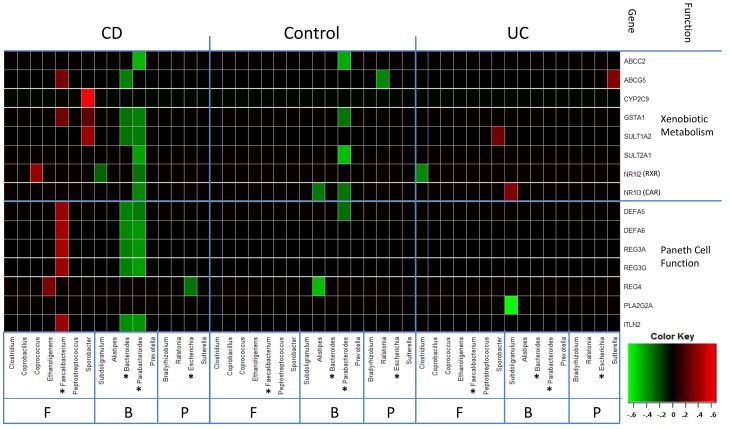
Correlations between selected mRNA transcripts and bacterial genera. Selected transcripts from the Paneth cell and xenobiotic metabolism microarray clusters are listed with their public reference along the vertical axis (see text). Selected bacterial genera are classified by phyla. CD, ileal CD phenotype; Control, non-IBD control phenotype; UC, UC phenotype; F, Firmicutes; B, Bacteroidetes; P, Proteobacteria. *Red squares* represent positive correlations (P<0.05), and *green squares* represent negative correlations (P<0.05).

As shown in Figure 2, a positive correlation (P<0.05) between the relative frequency of the Faecalibacterium genus (Firmicutes Phylum, Clostridium GroupIV) and mRNA expression levels of Paneth cell genes, including *DEFA5* and *DEFA6*, was observed in ileal CD patients but not in non-IBD controls or UC patients. Negative correlations (P<0.05) were observed between the relative frequency of the Bacteroidetes genus and Parabacteroides genus (Bacteroidetes phylum) and mRNA expression levels of the *REG* genes in ileal CD patients, but not in UC or non-IBD control patients. Furthermore, negative correlations (P<0.05) were observed between the relative frequency of the Parabacteroides genus (Bacteroidetes phylum) and mRNA expression levels of cellular detoxification genes in non-IBD control and ileal CD patients but not in UC patients. The highest correlation (out of >500 comparisons) was observed between Faecalibacterium and *DEFA6* (r = 0.59, P = 0.00024, FDR = 0.057) in ileal CD patients.

## Discussion

In this exploratory study, we report the results of the analysis integrating human ileal mucosal microarray expression profiles with microbiota profiles. Since the number of genes and bacterial taxa greatly exceed the number of samples, we sought to shrink these high dimensional datasets by grouping the bacteria taxa into broad phyla/subphyla categories, and by selecting potentially disease relevant transcripts by SAM followed by clustering using 1- correlation dissimilarity measure. We were gratified to observe that a number of the resulting clusters could be linked to canonical pathways by IPA. Furthermore inspection of one of the clusters revealed that it included a number of genes that were expressed in Paneth cells. We therefore selected gene transcripts within these three microarray clusters for further analysis. These genes included the alpha defensins and members of the regenerating gene (REG) family. The alpha defensins are antimicrobial peptides that are secreted by Paneth cells. Manipulation of alpha defensin expression in experimental animals has been shown to alter gut microbial composition [43]. On the other hand, monoassociation of a Bacteroidetes species with germ free animals was shown to alter regulation of Paneth cell gene expression [44]. Altered expression of alpha defensins have been associated with the ileal Crohn’s disease phenotype. The *REG* genes belong to the calcium (C-type) dependent lectin superfamily and have been noted to be upregulated in CD and UC intestinal tissues [45], [46]. Expression levels of cellular detoxification genes, which are target genes for the transcription factor pregnane X receptor (PXR and also termed NR1I2), have been previously noted to be down-regulated in the colons of UC patients [41]. The expression levels of these genes were correlated with bacterial genera that had been previously reported to be disease associated [42]. At a threshold of P≤0.05, exploratory analyses revealed potential correlations between transcript levels of specific genes with individual bacterial genera (e.g. Faecalibacterium, Bacteroidetes and Parabacteroides), that were modulated by disease phenotype. By honing in on these promising correlations identified by exploratory studies, we hope to be able to further confirm these observations in an expanded set of samples. Co-linearity between input variables may occur, despite our efforts to shrink the dimensions of the datasets. This may account for why *C. difficile* was not selected in this subset of the microbial dataset. Alternatively *C. difficile* may not have been selected because paired microarray and microbial data have been collected on a smaller number of subjects thus far. While the use of immunomodulators and anti-TNFα biologics were included as co-variates in the MANOVA [24], we cannot completely exclude the potential confounding effects of these drugs on the microbial composition and mucosal gene expression. Nevertheless our results demonstrate that integrating paired expression profiles and microbial data can lead to the discovery of biologically meaningful host-microbial interactions in inflammatory bowel diseases. We anticipate that as we expand the sample set, other associations will be detected.

## Materials and Methods

### Patients and Acquisition of Macroscopically Disease-unaffected Proximal Margin Ileal Tissue Samples

This study was approved by the Institutional Review Boards of Washington University-St. Louis and Stony Brook University. Ileal CD patients undergoing initial ileocolic resection, UC patients undergoing initial total colectomy and Control non-IBD patients undergoing either right hemicolectomy or total colectomy were prospectively enrolled in a consecutive fashion by the Washington University Digestive Diseases Research Core Center Tissue Procurement Facility to donate surgically resected tissue samples between April 2005 and February 2010. Patients who were unwilling or unable to give informed consent were excluded. Clinical information and patient samples were stripped of all identifying information and assigned a patient code and sample code. The de-identified patients were genotyped for the three major NOD2 and ATG16L1 genotypes and phenotyped as previously described [15]. All of the patients received antibiotics within one hour of incision. Ex-vivo biopsies were obtained of the disease unaffected proximal margin of the surgical resection specimens as previously described. RNA and DNA were extracted from the biopsy samples as previously described [15].

### Human Ileal Mucosal Expression Profiles

The test RNA and a common reference ileal RNA were labeled and the resulting probes were hybridized to Agilent Whole Human Genome Arrays (Agilent No. G4410A) as previously described [15]. The pre-processing, filtering and normalization of the array data was conducted using the R package LIMMA [47], [48]. Probes with all Genepix flags less than −50 were treated as absent and removed from the dataset. There were technical duplicates on three samples and the log2 ratios for these three samples were averaged prior to analysis. Genes that were differentially expressed between ileal CD vs. Control, UC vs. Control, and ileal CD vs. UC were selected by conducting three two-class unpaired comparisons using SAM, with a cutoff of change >1.5 fold and false discovery rate (FDR) <0.05 [29]. The hierarchical clustering was carried out by using 1-r dissimilarity measurement and Ward linkage as previously described [32], [34]. The cluster number was decided based on inspection of the coefficient of determination (R2) plot [49], [50]. The biological significance of these clusters was assessed by using Ingenuity Pathway Analysis (IPA) software [51]. To select cluster-enriched IPA canonical pathways, we lowered the threshold of the *p*-value from 0.05 to 0.001, and included only pathways that included ≥4 genes in the cluster. The data discussed in this publication have been deposited in NCBI’s Gene Expression Omnibus and are accessible through GEO Series accession number GSE24287 (http://www.ncbi.nlm.nih.gov/geo/query/acc.cgi?acc=GSE24287).

### Assessment of Ileal-associated Microbial Composition

The V3–V5 region was targeted by using barcoded primers 357F (5′-CCTACGGGAGGCAGCAG-3′) and 907R (5′CCGTCAATTCMTTTRAGT) and were identical to the primers used by the Human Microbiome Project to characterize the microbiota in healthy human subjects. All sequences were screened for fidelity to a 16 S rRNA bacterial covariance model (CM) based on secondary structure using the Infernal software package [52] and were checked for chimerism with ChimeraSlayer [53] http://microbiomeutil.sourceforge.net/#A_CS). Potentially chimeric sequences and sequences lacking high fidelity to the CM were removed from subsequent analysis. Genera level taxonomic calls were produced by the *RDP Classifier* [53], which performs naïve Bayesian taxonomic classification versus a training set. This project used the code and training set provided by RDP (Version 2.1, http://sourceforge.net/projects/rdpclassifier/) April 6, 2010 respectively. The sequences were also classified into seven phyla/subphyla categories. The seven categories were 1) *Actinobacteria*, 2) *Bacteroidetes*, 3) *Firmicutes.*Clostridium Group IV, 4) *Firmicutes.* Clostridium Group XIVa, 5.) *Firmicutes.* Bacillus, 6.) *Proteobacteria*, and 7.) Other taxa. The subdivisions of the Firmicutes phyla were based on concordance between the RDP classifier and the Greengenes 16 S rRNA phylogenetic schema [54]–[56]. The Clostridium GroupIV and Clostridium Group XIVa taxa are subsets of the Lachnospiriciae taxon [22], [57]. The sequence screening, classification, final binning and enumeration operations described were performed within a python based analysis pipeline created for this project [24]. Assembled Sanger sequences were deposited in GenBank accession HQ739096-HQ821395. 454 V1–V3 and V3–V5 sequences were deposited in the Sequence Read Archive accession SRX021348-SRX021368, SRX037800-SRX037802. Clinical and genotyping data can be accessed through the dbGAP authorized access system. Request access to: phs000255. The study accession is SRP002479 “Effect of Crohn’s disease risk alleles on enteric microbiota”. In order to request access to any of the individual-level datasets within the controlled-access portions of the database, the Principal Investigator (PI) and the Signing Official (SO) at the investigator’s institution will need to co-sign a request for data access, which will be reviewed by an NIH Data Access Committee at the appropriate NIH Institute or Center (https://dbgap.ncbi.nlm.nih.gov/aa/wga.cgi?page=login).

### Statistical Analysis

In order to investigate the relationship between gene expression and bacteria composition, permutational MANOVA with stepwise variable selection was performed for a vector including six bacteria taxa, which served as the dependent variable [58]. Because the dependent variable is a vector of compositions, the centered log ratio transformation was used on the bacterial proportions [59]. The cluster medians [36], [37] were chosen to represent the cluster centroids and included as input variables along with clinical information (patients phenotype, age, race, smoking, BMI, gender, *C._difficile*, 5-ASA, steroids, immunomodulator, TNF) and genotypes (NOD2 and ATG16L1).

## Supporting Information

Table S1 **A. Gene-probes upregulated in CD compared to Control (n = 502). B. Gene-probes downregulated in CD compared to Control (n = 594). C. Gene-probes upregulated in UC compared to Control (n = 371).**
(DOC)

Table S2 **Gene-probes in the 43 clusters.**
(DOCX)

Table S3 **Clusters obtained by after dimension reduction using SAM.** The clusters are listed along with the percentage of variation explained by the 1^st^ PC of the cluster, which is related to the compactness of the cluster. The bolded clusters are the clusters in which >40% of the gene-probes were concentrated in two of the 265 clusters obtained without prior dimension reduction. The clusters were considered enriched for genes that were differentially expressed in CD vs. Control, UC vs. Control or CD vs. UC if ≥50% of the genes with a correlation of ≥0.75% to the 1^st^ PC of the cluster demonstrated a significant fold change (see Supplementary Table 1). Clusters were considered enriched for genes in an IPA canonical pathway if P<0.01 and at least 4 genes were in the pathway. In addition we listed our interpretation of the biological significance of the pathway.
(DOCX)

## References

[1] Ng SC, Woodrow S, Patel N, Subhani J, Harbord M (2011). Role of genetic and environmental factors in British twins with inflammatory bowel disease. Inflamm Bowel Dis doi: 10.1002/ibd.21747.. [Epub ahead of print].

[2] Khor B, Gardet A, Xavier RJ (2011). Genetics and pathogenesis of inflammatory bowel disease Nature.

[3] Abraham C, Cho J (2009). Mechanisms of disease: inflammatory bowel disease New Engl.. J Medicine.

[4] Hancock L, Beckly J, Geremia A, Cooney R, Cummings F (2008). Clinical and molecular characteristics of isolated colonic Crohn’s disease Inflamm Bowel Dis.

[5] Waterman M, Xu W, Stempak JM (2011). Distinct and overlapping genetic loci in Crohn’s disease and ulcerative colitis: correlations with pathogenesis Inflamm Bowel Dis.

[6] Chen H, Lee A, Bowcock A, Zhu W, Li E (2011). Influence of Crohn’s disease and smoking on disease location Dis Colon Rectum.

[7] Cuthbert A, Fisher S, Mirza MM, King K, Hampe J (2002). The contribution of NOD2 gene mutations to the risk and site of disease in inflammatory bowel disease.. Gastroenterol.

[8] Lesage S, Zouali H, Cezard JP, Cézard JP, Colombel JF (2002). CARD15/NOD2 mutational analysis and genotype-phenotype correlation in 612 patients with inflammatory bowel disease.. Am J Human Genet.

[9] Ogura Y, Lala S, Xin W, Smith E, Dowds TA (2003). Expression of NOD2 in Paneth cells: a possible link to Crohn’s ileitis Gut.

[10] Bevins CL, Stange EF, Wehkamp J (2009). Decreased Paneth cell defensin expression in ileal Crohn’s disease is independent of inflammation, but linked to the NOD2 1007fs genotype Gut.

[11] Simms LA, Doecke JD, Walsh MD, Huang N, Fowler EV (2008). Reduced alpha-defensin expression is associated with inflammation and not NOD2 mutation status in ileal Crohn’s disease Gut.

[12] Prescott NJ, Fisher SA, Franke A, Hampe J, Onnie CM (2007). A nonsynonymous SNP in ATG16L1 predisposes to ileal Crohn’s disease and is independent of CARD15 and IBD5 Gastroenterol..

[13] Cadwell K, Liu JY, Brown SL, Miyoshi H, Loh J (2008). A key role for autophagy and the autophagy gene Atg16l1 in mouse and human intestinal Paneth cells. Nature..

[14] Cadwell K, Patel KK, Maloney NS, Liu TC, Ng AC (2010). Virus-plus susceptibility gene interaction determines Crohn’s disease gene Atg16L1 phenotypes in the intestine. Cell..

[15] Hamm C, Reimers M, McCullough C, Gorbe EB, Lu J (2010). NOD2 status and human ileal gene expression.. Inflamm Bowel Dis.

[16] Frank DN, St. Amand AL, Feldman RA, Boedeker EC, Harpaz N (2007). Molecular-phylogenetic characterization of microbial community imbalances in human inflammatory bowel diseases Proc Natl Acad Sci USA.

[17] Peterson DA, Frank DN, Pace N, Gordon JI (2008). Metagenomic approaches for defining the pathogenesis of inflammatory bowel diseases Cell Host Microbe.

[18] Sokol H, Lay C, Seksik P, Tannock GW (2008). Analysis of bacterial bowel communities of IBD patients: What has it revealed?. Inflamm Bowel Dis.

[19] Sokol H, Pigneur B, Watterlot L, Lakhdari O, Bermúdez-Humarán LG (2008). Faecalibacterium prausnitzii is an anti inflammatory commensal bacterium identified by gut microbiota analysis of Crohn disease patients Proc Natl Acad Sci USA.

[20] Willing B, Halfvarson WB, Dicksved J, Rosenquist M, Järnerot G (2009). Twin studies reveal specific imbalances in the mucosal-associated microbiota of patients with ileal Crohn’s disease Inflamm Bowel Dis.

[21] Qin J, Li R, Raes J, Arumugam M, Burgdorf KS (2009). A human gut microbial gene catalogue established by metagenomic sequencing Nature..

[22] Frank DN, Robertson CE, Hamm CM, Kpadeh Z, Zhang T (2011). Disease phenotype and genotype are associated with shifts in intestinal-microbiota in inflammatory bowel diseases Inflamm Bowel Dis.

[23] Willing B, Dicksved J, Halfvarson J, Andersson AF, Lucio M (2010). A pyrosequencing study in twins shows that gastrointestinal microbial profiles vary with inflammatory bowel disease phenotypes Gastroenterol.

[24] Li E, Hamm CM, Gulati AS, Sartor RB, Chen H (2011). Inflammatory bowel diseases phenotype, *C. difficile* and NOD2 genotype are associated with shifts in human ileum associated microbial composition PLoS ONE, accepted for publication..

[25] Frank DN, Zhu W, Sartor RB, Li E (2011). Investigating the biological and clinical significance of human dysbioses Trends Microbiol.

[26] Issa M, Vijaypal A, Graham MB, Beaulieu DB, Otterson MF (2007). Impact of Clostridium difficile on inflammatory bowel disease Clin Gastroenterol Hepatol.

[27] Rodemann JF, Dubberke ER, Reske KA, Seo da H, Stone CD (2007). The incidence of Clostridium difficile infection in inflammatory bowel disease. Clin Gastroenterol Hepatol..

[28] Nelson RL, Glenny AM, Song F (2009). Antimicrobial prophylaxis for colorectal surgery Cochrane Database Syst Rev.

[29] Tusher VG, Tibshirani R, Chu G (2001). Significance analysis of microarrays applied to the ionizing radiation response Proc Natl Acad Sci USA.

[30] Lawrance IC, Fiocchi C, Chakravarti S (2001). Ulcerative colitis and Crohn’s disease: distinctive gene expression profiles and novel susceptibility candidate genes Hum Mol Genet.

[31] Wu F, Dassopoulos T, Cope L, Maitra A, Brant SR (2007). Genome-wide gene expression differences in Crohn’s disease and ulcerative colitis from endoscopic pinch biopsies: insights into distinctive pathogenesis Inflamm Bowel Dis.

[32] Ward RF, Werner SL Analysis of variance of the composition of a migmatite (1963). Science.

[33] Singh W (2008). Robustness of three hierarchical agglomerative clustering techniques for ecological data Thesis for the degree of Master of Science in Environment and Natural Resources, Department of Mathematics, University of Iceland..

[34] Peterson J, Garges S, Giovanni M (2009). The NIH Human Microbiome Project Genome Res.

[35] Petrosino JF, Highlander S, Luna RA (2009). Metagenomic pyrosequencing and microbial identification Clin Chem.

[36] Ott RL, Longnecker M (2001). An introduction to statistical methods and data analysis Pacific Grove, CA Dyxbury..

[37] Yeung SM, Zhou F-C, Ye F-C, Pan H-Y, Gao S-H (2005). Early and sustained expression of latent and host modulating genes in coordinated transcriptional program of KSHV productive primary infection of human primary endothelial cells Virology..

[38] Vaughan IP, Omerod SJ (2005). Increasing the value of principal component analysis for simplifying ecological data: a case study for rivers and rive birds J Appl Ecol.

[39] Wehkamp J, Salzman NH, Porter E, Nuding S, Weichenthal M (2005). Reduced Paneth cell alpha-defensins in ileal Crohn’s disease Proc Natl Acad Sci USA.

[40] Simms LA, Doecke JD, Walsh MD, Huang N, Fowler EV (2008). Reduced alpha-defensin expression is associated with inflammation and not NOD2 mutation status in ileal Crohn’s disease Gut.

[41] Langmann T, Moehle C, Mauerer R, Scharl M, Liebisch G (2004). Loss of detoxification in inflammatory bowel disease: dysregulation of pregnane X receptor target genes Gastroenterol.

[42] Lepage P, Häsler R, Spehlmann ME, Rehman A, Zvirbliene A (2011). Twin study indicates loss of interaction between microbiota and mucosa of patients with ulcerative colitis Gastroenterol.

[43] Salzman NH, Hung K, Haribhai D (2011). Enteric defensins are essential regulators of intestinal microbial ecology Nat Immunol.

[44] Hooper LV, Wong MH, Thelin A, Hansson L, Falk PG (2001). Molecular analysis of commensal host-microbial relationships in the intestine Science.

[45] Dieckgraefe B, Stenson W, Korzenik JR, Swanson PE, Harrington CA (2000). Analysis of mucosal gene expression in inflammatory bowel disease by parallel oligonucleotide arrays Physiol Genomics.

[46] Hartupee JC, Zhang H, Bonaldo MF, Soares MB, Dieckgraefe BK (2001). Isolation and characterization of a cDNA encoding a novel member of the human regenerating protein family: Reg IV. Biochim Biophys Acta..

[47] Smyth GK, Speed TP (2003). Normalization of cDNA microarray data. Methods..

[48] Smyth GK (2005). Limma: linear models for microarray data. In Gentleman R, Carey V, Dudoit S, Irizarry R, Huber W eds. Bioinformatics and Computational Biology Solutions using R and Bioconductor.. New York: Springer.

[49] Eisen MB, Spellman PT, Brown PO, Botstein D (1998). Cluster analysis and display of genome-wide expression patterns. Proc Natl. Acad Sci USA..

[50] Sturn A, Quackenbush J, Trajanoski Z (2000). Genesis: cluster analysis of microarray data Bioinformatics.

[51] Sun J, Jia P, Fanous AH, van den Oord E (2010). Schizophrenia gene networks and pathways and their applications for novel candidate gene selection. PloS ONE..

[52] Nawrocki EP, Kolbe DL, Eddy SR (2009). Infernal 1.0: Inference of RNA alignments.. Bioinformatics.

[53] Haas BJ, Gevers D, Earl A, Feldgarden M, Ward DV (2011). Chimeric 16 S rRNA sequence formation and detection in Sanger and 454-pyrosequenced PCR amplicons Genome Res.

[54] Wang Q, Garrity GM, Tiedje JM, Cole JR (2007). Naive Bayesian classifier for rapid assignment of rRNA sequences into the new bacterial taxonomy Appl Environ Microbiol.

[55] Cole JR, Wang Q, Cardenas E, Fish J, Chai B (2009). The Ribosomal Database Project: improved alignments and new tools for rRNA analysis Nucleic Acids Res.

[56] DeSantis TZ, Hugenholtz P, Larsen N, Rojas M, Brodie EL (2006). Greengenes, a chimera-checked 16 S rRNA gene database and workbench compatible with ARB Appl Environ Microbiol.

[57] Collins MD, Lawson PA, Willems A, Cordoba JJ, Fernandez-Garayzabal J (1994). The phylogeny of the genus Clostridium: proposal of five new genera and eleven new species combinations.. Int J Syst Bacteriol.

[58] Anderson MJ (2001). A new method for non-parametric multivariate analysis of variance Austral Ecology.

[59] Aitchison J (1986). The Statistical Analysis of Compositional Data, Monographs on Statistics and Applied Probability.. London: Chapman & Hall Ltd.

