# Supplementary material for: Host Genes Related to Paneth Cells and Xenobiotic Metabolism Are Associated with Shifts in Human Ileum-Associated Microbial Composition
Source: PLoS One. 2012 Jun 13;7(6):e30044. doi: 10.1371/journal.pone.0030044 (PMC3374611; doi:10.1371/journal.pone.0030044)
Supplement: Table S1 — A. Gene-probes upregulated in CD compared to Control (n = 502). B. Gene-probes downregulated in CD compared to Control (n = 594). C. Gene-probes upregulated in UC compared to Control (n = 371). (DOC) [file pone.0030044.s001.doc]

| **Supplementary Table S1A. Gene-probes upregulated in CD compared to Control (n=502)** | | | |
| --- | --- | --- | --- |
| **Agilent ID** | **Gene Name or Accession Number** | **Fold Change** | **q-value** |
| A_32_P157391 | FOLH1B | 10.93 | <0.0001 |
| A_23_P47616 | [FOLH1](http://genome-www4.stanford.edu/cgi-bin/SMD/source/sourceResult?choice=Gene&option=Name&criteria=FOLH1) | 8.00 | <0.0001 |
| A_32_P178513 | [FOLH1](http://genome-www4.stanford.edu/cgi-bin/SMD/source/sourceResult?choice=Gene&option=Name&criteria=FOLH1) | 2.52 | <0.0001 |
| A_23_P20075 | [NPC1L1](http://genome-www4.stanford.edu/cgi-bin/SMD/source/sourceResult?choice=Gene&option=Name&criteria=NPC1L1) | 3.76 | <0.0001 |
| A_24_P140171 | [CRTAP](http://genome-www4.stanford.edu/cgi-bin/SMD/source/sourceResult?choice=Gene&option=Name&criteria=CRTAP) | 2.51 | <0.0001 |
| A_32_P127153 | [SORD](http://genome-www4.stanford.edu/cgi-bin/SMD/source/sourceResult?choice=Gene&option=Name&criteria=SORD) | 2.04 | <0.0001 |
| A_23_P155755 | [CXCL6](http://genome-www4.stanford.edu/cgi-bin/SMD/source/sourceResult?choice=Gene&option=Name&criteria=CXCL6) | 2.84 | <0.0001 |
| A_23_P380857 | [APOL4](http://genome-www4.stanford.edu/cgi-bin/SMD/source/sourceResult?choice=Gene&option=Name&criteria=APOL4) | 2.01 | <0.0001 |
| A_23_P19529 | [MLN](http://genome-www4.stanford.edu/cgi-bin/SMD/source/sourceResult?choice=Gene&option=Name&criteria=MLN) | 2.73 | <0.0001 |
| A_23_P86599 | [DMBT1](http://genome-www4.stanford.edu/cgi-bin/SMD/source/sourceResult?choice=Gene&option=Name&criteria=DMBT1) | 2.44 | <0.0001 |
| A_32_P95147 | [BC038559](http://genome-www4.stanford.edu/cgi-bin/SMD/source/sourceResult?choice=Gene&option=Name&criteria=BC038559) | 1.67 | <0.0001 |
| A_23_P112554 | [COL15A1](http://genome-www4.stanford.edu/cgi-bin/SMD/source/sourceResult?choice=Gene&option=Name&criteria=COL15A1) | 2.08 | <0.0001 |
| A_23_P211039 | [ADAMTS1](http://genome-www4.stanford.edu/cgi-bin/SMD/source/sourceResult?choice=Gene&option=Name&criteria=ADAMTS1) | 2.31 | <0.0001 |
| A_23_P13753 | [NFE2](http://genome-www4.stanford.edu/cgi-bin/SMD/source/sourceResult?choice=Gene&option=Name&criteria=NFE2) | 2.24 | <0.0001 |
| A_24_P944570 | [PXDN](http://genome-www4.stanford.edu/cgi-bin/SMD/source/sourceResult?choice=Gene&option=Name&criteria=PXDN) | 2.05 | <0.0001 |
| A_24_P92472 | [CFI](http://genome-www4.stanford.edu/cgi-bin/SMD/source/sourceResult?choice=Gene&option=Name&criteria=CFI) | 2.23 | <0.0001 |
| A_23_P200728 | [FCGR3A](http://genome-www4.stanford.edu/cgi-bin/SMD/source/sourceResult?choice=Gene&option=Name&criteria=FCGR3A) | 2.66 | <0.0001 |
| A_23_P169437 | [LCN2](http://genome-www4.stanford.edu/cgi-bin/SMD/source/sourceResult?choice=Gene&option=Name&criteria=LCN2) | 2.91 | <0.0001 |
| A_23_P19663 | [CTGF](http://genome-www4.stanford.edu/cgi-bin/SMD/source/sourceResult?choice=Gene&option=Name&criteria=CTGF) | 2.58 | <0.0001 |
| A_23_P133916 | [C2](http://genome-www4.stanford.edu/cgi-bin/SMD/source/sourceResult?choice=Gene&option=Name&criteria=C2) | 2.04 | <0.0001 |
| A_23_P7212 | [CFI](http://genome-www4.stanford.edu/cgi-bin/SMD/source/sourceResult?choice=Gene&option=Name&criteria=CFI) | 2.06 | <0.0001 |
| A_24_P389916 | [LRRC32](http://genome-www4.stanford.edu/cgi-bin/SMD/source/sourceResult?choice=Gene&option=Name&criteria=LRRC32) | 1.70 | <0.0001 |
| A_23_P502464 | [NOS2A](http://genome-www4.stanford.edu/cgi-bin/SMD/source/sourceResult?choice=Gene&option=Name&criteria=NOS2A) | 2.67 | <0.0001 |
| A_23_P19523 | [MLN](http://genome-www4.stanford.edu/cgi-bin/SMD/source/sourceResult?choice=Gene&option=Name&criteria=MLN) | 2.59 | <0.0001 |
| A_32_P162187 | [C2](http://genome-www4.stanford.edu/cgi-bin/SMD/source/sourceResult?choice=Gene&option=Name&criteria=C2) | 1.60 | <0.0001 |
| A_23_P65518 | [DACT1](http://genome-www4.stanford.edu/cgi-bin/SMD/source/sourceResult?choice=Gene&option=Name&criteria=DACT1) | 1.79 | <0.0001 |
| A_24_P416997 | [APOL3](http://genome-www4.stanford.edu/cgi-bin/SMD/source/sourceResult?choice=Gene&option=Name&criteria=APOL3) | 1.72 | <0.0001 |
| A_23_P7144 | [CXCL1](http://genome-www4.stanford.edu/cgi-bin/SMD/source/sourceResult?choice=Gene&option=Name&criteria=CXCL1) | 2.58 | <0.0001 |
| A_23_P31161 | [CPA2](http://genome-www4.stanford.edu/cgi-bin/SMD/source/sourceResult?choice=Gene&option=Name&criteria=CPA2) | 2.36 | <0.0001 |
| A_24_P370946 | [CYR61](http://genome-www4.stanford.edu/cgi-bin/SMD/source/sourceResult?choice=Gene&option=Name&criteria=CYR61) | 2.50 | <0.0001 |
| A_24_P101226 | [ENST00000354689](http://genome-www4.stanford.edu/cgi-bin/SMD/source/sourceResult?choice=Gene&option=Name&criteria=ENST00000354689) | 2.83 | <0.0001 |
| A_23_P383009 | [IGFBP5](http://genome-www4.stanford.edu/cgi-bin/SMD/source/sourceResult?choice=Gene&option=Name&criteria=IGFBP5) | 2.06 | <0.0001 |
| A_23_P211212 | [COL18A1](http://genome-www4.stanford.edu/cgi-bin/SMD/source/sourceResult?choice=Gene&option=Name&criteria=COL18A1) | 1.74 | <0.0001 |
| A_23_P434809 | [S100A8](http://genome-www4.stanford.edu/cgi-bin/SMD/source/sourceResult?choice=Gene&option=Name&criteria=S100A8) | 3.06 | <0.0001 |
| A_23_P395438 | [HTRA3](http://genome-www4.stanford.edu/cgi-bin/SMD/source/sourceResult?choice=Gene&option=Name&criteria=HTRA3) | 2.02 | <0.0001 |
| A_32_P162183 | [C2](http://genome-www4.stanford.edu/cgi-bin/SMD/source/sourceResult?choice=Gene&option=Name&criteria=C2) | 1.65 | <0.0001 |
| A_23_P211926 | [WNT5A](http://genome-www4.stanford.edu/cgi-bin/SMD/source/sourceResult?choice=Gene&option=Name&criteria=WNT5A) | 1.93 | <0.0001 |
| A_23_P111000 | [PSMB9](http://genome-www4.stanford.edu/cgi-bin/SMD/source/sourceResult?choice=Gene&option=Name&criteria=PSMB9) | 1.68 | <0.0001 |
| A_23_P119353 | [RASIP1](http://genome-www4.stanford.edu/cgi-bin/SMD/source/sourceResult?choice=Gene&option=Name&criteria=RASIP1) | 1.78 | <0.0001 |
| A_23_P428129 | [CDKN1C](http://genome-www4.stanford.edu/cgi-bin/SMD/source/sourceResult?choice=Gene&option=Name&criteria=CDKN1C) | 1.87 | <0.0001 |
| A_23_P342275 | [ADAMTS1](http://genome-www4.stanford.edu/cgi-bin/SMD/source/sourceResult?choice=Gene&option=Name&criteria=ADAMTS1) | 2.15 | <0.0001 |
| A_23_P64721 | [GPR109B](http://genome-www4.stanford.edu/cgi-bin/SMD/source/sourceResult?choice=Gene&option=Name&criteria=GPR109B) | 2.45 | <0.0001 |
| A_23_P149517 | [PIGR](http://genome-www4.stanford.edu/cgi-bin/SMD/source/sourceResult?choice=Gene&option=Name&criteria=PIGR) | 2.66 | <0.0001 |
| A_32_P100830 | [A_32_P100830](http://genome-www4.stanford.edu/cgi-bin/SMD/source/sourceResult?choice=Gene&option=Name&criteria=A_32_P100830) | 1.65 | <0.0001 |
| A_23_P46426 | [CYR61](http://genome-www4.stanford.edu/cgi-bin/SMD/source/sourceResult?choice=Gene&option=Name&criteria=CYR61) | 2.96 | <0.0001 |
| A_23_P46429 | [CYR61](http://genome-www4.stanford.edu/cgi-bin/SMD/source/sourceResult?choice=Gene&option=Name&criteria=CYR61) | 2.55 | <0.0001 |
| A_32_P74409 | [ENST00000339446](http://genome-www4.stanford.edu/cgi-bin/SMD/source/sourceResult?choice=Gene&option=Name&criteria=ENST00000339446) | 2.09 | <0.0001 |
| A_23_P23048 | [S100A9](http://genome-www4.stanford.edu/cgi-bin/SMD/source/sourceResult?choice=Gene&option=Name&criteria=S100A9) | 2.24 | <0.0001 |
| A_23_P20566 | [TPM2](http://genome-www4.stanford.edu/cgi-bin/SMD/source/sourceResult?choice=Gene&option=Name&criteria=TPM2) | 1.72 | <0.0001 |
| A_23_P207520 | [COL1A1](http://genome-www4.stanford.edu/cgi-bin/SMD/source/sourceResult?choice=Gene&option=Name&criteria=COL1A1) | 1.66 | <0.0001 |
| A_23_P38795 | [FPR1](http://genome-www4.stanford.edu/cgi-bin/SMD/source/sourceResult?choice=Gene&option=Name&criteria=FPR1) | 1.96 | <0.0001 |
| A_23_P106194 | [FOS](http://genome-www4.stanford.edu/cgi-bin/SMD/source/sourceResult?choice=Gene&option=Name&criteria=FOS) | 2.26 | <0.0001 |
| A_23_P151851 | [DUOX2](http://genome-www4.stanford.edu/cgi-bin/SMD/source/sourceResult?choice=Gene&option=Name&criteria=DUOX2) | 2.90 | <0.0001 |
| A_23_P255111 | [A_23_P255111](http://genome-www4.stanford.edu/cgi-bin/SMD/source/sourceResult?choice=Gene&option=Name&criteria=A_23_P255111) | 1.75 | <0.0001 |
| A_24_P281264 | [A_24_P281264](http://genome-www4.stanford.edu/cgi-bin/SMD/source/sourceResult?choice=Gene&option=Name&criteria=A_24_P281264) | 1.69 | <0.0001 |
| A_32_P327750 | [A_32_P327750](http://genome-www4.stanford.edu/cgi-bin/SMD/source/sourceResult?choice=Gene&option=Name&criteria=A_32_P327750) | 1.95 | <0.0001 |
| A_23_P158593 | [COL5A1](http://genome-www4.stanford.edu/cgi-bin/SMD/source/sourceResult?choice=Gene&option=Name&criteria=COL5A1) | 1.74 | <0.0001 |
| A_24_P100228 | [XBP1](http://genome-www4.stanford.edu/cgi-bin/SMD/source/sourceResult?choice=Gene&option=Name&criteria=XBP1) | 1.84 | <0.0001 |
| A_23_P79259 | [SH3BP4](http://genome-www4.stanford.edu/cgi-bin/SMD/source/sourceResult?choice=Gene&option=Name&criteria=SH3BP4) | 1.57 | <0.0001 |
| A_24_P298805 | [ENST00000360102](http://genome-www4.stanford.edu/cgi-bin/SMD/source/sourceResult?choice=Gene&option=Name&criteria=ENST00000360102) | 2.27 | <0.0001 |
| A_23_P106 | [USP33](http://genome-www4.stanford.edu/cgi-bin/SMD/source/sourceResult?choice=Gene&option=Name&criteria=USP33) | 1.77 | <0.0001 |
| A_23_P137856 | [MUC1](http://genome-www4.stanford.edu/cgi-bin/SMD/source/sourceResult?choice=Gene&option=Name&criteria=MUC1) | 2.17 | <0.0001 |
| A_24_P311917 | [BTN3A3](http://genome-www4.stanford.edu/cgi-bin/SMD/source/sourceResult?choice=Gene&option=Name&criteria=BTN3A3) | 1.55 | <0.0001 |
| A_24_P357809 | [C11orf17](http://genome-www4.stanford.edu/cgi-bin/SMD/source/sourceResult?choice=Gene&option=Name&criteria=C11orf17) | 1.51 | <0.0001 |
| A_24_P161853 | [LOC440361](http://genome-www4.stanford.edu/cgi-bin/SMD/source/sourceResult?choice=Gene&option=Name&criteria=LOC440361) | 2.53 | <0.0001 |
| A_24_P42264 | [LYZ](http://genome-www4.stanford.edu/cgi-bin/SMD/source/sourceResult?choice=Gene&option=Name&criteria=LYZ) | 2.59 | <0.0001 |
| A_24_P92683 | [IGHA1](http://genome-www4.stanford.edu/cgi-bin/SMD/source/sourceResult?choice=Gene&option=Name&criteria=IGHA1) | 2.57 | <0.0001 |
| A_23_P63390 | [LOC440607](http://genome-www4.stanford.edu/cgi-bin/SMD/source/sourceResult?choice=Gene&option=Name&criteria=LOC440607) | 2.23 | <0.0001 |
| A_23_P160559 | [ECM1](http://genome-www4.stanford.edu/cgi-bin/SMD/source/sourceResult?choice=Gene&option=Name&criteria=ECM1) | 1.65 | <0.0001 |
| A_24_P188218 | [MYL4](http://genome-www4.stanford.edu/cgi-bin/SMD/source/sourceResult?choice=Gene&option=Name&criteria=MYL4) | 1.83 | <0.0001 |
| A_23_P137097 | [SLC16A2](http://genome-www4.stanford.edu/cgi-bin/SMD/source/sourceResult?choice=Gene&option=Name&criteria=SLC16A2) | 1.88 | <0.0001 |
| A_32_P205624 | [ENST00000264554](http://genome-www4.stanford.edu/cgi-bin/SMD/source/sourceResult?choice=Gene&option=Name&criteria=ENST00000264554) | 1.59 | <0.0001 |
| A_23_P66635 | [CCL11](http://genome-www4.stanford.edu/cgi-bin/SMD/source/sourceResult?choice=Gene&option=Name&criteria=CCL11) | 1.97 | <0.0001 |
| A_24_P341126 | [A_24_P341126](http://genome-www4.stanford.edu/cgi-bin/SMD/source/sourceResult?choice=Gene&option=Name&criteria=A_24_P341126) | 2.39 | <0.0001 |
| A_24_P274270 | [STAT1](http://genome-www4.stanford.edu/cgi-bin/SMD/source/sourceResult?choice=Gene&option=Name&criteria=STAT1) | 1.61 | <0.0001 |
| A_24_P398585 | [UNG](http://genome-www4.stanford.edu/cgi-bin/SMD/source/sourceResult?choice=Gene&option=Name&criteria=UNG) | 1.68 | <0.0001 |
| A_24_P277934 | [COL1A2](http://genome-www4.stanford.edu/cgi-bin/SMD/source/sourceResult?choice=Gene&option=Name&criteria=COL1A2) | 1.71 | <0.0001 |
| A_24_P315941 | [D83692](http://genome-www4.stanford.edu/cgi-bin/SMD/source/sourceResult?choice=Gene&option=Name&criteria=D83692) | 2.68 | <0.0001 |
| A_24_P183664 | [KIAA0644](http://genome-www4.stanford.edu/cgi-bin/SMD/source/sourceResult?choice=Gene&option=Name&criteria=KIAA0644) | 1.61 | <0.0001 |
| A_23_P365267 | [SNED1](http://genome-www4.stanford.edu/cgi-bin/SMD/source/sourceResult?choice=Gene&option=Name&criteria=SNED1) | 1.56 | <0.0001 |
| A_23_P429998 | [FOSB](http://genome-www4.stanford.edu/cgi-bin/SMD/source/sourceResult?choice=Gene&option=Name&criteria=FOSB) | 2.40 | <0.0001 |
| A_23_P65388 | [CLEC14A](http://genome-www4.stanford.edu/cgi-bin/SMD/source/sourceResult?choice=Gene&option=Name&criteria=CLEC14A) | 1.60 | <0.0001 |
| A_24_P323298 | [LOC652159](http://genome-www4.stanford.edu/cgi-bin/SMD/source/sourceResult?choice=Gene&option=Name&criteria=LOC652159) | 2.48 | <0.0001 |
| A_24_P76868 | [X01147](http://genome-www4.stanford.edu/cgi-bin/SMD/source/sourceResult?choice=Gene&option=Name&criteria=X01147) | 2.15 | <0.0001 |
| A_32_P24832 | [OLFML3](http://genome-www4.stanford.edu/cgi-bin/SMD/source/sourceResult?choice=Gene&option=Name&criteria=OLFML3) | 1.57 | <0.0001 |
| A_23_P394304 | [PDZK1IP1](http://genome-www4.stanford.edu/cgi-bin/SMD/source/sourceResult?choice=Gene&option=Name&criteria=PDZK1IP1) | 1.89 | <0.0001 |
| A_23_P110712 | [DUSP1](http://genome-www4.stanford.edu/cgi-bin/SMD/source/sourceResult?choice=Gene&option=Name&criteria=DUSP1) | 1.76 | <0.0001 |
| A_24_P257416 | [CXCL2](http://genome-www4.stanford.edu/cgi-bin/SMD/source/sourceResult?choice=Gene&option=Name&criteria=CXCL2) | 1.80 | <0.0001 |
| A_24_P329065 | [BTN3A1](http://genome-www4.stanford.edu/cgi-bin/SMD/source/sourceResult?choice=Gene&option=Name&criteria=BTN3A1) | 1.53 | <0.0001 |
| A_23_P259292 | [C1QTNF5](http://genome-www4.stanford.edu/cgi-bin/SMD/source/sourceResult?choice=Gene&option=Name&criteria=C1QTNF5) | 1.51 | <0.0001 |
| A_23_P121533 | [SPON2](http://genome-www4.stanford.edu/cgi-bin/SMD/source/sourceResult?choice=Gene&option=Name&criteria=SPON2) | 1.73 | <0.0001 |
| A_23_P206899 | [TMEM159](http://genome-www4.stanford.edu/cgi-bin/SMD/source/sourceResult?choice=Gene&option=Name&criteria=TMEM159) | 1.66 | <0.0001 |
| A_24_P156113 | [EHD2](http://genome-www4.stanford.edu/cgi-bin/SMD/source/sourceResult?choice=Gene&option=Name&criteria=EHD2) | 1.61 | <0.0001 |
| A_23_P144959 | [CSPG2](http://genome-www4.stanford.edu/cgi-bin/SMD/source/sourceResult?choice=Gene&option=Name&criteria=CSPG2) | 1.52 | <0.0001 |
| A_24_P11315 | [OLFML3](http://genome-www4.stanford.edu/cgi-bin/SMD/source/sourceResult?choice=Gene&option=Name&criteria=OLFML3) | 1.68 | <0.0001 |
| A_23_P202837 | [CCND1](http://genome-www4.stanford.edu/cgi-bin/SMD/source/sourceResult?choice=Gene&option=Name&criteria=CCND1) | 1.75 | <0.0001 |
| A_24_P852001 | [ENST00000383048](http://genome-www4.stanford.edu/cgi-bin/SMD/source/sourceResult?choice=Gene&option=Name&criteria=ENST00000383048) | 2.48 | <0.0001 |
| A_23_P15727 | [FKBP10](http://genome-www4.stanford.edu/cgi-bin/SMD/source/sourceResult?choice=Gene&option=Name&criteria=FKBP10) | 1.88 | <0.0001 |
| A_24_P296772 | [PPP1R14A](http://genome-www4.stanford.edu/cgi-bin/SMD/source/sourceResult?choice=Gene&option=Name&criteria=PPP1R14A) | 1.62 | <0.0001 |
| A_32_P41254 | [C6orf117](http://genome-www4.stanford.edu/cgi-bin/SMD/source/sourceResult?choice=Gene&option=Name&criteria=C6orf117) | 1.57 | <0.0001 |
| A_24_P590547 | [IGHA1](http://genome-www4.stanford.edu/cgi-bin/SMD/source/sourceResult?choice=Gene&option=Name&criteria=IGHA1) | 2.46 | <0.0001 |
| A_32_P190864 | [A_32_P190864](http://genome-www4.stanford.edu/cgi-bin/SMD/source/sourceResult?choice=Gene&option=Name&criteria=A_32_P190864) | 1.55 | <0.0001 |
| A_24_P291814 | [COL12A1](http://genome-www4.stanford.edu/cgi-bin/SMD/source/sourceResult?choice=Gene&option=Name&criteria=COL12A1) | 1.84 | <0.0001 |
| A_23_P112957 | [A_23_P112957](http://genome-www4.stanford.edu/cgi-bin/SMD/source/sourceResult?choice=Gene&option=Name&criteria=A_23_P112957) | 1.93 | <0.0001 |
| A_23_P78342 | [LMAN1](http://genome-www4.stanford.edu/cgi-bin/SMD/source/sourceResult?choice=Gene&option=Name&criteria=LMAN1) | 1.56 | <0.0001 |
| A_23_P365614 | [NOTCH4](http://genome-www4.stanford.edu/cgi-bin/SMD/source/sourceResult?choice=Gene&option=Name&criteria=NOTCH4) | 1.54 | <0.0001 |
| A_24_P698136 | [LOC344967](http://genome-www4.stanford.edu/cgi-bin/SMD/source/sourceResult?choice=Gene&option=Name&criteria=LOC344967) | 1.63 | <0.0001 |
| A_24_P826046 | [AK024177](http://genome-www4.stanford.edu/cgi-bin/SMD/source/sourceResult?choice=Gene&option=Name&criteria=AK024177) | 1.57 | <0.0001 |
| A_32_P56001 | [CD93](http://genome-www4.stanford.edu/cgi-bin/SMD/source/sourceResult?choice=Gene&option=Name&criteria=CD93) | 1.65 | <0.0001 |
| A_23_P62890 | [GBP1](http://genome-www4.stanford.edu/cgi-bin/SMD/source/sourceResult?choice=Gene&option=Name&criteria=GBP1) | 1.82 | <0.0001 |
| A_23_P166408 | [OSM](http://genome-www4.stanford.edu/cgi-bin/SMD/source/sourceResult?choice=Gene&option=Name&criteria=OSM) | 2.01 | <0.0001 |
| A_24_P889462 | [IGHA1](http://genome-www4.stanford.edu/cgi-bin/SMD/source/sourceResult?choice=Gene&option=Name&criteria=IGHA1) | 2.32 | <0.0001 |
| A_23_P257144 | [PXDN](http://genome-www4.stanford.edu/cgi-bin/SMD/source/sourceResult?choice=Gene&option=Name&criteria=PXDN) | 1.60 | <0.0001 |
| A_23_P151805 | [FBLN5](http://genome-www4.stanford.edu/cgi-bin/SMD/source/sourceResult?choice=Gene&option=Name&criteria=FBLN5) | 1.57 | <0.0001 |
| A_24_P110242 | [ENST00000360102](http://genome-www4.stanford.edu/cgi-bin/SMD/source/sourceResult?choice=Gene&option=Name&criteria=ENST00000360102) | 2.56 | <0.0001 |
| A_24_P53976 | [GLUL](http://genome-www4.stanford.edu/cgi-bin/SMD/source/sourceResult?choice=Gene&option=Name&criteria=GLUL) | 1.65 | <0.0001 |
| A_24_P204727 | [Z18824](http://genome-www4.stanford.edu/cgi-bin/SMD/source/sourceResult?choice=Gene&option=Name&criteria=Z18824) | 2.17 | <0.0001 |
| A_32_P331700 | [A_32_P331700](http://genome-www4.stanford.edu/cgi-bin/SMD/source/sourceResult?choice=Gene&option=Name&criteria=A_32_P331700) | 1.70 | <0.0001 |
| A_23_P52425 | [NKX2-3](http://genome-www4.stanford.edu/cgi-bin/SMD/source/sourceResult?choice=Gene&option=Name&criteria=NKX2-3) | 1.66 | <0.0001 |
| A_23_P24104 | [PLAU](http://genome-www4.stanford.edu/cgi-bin/SMD/source/sourceResult?choice=Gene&option=Name&criteria=PLAU) | 1.66 | <0.0001 |
| A_24_P384604 | [A_24_P384604](http://genome-www4.stanford.edu/cgi-bin/SMD/source/sourceResult?choice=Gene&option=Name&criteria=A_24_P384604) | 2.24 | <0.0001 |
| A_24_P46066 | [CXCR6](http://genome-www4.stanford.edu/cgi-bin/SMD/source/sourceResult?choice=Gene&option=Name&criteria=CXCR6) | 1.84 | <0.0001 |
| A_24_P7040 | [A_24_P7040](http://genome-www4.stanford.edu/cgi-bin/SMD/source/sourceResult?choice=Gene&option=Name&criteria=A_24_P7040) | 1.59 | <0.0001 |
| A_23_P1759 | [AMICA1](http://genome-www4.stanford.edu/cgi-bin/SMD/source/sourceResult?choice=Gene&option=Name&criteria=AMICA1) | 1.59 | <0.0001 |
| A_24_P24053 | [ENST00000360623](http://genome-www4.stanford.edu/cgi-bin/SMD/source/sourceResult?choice=Gene&option=Name&criteria=ENST00000360623) | 2.50 | <0.0001 |
| A_23_P432573 | [MRGPRF](http://genome-www4.stanford.edu/cgi-bin/SMD/source/sourceResult?choice=Gene&option=Name&criteria=MRGPRF) | 1.63 | <0.0001 |
| A_32_P234405 | [AI916628](http://genome-www4.stanford.edu/cgi-bin/SMD/source/sourceResult?choice=Gene&option=Name&criteria=AI916628) | 1.83 | <0.0001 |
| A_23_P82775 | [SOX17](http://genome-www4.stanford.edu/cgi-bin/SMD/source/sourceResult?choice=Gene&option=Name&criteria=SOX17) | 1.76 | <0.0001 |
| A_23_P433016 | [FBLN1](http://genome-www4.stanford.edu/cgi-bin/SMD/source/sourceResult?choice=Gene&option=Name&criteria=FBLN1) | 1.65 | <0.0001 |
| A_23_P126782 | [F3](http://genome-www4.stanford.edu/cgi-bin/SMD/source/sourceResult?choice=Gene&option=Name&criteria=F3) | 1.77 | <0.0001 |
| A_23_P96641 | [PRPS2](http://genome-www4.stanford.edu/cgi-bin/SMD/source/sourceResult?choice=Gene&option=Name&criteria=PRPS2) | 1.70 | <0.0001 |
| A_24_P287043 | [IFITM2](http://genome-www4.stanford.edu/cgi-bin/SMD/source/sourceResult?choice=Gene&option=Name&criteria=IFITM2) | 1.57 | <0.0001 |
| A_24_P247749 | [RAB21](http://genome-www4.stanford.edu/cgi-bin/SMD/source/sourceResult?choice=Gene&option=Name&criteria=RAB21) | 1.90 | <0.0001 |
| A_23_P106617 | [WFDC1](http://genome-www4.stanford.edu/cgi-bin/SMD/source/sourceResult?choice=Gene&option=Name&criteria=WFDC1) | 1.56 | <0.0001 |
| A_23_P77103 | [SORD](http://genome-www4.stanford.edu/cgi-bin/SMD/source/sourceResult?choice=Gene&option=Name&criteria=SORD) | 1.72 | <0.0001 |
| A_23_P40453 | [CBR3](http://genome-www4.stanford.edu/cgi-bin/SMD/source/sourceResult?choice=Gene&option=Name&criteria=CBR3) | 1.52 | <0.0001 |
| A_24_P33895 | [ATF3](http://genome-www4.stanford.edu/cgi-bin/SMD/source/sourceResult?choice=Gene&option=Name&criteria=ATF3) | 1.92 | <0.0001 |
| A_23_P163787 | [MMP2](http://genome-www4.stanford.edu/cgi-bin/SMD/source/sourceResult?choice=Gene&option=Name&criteria=MMP2) | 1.81 | <0.0001 |
| A_23_P21249 | [AB063751](http://genome-www4.stanford.edu/cgi-bin/SMD/source/sourceResult?choice=Gene&option=Name&criteria=AB063751) | 2.42 | <0.0001 |
| A_23_P106602 | [CRISPLD2](http://genome-www4.stanford.edu/cgi-bin/SMD/source/sourceResult?choice=Gene&option=Name&criteria=CRISPLD2) | 1.52 | <0.0001 |
| A_24_P110487 | [A_24_P110487](http://genome-www4.stanford.edu/cgi-bin/SMD/source/sourceResult?choice=Gene&option=Name&criteria=A_24_P110487) | 2.24 | <0.0001 |
| A_23_P315364 | [CXCL2](http://genome-www4.stanford.edu/cgi-bin/SMD/source/sourceResult?choice=Gene&option=Name&criteria=CXCL2) | 2.31 | <0.0001 |
| A_23_P205389 | [MOAP1](http://genome-www4.stanford.edu/cgi-bin/SMD/source/sourceResult?choice=Gene&option=Name&criteria=MOAP1) | 1.57 | <0.0001 |
| A_24_P215653 | [CLEC14A](http://genome-www4.stanford.edu/cgi-bin/SMD/source/sourceResult?choice=Gene&option=Name&criteria=CLEC14A) | 1.59 | <0.0001 |
| A_23_P51926 | [PTAFR](http://genome-www4.stanford.edu/cgi-bin/SMD/source/sourceResult?choice=Gene&option=Name&criteria=PTAFR) | 1.58 | <0.0001 |
| A_24_P414999 | [LAPTM4B](http://genome-www4.stanford.edu/cgi-bin/SMD/source/sourceResult?choice=Gene&option=Name&criteria=LAPTM4B) | 1.75 | <0.0001 |
| A_24_P263786 | [BC022362](http://genome-www4.stanford.edu/cgi-bin/SMD/source/sourceResult?choice=Gene&option=Name&criteria=BC022362) | 2.22 | <0.0001 |
| A_32_P157671 | [A_32_P157671](http://genome-www4.stanford.edu/cgi-bin/SMD/source/sourceResult?choice=Gene&option=Name&criteria=A_32_P157671) | 1.96 | <0.0001 |
| A_23_P395001 | [SLC2A12](http://genome-www4.stanford.edu/cgi-bin/SMD/source/sourceResult?choice=Gene&option=Name&criteria=SLC2A12) | 2.09 | <0.0001 |
| A_23_P344421 | [ROBO4](http://genome-www4.stanford.edu/cgi-bin/SMD/source/sourceResult?choice=Gene&option=Name&criteria=ROBO4) | 1.56 | <0.0001 |
| A_24_P15550 | [AF035034](http://genome-www4.stanford.edu/cgi-bin/SMD/source/sourceResult?choice=Gene&option=Name&criteria=AF035034) | 2.04 | <0.0001 |
| A_24_P33341 | [ENST00000360102](http://genome-www4.stanford.edu/cgi-bin/SMD/source/sourceResult?choice=Gene&option=Name&criteria=ENST00000360102) | 2.35 | <0.0001 |
| A_24_P100684 | [ENST00000379895](http://genome-www4.stanford.edu/cgi-bin/SMD/source/sourceResult?choice=Gene&option=Name&criteria=ENST00000379895) | 2.37 | <0.0001 |
| A_23_P147106 | [FLJ20245](http://genome-www4.stanford.edu/cgi-bin/SMD/source/sourceResult?choice=Gene&option=Name&criteria=FLJ20245) | 1.53 | <0.0001 |
| A_23_P55828 | [CCL25](http://genome-www4.stanford.edu/cgi-bin/SMD/source/sourceResult?choice=Gene&option=Name&criteria=CCL25) | 2.32 | <0.0001 |
| A_23_P436259 | [ERN1](http://genome-www4.stanford.edu/cgi-bin/SMD/source/sourceResult?choice=Gene&option=Name&criteria=ERN1) | 1.56 | <0.0001 |
| A_23_P87545 | [IFITM3](http://genome-www4.stanford.edu/cgi-bin/SMD/source/sourceResult?choice=Gene&option=Name&criteria=IFITM3) | 1.54 | <0.0001 |
| A_24_P94034 | [USP22](http://genome-www4.stanford.edu/cgi-bin/SMD/source/sourceResult?choice=Gene&option=Name&criteria=USP22) | 1.55 | <0.0001 |
| A_32_P167592 | [ENST00000339867](http://genome-www4.stanford.edu/cgi-bin/SMD/source/sourceResult?choice=Gene&option=Name&criteria=ENST00000339867) | 1.57 | <0.0001 |
| A_23_P2990 | [CEBPE](http://genome-www4.stanford.edu/cgi-bin/SMD/source/sourceResult?choice=Gene&option=Name&criteria=CEBPE) | 1.63 | <0.0001 |
| A_23_P136173 | [CSF2RA](http://genome-www4.stanford.edu/cgi-bin/SMD/source/sourceResult?choice=Gene&option=Name&criteria=CSF2RA) | 1.69 | <0.0001 |
| A_23_P9997 | [A_23_P9997](http://genome-www4.stanford.edu/cgi-bin/SMD/source/sourceResult?choice=Gene&option=Name&criteria=A_23_P9997) | 1.86 | <0.0001 |
| A_23_P156687 | [CFB](http://genome-www4.stanford.edu/cgi-bin/SMD/source/sourceResult?choice=Gene&option=Name&criteria=CFB) | 1.80 | <0.0001 |
| A_24_P764690 | [CR611323](http://genome-www4.stanford.edu/cgi-bin/SMD/source/sourceResult?choice=Gene&option=Name&criteria=CR611323) | 1.66 | <0.0001 |
| A_23_P357207 | [C6orf117](http://genome-www4.stanford.edu/cgi-bin/SMD/source/sourceResult?choice=Gene&option=Name&criteria=C6orf117) | 1.58 | <0.0001 |
| A_23_P71316 | [RBPMS](http://genome-www4.stanford.edu/cgi-bin/SMD/source/sourceResult?choice=Gene&option=Name&criteria=RBPMS) | 1.67 | <0.0001 |
| A_24_P229447 | [ENST00000360102](http://genome-www4.stanford.edu/cgi-bin/SMD/source/sourceResult?choice=Gene&option=Name&criteria=ENST00000360102) | 2.36 | <0.0001 |
| A_23_P353035 | [IGFBP7](http://genome-www4.stanford.edu/cgi-bin/SMD/source/sourceResult?choice=Gene&option=Name&criteria=IGFBP7) | 1.67 | <0.0001 |
| A_24_P411561 | [HAVCR2](http://genome-www4.stanford.edu/cgi-bin/SMD/source/sourceResult?choice=Gene&option=Name&criteria=HAVCR2) | 1.57 | <0.0001 |
| A_24_P49267 | [A_24_P49267](http://genome-www4.stanford.edu/cgi-bin/SMD/source/sourceResult?choice=Gene&option=Name&criteria=A_24_P49267) | 1.59 | <0.0001 |
| A_24_P488083 | [AJ519285](http://genome-www4.stanford.edu/cgi-bin/SMD/source/sourceResult?choice=Gene&option=Name&criteria=AJ519285) | 2.30 | <0.0001 |
| A_24_P144346 | [Y11328](http://genome-www4.stanford.edu/cgi-bin/SMD/source/sourceResult?choice=Gene&option=Name&criteria=Y11328) | 2.32 | <0.0001 |
| A_23_P39465 | [BST2](http://genome-www4.stanford.edu/cgi-bin/SMD/source/sourceResult?choice=Gene&option=Name&criteria=BST2) | 1.80 | <0.0001 |
| A_23_P379475 | [DHCR24](http://genome-www4.stanford.edu/cgi-bin/SMD/source/sourceResult?choice=Gene&option=Name&criteria=DHCR24) | 1.52 | <0.0001 |
| A_23_P156890 | [TCF21](http://genome-www4.stanford.edu/cgi-bin/SMD/source/sourceResult?choice=Gene&option=Name&criteria=TCF21) | 1.76 | <0.0001 |
| A_24_P101642 | [LOC390712](http://genome-www4.stanford.edu/cgi-bin/SMD/source/sourceResult?choice=Gene&option=Name&criteria=LOC390712) | 2.12 | <0.0001 |
| A_24_P417352 | [IGHM](http://genome-www4.stanford.edu/cgi-bin/SMD/source/sourceResult?choice=Gene&option=Name&criteria=IGHM) | 1.98 | <0.0001 |
| A_23_P111701 | [GNG11](http://genome-www4.stanford.edu/cgi-bin/SMD/source/sourceResult?choice=Gene&option=Name&criteria=GNG11) | 1.55 | <0.0001 |
| A_24_P702749 | [AF471454](http://genome-www4.stanford.edu/cgi-bin/SMD/source/sourceResult?choice=Gene&option=Name&criteria=AF471454) | 2.28 | <0.0001 |
| A_24_P384119 | [LOC652791](http://genome-www4.stanford.edu/cgi-bin/SMD/source/sourceResult?choice=Gene&option=Name&criteria=LOC652791) | 2.33 | <0.0001 |
| A_24_P472081 | [ENST00000331195](http://genome-www4.stanford.edu/cgi-bin/SMD/source/sourceResult?choice=Gene&option=Name&criteria=ENST00000331195) | 1.88 | <0.0001 |
| A_23_P50175 | [SIGLEC7](http://genome-www4.stanford.edu/cgi-bin/SMD/source/sourceResult?choice=Gene&option=Name&criteria=SIGLEC7) | 1.52 | <0.0001 |
| A_23_P108673 | [FLJ13391](http://genome-www4.stanford.edu/cgi-bin/SMD/source/sourceResult?choice=Gene&option=Name&criteria=FLJ13391) | 1.54 | <0.0001 |
| A_32_P51988 | [ENST00000379879](http://genome-www4.stanford.edu/cgi-bin/SMD/source/sourceResult?choice=Gene&option=Name&criteria=ENST00000379879) | 2.12 | <0.0001 |
| A_23_P10121 | [SFRP1](http://genome-www4.stanford.edu/cgi-bin/SMD/source/sourceResult?choice=Gene&option=Name&criteria=SFRP1) | 1.77 | <0.0001 |
| A_24_P604784 | [AF103312](http://genome-www4.stanford.edu/cgi-bin/SMD/source/sourceResult?choice=Gene&option=Name&criteria=AF103312) | 2.05 | <0.0001 |
| A_23_P159435 | [BC107852](http://genome-www4.stanford.edu/cgi-bin/SMD/source/sourceResult?choice=Gene&option=Name&criteria=BC107852) | 2.20 | <0.0001 |
| A_23_P203475 | [PRKCDBP](http://genome-www4.stanford.edu/cgi-bin/SMD/source/sourceResult?choice=Gene&option=Name&criteria=PRKCDBP) | 1.56 | <0.0001 |
| A_23_P376449 | [A_23_P376449](http://genome-www4.stanford.edu/cgi-bin/SMD/source/sourceResult?choice=Gene&option=Name&criteria=A_23_P376449) | 1.52 | <0.0001 |
| A_23_P203957 | [TMTC1](http://genome-www4.stanford.edu/cgi-bin/SMD/source/sourceResult?choice=Gene&option=Name&criteria=TMTC1) | 1.53 | <0.0001 |
| A_23_P258136 | [MXRA5](http://genome-www4.stanford.edu/cgi-bin/SMD/source/sourceResult?choice=Gene&option=Name&criteria=MXRA5) | 1.61 | <0.0001 |
| A_23_P206661 | [NQO1](http://genome-www4.stanford.edu/cgi-bin/SMD/source/sourceResult?choice=Gene&option=Name&criteria=NQO1) | 1.73 | <0.0001 |
| A_23_P126593 | [S100A11](http://genome-www4.stanford.edu/cgi-bin/SMD/source/sourceResult?choice=Gene&option=Name&criteria=S100A11) | 1.58 | <0.0001 |
| A_23_P321949 | [PLA2G2A](http://genome-www4.stanford.edu/cgi-bin/SMD/source/sourceResult?choice=Gene&option=Name&criteria=PLA2G2A) | 1.99 | <0.0001 |
| A_24_P15388 | [BC024289](http://genome-www4.stanford.edu/cgi-bin/SMD/source/sourceResult?choice=Gene&option=Name&criteria=BC024289) | 2.21 | <0.0001 |
| A_24_P917316 | [AF035790](http://genome-www4.stanford.edu/cgi-bin/SMD/source/sourceResult?choice=Gene&option=Name&criteria=AF035790) | 1.74 | <0.0001 |
| A_23_P7562 | [ACSL6](http://genome-www4.stanford.edu/cgi-bin/SMD/source/sourceResult?choice=Gene&option=Name&criteria=ACSL6) | 1.64 | <0.0001 |
| A_23_P250607 | [PLS3](http://genome-www4.stanford.edu/cgi-bin/SMD/source/sourceResult?choice=Gene&option=Name&criteria=PLS3) | 1.61 | <0.0001 |
| A_23_P34915 | [ATF3](http://genome-www4.stanford.edu/cgi-bin/SMD/source/sourceResult?choice=Gene&option=Name&criteria=ATF3) | 2.12 | <0.0001 |
| A_23_P207058 | [SOCS3](http://genome-www4.stanford.edu/cgi-bin/SMD/source/sourceResult?choice=Gene&option=Name&criteria=SOCS3) | 2.08 | 0.0011 |
| A_23_P33196 | [COL5A2](http://genome-www4.stanford.edu/cgi-bin/SMD/source/sourceResult?choice=Gene&option=Name&criteria=COL5A2) | 1.63 | 0.0011 |
| A_24_P868905 | [A_24_P868905](http://genome-www4.stanford.edu/cgi-bin/SMD/source/sourceResult?choice=Gene&option=Name&criteria=A_24_P868905) | 1.54 | 0.0011 |
| A_23_P37702 | [TPSB2](http://genome-www4.stanford.edu/cgi-bin/SMD/source/sourceResult?choice=Gene&option=Name&criteria=TPSB2) | 1.90 | 0.0011 |
| A_24_P750327 | [AJ245002](http://genome-www4.stanford.edu/cgi-bin/SMD/source/sourceResult?choice=Gene&option=Name&criteria=AJ245002) | 2.18 | 0.0011 |
| A_23_P13222 | [RCN1](http://genome-www4.stanford.edu/cgi-bin/SMD/source/sourceResult?choice=Gene&option=Name&criteria=RCN1) | 1.54 | 0.0011 |
| A_24_P15610 | [A_24_P15610](http://genome-www4.stanford.edu/cgi-bin/SMD/source/sourceResult?choice=Gene&option=Name&criteria=A_24_P15610) | 1.63 | 0.0011 |
| A_24_P212024 | [ENST00000259219](http://genome-www4.stanford.edu/cgi-bin/SMD/source/sourceResult?choice=Gene&option=Name&criteria=ENST00000259219) | 2.06 | 0.0011 |
| A_23_P120103 | [KCNS3](http://genome-www4.stanford.edu/cgi-bin/SMD/source/sourceResult?choice=Gene&option=Name&criteria=KCNS3) | 1.78 | 0.0011 |
| A_24_P204574 | [A_24_P204574](http://genome-www4.stanford.edu/cgi-bin/SMD/source/sourceResult?choice=Gene&option=Name&criteria=A_24_P204574) | 1.88 | 0.0011 |
| A_23_P216501 | [TPM2](http://genome-www4.stanford.edu/cgi-bin/SMD/source/sourceResult?choice=Gene&option=Name&criteria=TPM2) | 1.51 | 0.0011 |
| A_32_P722809 | [IGKV1-5](http://genome-www4.stanford.edu/cgi-bin/SMD/source/sourceResult?choice=Gene&option=Name&criteria=IGKV1-5) | 2.01 | 0.0011 |
| A_32_P220798 | [CD34](http://genome-www4.stanford.edu/cgi-bin/SMD/source/sourceResult?choice=Gene&option=Name&criteria=CD34) | 1.67 | 0.0011 |
| A_23_P17481 | [SIGLEC1](http://genome-www4.stanford.edu/cgi-bin/SMD/source/sourceResult?choice=Gene&option=Name&criteria=SIGLEC1) | 1.51 | 0.0011 |
| A_32_P70158 | [LILRB3](http://genome-www4.stanford.edu/cgi-bin/SMD/source/sourceResult?choice=Gene&option=Name&criteria=LILRB3) | 1.88 | 0.0011 |
| A_24_P915692 | [PHLDA1](http://genome-www4.stanford.edu/cgi-bin/SMD/source/sourceResult?choice=Gene&option=Name&criteria=PHLDA1) | 1.59 | 0.0011 |
| A_23_P165848 | [EMILIN1](http://genome-www4.stanford.edu/cgi-bin/SMD/source/sourceResult?choice=Gene&option=Name&criteria=EMILIN1) | 1.73 | 0.0011 |
| A_32_P221966 | [AY102069](http://genome-www4.stanford.edu/cgi-bin/SMD/source/sourceResult?choice=Gene&option=Name&criteria=AY102069) | 1.59 | 0.0011 |
| A_24_P402242 | [COL3A1](http://genome-www4.stanford.edu/cgi-bin/SMD/source/sourceResult?choice=Gene&option=Name&criteria=COL3A1) | 1.90 | 0.0011 |
| A_24_P76210 | [THC2365247](http://genome-www4.stanford.edu/cgi-bin/SMD/source/sourceResult?choice=Gene&option=Name&criteria=THC2365247) | 1.63 | 0.0011 |
| A_23_P156957 | [NCOA7](http://genome-www4.stanford.edu/cgi-bin/SMD/source/sourceResult?choice=Gene&option=Name&criteria=NCOA7) | 1.58 | 0.0011 |
| A_23_P259763 | [AF471475](http://genome-www4.stanford.edu/cgi-bin/SMD/source/sourceResult?choice=Gene&option=Name&criteria=AF471475) | 2.38 | 0.0011 |
| A_23_P133474 | [GPX3](http://genome-www4.stanford.edu/cgi-bin/SMD/source/sourceResult?choice=Gene&option=Name&criteria=GPX3) | 1.58 | 0.0011 |
| A_24_P273679 | [YAP1](http://genome-www4.stanford.edu/cgi-bin/SMD/source/sourceResult?choice=Gene&option=Name&criteria=YAP1) | 2.06 | 0.0011 |
| A_23_P331748 | [CD33](http://genome-www4.stanford.edu/cgi-bin/SMD/source/sourceResult?choice=Gene&option=Name&criteria=CD33) | 1.55 | 0.0011 |
| A_23_P96191 | [AY998685](http://genome-www4.stanford.edu/cgi-bin/SMD/source/sourceResult?choice=Gene&option=Name&criteria=AY998685) | 1.95 | 0.0011 |
| A_23_P24948 | [KCNE3](http://genome-www4.stanford.edu/cgi-bin/SMD/source/sourceResult?choice=Gene&option=Name&criteria=KCNE3) | 1.65 | 0.0011 |
| A_23_P61068 | [A_23_P61068](http://genome-www4.stanford.edu/cgi-bin/SMD/source/sourceResult?choice=Gene&option=Name&criteria=A_23_P61068) | 1.87 | 0.0011 |
| A_23_P373126 | [BC035647](http://genome-www4.stanford.edu/cgi-bin/SMD/source/sourceResult?choice=Gene&option=Name&criteria=BC035647) | 1.54 | 0.0011 |
| A_23_P5983 | [PLTP](http://genome-www4.stanford.edu/cgi-bin/SMD/source/sourceResult?choice=Gene&option=Name&criteria=PLTP) | 1.57 | 0.0011 |
| A_23_P87013 | [TAGLN](http://genome-www4.stanford.edu/cgi-bin/SMD/source/sourceResult?choice=Gene&option=Name&criteria=TAGLN) | 1.58 | 0.0011 |
| A_23_P124632 | [IGHA1](http://genome-www4.stanford.edu/cgi-bin/SMD/source/sourceResult?choice=Gene&option=Name&criteria=IGHA1) | 2.05 | 0.0011 |
| A_23_P50638 | [LRG1](http://genome-www4.stanford.edu/cgi-bin/SMD/source/sourceResult?choice=Gene&option=Name&criteria=LRG1) | 1.61 | 0.0011 |
| A_23_P350782 | [ENST00000307840](http://genome-www4.stanford.edu/cgi-bin/SMD/source/sourceResult?choice=Gene&option=Name&criteria=ENST00000307840) | 1.88 | 0.0011 |
| A_23_P145264 | [HLA-F](http://genome-www4.stanford.edu/cgi-bin/SMD/source/sourceResult?choice=Gene&option=Name&criteria=HLA-F) | 1.52 | 0.0011 |
| A_23_P147109 | [FLJ20245](http://genome-www4.stanford.edu/cgi-bin/SMD/source/sourceResult?choice=Gene&option=Name&criteria=FLJ20245) | 1.81 | 0.0011 |
| A_24_P306905 | [LOC651928](http://genome-www4.stanford.edu/cgi-bin/SMD/source/sourceResult?choice=Gene&option=Name&criteria=LOC651928) | 1.77 | 0.0011 |
| A_23_P380754 | [PRSS1](http://genome-www4.stanford.edu/cgi-bin/SMD/source/sourceResult?choice=Gene&option=Name&criteria=PRSS1) | 1.57 | 0.0011 |
| A_23_P426305 | [AOC3](http://genome-www4.stanford.edu/cgi-bin/SMD/source/sourceResult?choice=Gene&option=Name&criteria=AOC3) | 1.57 | 0.0011 |
| A_24_P304449 | [KIAA0152](http://genome-www4.stanford.edu/cgi-bin/SMD/source/sourceResult?choice=Gene&option=Name&criteria=KIAA0152) | 1.55 | 0.0011 |
| A_23_P73328 | [AY003763](http://genome-www4.stanford.edu/cgi-bin/SMD/source/sourceResult?choice=Gene&option=Name&criteria=AY003763) | 2.14 | 0.0011 |
| A_23_P89431 | [CCL2](http://genome-www4.stanford.edu/cgi-bin/SMD/source/sourceResult?choice=Gene&option=Name&criteria=CCL2) | 1.74 | 0.0011 |
| A_23_P44053 | [A_23_P44053](http://genome-www4.stanford.edu/cgi-bin/SMD/source/sourceResult?choice=Gene&option=Name&criteria=A_23_P44053) | 2.06 | 0.0011 |
| A_24_P261417 | [DKK3](http://genome-www4.stanford.edu/cgi-bin/SMD/source/sourceResult?choice=Gene&option=Name&criteria=DKK3) | 1.53 | 0.0011 |
| A_24_P222655 | [C1QA](http://genome-www4.stanford.edu/cgi-bin/SMD/source/sourceResult?choice=Gene&option=Name&criteria=C1QA) | 1.59 | 0.0020 |
| A_24_P79300 | [CLDN5](http://genome-www4.stanford.edu/cgi-bin/SMD/source/sourceResult?choice=Gene&option=Name&criteria=CLDN5) | 1.70 | 0.0020 |
| A_23_P35883 | [FOXRED1](http://genome-www4.stanford.edu/cgi-bin/SMD/source/sourceResult?choice=Gene&option=Name&criteria=FOXRED1) | 1.52 | 0.0020 |
| A_23_P151297 | [TENC1](http://genome-www4.stanford.edu/cgi-bin/SMD/source/sourceResult?choice=Gene&option=Name&criteria=TENC1) | 1.52 | 0.0020 |
| A_24_P318990 | [BC012876](http://genome-www4.stanford.edu/cgi-bin/SMD/source/sourceResult?choice=Gene&option=Name&criteria=BC012876) | 1.80 | 0.0020 |
| A_23_P111888 | [CTHRC1](http://genome-www4.stanford.edu/cgi-bin/SMD/source/sourceResult?choice=Gene&option=Name&criteria=CTHRC1) | 1.57 | 0.0020 |
| A_24_P385326 | [ENST00000292357](http://genome-www4.stanford.edu/cgi-bin/SMD/source/sourceResult?choice=Gene&option=Name&criteria=ENST00000292357) | 1.65 | 0.0020 |
| A_23_P361654 | [IGKC](http://genome-www4.stanford.edu/cgi-bin/SMD/source/sourceResult?choice=Gene&option=Name&criteria=IGKC) | 1.99 | 0.0020 |
| A_24_P935491 | [COL3A1](http://genome-www4.stanford.edu/cgi-bin/SMD/source/sourceResult?choice=Gene&option=Name&criteria=COL3A1) | 1.69 | 0.0020 |
| A_32_P157927 | [ENST00000283657](http://genome-www4.stanford.edu/cgi-bin/SMD/source/sourceResult?choice=Gene&option=Name&criteria=ENST00000283657) | 1.84 | 0.0020 |
| A_32_P65022 | [ENST00000327926](http://genome-www4.stanford.edu/cgi-bin/SMD/source/sourceResult?choice=Gene&option=Name&criteria=ENST00000327926) | 1.92 | 0.0020 |
| A_24_P319635 | [MCL1](http://genome-www4.stanford.edu/cgi-bin/SMD/source/sourceResult?choice=Gene&option=Name&criteria=MCL1) | 1.53 | 0.0020 |
| A_24_P169713 | [THC2428956](http://genome-www4.stanford.edu/cgi-bin/SMD/source/sourceResult?choice=Gene&option=Name&criteria=THC2428956) | 1.80 | 0.0020 |
| A_24_P376391 | [PLXND1](http://genome-www4.stanford.edu/cgi-bin/SMD/source/sourceResult?choice=Gene&option=Name&criteria=PLXND1) | 1.51 | 0.0037 |
| A_24_P693986 | [LOC388610](http://genome-www4.stanford.edu/cgi-bin/SMD/source/sourceResult?choice=Gene&option=Name&criteria=LOC388610) | 1.67 | 0.0037 |
| A_23_P74001 | [S100A12](http://genome-www4.stanford.edu/cgi-bin/SMD/source/sourceResult?choice=Gene&option=Name&criteria=S100A12) | 1.80 | 0.0037 |
| A_23_P123596 | [GLDC](http://genome-www4.stanford.edu/cgi-bin/SMD/source/sourceResult?choice=Gene&option=Name&criteria=GLDC) | 1.59 | 0.0037 |
| A_24_P59220 | [ENST00000375923](http://genome-www4.stanford.edu/cgi-bin/SMD/source/sourceResult?choice=Gene&option=Name&criteria=ENST00000375923) | 1.54 | 0.0037 |
| A_24_P608268 | [U21252](http://genome-www4.stanford.edu/cgi-bin/SMD/source/sourceResult?choice=Gene&option=Name&criteria=U21252) | 2.09 | 0.0037 |
| A_23_P373017 | [CCL3](http://genome-www4.stanford.edu/cgi-bin/SMD/source/sourceResult?choice=Gene&option=Name&criteria=CCL3) | 1.62 | 0.0037 |
| A_24_P281374 | [A_24_P281374](http://genome-www4.stanford.edu/cgi-bin/SMD/source/sourceResult?choice=Gene&option=Name&criteria=A_24_P281374) | 1.55 | 0.0037 |
| A_23_P26854 | [KIAA0672](http://genome-www4.stanford.edu/cgi-bin/SMD/source/sourceResult?choice=Gene&option=Name&criteria=KIAA0672) | 1.52 | 0.0037 |
| A_23_P129903 | [TRIM16](http://genome-www4.stanford.edu/cgi-bin/SMD/source/sourceResult?choice=Gene&option=Name&criteria=TRIM16) | 1.52 | 0.0037 |
| A_23_P217326 | [FHL1](http://genome-www4.stanford.edu/cgi-bin/SMD/source/sourceResult?choice=Gene&option=Name&criteria=FHL1) | 1.56 | 0.0037 |
| A_23_P126186 | [DEGS1](http://genome-www4.stanford.edu/cgi-bin/SMD/source/sourceResult?choice=Gene&option=Name&criteria=DEGS1) | 1.53 | 0.0037 |
| A_24_P152325 | [ENST00000306804](http://genome-www4.stanford.edu/cgi-bin/SMD/source/sourceResult?choice=Gene&option=Name&criteria=ENST00000306804) | 1.59 | 0.0037 |
| A_23_P121011 | [AXUD1](http://genome-www4.stanford.edu/cgi-bin/SMD/source/sourceResult?choice=Gene&option=Name&criteria=AXUD1) | 1.63 | 0.0037 |
| A_23_P142533 | [COL3A1](http://genome-www4.stanford.edu/cgi-bin/SMD/source/sourceResult?choice=Gene&option=Name&criteria=COL3A1) | 1.57 | 0.0037 |
| A_23_P255345 | [VNN1](http://genome-www4.stanford.edu/cgi-bin/SMD/source/sourceResult?choice=Gene&option=Name&criteria=VNN1) | 2.31 | 0.0037 |
| A_23_P51518 | [RGS5](http://genome-www4.stanford.edu/cgi-bin/SMD/source/sourceResult?choice=Gene&option=Name&criteria=RGS5) | 1.62 | 0.0037 |
| A_32_P159192 | [ENST00000295339](http://genome-www4.stanford.edu/cgi-bin/SMD/source/sourceResult?choice=Gene&option=Name&criteria=ENST00000295339) | 1.81 | 0.0037 |
| A_23_P214080 | [EGR1](http://genome-www4.stanford.edu/cgi-bin/SMD/source/sourceResult?choice=Gene&option=Name&criteria=EGR1) | 2.04 | 0.0037 |
| A_23_P435390 | [A_23_P435390](http://genome-www4.stanford.edu/cgi-bin/SMD/source/sourceResult?choice=Gene&option=Name&criteria=A_23_P435390) | 2.05 | 0.0037 |
| A_23_P157793 | [CA9](http://genome-www4.stanford.edu/cgi-bin/SMD/source/sourceResult?choice=Gene&option=Name&criteria=CA9) | 1.55 | 0.0037 |
| A_24_P361816 | [IGLV6-57](http://genome-www4.stanford.edu/cgi-bin/SMD/source/sourceResult?choice=Gene&option=Name&criteria=IGLV6-57) | 1.65 | 0.0037 |
| A_23_P84791 | [A_23_P84791](http://genome-www4.stanford.edu/cgi-bin/SMD/source/sourceResult?choice=Gene&option=Name&criteria=A_23_P84791) | 1.71 | 0.0037 |
| A_23_P154358 | [PROM2](http://genome-www4.stanford.edu/cgi-bin/SMD/source/sourceResult?choice=Gene&option=Name&criteria=PROM2) | 1.57 | 0.0037 |
| A_23_P76450 | [PHLDA1](http://genome-www4.stanford.edu/cgi-bin/SMD/source/sourceResult?choice=Gene&option=Name&criteria=PHLDA1) | 1.84 | 0.0037 |
| A_24_P677559 | [BC032451](http://genome-www4.stanford.edu/cgi-bin/SMD/source/sourceResult?choice=Gene&option=Name&criteria=BC032451) | 1.70 | 0.0037 |
| A_23_P1014 | [ENST00000367003](http://genome-www4.stanford.edu/cgi-bin/SMD/source/sourceResult?choice=Gene&option=Name&criteria=ENST00000367003) | 1.57 | 0.0037 |
| A_23_P152620 | [TNFSF13](http://genome-www4.stanford.edu/cgi-bin/SMD/source/sourceResult?choice=Gene&option=Name&criteria=TNFSF13) | 1.58 | 0.0037 |
| A_23_P2789 | [OLFM4](http://genome-www4.stanford.edu/cgi-bin/SMD/source/sourceResult?choice=Gene&option=Name&criteria=OLFM4) | 1.83 | 0.0037 |
| A_24_P924932 | [C1orf178](http://genome-www4.stanford.edu/cgi-bin/SMD/source/sourceResult?choice=Gene&option=Name&criteria=C1orf178) | 1.61 | 0.0037 |
| A_23_P160920 | [PDZK1IP1](http://genome-www4.stanford.edu/cgi-bin/SMD/source/sourceResult?choice=Gene&option=Name&criteria=PDZK1IP1) | 1.58 | 0.0037 |
| A_24_P626951 | [ENST00000295410](http://genome-www4.stanford.edu/cgi-bin/SMD/source/sourceResult?choice=Gene&option=Name&criteria=ENST00000295410) | 1.86 | 0.0050 |
| A_24_P538459 | [AF076205](http://genome-www4.stanford.edu/cgi-bin/SMD/source/sourceResult?choice=Gene&option=Name&criteria=AF076205) | 1.78 | 0.0050 |
| A_23_P91390 | [THBD](http://genome-www4.stanford.edu/cgi-bin/SMD/source/sourceResult?choice=Gene&option=Name&criteria=THBD) | 1.56 | 0.0050 |
| A_23_P203882 | [MMP19](http://genome-www4.stanford.edu/cgi-bin/SMD/source/sourceResult?choice=Gene&option=Name&criteria=MMP19) | 1.78 | 0.0050 |
| A_24_P494425 | [ENST00000377226](http://genome-www4.stanford.edu/cgi-bin/SMD/source/sourceResult?choice=Gene&option=Name&criteria=ENST00000377226) | 2.00 | 0.0050 |
| A_23_P39590 | [XDH](http://genome-www4.stanford.edu/cgi-bin/SMD/source/sourceResult?choice=Gene&option=Name&criteria=XDH) | 1.68 | 0.0050 |
| A_23_P17837 | [APOL1](http://genome-www4.stanford.edu/cgi-bin/SMD/source/sourceResult?choice=Gene&option=Name&criteria=APOL1) | 1.64 | 0.0050 |
| A_23_P137697 | [SELP](http://genome-www4.stanford.edu/cgi-bin/SMD/source/sourceResult?choice=Gene&option=Name&criteria=SELP) | 1.59 | 0.0050 |
| A_23_P125977 | [C1QC](http://genome-www4.stanford.edu/cgi-bin/SMD/source/sourceResult?choice=Gene&option=Name&criteria=C1QC) | 1.70 | 0.0050 |
| A_24_P272146 | [IGKC](http://genome-www4.stanford.edu/cgi-bin/SMD/source/sourceResult?choice=Gene&option=Name&criteria=IGKC) | 1.85 | 0.0050 |
| A_23_P21800 | [BC032451](http://genome-www4.stanford.edu/cgi-bin/SMD/source/sourceResult?choice=Gene&option=Name&criteria=BC032451) | 1.76 | 0.0050 |
| A_23_P149562 | [ARHGAP29](http://genome-www4.stanford.edu/cgi-bin/SMD/source/sourceResult?choice=Gene&option=Name&criteria=ARHGAP29) | 1.61 | 0.0050 |
| A_24_P510357 | [IGLV2-14](http://genome-www4.stanford.edu/cgi-bin/SMD/source/sourceResult?choice=Gene&option=Name&criteria=IGLV2-14) | 1.73 | 0.0050 |
| A_24_P484904 | [AJ399872](http://genome-www4.stanford.edu/cgi-bin/SMD/source/sourceResult?choice=Gene&option=Name&criteria=AJ399872) | 1.81 | 0.0050 |
| A_24_P183150 | [CXCL3](http://genome-www4.stanford.edu/cgi-bin/SMD/source/sourceResult?choice=Gene&option=Name&criteria=CXCL3) | 1.90 | 0.0050 |
| A_23_P105562 | [VWF](http://genome-www4.stanford.edu/cgi-bin/SMD/source/sourceResult?choice=Gene&option=Name&criteria=VWF) | 1.55 | 0.0050 |
| A_24_P299685 | [PDPN](http://genome-www4.stanford.edu/cgi-bin/SMD/source/sourceResult?choice=Gene&option=Name&criteria=PDPN) | 1.52 | 0.0050 |
| A_23_P32444 | [MXRA8](http://genome-www4.stanford.edu/cgi-bin/SMD/source/sourceResult?choice=Gene&option=Name&criteria=MXRA8) | 1.51 | 0.0050 |
| A_23_P159163 | [A_23_P159163](http://genome-www4.stanford.edu/cgi-bin/SMD/source/sourceResult?choice=Gene&option=Name&criteria=A_23_P159163) | 1.93 | 0.0050 |
| A_24_P328320 | [GORASP2](http://genome-www4.stanford.edu/cgi-bin/SMD/source/sourceResult?choice=Gene&option=Name&criteria=GORASP2) | 1.75 | 0.0050 |
| A_24_P212997 | [A_24_P212997](http://genome-www4.stanford.edu/cgi-bin/SMD/source/sourceResult?choice=Gene&option=Name&criteria=A_24_P212997) | 1.53 | 0.0050 |
| A_32_P231617 | [TM4SF1](http://genome-www4.stanford.edu/cgi-bin/SMD/source/sourceResult?choice=Gene&option=Name&criteria=TM4SF1) | 1.57 | 0.0050 |
| A_23_P103110 | [MAFF](http://genome-www4.stanford.edu/cgi-bin/SMD/source/sourceResult?choice=Gene&option=Name&criteria=MAFF) | 1.53 | 0.0050 |
| A_24_P315854 | [ENST00000216649](http://genome-www4.stanford.edu/cgi-bin/SMD/source/sourceResult?choice=Gene&option=Name&criteria=ENST00000216649) | 1.87 | 0.0050 |
| A_23_P306203 | [SAA2](http://genome-www4.stanford.edu/cgi-bin/SMD/source/sourceResult?choice=Gene&option=Name&criteria=SAA2) | 1.68 | 0.0050 |
| A_32_P148118 | [ENST00000331696](http://genome-www4.stanford.edu/cgi-bin/SMD/source/sourceResult?choice=Gene&option=Name&criteria=ENST00000331696) | 1.76 | 0.0050 |
| A_23_P136026 | [IGHA1](http://genome-www4.stanford.edu/cgi-bin/SMD/source/sourceResult?choice=Gene&option=Name&criteria=IGHA1) | 1.85 | 0.0050 |
| A_23_P3038 | [GPX2](http://genome-www4.stanford.edu/cgi-bin/SMD/source/sourceResult?choice=Gene&option=Name&criteria=GPX2) | 1.54 | 0.0062 |
| A_24_P788878 | [THC2371963](http://genome-www4.stanford.edu/cgi-bin/SMD/source/sourceResult?choice=Gene&option=Name&criteria=THC2371963) | 1.60 | 0.0062 |
| A_23_P19936 | [KDELR2](http://genome-www4.stanford.edu/cgi-bin/SMD/source/sourceResult?choice=Gene&option=Name&criteria=KDELR2) | 1.52 | 0.0062 |
| A_23_P12746 | [MRC1L1](http://genome-www4.stanford.edu/cgi-bin/SMD/source/sourceResult?choice=Gene&option=Name&criteria=MRC1L1) | 1.57 | 0.0062 |
| A_24_P199774 | [TINAGL1](http://genome-www4.stanford.edu/cgi-bin/SMD/source/sourceResult?choice=Gene&option=Name&criteria=TINAGL1) | 1.70 | 0.0062 |
| A_24_P93523 | [LOC647460](http://genome-www4.stanford.edu/cgi-bin/SMD/source/sourceResult?choice=Gene&option=Name&criteria=LOC647460) | 1.58 | 0.0062 |
| A_24_P714707 | [A_24_P714707](http://genome-www4.stanford.edu/cgi-bin/SMD/source/sourceResult?choice=Gene&option=Name&criteria=A_24_P714707) | 1.51 | 0.0062 |
| A_23_P211233 | [COL6A2](http://genome-www4.stanford.edu/cgi-bin/SMD/source/sourceResult?choice=Gene&option=Name&criteria=COL6A2) | 1.84 | 0.0062 |
| A_32_P107372 | [GBP1](http://genome-www4.stanford.edu/cgi-bin/SMD/source/sourceResult?choice=Gene&option=Name&criteria=GBP1) | 1.50 | 0.0062 |
| A_24_P6850 | [A_24_P6850](http://genome-www4.stanford.edu/cgi-bin/SMD/source/sourceResult?choice=Gene&option=Name&criteria=A_24_P6850) | 1.55 | 0.0062 |
| A_23_P70571 | [SLC39A7](http://genome-www4.stanford.edu/cgi-bin/SMD/source/sourceResult?choice=Gene&option=Name&criteria=SLC39A7) | 1.72 | 0.0062 |
| A_23_P1691 | [MMP1](http://genome-www4.stanford.edu/cgi-bin/SMD/source/sourceResult?choice=Gene&option=Name&criteria=MMP1) | 1.96 | 0.0062 |
| A_24_P409420 | [A_24_P409420](http://genome-www4.stanford.edu/cgi-bin/SMD/source/sourceResult?choice=Gene&option=Name&criteria=A_24_P409420) | 1.56 | 0.0062 |
| A_23_P213562 | [F2R](http://genome-www4.stanford.edu/cgi-bin/SMD/source/sourceResult?choice=Gene&option=Name&criteria=F2R) | 1.61 | 0.0062 |
| A_24_P357847 | [BC030813](http://genome-www4.stanford.edu/cgi-bin/SMD/source/sourceResult?choice=Gene&option=Name&criteria=BC030813) | 1.79 | 0.0062 |
| A_24_P358321 | [ENST00000377233](http://genome-www4.stanford.edu/cgi-bin/SMD/source/sourceResult?choice=Gene&option=Name&criteria=ENST00000377233) | 1.75 | 0.0062 |
| A_24_P349590 | [ENST00000318251](http://genome-www4.stanford.edu/cgi-bin/SMD/source/sourceResult?choice=Gene&option=Name&criteria=ENST00000318251) | 1.59 | 0.0062 |
| A_23_P156185 | [APXL2](http://genome-www4.stanford.edu/cgi-bin/SMD/source/sourceResult?choice=Gene&option=Name&criteria=APXL2) | 1.59 | 0.0062 |
| A_23_P41854 | [CARD6](http://genome-www4.stanford.edu/cgi-bin/SMD/source/sourceResult?choice=Gene&option=Name&criteria=CARD6) | 1.55 | 0.0062 |
| A_24_P518369 | [A_24_P518369](http://genome-www4.stanford.edu/cgi-bin/SMD/source/sourceResult?choice=Gene&option=Name&criteria=A_24_P518369) | 1.72 | 0.0062 |
| A_24_P12438 | [NCOA7](http://genome-www4.stanford.edu/cgi-bin/SMD/source/sourceResult?choice=Gene&option=Name&criteria=NCOA7) | 1.57 | 0.0062 |
| A_24_P66578 | [ENST00000310579](http://genome-www4.stanford.edu/cgi-bin/SMD/source/sourceResult?choice=Gene&option=Name&criteria=ENST00000310579) | 1.77 | 0.0062 |
| A_23_P425681 | [CCK](http://genome-www4.stanford.edu/cgi-bin/SMD/source/sourceResult?choice=Gene&option=Name&criteria=CCK) | 1.89 | 0.0062 |
| A_23_P217428 | [ARHGAP6](http://genome-www4.stanford.edu/cgi-bin/SMD/source/sourceResult?choice=Gene&option=Name&criteria=ARHGAP6) | 1.50 | 0.0062 |
| A_24_P332595 | [A_24_P332595](http://genome-www4.stanford.edu/cgi-bin/SMD/source/sourceResult?choice=Gene&option=Name&criteria=A_24_P332595) | 1.53 | 0.0087 |
| A_32_P140501 | [A_32_P140501](http://genome-www4.stanford.edu/cgi-bin/SMD/source/sourceResult?choice=Gene&option=Name&criteria=A_32_P140501) | 1.54 | 0.0087 |
| A_23_P56630 | [STAT1](http://genome-www4.stanford.edu/cgi-bin/SMD/source/sourceResult?choice=Gene&option=Name&criteria=STAT1) | 1.54 | 0.0087 |
| A_24_P490109 | [A_24_P490109](http://genome-www4.stanford.edu/cgi-bin/SMD/source/sourceResult?choice=Gene&option=Name&criteria=A_24_P490109) | 1.72 | 0.0087 |
| A_24_P465799 | [AF063695](http://genome-www4.stanford.edu/cgi-bin/SMD/source/sourceResult?choice=Gene&option=Name&criteria=AF063695) | 1.76 | 0.0087 |
| A_32_P164246 | [FOXQ1](http://genome-www4.stanford.edu/cgi-bin/SMD/source/sourceResult?choice=Gene&option=Name&criteria=FOXQ1) | 1.65 | 0.0087 |
| A_23_P124300 | [BCMO1](http://genome-www4.stanford.edu/cgi-bin/SMD/source/sourceResult?choice=Gene&option=Name&criteria=BCMO1) | 1.58 | 0.0087 |
| A_23_P36445 | [TMED2](http://genome-www4.stanford.edu/cgi-bin/SMD/source/sourceResult?choice=Gene&option=Name&criteria=TMED2) | 1.65 | 0.0087 |
| A_24_P387839 | [DEGS1](http://genome-www4.stanford.edu/cgi-bin/SMD/source/sourceResult?choice=Gene&option=Name&criteria=DEGS1) | 1.63 | 0.0087 |
| A_23_P116235 | [MDK](http://genome-www4.stanford.edu/cgi-bin/SMD/source/sourceResult?choice=Gene&option=Name&criteria=MDK) | 1.56 | 0.0087 |
| A_24_P383660 | [A_24_P383660](http://genome-www4.stanford.edu/cgi-bin/SMD/source/sourceResult?choice=Gene&option=Name&criteria=A_24_P383660) | 1.52 | 0.0087 |
| A_23_P6909 | [CCRL1](http://genome-www4.stanford.edu/cgi-bin/SMD/source/sourceResult?choice=Gene&option=Name&criteria=CCRL1) | 1.52 | 0.0087 |
| A_23_P208126 | [SERPINB5](http://genome-www4.stanford.edu/cgi-bin/SMD/source/sourceResult?choice=Gene&option=Name&criteria=SERPINB5) | 1.58 | 0.0087 |
| A_23_P71379 | [PSCA](http://genome-www4.stanford.edu/cgi-bin/SMD/source/sourceResult?choice=Gene&option=Name&criteria=PSCA) | 2.05 | 0.0087 |
| A_24_P813550 | [ENST00000216649](http://genome-www4.stanford.edu/cgi-bin/SMD/source/sourceResult?choice=Gene&option=Name&criteria=ENST00000216649) | 1.85 | 0.0087 |
| A_23_P119562 | [CFD](http://genome-www4.stanford.edu/cgi-bin/SMD/source/sourceResult?choice=Gene&option=Name&criteria=CFD) | 1.57 | 0.0087 |
| A_23_P64938 | [MBOAT5](http://genome-www4.stanford.edu/cgi-bin/SMD/source/sourceResult?choice=Gene&option=Name&criteria=MBOAT5) | 1.75 | 0.0087 |
| A_23_P128230 | [NR4A1](http://genome-www4.stanford.edu/cgi-bin/SMD/source/sourceResult?choice=Gene&option=Name&criteria=NR4A1) | 1.78 | 0.0115 |
| A_23_P123265 | [SUMF2](http://genome-www4.stanford.edu/cgi-bin/SMD/source/sourceResult?choice=Gene&option=Name&criteria=SUMF2) | 1.51 | 0.0115 |
| A_32_P39440 | [BC030813](http://genome-www4.stanford.edu/cgi-bin/SMD/source/sourceResult?choice=Gene&option=Name&criteria=BC030813) | 1.77 | 0.0115 |
| A_23_P6362 | [DERL3](http://genome-www4.stanford.edu/cgi-bin/SMD/source/sourceResult?choice=Gene&option=Name&criteria=DERL3) | 1.71 | 0.0115 |
| A_24_P161809 | [ENST00000344214](http://genome-www4.stanford.edu/cgi-bin/SMD/source/sourceResult?choice=Gene&option=Name&criteria=ENST00000344214) | 1.51 | 0.0115 |
| A_23_P390206 | [ENST00000358917](http://genome-www4.stanford.edu/cgi-bin/SMD/source/sourceResult?choice=Gene&option=Name&criteria=ENST00000358917) | 1.64 | 0.0115 |
| A_23_P46141 | [CTSS](http://genome-www4.stanford.edu/cgi-bin/SMD/source/sourceResult?choice=Gene&option=Name&criteria=CTSS) | 1.50 | 0.0115 |
| A_24_P639701 | [AY062331](http://genome-www4.stanford.edu/cgi-bin/SMD/source/sourceResult?choice=Gene&option=Name&criteria=AY062331) | 1.61 | 0.0115 |
| A_24_P75708 | [A_24_P75708](http://genome-www4.stanford.edu/cgi-bin/SMD/source/sourceResult?choice=Gene&option=Name&criteria=A_24_P75708) | 1.58 | 0.0115 |
| A_23_P161698 | [MMP3](http://genome-www4.stanford.edu/cgi-bin/SMD/source/sourceResult?choice=Gene&option=Name&criteria=MMP3) | 1.74 | 0.0115 |
| A_32_P191895 | [BC045716](http://genome-www4.stanford.edu/cgi-bin/SMD/source/sourceResult?choice=Gene&option=Name&criteria=BC045716) | 1.51 | 0.0115 |
| A_23_P122924 | [INHBA](http://genome-www4.stanford.edu/cgi-bin/SMD/source/sourceResult?choice=Gene&option=Name&criteria=INHBA) | 1.52 | 0.0115 |
| A_32_P148710 | [CFL1](http://genome-www4.stanford.edu/cgi-bin/SMD/source/sourceResult?choice=Gene&option=Name&criteria=CFL1) | 1.58 | 0.0115 |
| A_23_P126248 | [RNF186](http://genome-www4.stanford.edu/cgi-bin/SMD/source/sourceResult?choice=Gene&option=Name&criteria=RNF186) | 1.67 | 0.0115 |
| A_23_P85201 | [PLP1](http://genome-www4.stanford.edu/cgi-bin/SMD/source/sourceResult?choice=Gene&option=Name&criteria=PLP1) | 1.53 | 0.0115 |
| A_24_P179107 | [AF267875](http://genome-www4.stanford.edu/cgi-bin/SMD/source/sourceResult?choice=Gene&option=Name&criteria=AF267875) | 1.65 | 0.0115 |
| A_23_P123503 | [TRIB1](http://genome-www4.stanford.edu/cgi-bin/SMD/source/sourceResult?choice=Gene&option=Name&criteria=TRIB1) | 1.51 | 0.0115 |
| A_24_P68649 | [RNPEP](http://genome-www4.stanford.edu/cgi-bin/SMD/source/sourceResult?choice=Gene&option=Name&criteria=RNPEP) | 1.66 | 0.0115 |
| A_24_P578445 | [A_24_P578445](http://genome-www4.stanford.edu/cgi-bin/SMD/source/sourceResult?choice=Gene&option=Name&criteria=A_24_P578445) | 1.53 | 0.0115 |
| A_23_P360605 | [KIAA0802](http://genome-www4.stanford.edu/cgi-bin/SMD/source/sourceResult?choice=Gene&option=Name&criteria=KIAA0802) | 1.52 | 0.0139 |
| A_23_P158868 | [A_23_P158868](http://genome-www4.stanford.edu/cgi-bin/SMD/source/sourceResult?choice=Gene&option=Name&criteria=A_23_P158868) | 1.82 | 0.0139 |
| A_24_P78531 | [CLEC4E](http://genome-www4.stanford.edu/cgi-bin/SMD/source/sourceResult?choice=Gene&option=Name&criteria=CLEC4E) | 1.59 | 0.0139 |
| A_24_P118341 | [BF869497](http://genome-www4.stanford.edu/cgi-bin/SMD/source/sourceResult?choice=Gene&option=Name&criteria=BF869497) | 1.53 | 0.0139 |
| A_23_P88347 | FERMT2 | 1.53 | 0.0139 |
| A_23_P155624 | [AP2M1](http://genome-www4.stanford.edu/cgi-bin/SMD/source/sourceResult?choice=Gene&option=Name&criteria=AP2M1) | 1.53 | 0.0139 |
| A_24_P204374 | [ENST00000328419](http://genome-www4.stanford.edu/cgi-bin/SMD/source/sourceResult?choice=Gene&option=Name&criteria=ENST00000328419) | 1.66 | 0.0139 |
| A_24_P605563 | [AY172962](http://genome-www4.stanford.edu/cgi-bin/SMD/source/sourceResult?choice=Gene&option=Name&criteria=AY172962) | 1.82 | 0.0139 |
| A_23_P369899 | [TMEM158](http://genome-www4.stanford.edu/cgi-bin/SMD/source/sourceResult?choice=Gene&option=Name&criteria=TMEM158) | 1.66 | 0.0139 |
| A_23_P214079 | [SPINK1](http://genome-www4.stanford.edu/cgi-bin/SMD/source/sourceResult?choice=Gene&option=Name&criteria=SPINK1) | 1.53 | 0.0139 |
| A_23_P112026 | [IDO1](http://genome-www4.stanford.edu/cgi-bin/SMD/source/sourceResult?choice=Gene&option=Name&criteria=INDO) | 1.61 | 0.0139 |
| A_24_P335025 | [ENTPD1](http://genome-www4.stanford.edu/cgi-bin/SMD/source/sourceResult?choice=Gene&option=Name&criteria=ENTPD1) | 1.54 | 0.0139 |
| A_23_P70095 | [CD74](http://genome-www4.stanford.edu/cgi-bin/SMD/source/sourceResult?choice=Gene&option=Name&criteria=CD74) | 1.56 | 0.0139 |
| A_23_P21260 | [ENST00000359488](http://genome-www4.stanford.edu/cgi-bin/SMD/source/sourceResult?choice=Gene&option=Name&criteria=ENST00000359488) | 1.67 | 0.0139 |
| A_23_P93349 | [SLC44A4](http://genome-www4.stanford.edu/cgi-bin/SMD/source/sourceResult?choice=Gene&option=Name&criteria=SLC44A4) | 1.67 | 0.0139 |
| A_24_P337700 | [VNN1](http://genome-www4.stanford.edu/cgi-bin/SMD/source/sourceResult?choice=Gene&option=Name&criteria=VNN1) | 2.11 | 0.0139 |
| A_32_P225816 | [PRDM16](http://genome-www4.stanford.edu/cgi-bin/SMD/source/sourceResult?choice=Gene&option=Name&criteria=PRDM16) | 1.51 | 0.0139 |
| A_23_P147641 | [TCEA2](http://genome-www4.stanford.edu/cgi-bin/SMD/source/sourceResult?choice=Gene&option=Name&criteria=TCEA2) | 1.56 | 0.0139 |
| A_23_P96568 | [FLNA](http://genome-www4.stanford.edu/cgi-bin/SMD/source/sourceResult?choice=Gene&option=Name&criteria=FLNA) | 1.76 | 0.0139 |
| A_23_P207456 | [CCL8](http://genome-www4.stanford.edu/cgi-bin/SMD/source/sourceResult?choice=Gene&option=Name&criteria=CCL8) | 1.73 | 0.0139 |
| A_24_P140608 | [HBEGF](http://genome-www4.stanford.edu/cgi-bin/SMD/source/sourceResult?choice=Gene&option=Name&criteria=HBEGF) | 1.67 | 0.0139 |
| A_23_P10127 | [SFRP1](http://genome-www4.stanford.edu/cgi-bin/SMD/source/sourceResult?choice=Gene&option=Name&criteria=SFRP1) | 1.56 | 0.0139 |
| A_23_P157875 | [FCN1](http://genome-www4.stanford.edu/cgi-bin/SMD/source/sourceResult?choice=Gene&option=Name&criteria=FCN1) | 1.51 | 0.0139 |
| A_23_P84596 | [PACAP](http://genome-www4.stanford.edu/cgi-bin/SMD/source/sourceResult?choice=Gene&option=Name&criteria=PACAP) | 1.67 | 0.0139 |
| A_23_P35820 | [CFL1](http://genome-www4.stanford.edu/cgi-bin/SMD/source/sourceResult?choice=Gene&option=Name&criteria=CFL1) | 1.51 | 0.0139 |
| A_24_P13572 | [DLST](http://genome-www4.stanford.edu/cgi-bin/SMD/source/sourceResult?choice=Gene&option=Name&criteria=DLST) | 1.54 | 0.0139 |
| A_24_P16004 | [NP102468](http://genome-www4.stanford.edu/cgi-bin/SMD/source/sourceResult?choice=Gene&option=Name&criteria=NP102468) | 1.80 | 0.0139 |
| A_23_P500010 | [KLK12](http://genome-www4.stanford.edu/cgi-bin/SMD/source/sourceResult?choice=Gene&option=Name&criteria=KLK12) | 1.60 | 0.0139 |
| A_24_P307375 | [ENST00000312946](http://genome-www4.stanford.edu/cgi-bin/SMD/source/sourceResult?choice=Gene&option=Name&criteria=ENST00000312946) | 1.69 | 0.0139 |
| A_24_P253003 | [WNT11](http://genome-www4.stanford.edu/cgi-bin/SMD/source/sourceResult?choice=Gene&option=Name&criteria=WNT11) | 1.69 | 0.0164 |
| A_32_P163858 | [SCD](http://genome-www4.stanford.edu/cgi-bin/SMD/source/sourceResult?choice=Gene&option=Name&criteria=SCD) | 1.53 | 0.0164 |
| A_23_P114008 | [TM4SF20](http://genome-www4.stanford.edu/cgi-bin/SMD/source/sourceResult?choice=Gene&option=Name&criteria=TM4SF20) | 2.21 | 0.0164 |
| A_23_P62857 | [A_23_P62857](http://genome-www4.stanford.edu/cgi-bin/SMD/source/sourceResult?choice=Gene&option=Name&criteria=A_23_P62857) | 1.50 | 0.0164 |
| A_23_P111766 | [A_23_P111766](http://genome-www4.stanford.edu/cgi-bin/SMD/source/sourceResult?choice=Gene&option=Name&criteria=A_23_P111766) | 1.59 | 0.0164 |
| A_24_P382661 | [ETS2](http://genome-www4.stanford.edu/cgi-bin/SMD/source/sourceResult?choice=Gene&option=Name&criteria=ETS2) | 1.50 | 0.0164 |
| A_23_P170830 | [CD709370](http://genome-www4.stanford.edu/cgi-bin/SMD/source/sourceResult?choice=Gene&option=Name&criteria=CD709370) | 1.71 | 0.0164 |
| A_24_P399622 | [COPE](http://genome-www4.stanford.edu/cgi-bin/SMD/source/sourceResult?choice=Gene&option=Name&criteria=COPE) | 1.53 | 0.0164 |
| A_23_P74290 | [GBP5](http://genome-www4.stanford.edu/cgi-bin/SMD/source/sourceResult?choice=Gene&option=Name&criteria=GBP5) | 1.54 | 0.0164 |
| A_23_P393620 | [TFPI2](http://genome-www4.stanford.edu/cgi-bin/SMD/source/sourceResult?choice=Gene&option=Name&criteria=TFPI2) | 1.81 | 0.0164 |
| A_23_P36448 | [TMED2](http://genome-www4.stanford.edu/cgi-bin/SMD/source/sourceResult?choice=Gene&option=Name&criteria=TMED2) | 1.53 | 0.0164 |
| A_24_P283221 | [BIRC1](http://genome-www4.stanford.edu/cgi-bin/SMD/source/sourceResult?choice=Gene&option=Name&criteria=BIRC1) | 1.55 | 0.0164 |
| A_23_P60339 | [C9orf64](http://genome-www4.stanford.edu/cgi-bin/SMD/source/sourceResult?choice=Gene&option=Name&criteria=C9orf64) | 1.51 | 0.0164 |
| A_23_P129209 | [IDH2](http://genome-www4.stanford.edu/cgi-bin/SMD/source/sourceResult?choice=Gene&option=Name&criteria=IDH2) | 1.56 | 0.0164 |
| A_24_P941217 | [SGPP2](http://genome-www4.stanford.edu/cgi-bin/SMD/source/sourceResult?choice=Gene&option=Name&criteria=SGPP2) | 1.64 | 0.0164 |
| A_24_P83102 | [IGLL1](http://genome-www4.stanford.edu/cgi-bin/SMD/source/sourceResult?choice=Gene&option=Name&criteria=IGLL1) | 1.61 | 0.0164 |
| A_23_P212089 | [NFKBIZ](http://genome-www4.stanford.edu/cgi-bin/SMD/source/sourceResult?choice=Gene&option=Name&criteria=NFKBIZ) | 1.55 | 0.0164 |
| A_24_P413286 | [A_24_P413286](http://genome-www4.stanford.edu/cgi-bin/SMD/source/sourceResult?choice=Gene&option=Name&criteria=A_24_P413286) | 1.68 | 0.0164 |
| A_32_P72553 | [LOC162073](http://genome-www4.stanford.edu/cgi-bin/SMD/source/sourceResult?choice=Gene&option=Name&criteria=LOC162073) | 1.52 | 0.0164 |
| A_23_P3532 | [LITAF](http://genome-www4.stanford.edu/cgi-bin/SMD/source/sourceResult?choice=Gene&option=Name&criteria=LITAF) | 1.55 | 0.0196 |
| A_23_P209995 | [IL1RN](http://genome-www4.stanford.edu/cgi-bin/SMD/source/sourceResult?choice=Gene&option=Name&criteria=IL1RN) | 1.67 | 0.0196 |
| A_23_P90710 | [DES](http://genome-www4.stanford.edu/cgi-bin/SMD/source/sourceResult?choice=Gene&option=Name&criteria=DES) | 1.65 | 0.0196 |
| A_24_P375609 | [EIF5A](http://genome-www4.stanford.edu/cgi-bin/SMD/source/sourceResult?choice=Gene&option=Name&criteria=EIF5A) | 1.59 | 0.0196 |
| A_23_P404698 | [FLJ35880](http://genome-www4.stanford.edu/cgi-bin/SMD/source/sourceResult?choice=Gene&option=Name&criteria=FLJ35880) | 1.59 | 0.0196 |
| A_23_P43988 | [DPYD](http://genome-www4.stanford.edu/cgi-bin/SMD/source/sourceResult?choice=Gene&option=Name&criteria=DPYD) | 1.55 | 0.0196 |
| A_23_P126613 | [AQP10](http://genome-www4.stanford.edu/cgi-bin/SMD/source/sourceResult?choice=Gene&option=Name&criteria=AQP10) | 1.63 | 0.0196 |
| A_24_P392774 | [HSD17B12](http://genome-www4.stanford.edu/cgi-bin/SMD/source/sourceResult?choice=Gene&option=Name&criteria=HSD17B12) | 1.57 | 0.0196 |
| A_24_P356592 | [PRKAR1A](http://genome-www4.stanford.edu/cgi-bin/SMD/source/sourceResult?choice=Gene&option=Name&criteria=PRKAR1A) | 1.57 | 0.0196 |
| A_24_P203886 | [A_24_P203886](http://genome-www4.stanford.edu/cgi-bin/SMD/source/sourceResult?choice=Gene&option=Name&criteria=A_24_P203886) | 1.51 | 0.0196 |
| A_24_P241996 | [AY043127](http://genome-www4.stanford.edu/cgi-bin/SMD/source/sourceResult?choice=Gene&option=Name&criteria=AY043127) | 1.54 | 0.0196 |
| A_23_P79217 | [LCT](http://genome-www4.stanford.edu/cgi-bin/SMD/source/sourceResult?choice=Gene&option=Name&criteria=LCT) | 2.56 | 0.0196 |
| A_23_P134347 | [CPVL](http://genome-www4.stanford.edu/cgi-bin/SMD/source/sourceResult?choice=Gene&option=Name&criteria=CPVL) | 1.52 | 0.0196 |
| A_32_P132194 | [BX110985](http://genome-www4.stanford.edu/cgi-bin/SMD/source/sourceResult?choice=Gene&option=Name&criteria=BX110985) | 1.60 | 0.0196 |
| A_23_P42257 | [IER3](http://genome-www4.stanford.edu/cgi-bin/SMD/source/sourceResult?choice=Gene&option=Name&criteria=IER3) | 1.79 | 0.0196 |
| A_32_P142440 | [PCSK9](http://genome-www4.stanford.edu/cgi-bin/SMD/source/sourceResult?choice=Gene&option=Name&criteria=PCSK9) | 1.59 | 0.0196 |
| A_23_P16673 | [CNN2](http://genome-www4.stanford.edu/cgi-bin/SMD/source/sourceResult?choice=Gene&option=Name&criteria=CNN2) | 1.51 | 0.0196 |
| A_24_P347854 | [SYNGR2](http://genome-www4.stanford.edu/cgi-bin/SMD/source/sourceResult?choice=Gene&option=Name&criteria=SYNGR2) | 1.62 | 0.0214 |
| A_23_P86470 | [CH25H](http://genome-www4.stanford.edu/cgi-bin/SMD/source/sourceResult?choice=Gene&option=Name&criteria=CH25H) | 1.57 | 0.0214 |
| A_23_P51538 | [GPA33](http://genome-www4.stanford.edu/cgi-bin/SMD/source/sourceResult?choice=Gene&option=Name&criteria=GPA33) | 1.52 | 0.0214 |
| A_24_P31235 | [EIF5A](http://genome-www4.stanford.edu/cgi-bin/SMD/source/sourceResult?choice=Gene&option=Name&criteria=EIF5A) | 1.57 | 0.0214 |
| A_32_P234459 | [HLA-H](http://genome-www4.stanford.edu/cgi-bin/SMD/source/sourceResult?choice=Gene&option=Name&criteria=HLA-H) | 1.70 | 0.0214 |
| A_32_P94444 | [PRSS2](http://genome-www4.stanford.edu/cgi-bin/SMD/source/sourceResult?choice=Gene&option=Name&criteria=PRSS2) | 1.60 | 0.0214 |
| A_24_P239076 | [CTA-246H3.1](http://genome-www4.stanford.edu/cgi-bin/SMD/source/sourceResult?choice=Gene&option=Name&criteria=CTA-246H3.1) | 1.56 | 0.0214 |
| A_23_P102864 | [PRSS7](http://genome-www4.stanford.edu/cgi-bin/SMD/source/sourceResult?choice=Gene&option=Name&criteria=PRSS7) | 1.94 | 0.0214 |
| A_24_P519504 | [X57802](http://genome-www4.stanford.edu/cgi-bin/SMD/source/sourceResult?choice=Gene&option=Name&criteria=X57802) | 1.68 | 0.0214 |
| A_24_P101771 | [A_24_P101771](http://genome-www4.stanford.edu/cgi-bin/SMD/source/sourceResult?choice=Gene&option=Name&criteria=A_24_P101771) | 1.75 | 0.0214 |
| A_23_P259071 | [AREG](http://genome-www4.stanford.edu/cgi-bin/SMD/source/sourceResult?choice=Gene&option=Name&criteria=AREG) | 1.89 | 0.0214 |
| A_23_P10640 | [ENPP7](http://genome-www4.stanford.edu/cgi-bin/SMD/source/sourceResult?choice=Gene&option=Name&criteria=ENPP7) | 1.52 | 0.0214 |
| A_32_P65628 | [REG3G](http://genome-www4.stanford.edu/cgi-bin/SMD/source/sourceResult?choice=Gene&option=Name&criteria=REG3G) | 1.78 | 0.0214 |
| A_23_P217088 | [AK1](http://genome-www4.stanford.edu/cgi-bin/SMD/source/sourceResult?choice=Gene&option=Name&criteria=AK1) | 1.62 | 0.0249 |
| A_23_P216429 | [ASPN](http://genome-www4.stanford.edu/cgi-bin/SMD/source/sourceResult?choice=Gene&option=Name&criteria=ASPN) | 1.70 | 0.0249 |
| A_23_P103496 | [GBP4](http://genome-www4.stanford.edu/cgi-bin/SMD/source/sourceResult?choice=Gene&option=Name&criteria=GBP4) | 1.57 | 0.0249 |
| A_32_P97169 | [ENST00000377047](http://genome-www4.stanford.edu/cgi-bin/SMD/source/sourceResult?choice=Gene&option=Name&criteria=ENST00000377047) | 1.50 | 0.0249 |
| A_24_P161933 | [CR608347](http://genome-www4.stanford.edu/cgi-bin/SMD/source/sourceResult?choice=Gene&option=Name&criteria=CR608347) | 1.62 | 0.0249 |
| A_23_P30848 | [HLA-E](http://genome-www4.stanford.edu/cgi-bin/SMD/source/sourceResult?choice=Gene&option=Name&criteria=HLA-E) | 1.66 | 0.0249 |
| A_23_P211468 | [AA837799](http://genome-www4.stanford.edu/cgi-bin/SMD/source/sourceResult?choice=Gene&option=Name&criteria=AA837799) | 1.61 | 0.0279 |
| A_24_P110012 | [FLJ45422](http://genome-www4.stanford.edu/cgi-bin/SMD/source/sourceResult?choice=Gene&option=Name&criteria=FLJ45422) | 1.66 | 0.0279 |
| A_23_P6596 | [HES1](http://genome-www4.stanford.edu/cgi-bin/SMD/source/sourceResult?choice=Gene&option=Name&criteria=HES1) | 1.55 | 0.0279 |
| A_32_P148122 | [IGKC](http://genome-www4.stanford.edu/cgi-bin/SMD/source/sourceResult?choice=Gene&option=Name&criteria=IGKC) | 1.51 | 0.0279 |
| A_24_P917492 | [TTLL3](http://genome-www4.stanford.edu/cgi-bin/SMD/source/sourceResult?choice=Gene&option=Name&criteria=TTLL3) | 1.54 | 0.0279 |
| A_23_P119943 | [IGFBP2](http://genome-www4.stanford.edu/cgi-bin/SMD/source/sourceResult?choice=Gene&option=Name&criteria=IGFBP2) | 1.56 | 0.0279 |
| A_23_P28238 | [SNX17](http://genome-www4.stanford.edu/cgi-bin/SMD/source/sourceResult?choice=Gene&option=Name&criteria=SNX17) | 1.55 | 0.0306 |
| A_24_P333479 | [SLC39A14](http://genome-www4.stanford.edu/cgi-bin/SMD/source/sourceResult?choice=Gene&option=Name&criteria=SLC39A14) | 1.51 | 0.0306 |
| A_24_P263767 | [ENST00000376793](http://genome-www4.stanford.edu/cgi-bin/SMD/source/sourceResult?choice=Gene&option=Name&criteria=ENST00000376793) | 1.62 | 0.0306 |
| A_23_P18452 | [CXCL9](http://genome-www4.stanford.edu/cgi-bin/SMD/source/sourceResult?choice=Gene&option=Name&criteria=CXCL9) | 1.79 | 0.0306 |
| A_23_P102551 | [MALL](http://genome-www4.stanford.edu/cgi-bin/SMD/source/sourceResult?choice=Gene&option=Name&criteria=MALL) | 1.51 | 0.0306 |
| A_24_P244800 | [NDRG2](http://genome-www4.stanford.edu/cgi-bin/SMD/source/sourceResult?choice=Gene&option=Name&criteria=NDRG2) | 1.51 | 0.0306 |
| A_24_P15973 | [A_24_P15973](http://genome-www4.stanford.edu/cgi-bin/SMD/source/sourceResult?choice=Gene&option=Name&criteria=A_24_P15973) | 1.53 | 0.0306 |
| A_23_P7827 | [RP1-93H18.5](http://genome-www4.stanford.edu/cgi-bin/SMD/source/sourceResult?choice=Gene&option=Name&criteria=RP1-93H18.5) | 1.55 | 0.0306 |
| A_23_P252817 | [SST](http://genome-www4.stanford.edu/cgi-bin/SMD/source/sourceResult?choice=Gene&option=Name&criteria=SST) | 1.57 | 0.0306 |
| A_23_P150457 | [XLKD1](http://genome-www4.stanford.edu/cgi-bin/SMD/source/sourceResult?choice=Gene&option=Name&criteria=XLKD1) | 1.54 | 0.0306 |
| A_24_P284959 | [PCDH18](http://genome-www4.stanford.edu/cgi-bin/SMD/source/sourceResult?choice=Gene&option=Name&criteria=PCDH18) | 1.56 | 0.0306 |
| A_24_P376483 | [HLA-A](http://genome-www4.stanford.edu/cgi-bin/SMD/source/sourceResult?choice=Gene&option=Name&criteria=HLA-A) | 1.60 | 0.0344 |
| A_23_P205959 | [ALDH1A3](http://genome-www4.stanford.edu/cgi-bin/SMD/source/sourceResult?choice=Gene&option=Name&criteria=ALDH1A3) | 1.60 | 0.0344 |
| A_24_P58673 | [REG4](http://genome-www4.stanford.edu/cgi-bin/SMD/source/sourceResult?choice=Gene&option=Name&criteria=REG4) | 1.54 | 0.0344 |
| A_23_P214408 | [UNC93A](http://genome-www4.stanford.edu/cgi-bin/SMD/source/sourceResult?choice=Gene&option=Name&criteria=UNC93A) | 1.72 | 0.0344 |
| A_24_P881527 | [CTNND1](http://genome-www4.stanford.edu/cgi-bin/SMD/source/sourceResult?choice=Gene&option=Name&criteria=CTNND1) | 1.53 | 0.0344 |
| A_23_P70539 | [HLA-C](http://genome-www4.stanford.edu/cgi-bin/SMD/source/sourceResult?choice=Gene&option=Name&criteria=HLA-C) | 1.59 | 0.0344 |
| A_32_P76137 | [ENST00000328018](http://genome-www4.stanford.edu/cgi-bin/SMD/source/sourceResult?choice=Gene&option=Name&criteria=ENST00000328018) | 1.57 | 0.0367 |
| A_23_P82886 | [DEFA6](http://genome-www4.stanford.edu/cgi-bin/SMD/source/sourceResult?choice=Gene&option=Name&criteria=DEFA6) | 1.55 | 0.0367 |
| A_24_P374863 | [RLTPR](http://genome-www4.stanford.edu/cgi-bin/SMD/source/sourceResult?choice=Gene&option=Name&criteria=RLTPR) | 1.53 | 0.0367 |
| A_32_P86763 | [TGM2](http://genome-www4.stanford.edu/cgi-bin/SMD/source/sourceResult?choice=Gene&option=Name&criteria=TGM2) | 1.55 | 0.0367 |
| A_23_P47102 | [ACY3](http://genome-www4.stanford.edu/cgi-bin/SMD/source/sourceResult?choice=Gene&option=Name&criteria=ACY3) | 1.54 | 0.0384 |
| A_23_P160940 | [ABCA4](http://genome-www4.stanford.edu/cgi-bin/SMD/source/sourceResult?choice=Gene&option=Name&criteria=ABCA4) | 1.51 | 0.0384 |
| A_24_P98723 | [LIN7C](http://genome-www4.stanford.edu/cgi-bin/SMD/source/sourceResult?choice=Gene&option=Name&criteria=LIN7C) | 1.51 | 0.0384 |
| A_23_P146233 | [LPL](http://genome-www4.stanford.edu/cgi-bin/SMD/source/sourceResult?choice=Gene&option=Name&criteria=LPL) | 1.60 | 0.0384 |
| A_32_P43664 | [AW136683](http://genome-www4.stanford.edu/cgi-bin/SMD/source/sourceResult?choice=Gene&option=Name&criteria=AW136683) | 1.50 | 0.0410 |
| A_24_P316454 | [BC022826](http://genome-www4.stanford.edu/cgi-bin/SMD/source/sourceResult?choice=Gene&option=Name&criteria=BC022826) | 1.61 | 0.0410 |
| A_23_P214267 | [GPR110](http://genome-www4.stanford.edu/cgi-bin/SMD/source/sourceResult?choice=Gene&option=Name&criteria=GPR110) | 1.56 | 0.0410 |
| A_24_P53778 | [ITLN2](http://genome-www4.stanford.edu/cgi-bin/SMD/source/sourceResult?choice=Gene&option=Name&criteria=ITLN2) | 1.58 | 0.0410 |
| A_24_P363711 | [DEFA6](http://genome-www4.stanford.edu/cgi-bin/SMD/source/sourceResult?choice=Gene&option=Name&criteria=DEFA6) | 1.51 | 0.0410 |
| A_23_P119936 | [REG3A](http://genome-www4.stanford.edu/cgi-bin/SMD/source/sourceResult?choice=Gene&option=Name&criteria=REG3A) | 1.52 | 0.0421 |
| A_23_P155786 | [SULT1E1](http://genome-www4.stanford.edu/cgi-bin/SMD/source/sourceResult?choice=Gene&option=Name&criteria=SULT1E1) | 1.68 | 0.0421 |
| A_23_P137238 | [SMCY](http://genome-www4.stanford.edu/cgi-bin/SMD/source/sourceResult?choice=Gene&option=Name&criteria=SMCY) | 1.63 | 0.0437 |
| A_24_P326084 | [HLA-DQA1](http://genome-www4.stanford.edu/cgi-bin/SMD/source/sourceResult?choice=Gene&option=Name&criteria=HLA-DQA1) | 1.81 | 0.0437 |
| A_23_P259314 | [RPS4Y1](http://genome-www4.stanford.edu/cgi-bin/SMD/source/sourceResult?choice=Gene&option=Name&criteria=RPS4Y1) | 1.67 | 0.0443 |
| A_23_P79518 | [IL1B](http://genome-www4.stanford.edu/cgi-bin/SMD/source/sourceResult?choice=Gene&option=Name&criteria=IL1B) | 1.52 | 0.0453 |
| A_23_P119763 | [ABCG5](http://genome-www4.stanford.edu/cgi-bin/SMD/source/sourceResult?choice=Gene&option=Name&criteria=ABCG5) | 1.60 | 0.0453 |
| A_23_P8913 | [CA2](http://genome-www4.stanford.edu/cgi-bin/SMD/source/sourceResult?choice=Gene&option=Name&criteria=CA2) | 1.86 | 0.0453 |
|  |  |  |  |
| **Supplementary Table S1B. Gene-probes downregulated in CD compared to Control (n=594)** | | | |
| **Agilent ID** | **Gene Name or Accession Number** | **Fold Change** | **q-value** |
| A_23_P115201 | [FCRL4](http://genome-www4.stanford.edu/cgi-bin/SMD/source/sourceResult?choice=Gene&option=Name&criteria=FCRL4) | 0.19 | <0.0001 |
| A_32_P34920 | [FOXD1](http://genome-www4.stanford.edu/cgi-bin/SMD/source/sourceResult?choice=Gene&option=Name&criteria=FOXD1) | 0.34 | <0.0001 |
| A_32_P216122 | [AK130891](http://genome-www4.stanford.edu/cgi-bin/SMD/source/sourceResult?choice=Gene&option=Name&criteria=AK130891) | 0.44 | <0.0001 |
| A_23_P362694 | [C4orf7](http://genome-www4.stanford.edu/cgi-bin/SMD/source/sourceResult?choice=Gene&option=Name&criteria=C4orf7) | 0.22 | <0.0001 |
| A_23_P121695 | [CXCL13](http://genome-www4.stanford.edu/cgi-bin/SMD/source/sourceResult?choice=Gene&option=Name&criteria=CXCL13) | 0.14 | <0.0001 |
| A_23_P357717 | [TCL1A](http://genome-www4.stanford.edu/cgi-bin/SMD/source/sourceResult?choice=Gene&option=Name&criteria=TCL1A) | 0.21 | <0.0001 |
| A_23_P57709 | [PCOLCE2](http://genome-www4.stanford.edu/cgi-bin/SMD/source/sourceResult?choice=Gene&option=Name&criteria=PCOLCE2) | 0.25 | <0.0001 |
| A_24_P174353 | [A_24_P174353](http://genome-www4.stanford.edu/cgi-bin/SMD/source/sourceResult?choice=Gene&option=Name&criteria=A_24_P174353) | 0.27 | <0.0001 |
| A_24_P940348 | [BCNP1](http://genome-www4.stanford.edu/cgi-bin/SMD/source/sourceResult?choice=Gene&option=Name&criteria=BCNP1) | 0.26 | <0.0001 |
| A_23_P366453 | [KHDRBS2](http://genome-www4.stanford.edu/cgi-bin/SMD/source/sourceResult?choice=Gene&option=Name&criteria=KHDRBS2) | 0.31 | <0.0001 |
| A_23_P115192 | [FCRL4](http://genome-www4.stanford.edu/cgi-bin/SMD/source/sourceResult?choice=Gene&option=Name&criteria=FCRL4) | 0.26 | <0.0001 |
| A_23_P214208 | [CNR1](http://genome-www4.stanford.edu/cgi-bin/SMD/source/sourceResult?choice=Gene&option=Name&criteria=CNR1) | 0.32 | <0.0001 |
| A_24_P621701 | [THC2397697](http://genome-www4.stanford.edu/cgi-bin/SMD/source/sourceResult?choice=Gene&option=Name&criteria=THC2397697) | 0.30 | <0.0001 |
| A_32_P172545 | [THC2315069](http://genome-www4.stanford.edu/cgi-bin/SMD/source/sourceResult?choice=Gene&option=Name&criteria=THC2315069) | 0.49 | <0.0001 |
| A_32_P71876 | [THC2438936](http://genome-www4.stanford.edu/cgi-bin/SMD/source/sourceResult?choice=Gene&option=Name&criteria=THC2438936) | 0.32 | <0.0001 |
| A_23_P124542 | [CR2](http://genome-www4.stanford.edu/cgi-bin/SMD/source/sourceResult?choice=Gene&option=Name&criteria=CR2) | 0.33 | <0.0001 |
| A_23_P138524 | [CPXM2](http://genome-www4.stanford.edu/cgi-bin/SMD/source/sourceResult?choice=Gene&option=Name&criteria=CPXM2) | 0.45 | <0.0001 |
| A_23_P30634 | [BACH2](http://genome-www4.stanford.edu/cgi-bin/SMD/source/sourceResult?choice=Gene&option=Name&criteria=BACH2) | 0.49 | <0.0001 |
| A_23_P115200 | [FCRL4](http://genome-www4.stanford.edu/cgi-bin/SMD/source/sourceResult?choice=Gene&option=Name&criteria=FCRL4) | 0.27 | <0.0001 |
| A_23_P372478 | [SERPINA9](http://genome-www4.stanford.edu/cgi-bin/SMD/source/sourceResult?choice=Gene&option=Name&criteria=SERPINA9) | 0.40 | <0.0001 |
| A_32_P174285 | [THC2442021](http://genome-www4.stanford.edu/cgi-bin/SMD/source/sourceResult?choice=Gene&option=Name&criteria=THC2442021) | 0.42 | <0.0001 |
| A_32_P33213 | [THC2358845](http://genome-www4.stanford.edu/cgi-bin/SMD/source/sourceResult?choice=Gene&option=Name&criteria=THC2358845) | 0.44 | <0.0001 |
| A_23_P339079 | [ZNF573](http://genome-www4.stanford.edu/cgi-bin/SMD/source/sourceResult?choice=Gene&option=Name&criteria=ZNF573) | 0.57 | <0.0001 |
| A_23_P46039 | [FCRLM1](http://genome-www4.stanford.edu/cgi-bin/SMD/source/sourceResult?choice=Gene&option=Name&criteria=FCRLM1) | 0.33 | <0.0001 |
| A_24_P916364 | [AK025613](http://genome-www4.stanford.edu/cgi-bin/SMD/source/sourceResult?choice=Gene&option=Name&criteria=AK025613) | 0.56 | <0.0001 |
| A_24_P883109 | [TYW1](http://genome-www4.stanford.edu/cgi-bin/SMD/source/sourceResult?choice=Gene&option=Name&criteria=TYW1) | 0.54 | <0.0001 |
| A_23_P166371 | [VPREB3](http://genome-www4.stanford.edu/cgi-bin/SMD/source/sourceResult?choice=Gene&option=Name&criteria=VPREB3) | 0.47 | <0.0001 |
| A_23_P149368 | [FCRL1](http://genome-www4.stanford.edu/cgi-bin/SMD/source/sourceResult?choice=Gene&option=Name&criteria=FCRL1) | 0.37 | <0.0001 |
| A_23_P10232 | [BANK1](http://genome-www4.stanford.edu/cgi-bin/SMD/source/sourceResult?choice=Gene&option=Name&criteria=BANK1) | 0.32 | <0.0001 |
| A_23_P359174 | [BC069659](http://genome-www4.stanford.edu/cgi-bin/SMD/source/sourceResult?choice=Gene&option=Name&criteria=BC069659) | 0.58 | <0.0001 |
| A_32_P208200 | [THC2393794](http://genome-www4.stanford.edu/cgi-bin/SMD/source/sourceResult?choice=Gene&option=Name&criteria=THC2393794) | 0.43 | <0.0001 |
| A_23_P358438 | [FCRL3](http://genome-www4.stanford.edu/cgi-bin/SMD/source/sourceResult?choice=Gene&option=Name&criteria=FCRL3) | 0.38 | <0.0001 |
| A_23_P116371 | [MS4A1](http://genome-www4.stanford.edu/cgi-bin/SMD/source/sourceResult?choice=Gene&option=Name&criteria=MS4A1) | 0.32 | <0.0001 |
| A_23_P434430 | [ZNF439](http://genome-www4.stanford.edu/cgi-bin/SMD/source/sourceResult?choice=Gene&option=Name&criteria=ZNF439) | 0.51 | <0.0001 |
| A_32_P45375 | [AF037219](http://genome-www4.stanford.edu/cgi-bin/SMD/source/sourceResult?choice=Gene&option=Name&criteria=AF037219) | 0.51 | <0.0001 |
| A_23_P2041 | [MICALCL](http://genome-www4.stanford.edu/cgi-bin/SMD/source/sourceResult?choice=Gene&option=Name&criteria=MICALCL) | 0.53 | <0.0001 |
| A_24_P759584 | [DIO3OS](http://genome-www4.stanford.edu/cgi-bin/SMD/source/sourceResult?choice=Gene&option=Name&criteria=DIO3OS) | 0.50 | <0.0001 |
| A_24_P133905 | [CCL23](http://genome-www4.stanford.edu/cgi-bin/SMD/source/sourceResult?choice=Gene&option=Name&criteria=CCL23) | 0.34 | <0.0001 |
| A_24_P252945 | [BLR1](http://genome-www4.stanford.edu/cgi-bin/SMD/source/sourceResult?choice=Gene&option=Name&criteria=BLR1) | 0.35 | <0.0001 |
| A_23_P113572 | [CD19](http://genome-www4.stanford.edu/cgi-bin/SMD/source/sourceResult?choice=Gene&option=Name&criteria=CD19) | 0.39 | <0.0001 |
| A_24_P910490 | [THC2340757](http://genome-www4.stanford.edu/cgi-bin/SMD/source/sourceResult?choice=Gene&option=Name&criteria=THC2340757) | 0.54 | <0.0001 |
| A_32_P146844 | [THC2406576](http://genome-www4.stanford.edu/cgi-bin/SMD/source/sourceResult?choice=Gene&option=Name&criteria=THC2406576) | 0.61 | <0.0001 |
| A_23_P87049 | [SORL1](http://genome-www4.stanford.edu/cgi-bin/SMD/source/sourceResult?choice=Gene&option=Name&criteria=SORL1) | 0.58 | <0.0001 |
| A_32_P198620 | [THC2438685](http://genome-www4.stanford.edu/cgi-bin/SMD/source/sourceResult?choice=Gene&option=Name&criteria=THC2438685) | 0.55 | <0.0001 |
| A_24_P465879 | [AK026466](http://genome-www4.stanford.edu/cgi-bin/SMD/source/sourceResult?choice=Gene&option=Name&criteria=AK026466) | 0.49 | <0.0001 |
| A_24_P913216 | [AK022299](http://genome-www4.stanford.edu/cgi-bin/SMD/source/sourceResult?choice=Gene&option=Name&criteria=AK022299) | 0.55 | <0.0001 |
| A_23_P500093 | [RGS13](http://genome-www4.stanford.edu/cgi-bin/SMD/source/sourceResult?choice=Gene&option=Name&criteria=RGS13) | 0.33 | <0.0001 |
| A_23_P160751 | [FCRL2](http://genome-www4.stanford.edu/cgi-bin/SMD/source/sourceResult?choice=Gene&option=Name&criteria=FCRL2) | 0.37 | <0.0001 |
| A_23_P163251 | [PAQR5](http://genome-www4.stanford.edu/cgi-bin/SMD/source/sourceResult?choice=Gene&option=Name&criteria=PAQR5) | 0.43 | <0.0001 |
| A_32_P37584 | [AW340352](http://genome-www4.stanford.edu/cgi-bin/SMD/source/sourceResult?choice=Gene&option=Name&criteria=AW340352) | 0.49 | <0.0001 |
| A_23_P170679 | [COL4A3](http://genome-www4.stanford.edu/cgi-bin/SMD/source/sourceResult?choice=Gene&option=Name&criteria=COL4A3) | 0.40 | <0.0001 |
| A_32_P105110 | [AK057196](http://genome-www4.stanford.edu/cgi-bin/SMD/source/sourceResult?choice=Gene&option=Name&criteria=AK057196) | 0.58 | <0.0001 |
| A_23_P110492 | MARCH6 | 0.56 | <0.0001 |
| A_23_P4551 | [SETBP1](http://genome-www4.stanford.edu/cgi-bin/SMD/source/sourceResult?choice=Gene&option=Name&criteria=SETBP1) | 0.55 | <0.0001 |
| A_32_P40463 | [NUDT9P1](http://genome-www4.stanford.edu/cgi-bin/SMD/source/sourceResult?choice=Gene&option=Name&criteria=NUDT9P1) | 0.55 | <0.0001 |
| A_32_P48054 | [ENST00000374472](http://genome-www4.stanford.edu/cgi-bin/SMD/source/sourceResult?choice=Gene&option=Name&criteria=ENST00000374472) | 0.37 | <0.0001 |
| A_32_P138409 | [THC2442489](http://genome-www4.stanford.edu/cgi-bin/SMD/source/sourceResult?choice=Gene&option=Name&criteria=THC2442489) | 0.53 | <0.0001 |
| A_32_P90685 | [THC2435791](http://genome-www4.stanford.edu/cgi-bin/SMD/source/sourceResult?choice=Gene&option=Name&criteria=THC2435791) | 0.52 | <0.0001 |
| A_32_P206549 | [BC035156](http://genome-www4.stanford.edu/cgi-bin/SMD/source/sourceResult?choice=Gene&option=Name&criteria=BC035156) | 0.59 | <0.0001 |
| A_23_P15876 | [ALPK2](http://genome-www4.stanford.edu/cgi-bin/SMD/source/sourceResult?choice=Gene&option=Name&criteria=ALPK2) | 0.36 | <0.0001 |
| A_32_P19193 | [THC2416695](http://genome-www4.stanford.edu/cgi-bin/SMD/source/sourceResult?choice=Gene&option=Name&criteria=THC2416695) | 0.56 | <0.0001 |
| A_23_P36641 | [AICDA](http://genome-www4.stanford.edu/cgi-bin/SMD/source/sourceResult?choice=Gene&option=Name&criteria=AICDA) | 0.35 | <0.0001 |
| A_24_P923922 | [LOC400509](http://genome-www4.stanford.edu/cgi-bin/SMD/source/sourceResult?choice=Gene&option=Name&criteria=LOC400509) | 0.61 | <0.0001 |
| A_23_P310931 | [CNR2](http://genome-www4.stanford.edu/cgi-bin/SMD/source/sourceResult?choice=Gene&option=Name&criteria=CNR2) | 0.51 | <0.0001 |
| A_23_P253791 | [CAMP](http://genome-www4.stanford.edu/cgi-bin/SMD/source/sourceResult?choice=Gene&option=Name&criteria=CAMP) | 0.33 | <0.0001 |
| A_23_P363316 | [HOXB5](http://genome-www4.stanford.edu/cgi-bin/SMD/source/sourceResult?choice=Gene&option=Name&criteria=HOXB5) | 0.51 | <0.0001 |
| A_23_P98910 | [LRMP](http://genome-www4.stanford.edu/cgi-bin/SMD/source/sourceResult?choice=Gene&option=Name&criteria=LRMP) | 0.52 | <0.0001 |
| A_24_P264928 | [FAM44A](http://genome-www4.stanford.edu/cgi-bin/SMD/source/sourceResult?choice=Gene&option=Name&criteria=FAM44A) | 0.52 | <0.0001 |
| A_32_P13168 | [BX115853](http://genome-www4.stanford.edu/cgi-bin/SMD/source/sourceResult?choice=Gene&option=Name&criteria=BX115853) | 0.40 | <0.0001 |
| A_23_P258088 | [PACSIN1](http://genome-www4.stanford.edu/cgi-bin/SMD/source/sourceResult?choice=Gene&option=Name&criteria=PACSIN1) | 0.52 | <0.0001 |
| A_24_P693433 | [AK022364](http://genome-www4.stanford.edu/cgi-bin/SMD/source/sourceResult?choice=Gene&option=Name&criteria=AK022364) | 0.59 | <0.0001 |
| A_23_P7185 | [BRDG1](http://genome-www4.stanford.edu/cgi-bin/SMD/source/sourceResult?choice=Gene&option=Name&criteria=BRDG1) | 0.35 | <0.0001 |
| A_23_P99253 | [LIN7A](http://genome-www4.stanford.edu/cgi-bin/SMD/source/sourceResult?choice=Gene&option=Name&criteria=LIN7A) | 0.51 | <0.0001 |
| A_24_P911960 | [BM664910](http://genome-www4.stanford.edu/cgi-bin/SMD/source/sourceResult?choice=Gene&option=Name&criteria=BM664910) | 0.65 | <0.0001 |
| A_32_P187304 | [THC2408868](http://genome-www4.stanford.edu/cgi-bin/SMD/source/sourceResult?choice=Gene&option=Name&criteria=THC2408868) | 0.64 | <0.0001 |
| A_24_P741023 | [BC008476](http://genome-www4.stanford.edu/cgi-bin/SMD/source/sourceResult?choice=Gene&option=Name&criteria=BC008476) | 0.52 | <0.0001 |
| A_32_P209582 | [LOC645238](http://genome-www4.stanford.edu/cgi-bin/SMD/source/sourceResult?choice=Gene&option=Name&criteria=LOC645238) | 0.60 | <0.0001 |
| A_32_P230537 | [A_32_P230537](http://genome-www4.stanford.edu/cgi-bin/SMD/source/sourceResult?choice=Gene&option=Name&criteria=A_32_P230537) | 0.53 | <0.0001 |
| A_32_P133038 | [THC2358845](http://genome-www4.stanford.edu/cgi-bin/SMD/source/sourceResult?choice=Gene&option=Name&criteria=THC2358845) | 0.48 | <0.0001 |
| A_23_P381746 | [KIAA1713](http://genome-www4.stanford.edu/cgi-bin/SMD/source/sourceResult?choice=Gene&option=Name&criteria=KIAA1713) | 0.53 | <0.0001 |
| A_32_P185701 | [C11orf67](http://genome-www4.stanford.edu/cgi-bin/SMD/source/sourceResult?choice=Gene&option=Name&criteria=C11orf67) | 0.55 | <0.0001 |
| A_24_P867201 | [CR613944](http://genome-www4.stanford.edu/cgi-bin/SMD/source/sourceResult?choice=Gene&option=Name&criteria=CR613944) | 0.44 | <0.0001 |
| A_24_P136497 | [DENND4A](http://genome-www4.stanford.edu/cgi-bin/SMD/source/sourceResult?choice=Gene&option=Name&criteria=DENND4A) | 0.66 | <0.0001 |
| A_24_P629883 | [UNQ6488](http://genome-www4.stanford.edu/cgi-bin/SMD/source/sourceResult?choice=Gene&option=Name&criteria=UNQ6488) | 0.58 | <0.0001 |
| A_23_P33583 | [DNAH7](http://genome-www4.stanford.edu/cgi-bin/SMD/source/sourceResult?choice=Gene&option=Name&criteria=DNAH7) | 0.56 | <0.0001 |
| A_24_P98210 | [TFEC](http://genome-www4.stanford.edu/cgi-bin/SMD/source/sourceResult?choice=Gene&option=Name&criteria=TFEC) | 0.54 | <0.0001 |
| A_24_P462330 | [BF433725](http://genome-www4.stanford.edu/cgi-bin/SMD/source/sourceResult?choice=Gene&option=Name&criteria=BF433725) | 0.62 | <0.0001 |
| A_23_P140405 | [CHES1](http://genome-www4.stanford.edu/cgi-bin/SMD/source/sourceResult?choice=Gene&option=Name&criteria=CHES1) | 0.66 | <0.0001 |
| A_24_P276576 | [FCRLM1](http://genome-www4.stanford.edu/cgi-bin/SMD/source/sourceResult?choice=Gene&option=Name&criteria=FCRLM1) | 0.32 | <0.0001 |
| A_24_P930337 | [A_24_P930337](http://genome-www4.stanford.edu/cgi-bin/SMD/source/sourceResult?choice=Gene&option=Name&criteria=A_24_P930337) | 0.59 | <0.0001 |
| A_32_P164573 | [THC2314215](http://genome-www4.stanford.edu/cgi-bin/SMD/source/sourceResult?choice=Gene&option=Name&criteria=THC2314215) | 0.58 | <0.0001 |
| A_23_P93772 | [HOXA5](http://genome-www4.stanford.edu/cgi-bin/SMD/source/sourceResult?choice=Gene&option=Name&criteria=HOXA5) | 0.49 | <0.0001 |
| A_23_P427014 | [CLDN8](http://genome-www4.stanford.edu/cgi-bin/SMD/source/sourceResult?choice=Gene&option=Name&criteria=CLDN8) | 0.30 | <0.0001 |
| A_32_P211026 | [THC2342793](http://genome-www4.stanford.edu/cgi-bin/SMD/source/sourceResult?choice=Gene&option=Name&criteria=THC2342793) | 0.55 | <0.0001 |
| A_23_P435183 | [ENST00000308482](http://genome-www4.stanford.edu/cgi-bin/SMD/source/sourceResult?choice=Gene&option=Name&criteria=ENST00000308482) | 0.56 | <0.0001 |
| A_32_P71171 | [A_32_P71171](http://genome-www4.stanford.edu/cgi-bin/SMD/source/sourceResult?choice=Gene&option=Name&criteria=A_32_P71171) | 0.54 | <0.0001 |
| A_32_P125589 | [THC2343933](http://genome-www4.stanford.edu/cgi-bin/SMD/source/sourceResult?choice=Gene&option=Name&criteria=THC2343933) | 0.52 | <0.0001 |
| A_24_P606239 | [AK056073](http://genome-www4.stanford.edu/cgi-bin/SMD/source/sourceResult?choice=Gene&option=Name&criteria=AK056073) | 0.56 | <0.0001 |
| A_23_P405707 | [BCOR](http://genome-www4.stanford.edu/cgi-bin/SMD/source/sourceResult?choice=Gene&option=Name&criteria=BCOR) | 0.60 | <0.0001 |
| A_23_P39766 | [GLS](http://genome-www4.stanford.edu/cgi-bin/SMD/source/sourceResult?choice=Gene&option=Name&criteria=GLS) | 0.55 | <0.0001 |
| A_32_P162862 | [LOC644891](http://genome-www4.stanford.edu/cgi-bin/SMD/source/sourceResult?choice=Gene&option=Name&criteria=LOC644891) | 0.50 | <0.0001 |
| A_23_P40896 | [SLC25A36](http://genome-www4.stanford.edu/cgi-bin/SMD/source/sourceResult?choice=Gene&option=Name&criteria=SLC25A36) | 0.61 | <0.0001 |
| A_32_P3783 | [THC2376586](http://genome-www4.stanford.edu/cgi-bin/SMD/source/sourceResult?choice=Gene&option=Name&criteria=THC2376586) | 0.58 | <0.0001 |
| A_32_P5542 | [AF131782](http://genome-www4.stanford.edu/cgi-bin/SMD/source/sourceResult?choice=Gene&option=Name&criteria=AF131782) | 0.57 | <0.0001 |
| A_32_P72067 | [ARHGAP24](http://genome-www4.stanford.edu/cgi-bin/SMD/source/sourceResult?choice=Gene&option=Name&criteria=ARHGAP24) | 0.50 | <0.0001 |
| A_32_P228886 | [BX115350](http://genome-www4.stanford.edu/cgi-bin/SMD/source/sourceResult?choice=Gene&option=Name&criteria=BX115350) | 0.53 | <0.0001 |
| A_24_P910372 | [THC2349681](http://genome-www4.stanford.edu/cgi-bin/SMD/source/sourceResult?choice=Gene&option=Name&criteria=THC2349681) | 0.54 | <0.0001 |
| A_23_P157495 | [PPP3CC](http://genome-www4.stanford.edu/cgi-bin/SMD/source/sourceResult?choice=Gene&option=Name&criteria=PPP3CC) | 0.66 | <0.0001 |
| A_23_P423074 | [KIAA0888](http://genome-www4.stanford.edu/cgi-bin/SMD/source/sourceResult?choice=Gene&option=Name&criteria=KIAA0888) | 0.46 | <0.0001 |
| A_32_P193908 | [BM968705](http://genome-www4.stanford.edu/cgi-bin/SMD/source/sourceResult?choice=Gene&option=Name&criteria=BM968705) | 0.66 | <0.0001 |
| A_24_P916816 | [SP100](http://genome-www4.stanford.edu/cgi-bin/SMD/source/sourceResult?choice=Gene&option=Name&criteria=SP100) | 0.56 | <0.0001 |
| A_32_P83811 | [AK124936](http://genome-www4.stanford.edu/cgi-bin/SMD/source/sourceResult?choice=Gene&option=Name&criteria=AK124936) | 0.49 | <0.0001 |
| A_32_P974 | [FLJ16734](http://genome-www4.stanford.edu/cgi-bin/SMD/source/sourceResult?choice=Gene&option=Name&criteria=FLJ16734) | 0.63 | <0.0001 |
| A_23_P111811 | [ING3](http://genome-www4.stanford.edu/cgi-bin/SMD/source/sourceResult?choice=Gene&option=Name&criteria=ING3) | 0.65 | <0.0001 |
| A_24_P148263 | [A_24_P148263](http://genome-www4.stanford.edu/cgi-bin/SMD/source/sourceResult?choice=Gene&option=Name&criteria=A_24_P148263) | 0.48 | <0.0001 |
| A_24_P307665 | [AK021848](http://genome-www4.stanford.edu/cgi-bin/SMD/source/sourceResult?choice=Gene&option=Name&criteria=AK021848) | 0.61 | <0.0001 |
| A_23_P75915 | [RIC3](http://genome-www4.stanford.edu/cgi-bin/SMD/source/sourceResult?choice=Gene&option=Name&criteria=RIC3) | 0.53 | <0.0001 |
| A_32_P177725 | [THC2416098](http://genome-www4.stanford.edu/cgi-bin/SMD/source/sourceResult?choice=Gene&option=Name&criteria=THC2416098) | 0.58 | <0.0001 |
| A_24_P922858 | [BF727393](http://genome-www4.stanford.edu/cgi-bin/SMD/source/sourceResult?choice=Gene&option=Name&criteria=BF727393) | 0.60 | <0.0001 |
| A_23_P166848 | [LTF](http://genome-www4.stanford.edu/cgi-bin/SMD/source/sourceResult?choice=Gene&option=Name&criteria=LTF) | 0.43 | <0.0001 |
| A_32_P201150 | [THC2399007](http://genome-www4.stanford.edu/cgi-bin/SMD/source/sourceResult?choice=Gene&option=Name&criteria=THC2399007) | 0.66 | <0.0001 |
| A_24_P529786 | [AK091744](http://genome-www4.stanford.edu/cgi-bin/SMD/source/sourceResult?choice=Gene&option=Name&criteria=AK091744) | 0.61 | <0.0001 |
| A_32_P92117 | [BE165955](http://genome-www4.stanford.edu/cgi-bin/SMD/source/sourceResult?choice=Gene&option=Name&criteria=BE165955) | 0.62 | <0.0001 |
| A_32_P385 | [THC2317278](http://genome-www4.stanford.edu/cgi-bin/SMD/source/sourceResult?choice=Gene&option=Name&criteria=THC2317278) | 0.51 | <0.0001 |
| A_24_P926025 | [DKFZp547E087](http://genome-www4.stanford.edu/cgi-bin/SMD/source/sourceResult?choice=Gene&option=Name&criteria=DKFZp547E087) | 0.52 | <0.0001 |
| A_23_P31725 | [BLK](http://genome-www4.stanford.edu/cgi-bin/SMD/source/sourceResult?choice=Gene&option=Name&criteria=BLK) | 0.39 | <0.0001 |
| A_32_P6452 | [A_32_P6452](http://genome-www4.stanford.edu/cgi-bin/SMD/source/sourceResult?choice=Gene&option=Name&criteria=A_32_P6452) | 0.66 | <0.0001 |
| A_23_P152926 | [GP1BA](http://genome-www4.stanford.edu/cgi-bin/SMD/source/sourceResult?choice=Gene&option=Name&criteria=GP1BA) | 0.40 | <0.0001 |
| A_32_P208039 | [THC2314205](http://genome-www4.stanford.edu/cgi-bin/SMD/source/sourceResult?choice=Gene&option=Name&criteria=THC2314205) | 0.65 | <0.0001 |
| A_24_P366777 | [NOTCH2NL](http://genome-www4.stanford.edu/cgi-bin/SMD/source/sourceResult?choice=Gene&option=Name&criteria=NOTCH2NL) | 0.65 | <0.0001 |
| A_32_P48198 | [LOC400509](http://genome-www4.stanford.edu/cgi-bin/SMD/source/sourceResult?choice=Gene&option=Name&criteria=LOC400509) | 0.60 | <0.0001 |
| A_24_P922261 | [SRGAP1](http://genome-www4.stanford.edu/cgi-bin/SMD/source/sourceResult?choice=Gene&option=Name&criteria=SRGAP1) | 0.58 | <0.0001 |
| A_32_P198282 | [THC2381045](http://genome-www4.stanford.edu/cgi-bin/SMD/source/sourceResult?choice=Gene&option=Name&criteria=THC2381045) | 0.61 | <0.0001 |
| A_24_P194886 | [EHBP1](http://genome-www4.stanford.edu/cgi-bin/SMD/source/sourceResult?choice=Gene&option=Name&criteria=EHBP1) | 0.61 | <0.0001 |
| A_23_P370830 | [KLHL14](http://genome-www4.stanford.edu/cgi-bin/SMD/source/sourceResult?choice=Gene&option=Name&criteria=KLHL14) | 0.42 | <0.0001 |
| A_23_P345799 | [BCNP1](http://genome-www4.stanford.edu/cgi-bin/SMD/source/sourceResult?choice=Gene&option=Name&criteria=BCNP1) | 0.54 | <0.0001 |
| A_23_P155257 | [FOXP1](http://genome-www4.stanford.edu/cgi-bin/SMD/source/sourceResult?choice=Gene&option=Name&criteria=FOXP1) | 0.65 | <0.0001 |
| A_24_P37020 | [THC2282321](http://genome-www4.stanford.edu/cgi-bin/SMD/source/sourceResult?choice=Gene&option=Name&criteria=THC2282321) | 0.53 | <0.0001 |
| A_32_P233834 | [THC2392085](http://genome-www4.stanford.edu/cgi-bin/SMD/source/sourceResult?choice=Gene&option=Name&criteria=THC2392085) | 0.65 | <0.0001 |
| A_23_P336342 | [AK124576](http://genome-www4.stanford.edu/cgi-bin/SMD/source/sourceResult?choice=Gene&option=Name&criteria=AK124576) | 0.64 | <0.0001 |
| A_32_P88987 | [AK022346](http://genome-www4.stanford.edu/cgi-bin/SMD/source/sourceResult?choice=Gene&option=Name&criteria=AK022346) | 0.63 | <0.0001 |
| A_32_P233250 | [THC2404896](http://genome-www4.stanford.edu/cgi-bin/SMD/source/sourceResult?choice=Gene&option=Name&criteria=THC2404896) | 0.66 | <0.0001 |
| A_23_P6561 | [FLJ10213](http://genome-www4.stanford.edu/cgi-bin/SMD/source/sourceResult?choice=Gene&option=Name&criteria=FLJ10213) | 0.65 | <0.0001 |
| A_24_P140204 | [PXK](http://genome-www4.stanford.edu/cgi-bin/SMD/source/sourceResult?choice=Gene&option=Name&criteria=PXK) | 0.60 | <0.0001 |
| A_24_P920904 | [C8orf59](http://genome-www4.stanford.edu/cgi-bin/SMD/source/sourceResult?choice=Gene&option=Name&criteria=C8orf59) | 0.61 | <0.0001 |
| A_23_P382302 | [RIF1](http://genome-www4.stanford.edu/cgi-bin/SMD/source/sourceResult?choice=Gene&option=Name&criteria=RIF1) | 0.54 | <0.0001 |
| A_23_P216038 | [PHF20L1](http://genome-www4.stanford.edu/cgi-bin/SMD/source/sourceResult?choice=Gene&option=Name&criteria=PHF20L1) | 0.65 | <0.0001 |
| A_24_P182947 | [GCET2](http://genome-www4.stanford.edu/cgi-bin/SMD/source/sourceResult?choice=Gene&option=Name&criteria=GCET2) | 0.43 | <0.0001 |
| A_24_P26114 | [OXSR1](http://genome-www4.stanford.edu/cgi-bin/SMD/source/sourceResult?choice=Gene&option=Name&criteria=OXSR1) | 0.63 | <0.0001 |
| A_23_P2901 | [C14orf113](http://genome-www4.stanford.edu/cgi-bin/SMD/source/sourceResult?choice=Gene&option=Name&criteria=C14orf113) | 0.58 | <0.0001 |
| A_23_P425932 | [VTI1A](http://genome-www4.stanford.edu/cgi-bin/SMD/source/sourceResult?choice=Gene&option=Name&criteria=VTI1A) | 0.63 | <0.0001 |
| A_32_P73071 | [THC2453866](http://genome-www4.stanford.edu/cgi-bin/SMD/source/sourceResult?choice=Gene&option=Name&criteria=THC2453866) | 0.62 | <0.0001 |
| A_24_P941708 | [RUFY2](http://genome-www4.stanford.edu/cgi-bin/SMD/source/sourceResult?choice=Gene&option=Name&criteria=RUFY2) | 0.63 | <0.0001 |
| A_32_P122715 | [THC2280867](http://genome-www4.stanford.edu/cgi-bin/SMD/source/sourceResult?choice=Gene&option=Name&criteria=THC2280867) | 0.61 | <0.0001 |
| A_24_P188056 | [PARP11](http://genome-www4.stanford.edu/cgi-bin/SMD/source/sourceResult?choice=Gene&option=Name&criteria=PARP11) | 0.54 | <0.0001 |
| A_32_P73304 | [THC2360305](http://genome-www4.stanford.edu/cgi-bin/SMD/source/sourceResult?choice=Gene&option=Name&criteria=THC2360305) | 0.60 | <0.0001 |
| A_32_P122579 | [THC2284074](http://genome-www4.stanford.edu/cgi-bin/SMD/source/sourceResult?choice=Gene&option=Name&criteria=THC2284074) | 0.56 | <0.0001 |
| A_24_P314477 | [TUBB2B](http://genome-www4.stanford.edu/cgi-bin/SMD/source/sourceResult?choice=Gene&option=Name&criteria=TUBB2B) | 0.50 | <0.0001 |
| A_23_P359052 | [FAM44A](http://genome-www4.stanford.edu/cgi-bin/SMD/source/sourceResult?choice=Gene&option=Name&criteria=FAM44A) | 0.57 | <0.0001 |
| A_32_P216004 | [KCTD9](http://genome-www4.stanford.edu/cgi-bin/SMD/source/sourceResult?choice=Gene&option=Name&criteria=KCTD9) | 0.54 | <0.0001 |
| A_24_P50829 | [TRPM7](http://genome-www4.stanford.edu/cgi-bin/SMD/source/sourceResult?choice=Gene&option=Name&criteria=TRPM7) | 0.51 | <0.0001 |
| A_24_P943393 | [AHNAK](http://genome-www4.stanford.edu/cgi-bin/SMD/source/sourceResult?choice=Gene&option=Name&criteria=AHNAK) | 0.65 | <0.0001 |
| A_23_P48387 | [APRIN](http://genome-www4.stanford.edu/cgi-bin/SMD/source/sourceResult?choice=Gene&option=Name&criteria=APRIN) | 0.63 | <0.0001 |
| A_32_P138503 | [THC2449800](http://genome-www4.stanford.edu/cgi-bin/SMD/source/sourceResult?choice=Gene&option=Name&criteria=THC2449800) | 0.54 | <0.0001 |
| A_32_P85880 | [A_32_P85880](http://genome-www4.stanford.edu/cgi-bin/SMD/source/sourceResult?choice=Gene&option=Name&criteria=A_32_P85880) | 0.61 | <0.0001 |
| A_32_P123106 | [PAPOLG](http://genome-www4.stanford.edu/cgi-bin/SMD/source/sourceResult?choice=Gene&option=Name&criteria=PAPOLG) | 0.63 | <0.0001 |
| A_23_P157679 | [C8orf53](http://genome-www4.stanford.edu/cgi-bin/SMD/source/sourceResult?choice=Gene&option=Name&criteria=C8orf53) | 0.65 | <0.0001 |
| A_23_P70069 | [AK000420](http://genome-www4.stanford.edu/cgi-bin/SMD/source/sourceResult?choice=Gene&option=Name&criteria=AK000420) | 0.59 | <0.0001 |
| A_23_P301530 | [ANK3](http://genome-www4.stanford.edu/cgi-bin/SMD/source/sourceResult?choice=Gene&option=Name&criteria=ANK3) | 0.66 | <0.0001 |
| A_23_P10025 | [NELL2](http://genome-www4.stanford.edu/cgi-bin/SMD/source/sourceResult?choice=Gene&option=Name&criteria=NELL2) | 0.54 | <0.0001 |
| A_24_P347447 | [DAAM1](http://genome-www4.stanford.edu/cgi-bin/SMD/source/sourceResult?choice=Gene&option=Name&criteria=DAAM1) | 0.65 | <0.0001 |
| A_32_P181271 | [THC2323620](http://genome-www4.stanford.edu/cgi-bin/SMD/source/sourceResult?choice=Gene&option=Name&criteria=THC2323620) | 0.56 | <0.0001 |
| A_24_P932388 | [DB340110](http://genome-www4.stanford.edu/cgi-bin/SMD/source/sourceResult?choice=Gene&option=Name&criteria=DB340110) | 0.50 | <0.0001 |
| A_24_P302833 | [APPL](http://genome-www4.stanford.edu/cgi-bin/SMD/source/sourceResult?choice=Gene&option=Name&criteria=APPL) | 0.63 | <0.0001 |
| A_32_P55427 | [THC2319816](http://genome-www4.stanford.edu/cgi-bin/SMD/source/sourceResult?choice=Gene&option=Name&criteria=THC2319816) | 0.58 | <0.0001 |
| A_24_P925211 | [AK125129](http://genome-www4.stanford.edu/cgi-bin/SMD/source/sourceResult?choice=Gene&option=Name&criteria=AK125129) | 0.66 | <0.0001 |
| A_32_P118556 | [A_32_P118556](http://genome-www4.stanford.edu/cgi-bin/SMD/source/sourceResult?choice=Gene&option=Name&criteria=A_32_P118556) | 0.60 | <0.0001 |
| A_32_P158723 | [AK123861](http://genome-www4.stanford.edu/cgi-bin/SMD/source/sourceResult?choice=Gene&option=Name&criteria=AK123861) | 0.60 | <0.0001 |
| A_23_P7503 | [TIMD4](http://genome-www4.stanford.edu/cgi-bin/SMD/source/sourceResult?choice=Gene&option=Name&criteria=TIMD4) | 0.36 | <0.0001 |
| A_32_P34703 | [THC2377375](http://genome-www4.stanford.edu/cgi-bin/SMD/source/sourceResult?choice=Gene&option=Name&criteria=THC2377375) | 0.58 | <0.0001 |
| A_32_P25243 | [THC2316980](http://genome-www4.stanford.edu/cgi-bin/SMD/source/sourceResult?choice=Gene&option=Name&criteria=THC2316980) | 0.63 | <0.0001 |
| A_24_P109432 | [ENST00000339968](http://genome-www4.stanford.edu/cgi-bin/SMD/source/sourceResult?choice=Gene&option=Name&criteria=ENST00000339968) | 0.52 | <0.0001 |
| A_32_P206391 | [A_32_P206391](http://genome-www4.stanford.edu/cgi-bin/SMD/source/sourceResult?choice=Gene&option=Name&criteria=A_32_P206391) | 0.63 | <0.0001 |
| A_23_P66137 | [SOX8](http://genome-www4.stanford.edu/cgi-bin/SMD/source/sourceResult?choice=Gene&option=Name&criteria=SOX8) | 0.63 | <0.0001 |
| A_23_P25069 | [OVOS2](http://genome-www4.stanford.edu/cgi-bin/SMD/source/sourceResult?choice=Gene&option=Name&criteria=OVOS2) | 0.57 | <0.0001 |
| A_23_P201510 | [FCAMR](http://genome-www4.stanford.edu/cgi-bin/SMD/source/sourceResult?choice=Gene&option=Name&criteria=FCAMR) | 0.65 | <0.0001 |
| A_32_P95015 | [THC2404993](http://genome-www4.stanford.edu/cgi-bin/SMD/source/sourceResult?choice=Gene&option=Name&criteria=THC2404993) | 0.52 | <0.0001 |
| A_23_P147869 | [KIAA1109](http://genome-www4.stanford.edu/cgi-bin/SMD/source/sourceResult?choice=Gene&option=Name&criteria=KIAA1109) | 0.60 | <0.0001 |
| A_24_P787914 | [U25029](http://genome-www4.stanford.edu/cgi-bin/SMD/source/sourceResult?choice=Gene&option=Name&criteria=U25029) | 0.56 | <0.0001 |
| A_23_P207201 | [CD79B](http://genome-www4.stanford.edu/cgi-bin/SMD/source/sourceResult?choice=Gene&option=Name&criteria=CD79B) | 0.50 | <0.0001 |
| A_23_P56578 | [VIT](http://genome-www4.stanford.edu/cgi-bin/SMD/source/sourceResult?choice=Gene&option=Name&criteria=VIT) | 0.51 | <0.0001 |
| A_32_P42149 | [A_32_P42149](http://genome-www4.stanford.edu/cgi-bin/SMD/source/sourceResult?choice=Gene&option=Name&criteria=A_32_P42149) | 0.61 | <0.0001 |
| A_32_P80587 | [A_32_P80587](http://genome-www4.stanford.edu/cgi-bin/SMD/source/sourceResult?choice=Gene&option=Name&criteria=A_32_P80587) | 0.58 | <0.0001 |
| A_24_P932308 | [COQ9](http://genome-www4.stanford.edu/cgi-bin/SMD/source/sourceResult?choice=Gene&option=Name&criteria=COQ9) | 0.57 | <0.0001 |
| A_23_P371076 | [KLF12](http://genome-www4.stanford.edu/cgi-bin/SMD/source/sourceResult?choice=Gene&option=Name&criteria=KLF12) | 0.52 | <0.0001 |
| A_24_P592060 | [THC2269397](http://genome-www4.stanford.edu/cgi-bin/SMD/source/sourceResult?choice=Gene&option=Name&criteria=THC2269397) | 0.61 | <0.0001 |
| A_23_P124335 | [LOC642299](http://genome-www4.stanford.edu/cgi-bin/SMD/source/sourceResult?choice=Gene&option=Name&criteria=LOC642299) | 0.64 | <0.0001 |
| A_32_P186921 | [ZNF616](http://genome-www4.stanford.edu/cgi-bin/SMD/source/sourceResult?choice=Gene&option=Name&criteria=ZNF616) | 0.66 | <0.0001 |
| A_23_P339633 | [CCDC75](http://genome-www4.stanford.edu/cgi-bin/SMD/source/sourceResult?choice=Gene&option=Name&criteria=CCDC75) | 0.58 | <0.0001 |
| A_24_P928272 | [AF086376](http://genome-www4.stanford.edu/cgi-bin/SMD/source/sourceResult?choice=Gene&option=Name&criteria=AF086376) | 0.63 | <0.0001 |
| A_24_P212457 | [GON4L](http://genome-www4.stanford.edu/cgi-bin/SMD/source/sourceResult?choice=Gene&option=Name&criteria=GON4L) | 0.61 | <0.0001 |
| A_23_P204810 | [OSBPL8](http://genome-www4.stanford.edu/cgi-bin/SMD/source/sourceResult?choice=Gene&option=Name&criteria=OSBPL8) | 0.62 | <0.0001 |
| A_23_P382835 | [P2RY1](http://genome-www4.stanford.edu/cgi-bin/SMD/source/sourceResult?choice=Gene&option=Name&criteria=P2RY1) | 0.57 | <0.0001 |
| A_24_P118171 | [ENST00000369615](http://genome-www4.stanford.edu/cgi-bin/SMD/source/sourceResult?choice=Gene&option=Name&criteria=ENST00000369615) | 0.62 | <0.0001 |
| A_24_P927222 | [ENST00000265271](http://genome-www4.stanford.edu/cgi-bin/SMD/source/sourceResult?choice=Gene&option=Name&criteria=ENST00000265271) | 0.64 | <0.0001 |
| A_32_P222060 | [THC2345721](http://genome-www4.stanford.edu/cgi-bin/SMD/source/sourceResult?choice=Gene&option=Name&criteria=THC2345721) | 0.61 | <0.0001 |
| A_24_P934704 | [A_24_P934704](http://genome-www4.stanford.edu/cgi-bin/SMD/source/sourceResult?choice=Gene&option=Name&criteria=A_24_P934704) | 0.52 | <0.0001 |
| A_23_P351286 | [FLJ22814](http://genome-www4.stanford.edu/cgi-bin/SMD/source/sourceResult?choice=Gene&option=Name&criteria=FLJ22814) | 0.53 | <0.0001 |
| A_23_P16806 | [MGC10701](http://genome-www4.stanford.edu/cgi-bin/SMD/source/sourceResult?choice=Gene&option=Name&criteria=MGC10701) | 0.61 | <0.0001 |
| A_32_P4581 | [AK130118](http://genome-www4.stanford.edu/cgi-bin/SMD/source/sourceResult?choice=Gene&option=Name&criteria=AK130118) | 0.65 | <0.0001 |
| A_32_P221641 | [A_32_P221641](http://genome-www4.stanford.edu/cgi-bin/SMD/source/sourceResult?choice=Gene&option=Name&criteria=A_32_P221641) | 0.56 | <0.0001 |
| A_23_P37111 | [DICER1](http://genome-www4.stanford.edu/cgi-bin/SMD/source/sourceResult?choice=Gene&option=Name&criteria=DICER1) | 0.67 | <0.0001 |
| A_32_P137604 | [BC018597](http://genome-www4.stanford.edu/cgi-bin/SMD/source/sourceResult?choice=Gene&option=Name&criteria=BC018597) | 0.52 | <0.0001 |
| A_32_P64016 | [LOC440352](http://genome-www4.stanford.edu/cgi-bin/SMD/source/sourceResult?choice=Gene&option=Name&criteria=LOC440352) | 0.48 | <0.0001 |
| A_32_P106315 | [SPATA5](http://genome-www4.stanford.edu/cgi-bin/SMD/source/sourceResult?choice=Gene&option=Name&criteria=SPATA5) | 0.61 | <0.0001 |
| A_23_P85269 | [TTN](http://genome-www4.stanford.edu/cgi-bin/SMD/source/sourceResult?choice=Gene&option=Name&criteria=TTN) | 0.57 | <0.0001 |
| A_23_P352266 | [BCL2](http://genome-www4.stanford.edu/cgi-bin/SMD/source/sourceResult?choice=Gene&option=Name&criteria=BCL2) | 0.65 | <0.0001 |
| A_24_P301954 | [MGC16384](http://genome-www4.stanford.edu/cgi-bin/SMD/source/sourceResult?choice=Gene&option=Name&criteria=MGC16384) | 0.65 | <0.0001 |
| A_23_P96369 | [CXorf57](http://genome-www4.stanford.edu/cgi-bin/SMD/source/sourceResult?choice=Gene&option=Name&criteria=CXorf57) | 0.55 | <0.0001 |
| A_23_P52846 | [THC2447689](http://genome-www4.stanford.edu/cgi-bin/SMD/source/sourceResult?choice=Gene&option=Name&criteria=THC2447689) | 0.58 | <0.0001 |
| A_32_P211141 | [LOC90624](http://genome-www4.stanford.edu/cgi-bin/SMD/source/sourceResult?choice=Gene&option=Name&criteria=LOC90624) | 0.64 | <0.0001 |
| A_23_P39137 | [GRLF1](http://genome-www4.stanford.edu/cgi-bin/SMD/source/sourceResult?choice=Gene&option=Name&criteria=GRLF1) | 0.58 | <0.0001 |
| A_32_P144007 | [THC2381800](http://genome-www4.stanford.edu/cgi-bin/SMD/source/sourceResult?choice=Gene&option=Name&criteria=THC2381800) | 0.63 | <0.0001 |
| A_24_P328504 | [SP140](http://genome-www4.stanford.edu/cgi-bin/SMD/source/sourceResult?choice=Gene&option=Name&criteria=SP140) | 0.56 | <0.0001 |
| A_23_P391344 | [RASGEF1A](http://genome-www4.stanford.edu/cgi-bin/SMD/source/sourceResult?choice=Gene&option=Name&criteria=RASGEF1A) | 0.52 | <0.0001 |
| A_23_P90542 | [ZNF540](http://genome-www4.stanford.edu/cgi-bin/SMD/source/sourceResult?choice=Gene&option=Name&criteria=ZNF540) | 0.55 | <0.0001 |
| A_32_P101799 | [THC2282944](http://genome-www4.stanford.edu/cgi-bin/SMD/source/sourceResult?choice=Gene&option=Name&criteria=THC2282944) | 0.61 | <0.0001 |
| A_32_P87631 | [BC017507](http://genome-www4.stanford.edu/cgi-bin/SMD/source/sourceResult?choice=Gene&option=Name&criteria=BC017507) | 0.65 | <0.0001 |
| A_23_P66948 | [FAM59A](http://genome-www4.stanford.edu/cgi-bin/SMD/source/sourceResult?choice=Gene&option=Name&criteria=FAM59A) | 0.66 | <0.0001 |
| A_32_P48526 | [THC2341087](http://genome-www4.stanford.edu/cgi-bin/SMD/source/sourceResult?choice=Gene&option=Name&criteria=THC2341087) | 0.59 | <0.0001 |
| A_32_P41153 | [BM929882](http://genome-www4.stanford.edu/cgi-bin/SMD/source/sourceResult?choice=Gene&option=Name&criteria=BM929882) | 0.60 | <0.0001 |
| A_32_P194891 | [THC2282954](http://genome-www4.stanford.edu/cgi-bin/SMD/source/sourceResult?choice=Gene&option=Name&criteria=THC2282954) | 0.54 | <0.0001 |
| A_32_P7308 | [THC2407640](http://genome-www4.stanford.edu/cgi-bin/SMD/source/sourceResult?choice=Gene&option=Name&criteria=THC2407640) | 0.61 | <0.0001 |
| A_23_P343398 | [CCR7](http://genome-www4.stanford.edu/cgi-bin/SMD/source/sourceResult?choice=Gene&option=Name&criteria=CCR7) | 0.43 | <0.0001 |
| A_24_P136471 | [SLC14A2](http://genome-www4.stanford.edu/cgi-bin/SMD/source/sourceResult?choice=Gene&option=Name&criteria=SLC14A2) | 0.48 | <0.0001 |
| A_32_P8813 | [LOC283663](http://genome-www4.stanford.edu/cgi-bin/SMD/source/sourceResult?choice=Gene&option=Name&criteria=LOC283663) | 0.58 | <0.0001 |
| A_23_P394562 | [ENST00000339363](http://genome-www4.stanford.edu/cgi-bin/SMD/source/sourceResult?choice=Gene&option=Name&criteria=ENST00000339363) | 0.57 | <0.0001 |
| A_32_P71858 | [AK124776](http://genome-www4.stanford.edu/cgi-bin/SMD/source/sourceResult?choice=Gene&option=Name&criteria=AK124776) | 0.62 | <0.0001 |
| A_32_P98059 | [AK126329](http://genome-www4.stanford.edu/cgi-bin/SMD/source/sourceResult?choice=Gene&option=Name&criteria=AK126329) | 0.66 | <0.0001 |
| A_32_P152986 | [THC2313300](http://genome-www4.stanford.edu/cgi-bin/SMD/source/sourceResult?choice=Gene&option=Name&criteria=THC2313300) | 0.66 | <0.0001 |
| A_23_P11017 | [AK021866](http://genome-www4.stanford.edu/cgi-bin/SMD/source/sourceResult?choice=Gene&option=Name&criteria=AK021866) | 0.63 | <0.0001 |
| A_23_P204689 | [CLEC2D](http://genome-www4.stanford.edu/cgi-bin/SMD/source/sourceResult?choice=Gene&option=Name&criteria=CLEC2D) | 0.60 | <0.0001 |
| A_23_P39067 | [SPIB](http://genome-www4.stanford.edu/cgi-bin/SMD/source/sourceResult?choice=Gene&option=Name&criteria=SPIB) | 0.63 | <0.0001 |
| A_23_P431252 | [KBTBD8](http://genome-www4.stanford.edu/cgi-bin/SMD/source/sourceResult?choice=Gene&option=Name&criteria=KBTBD8) | 0.61 | <0.0001 |
| A_23_P212500 | [TF](http://genome-www4.stanford.edu/cgi-bin/SMD/source/sourceResult?choice=Gene&option=Name&criteria=TF) | 0.65 | <0.0001 |
| A_32_P44394 | [AIM2](http://genome-www4.stanford.edu/cgi-bin/SMD/source/sourceResult?choice=Gene&option=Name&criteria=AIM2) | 0.44 | <0.0001 |
| A_32_P107777 | [THC2339241](http://genome-www4.stanford.edu/cgi-bin/SMD/source/sourceResult?choice=Gene&option=Name&criteria=THC2339241) | 0.66 | <0.0001 |
| A_23_P433785 | [P2RX5](http://genome-www4.stanford.edu/cgi-bin/SMD/source/sourceResult?choice=Gene&option=Name&criteria=P2RX5) | 0.57 | <0.0001 |
| A_24_P153576 | [SHPRH](http://genome-www4.stanford.edu/cgi-bin/SMD/source/sourceResult?choice=Gene&option=Name&criteria=SHPRH) | 0.61 | <0.0001 |
| A_32_P166480 | [C9orf93](http://genome-www4.stanford.edu/cgi-bin/SMD/source/sourceResult?choice=Gene&option=Name&criteria=C9orf93) | 0.60 | <0.0001 |
| A_23_P209712 | [SP100](http://genome-www4.stanford.edu/cgi-bin/SMD/source/sourceResult?choice=Gene&option=Name&criteria=SP100) | 0.63 | <0.0001 |
| A_32_P215462 | [BQ272125](http://genome-www4.stanford.edu/cgi-bin/SMD/source/sourceResult?choice=Gene&option=Name&criteria=BQ272125) | 0.62 | <0.0001 |
| A_23_P155197 | [PTPLB](http://genome-www4.stanford.edu/cgi-bin/SMD/source/sourceResult?choice=Gene&option=Name&criteria=PTPLB) | 0.57 | <0.0001 |
| A_32_P181826 | [THC2342112](http://genome-www4.stanford.edu/cgi-bin/SMD/source/sourceResult?choice=Gene&option=Name&criteria=THC2342112) | 0.65 | <0.0001 |
| A_32_P224638 | [THC2407541](http://genome-www4.stanford.edu/cgi-bin/SMD/source/sourceResult?choice=Gene&option=Name&criteria=THC2407541) | 0.63 | <0.0001 |
| A_24_P395415 | [AF343666](http://genome-www4.stanford.edu/cgi-bin/SMD/source/sourceResult?choice=Gene&option=Name&criteria=AF343666) | 0.51 | <0.0001 |
| A_32_P195346 | [THC2443199](http://genome-www4.stanford.edu/cgi-bin/SMD/source/sourceResult?choice=Gene&option=Name&criteria=THC2443199) | 0.63 | <0.0001 |
| A_23_P159316 | [BFSP2](http://genome-www4.stanford.edu/cgi-bin/SMD/source/sourceResult?choice=Gene&option=Name&criteria=BFSP2) | 0.47 | <0.0001 |
| A_32_P220580 | [AK124352](http://genome-www4.stanford.edu/cgi-bin/SMD/source/sourceResult?choice=Gene&option=Name&criteria=AK124352) | 0.59 | <0.0001 |
| A_24_P747419 | [THC2376828](http://genome-www4.stanford.edu/cgi-bin/SMD/source/sourceResult?choice=Gene&option=Name&criteria=THC2376828) | 0.57 | <0.0001 |
| A_24_P307195 | [AASDH](http://genome-www4.stanford.edu/cgi-bin/SMD/source/sourceResult?choice=Gene&option=Name&criteria=AASDH) | 0.67 | <0.0001 |
| A_23_P89871 | [ZNF415](http://genome-www4.stanford.edu/cgi-bin/SMD/source/sourceResult?choice=Gene&option=Name&criteria=ZNF415) | 0.64 | <0.0001 |
| A_32_P6972 | [THC2341837](http://genome-www4.stanford.edu/cgi-bin/SMD/source/sourceResult?choice=Gene&option=Name&criteria=THC2341837) | 0.64 | <0.0001 |
| A_23_P39294 | [ENST00000312785](http://genome-www4.stanford.edu/cgi-bin/SMD/source/sourceResult?choice=Gene&option=Name&criteria=ENST00000312785) | 0.57 | <0.0001 |
| A_23_P500998 | [HOXA9](http://genome-www4.stanford.edu/cgi-bin/SMD/source/sourceResult?choice=Gene&option=Name&criteria=HOXA9) | 0.36 | <0.0001 |
| A_23_P135123 | [BG216229](http://genome-www4.stanford.edu/cgi-bin/SMD/source/sourceResult?choice=Gene&option=Name&criteria=BG216229) | 0.63 | <0.0001 |
| A_32_P84242 | [KIAA0888](http://genome-www4.stanford.edu/cgi-bin/SMD/source/sourceResult?choice=Gene&option=Name&criteria=KIAA0888) | 0.56 | <0.0001 |
| A_23_P212869 | [GABRA2](http://genome-www4.stanford.edu/cgi-bin/SMD/source/sourceResult?choice=Gene&option=Name&criteria=GABRA2) | 0.55 | <0.0001 |
| A_32_P132766 | [THC2406017](http://genome-www4.stanford.edu/cgi-bin/SMD/source/sourceResult?choice=Gene&option=Name&criteria=THC2406017) | 0.65 | <0.0001 |
| A_24_P346101 | [MGC21644](http://genome-www4.stanford.edu/cgi-bin/SMD/source/sourceResult?choice=Gene&option=Name&criteria=MGC21644) | 0.61 | <0.0001 |
| A_32_P46510 | [ZC3H6](http://genome-www4.stanford.edu/cgi-bin/SMD/source/sourceResult?choice=Gene&option=Name&criteria=ZC3H6) | 0.66 | <0.0001 |
| A_23_P347070 | [PAG1](http://genome-www4.stanford.edu/cgi-bin/SMD/source/sourceResult?choice=Gene&option=Name&criteria=PAG1) | 0.63 | <0.0001 |
| A_32_P49764 | [CX788817](http://genome-www4.stanford.edu/cgi-bin/SMD/source/sourceResult?choice=Gene&option=Name&criteria=CX788817) | 0.50 | <0.0001 |
| A_32_P111394 | [THC2441040](http://genome-www4.stanford.edu/cgi-bin/SMD/source/sourceResult?choice=Gene&option=Name&criteria=THC2441040) | 0.61 | <0.0001 |
| A_24_P935682 | [AY358248](http://genome-www4.stanford.edu/cgi-bin/SMD/source/sourceResult?choice=Gene&option=Name&criteria=AY358248) | 0.62 | <0.0001 |
| A_23_P321984 | [DCAL1](http://genome-www4.stanford.edu/cgi-bin/SMD/source/sourceResult?choice=Gene&option=Name&criteria=DCAL1) | 0.53 | <0.0001 |
| A_32_P128974 | [THC2280383](http://genome-www4.stanford.edu/cgi-bin/SMD/source/sourceResult?choice=Gene&option=Name&criteria=THC2280383) | 0.66 | <0.0001 |
| A_23_P375566 | [STXBP4](http://genome-www4.stanford.edu/cgi-bin/SMD/source/sourceResult?choice=Gene&option=Name&criteria=STXBP4) | 0.64 | <0.0001 |
| A_23_P428468 | [ENST00000369577](http://genome-www4.stanford.edu/cgi-bin/SMD/source/sourceResult?choice=Gene&option=Name&criteria=ENST00000369577) | 0.64 | <0.0001 |
| A_23_P127789 | [AHNAK](http://genome-www4.stanford.edu/cgi-bin/SMD/source/sourceResult?choice=Gene&option=Name&criteria=AHNAK) | 0.61 | <0.0001 |
| A_32_P190682 | [THC2347318](http://genome-www4.stanford.edu/cgi-bin/SMD/source/sourceResult?choice=Gene&option=Name&criteria=THC2347318) | 0.63 | <0.0001 |
| A_23_P212179 | [HRH1](http://genome-www4.stanford.edu/cgi-bin/SMD/source/sourceResult?choice=Gene&option=Name&criteria=HRH1) | 0.62 | <0.0001 |
| A_24_P418637 | [MACF1](http://genome-www4.stanford.edu/cgi-bin/SMD/source/sourceResult?choice=Gene&option=Name&criteria=MACF1) | 0.58 | <0.0001 |
| A_24_P298224 | [FLJ34870](http://genome-www4.stanford.edu/cgi-bin/SMD/source/sourceResult?choice=Gene&option=Name&criteria=FLJ34870) | 0.62 | <0.0001 |
| A_32_P58912 | [A_32_P58912](http://genome-www4.stanford.edu/cgi-bin/SMD/source/sourceResult?choice=Gene&option=Name&criteria=A_32_P58912) | 0.63 | 0.0012 |
| A_23_P146783 | [NEB](http://genome-www4.stanford.edu/cgi-bin/SMD/source/sourceResult?choice=Gene&option=Name&criteria=NEB) | 0.52 | 0.0012 |
| A_24_P136807 | [RFC1](http://genome-www4.stanford.edu/cgi-bin/SMD/source/sourceResult?choice=Gene&option=Name&criteria=RFC1) | 0.63 | 0.0012 |
| A_24_P248251 | [SMARCA3](http://genome-www4.stanford.edu/cgi-bin/SMD/source/sourceResult?choice=Gene&option=Name&criteria=SMARCA3) | 0.60 | 0.0012 |
| A_23_P9402 | [CNTFR](http://genome-www4.stanford.edu/cgi-bin/SMD/source/sourceResult?choice=Gene&option=Name&criteria=CNTFR) | 0.60 | 0.0012 |
| A_32_P19561 | [THC2407737](http://genome-www4.stanford.edu/cgi-bin/SMD/source/sourceResult?choice=Gene&option=Name&criteria=THC2407737) | 0.54 | 0.0012 |
| A_23_P154379 | [NAT8](http://genome-www4.stanford.edu/cgi-bin/SMD/source/sourceResult?choice=Gene&option=Name&criteria=NAT8) | 0.44 | 0.0012 |
| A_23_P300301 | [JMJD1C](http://genome-www4.stanford.edu/cgi-bin/SMD/source/sourceResult?choice=Gene&option=Name&criteria=JMJD1C) | 0.58 | 0.0012 |
| A_23_P26358 | [SMG1](http://genome-www4.stanford.edu/cgi-bin/SMD/source/sourceResult?choice=Gene&option=Name&criteria=SMG1) | 0.57 | 0.0012 |
| A_24_P238402 | [ZNF644](http://genome-www4.stanford.edu/cgi-bin/SMD/source/sourceResult?choice=Gene&option=Name&criteria=ZNF644) | 0.63 | 0.0012 |
| A_32_P220700 | [THC2440162](http://genome-www4.stanford.edu/cgi-bin/SMD/source/sourceResult?choice=Gene&option=Name&criteria=THC2440162) | 0.65 | 0.0012 |
| A_23_P156218 | [GZMK](http://genome-www4.stanford.edu/cgi-bin/SMD/source/sourceResult?choice=Gene&option=Name&criteria=GZMK) | 0.60 | 0.0012 |
| A_32_P143000 | [ENST00000261275](http://genome-www4.stanford.edu/cgi-bin/SMD/source/sourceResult?choice=Gene&option=Name&criteria=ENST00000261275) | 0.65 | 0.0012 |
| A_32_P43878 | [THC2379275](http://genome-www4.stanford.edu/cgi-bin/SMD/source/sourceResult?choice=Gene&option=Name&criteria=THC2379275) | 0.56 | 0.0012 |
| A_24_P799680 | [A_24_P799680](http://genome-www4.stanford.edu/cgi-bin/SMD/source/sourceResult?choice=Gene&option=Name&criteria=A_24_P799680) | 0.66 | 0.0012 |
| A_24_P67980 | [AK054946](http://genome-www4.stanford.edu/cgi-bin/SMD/source/sourceResult?choice=Gene&option=Name&criteria=AK054946) | 0.55 | 0.0012 |
| A_24_P576219 | [BC035091](http://genome-www4.stanford.edu/cgi-bin/SMD/source/sourceResult?choice=Gene&option=Name&criteria=BC035091) | 0.64 | 0.0012 |
| A_32_P132276 | [BE091362](http://genome-www4.stanford.edu/cgi-bin/SMD/source/sourceResult?choice=Gene&option=Name&criteria=BE091362) | 0.63 | 0.0012 |
| A_32_P167883 | [THC2344420](http://genome-www4.stanford.edu/cgi-bin/SMD/source/sourceResult?choice=Gene&option=Name&criteria=THC2344420) | 0.65 | 0.0012 |
| A_24_P813730 | [THC2378508](http://genome-www4.stanford.edu/cgi-bin/SMD/source/sourceResult?choice=Gene&option=Name&criteria=THC2378508) | 0.66 | 0.0012 |
| A_24_P715530 | [AK025082](http://genome-www4.stanford.edu/cgi-bin/SMD/source/sourceResult?choice=Gene&option=Name&criteria=AK025082) | 0.65 | 0.0012 |
| A_24_P941359 | [C6orf32](http://genome-www4.stanford.edu/cgi-bin/SMD/source/sourceResult?choice=Gene&option=Name&criteria=C6orf32) | 0.48 | 0.0012 |
| A_23_P26713 | [RPL23](http://genome-www4.stanford.edu/cgi-bin/SMD/source/sourceResult?choice=Gene&option=Name&criteria=RPL23) | 0.60 | 0.0012 |
| A_32_P225301 | [THC2280343](http://genome-www4.stanford.edu/cgi-bin/SMD/source/sourceResult?choice=Gene&option=Name&criteria=THC2280343) | 0.61 | 0.0012 |
| A_32_P9931 | [A_32_P9931](http://genome-www4.stanford.edu/cgi-bin/SMD/source/sourceResult?choice=Gene&option=Name&criteria=A_32_P9931) | 0.63 | 0.0012 |
| A_32_P139260 | [THC2410817](http://genome-www4.stanford.edu/cgi-bin/SMD/source/sourceResult?choice=Gene&option=Name&criteria=THC2410817) | 0.66 | 0.0012 |
| A_23_P383698 | [LOC440345](http://genome-www4.stanford.edu/cgi-bin/SMD/source/sourceResult?choice=Gene&option=Name&criteria=LOC440345) | 0.57 | 0.0012 |
| A_32_P113472 | [CA314451](http://genome-www4.stanford.edu/cgi-bin/SMD/source/sourceResult?choice=Gene&option=Name&criteria=CA314451) | 0.61 | 0.0012 |
| A_23_P131825 | [TNNC2](http://genome-www4.stanford.edu/cgi-bin/SMD/source/sourceResult?choice=Gene&option=Name&criteria=TNNC2) | 0.61 | 0.0012 |
| A_32_P191735 | [CK820941](http://genome-www4.stanford.edu/cgi-bin/SMD/source/sourceResult?choice=Gene&option=Name&criteria=CK820941) | 0.60 | 0.0012 |
| A_23_P351320 | [CDC40](http://genome-www4.stanford.edu/cgi-bin/SMD/source/sourceResult?choice=Gene&option=Name&criteria=CDC40) | 0.64 | 0.0012 |
| A_24_P375592 | [PHF20](http://genome-www4.stanford.edu/cgi-bin/SMD/source/sourceResult?choice=Gene&option=Name&criteria=PHF20) | 0.65 | 0.0012 |
| A_32_P87074 | [AK022150](http://genome-www4.stanford.edu/cgi-bin/SMD/source/sourceResult?choice=Gene&option=Name&criteria=AK022150) | 0.61 | 0.0012 |
| A_32_P169222 | [THC2328913](http://genome-www4.stanford.edu/cgi-bin/SMD/source/sourceResult?choice=Gene&option=Name&criteria=THC2328913) | 0.67 | 0.0012 |
| A_32_P156136 | [NARG1L](http://genome-www4.stanford.edu/cgi-bin/SMD/source/sourceResult?choice=Gene&option=Name&criteria=NARG1L) | 0.62 | 0.0012 |
| A_32_P95541 | [AA564209](http://genome-www4.stanford.edu/cgi-bin/SMD/source/sourceResult?choice=Gene&option=Name&criteria=AA564209) | 0.65 | 0.0012 |
| A_24_P617818 | [THC2382362](http://genome-www4.stanford.edu/cgi-bin/SMD/source/sourceResult?choice=Gene&option=Name&criteria=THC2382362) | 0.64 | 0.0012 |
| A_23_P125748 | [ZMAT1](http://genome-www4.stanford.edu/cgi-bin/SMD/source/sourceResult?choice=Gene&option=Name&criteria=ZMAT1) | 0.60 | 0.0012 |
| A_24_P924589 | [THC2354953](http://genome-www4.stanford.edu/cgi-bin/SMD/source/sourceResult?choice=Gene&option=Name&criteria=THC2354953) | 0.66 | 0.0012 |
| A_23_P218784 | [DDX17](http://genome-www4.stanford.edu/cgi-bin/SMD/source/sourceResult?choice=Gene&option=Name&criteria=DDX17) | 0.65 | 0.0012 |
| A_23_P133216 | [NLN](http://genome-www4.stanford.edu/cgi-bin/SMD/source/sourceResult?choice=Gene&option=Name&criteria=NLN) | 0.63 | 0.0012 |
| A_32_P126362 | [THC2324430](http://genome-www4.stanford.edu/cgi-bin/SMD/source/sourceResult?choice=Gene&option=Name&criteria=THC2324430) | 0.66 | 0.0012 |
| A_23_P155688 | [SPINK2](http://genome-www4.stanford.edu/cgi-bin/SMD/source/sourceResult?choice=Gene&option=Name&criteria=SPINK2) | 0.62 | 0.0012 |
| A_32_P89087 | [AL134462](http://genome-www4.stanford.edu/cgi-bin/SMD/source/sourceResult?choice=Gene&option=Name&criteria=AL134462) | 0.66 | 0.0012 |
| A_23_P51082 | [THC2242915](http://genome-www4.stanford.edu/cgi-bin/SMD/source/sourceResult?choice=Gene&option=Name&criteria=THC2242915) | 0.64 | 0.0012 |
| A_32_P162095 | [THC2381061](http://genome-www4.stanford.edu/cgi-bin/SMD/source/sourceResult?choice=Gene&option=Name&criteria=THC2381061) | 0.62 | 0.0012 |
| A_23_P70968 | [HOXA7](http://genome-www4.stanford.edu/cgi-bin/SMD/source/sourceResult?choice=Gene&option=Name&criteria=HOXA7) | 0.53 | 0.0012 |
| A_32_P227657 | [THC2378933](http://genome-www4.stanford.edu/cgi-bin/SMD/source/sourceResult?choice=Gene&option=Name&criteria=THC2378933) | 0.62 | 0.0012 |
| A_24_P136711 | [BC030757](http://genome-www4.stanford.edu/cgi-bin/SMD/source/sourceResult?choice=Gene&option=Name&criteria=BC030757) | 0.64 | 0.0012 |
| A_32_P124392 | [A_32_P124392](http://genome-www4.stanford.edu/cgi-bin/SMD/source/sourceResult?choice=Gene&option=Name&criteria=A_32_P124392) | 0.66 | 0.0012 |
| A_32_P134290 | [ZCCHC2](http://genome-www4.stanford.edu/cgi-bin/SMD/source/sourceResult?choice=Gene&option=Name&criteria=ZCCHC2) | 0.63 | 0.0012 |
| A_24_P254106 | [FLJ22814](http://genome-www4.stanford.edu/cgi-bin/SMD/source/sourceResult?choice=Gene&option=Name&criteria=FLJ22814) | 0.45 | 0.0012 |
| A_23_P25587 | [LECT1](http://genome-www4.stanford.edu/cgi-bin/SMD/source/sourceResult?choice=Gene&option=Name&criteria=LECT1) | 0.67 | 0.0012 |
| A_32_P13991 | [THC2405366](http://genome-www4.stanford.edu/cgi-bin/SMD/source/sourceResult?choice=Gene&option=Name&criteria=THC2405366) | 0.64 | 0.0012 |
| A_24_P98948 | [BU943730](http://genome-www4.stanford.edu/cgi-bin/SMD/source/sourceResult?choice=Gene&option=Name&criteria=BU943730) | 0.53 | 0.0012 |
| A_24_P538708 | [AK124263](http://genome-www4.stanford.edu/cgi-bin/SMD/source/sourceResult?choice=Gene&option=Name&criteria=AK124263) | 0.63 | 0.0012 |
| A_23_P390722 | [IFRG15](http://genome-www4.stanford.edu/cgi-bin/SMD/source/sourceResult?choice=Gene&option=Name&criteria=IFRG15) | 0.66 | 0.0012 |
| A_24_P940079 | [A_24_P940079](http://genome-www4.stanford.edu/cgi-bin/SMD/source/sourceResult?choice=Gene&option=Name&criteria=A_24_P940079) | 0.65 | 0.0012 |
| A_32_P170406 | [TLN2](http://genome-www4.stanford.edu/cgi-bin/SMD/source/sourceResult?choice=Gene&option=Name&criteria=TLN2) | 0.59 | 0.0012 |
| A_32_P115717 | [LOC643277](http://genome-www4.stanford.edu/cgi-bin/SMD/source/sourceResult?choice=Gene&option=Name&criteria=LOC643277) | 0.64 | 0.0012 |
| A_32_P1144 | [AK091357](http://genome-www4.stanford.edu/cgi-bin/SMD/source/sourceResult?choice=Gene&option=Name&criteria=AK091357) | 0.65 | 0.0012 |
| A_32_P72822 | [CCNB2](http://genome-www4.stanford.edu/cgi-bin/SMD/source/sourceResult?choice=Gene&option=Name&criteria=CCNB2) | 0.66 | 0.0020 |
| A_24_P25252 | [ANKRD12](http://genome-www4.stanford.edu/cgi-bin/SMD/source/sourceResult?choice=Gene&option=Name&criteria=ANKRD12) | 0.66 | 0.0020 |
| A_32_P75399 | [THC2308675](http://genome-www4.stanford.edu/cgi-bin/SMD/source/sourceResult?choice=Gene&option=Name&criteria=THC2308675) | 0.63 | 0.0020 |
| A_23_P136753 | [THC2275252](http://genome-www4.stanford.edu/cgi-bin/SMD/source/sourceResult?choice=Gene&option=Name&criteria=THC2275252) | 0.57 | 0.0020 |
| A_24_P67096 | [ABCA5](http://genome-www4.stanford.edu/cgi-bin/SMD/source/sourceResult?choice=Gene&option=Name&criteria=ABCA5) | 0.66 | 0.0020 |
| A_32_P117730 | [THC2283727](http://genome-www4.stanford.edu/cgi-bin/SMD/source/sourceResult?choice=Gene&option=Name&criteria=THC2283727) | 0.62 | 0.0020 |
| A_23_P45087 | [ZNF588](http://genome-www4.stanford.edu/cgi-bin/SMD/source/sourceResult?choice=Gene&option=Name&criteria=ZNF588) | 0.58 | 0.0020 |
| A_32_P40424 | [AA630774](http://genome-www4.stanford.edu/cgi-bin/SMD/source/sourceResult?choice=Gene&option=Name&criteria=AA630774) | 0.65 | 0.0020 |
| A_32_P313405 | [LAMA1](http://genome-www4.stanford.edu/cgi-bin/SMD/source/sourceResult?choice=Gene&option=Name&criteria=LAMA1) | 0.59 | 0.0020 |
| A_24_P593120 | [BF949582](http://genome-www4.stanford.edu/cgi-bin/SMD/source/sourceResult?choice=Gene&option=Name&criteria=BF949582) | 0.60 | 0.0020 |
| A_23_P115726 | [SLC16A9](http://genome-www4.stanford.edu/cgi-bin/SMD/source/sourceResult?choice=Gene&option=Name&criteria=SLC16A9) | 0.49 | 0.0020 |
| A_32_P220523 | [THC2439328](http://genome-www4.stanford.edu/cgi-bin/SMD/source/sourceResult?choice=Gene&option=Name&criteria=THC2439328) | 0.66 | 0.0020 |
| A_23_P404211 | [FANCM](http://genome-www4.stanford.edu/cgi-bin/SMD/source/sourceResult?choice=Gene&option=Name&criteria=FANCM) | 0.62 | 0.0020 |
| A_32_P110243 | [BC071734](http://genome-www4.stanford.edu/cgi-bin/SMD/source/sourceResult?choice=Gene&option=Name&criteria=BC071734) | 0.66 | 0.0020 |
| A_24_P399220 | [HOXB3](http://genome-www4.stanford.edu/cgi-bin/SMD/source/sourceResult?choice=Gene&option=Name&criteria=HOXB3) | 0.65 | 0.0020 |
| A_23_P328621 | [MGC20470](http://genome-www4.stanford.edu/cgi-bin/SMD/source/sourceResult?choice=Gene&option=Name&criteria=MGC20470) | 0.64 | 0.0020 |
| A_32_P110820 | [THC2312274](http://genome-www4.stanford.edu/cgi-bin/SMD/source/sourceResult?choice=Gene&option=Name&criteria=THC2312274) | 0.67 | 0.0020 |
| A_32_P61298 | [CDR1](http://genome-www4.stanford.edu/cgi-bin/SMD/source/sourceResult?choice=Gene&option=Name&criteria=CDR1) | 0.63 | 0.0020 |
| A_24_P944331 | [PAQR5](http://genome-www4.stanford.edu/cgi-bin/SMD/source/sourceResult?choice=Gene&option=Name&criteria=PAQR5) | 0.49 | 0.0020 |
| A_24_P76158 | [DOCK11](http://genome-www4.stanford.edu/cgi-bin/SMD/source/sourceResult?choice=Gene&option=Name&criteria=DOCK11) | 0.67 | 0.0020 |
| A_24_P319647 | [FCRL2](http://genome-www4.stanford.edu/cgi-bin/SMD/source/sourceResult?choice=Gene&option=Name&criteria=FCRL2) | 0.65 | 0.0020 |
| A_24_P367666 | [ASH1L](http://genome-www4.stanford.edu/cgi-bin/SMD/source/sourceResult?choice=Gene&option=Name&criteria=ASH1L) | 0.62 | 0.0020 |
| A_32_P54616 | [KIAA1407](http://genome-www4.stanford.edu/cgi-bin/SMD/source/sourceResult?choice=Gene&option=Name&criteria=KIAA1407) | 0.66 | 0.0020 |
| A_24_P299007 | [AF187554](http://genome-www4.stanford.edu/cgi-bin/SMD/source/sourceResult?choice=Gene&option=Name&criteria=AF187554) | 0.64 | 0.0020 |
| A_24_P376139 | [AK057798](http://genome-www4.stanford.edu/cgi-bin/SMD/source/sourceResult?choice=Gene&option=Name&criteria=AK057798) | 0.66 | 0.0020 |
| A_32_P165297 | [ENST00000370857](http://genome-www4.stanford.edu/cgi-bin/SMD/source/sourceResult?choice=Gene&option=Name&criteria=ENST00000370857) | 0.64 | 0.0020 |
| A_32_P184394 | [TFEC](http://genome-www4.stanford.edu/cgi-bin/SMD/source/sourceResult?choice=Gene&option=Name&criteria=TFEC) | 0.46 | 0.0020 |
| A_32_P197561 | [AK123757](http://genome-www4.stanford.edu/cgi-bin/SMD/source/sourceResult?choice=Gene&option=Name&criteria=AK123757) | 0.56 | 0.0020 |
| A_24_P298495 | [AF289590](http://genome-www4.stanford.edu/cgi-bin/SMD/source/sourceResult?choice=Gene&option=Name&criteria=AF289590) | 0.62 | 0.0020 |
| A_24_P890536 | [CR627148](http://genome-www4.stanford.edu/cgi-bin/SMD/source/sourceResult?choice=Gene&option=Name&criteria=CR627148) | 0.60 | 0.0020 |
| A_23_P156076 | [AGXT2](http://genome-www4.stanford.edu/cgi-bin/SMD/source/sourceResult?choice=Gene&option=Name&criteria=AGXT2) | 0.49 | 0.0020 |
| A_23_P6822 | [ITIH3](http://genome-www4.stanford.edu/cgi-bin/SMD/source/sourceResult?choice=Gene&option=Name&criteria=ITIH3) | 0.65 | 0.0020 |
| A_23_P143935 | [PIGZ](http://genome-www4.stanford.edu/cgi-bin/SMD/source/sourceResult?choice=Gene&option=Name&criteria=PIGZ) | 0.50 | 0.0020 |
| A_23_P132956 | [UCHL1](http://genome-www4.stanford.edu/cgi-bin/SMD/source/sourceResult?choice=Gene&option=Name&criteria=UCHL1) | 0.61 | 0.0020 |
| A_32_P204565 | [A_32_P204565](http://genome-www4.stanford.edu/cgi-bin/SMD/source/sourceResult?choice=Gene&option=Name&criteria=A_32_P204565) | 0.64 | 0.0020 |
| A_23_P140009 | [SLC10A2](http://genome-www4.stanford.edu/cgi-bin/SMD/source/sourceResult?choice=Gene&option=Name&criteria=SLC10A2) | 0.47 | 0.0037 |
| A_32_P67533 | [L3MBTL3](http://genome-www4.stanford.edu/cgi-bin/SMD/source/sourceResult?choice=Gene&option=Name&criteria=L3MBTL3) | 0.66 | 0.0037 |
| A_23_P97173 | [HSD3B1](http://genome-www4.stanford.edu/cgi-bin/SMD/source/sourceResult?choice=Gene&option=Name&criteria=HSD3B1) | 0.65 | 0.0037 |
| A_32_P61684 | [PAG1](http://genome-www4.stanford.edu/cgi-bin/SMD/source/sourceResult?choice=Gene&option=Name&criteria=PAG1) | 0.65 | 0.0037 |
| A_32_P31827 | [THC2279918](http://genome-www4.stanford.edu/cgi-bin/SMD/source/sourceResult?choice=Gene&option=Name&criteria=THC2279918) | 0.66 | 0.0037 |
| A_24_P303052 | [PPARGC1A](http://genome-www4.stanford.edu/cgi-bin/SMD/source/sourceResult?choice=Gene&option=Name&criteria=PPARGC1A) | 0.63 | 0.0037 |
| A_32_P111266 | [THC2453189](http://genome-www4.stanford.edu/cgi-bin/SMD/source/sourceResult?choice=Gene&option=Name&criteria=THC2453189) | 0.65 | 0.0037 |
| A_23_P139500 | [BHLHB3](http://genome-www4.stanford.edu/cgi-bin/SMD/source/sourceResult?choice=Gene&option=Name&criteria=BHLHB3) | 0.60 | 0.0037 |
| A_24_P247910 | [BDP1](http://genome-www4.stanford.edu/cgi-bin/SMD/source/sourceResult?choice=Gene&option=Name&criteria=BDP1) | 0.62 | 0.0037 |
| A_24_P178106 | [YTHDC2](http://genome-www4.stanford.edu/cgi-bin/SMD/source/sourceResult?choice=Gene&option=Name&criteria=YTHDC2) | 0.66 | 0.0037 |
| A_32_P30345 | [THC2437906](http://genome-www4.stanford.edu/cgi-bin/SMD/source/sourceResult?choice=Gene&option=Name&criteria=THC2437906) | 0.63 | 0.0037 |
| A_32_P107876 | [FRAS1](http://genome-www4.stanford.edu/cgi-bin/SMD/source/sourceResult?choice=Gene&option=Name&criteria=FRAS1) | 0.59 | 0.0037 |
| A_32_P115446 | [THC2280176](http://genome-www4.stanford.edu/cgi-bin/SMD/source/sourceResult?choice=Gene&option=Name&criteria=THC2280176) | 0.66 | 0.0037 |
| A_23_P213166 | [LOC91431](http://genome-www4.stanford.edu/cgi-bin/SMD/source/sourceResult?choice=Gene&option=Name&criteria=LOC91431) | 0.66 | 0.0037 |
| A_24_P409681 | [A_24_P409681](http://genome-www4.stanford.edu/cgi-bin/SMD/source/sourceResult?choice=Gene&option=Name&criteria=A_24_P409681) | 0.65 | 0.0037 |
| A_23_P347632 | [MTSS1](http://genome-www4.stanford.edu/cgi-bin/SMD/source/sourceResult?choice=Gene&option=Name&criteria=MTSS1) | 0.63 | 0.0037 |
| A_32_P89646 | [A_32_P89646](http://genome-www4.stanford.edu/cgi-bin/SMD/source/sourceResult?choice=Gene&option=Name&criteria=A_32_P89646) | 0.65 | 0.0037 |
| A_32_P211048 | [A_32_P211048](http://genome-www4.stanford.edu/cgi-bin/SMD/source/sourceResult?choice=Gene&option=Name&criteria=A_32_P211048) | 0.64 | 0.0037 |
| A_23_P36865 | [CEP290](http://genome-www4.stanford.edu/cgi-bin/SMD/source/sourceResult?choice=Gene&option=Name&criteria=CEP290) | 0.64 | 0.0037 |
| A_24_P935852 | [A_24_P935852](http://genome-www4.stanford.edu/cgi-bin/SMD/source/sourceResult?choice=Gene&option=Name&criteria=A_24_P935852) | 0.51 | 0.0037 |
| A_23_P217187 | [A_23_P217187](http://genome-www4.stanford.edu/cgi-bin/SMD/source/sourceResult?choice=Gene&option=Name&criteria=A_23_P217187) | 0.59 | 0.0037 |
| A_32_P53976 | [A_32_P53976](http://genome-www4.stanford.edu/cgi-bin/SMD/source/sourceResult?choice=Gene&option=Name&criteria=A_32_P53976) | 0.62 | 0.0037 |
| A_32_P146635 | [CR603982](http://genome-www4.stanford.edu/cgi-bin/SMD/source/sourceResult?choice=Gene&option=Name&criteria=CR603982) | 0.66 | 0.0037 |
| A_32_P206479 | [ENST00000371030](http://genome-www4.stanford.edu/cgi-bin/SMD/source/sourceResult?choice=Gene&option=Name&criteria=ENST00000371030) | 0.63 | 0.0037 |
| A_24_P324141 | [ENST00000349637](http://genome-www4.stanford.edu/cgi-bin/SMD/source/sourceResult?choice=Gene&option=Name&criteria=ENST00000349637) | 0.65 | 0.0037 |
| A_23_P101407 | [C3](http://genome-www4.stanford.edu/cgi-bin/SMD/source/sourceResult?choice=Gene&option=Name&criteria=C3) | 0.54 | 0.0037 |
| A_32_P227921 | [THC2283605](http://genome-www4.stanford.edu/cgi-bin/SMD/source/sourceResult?choice=Gene&option=Name&criteria=THC2283605) | 0.58 | 0.0037 |
| A_32_P163996 | [THC2376737](http://genome-www4.stanford.edu/cgi-bin/SMD/source/sourceResult?choice=Gene&option=Name&criteria=THC2376737) | 0.56 | 0.0037 |
| A_23_P356585 | [HLF](http://genome-www4.stanford.edu/cgi-bin/SMD/source/sourceResult?choice=Gene&option=Name&criteria=HLF) | 0.63 | 0.0037 |
| A_32_P111492 | [A_32_P111492](http://genome-www4.stanford.edu/cgi-bin/SMD/source/sourceResult?choice=Gene&option=Name&criteria=A_32_P111492) | 0.59 | 0.0037 |
| A_24_P539226 | [A_24_P539226](http://genome-www4.stanford.edu/cgi-bin/SMD/source/sourceResult?choice=Gene&option=Name&criteria=A_24_P539226) | 0.66 | 0.0050 |
| A_24_P889980 | [BC047753](http://genome-www4.stanford.edu/cgi-bin/SMD/source/sourceResult?choice=Gene&option=Name&criteria=BC047753) | 0.64 | 0.0050 |
| A_23_P162449 | [SRGAP1](http://genome-www4.stanford.edu/cgi-bin/SMD/source/sourceResult?choice=Gene&option=Name&criteria=SRGAP1) | 0.67 | 0.0050 |
| A_23_P28898 | [PLCB4](http://genome-www4.stanford.edu/cgi-bin/SMD/source/sourceResult?choice=Gene&option=Name&criteria=PLCB4) | 0.65 | 0.0050 |
| A_23_P43484 | [CDKN2A](http://genome-www4.stanford.edu/cgi-bin/SMD/source/sourceResult?choice=Gene&option=Name&criteria=CDKN2A) | 0.63 | 0.0050 |
| A_32_P81324 | [THC2404993](http://genome-www4.stanford.edu/cgi-bin/SMD/source/sourceResult?choice=Gene&option=Name&criteria=THC2404993) | 0.55 | 0.0050 |
| A_24_P913828 | [MUC3A](http://genome-www4.stanford.edu/cgi-bin/SMD/source/sourceResult?choice=Gene&option=Name&criteria=MUC3A) | 0.64 | 0.0050 |
| A_32_P231346 | [THC2458855](http://genome-www4.stanford.edu/cgi-bin/SMD/source/sourceResult?choice=Gene&option=Name&criteria=THC2458855) | 0.66 | 0.0050 |
| A_24_P272451 | [UNQ5783](http://genome-www4.stanford.edu/cgi-bin/SMD/source/sourceResult?choice=Gene&option=Name&criteria=UNQ5783) | 0.54 | 0.0050 |
| A_23_P205355 | [SERPINA5](http://genome-www4.stanford.edu/cgi-bin/SMD/source/sourceResult?choice=Gene&option=Name&criteria=SERPINA5) | 0.61 | 0.0050 |
| A_24_P391811 | [DOCK10](http://genome-www4.stanford.edu/cgi-bin/SMD/source/sourceResult?choice=Gene&option=Name&criteria=DOCK10) | 0.59 | 0.0050 |
| A_23_P7483 | [BDP1](http://genome-www4.stanford.edu/cgi-bin/SMD/source/sourceResult?choice=Gene&option=Name&criteria=BDP1) | 0.65 | 0.0050 |
| A_32_P115277 | [THC2279466](http://genome-www4.stanford.edu/cgi-bin/SMD/source/sourceResult?choice=Gene&option=Name&criteria=THC2279466) | 0.65 | 0.0050 |
| A_32_P107002 | [LOC400509](http://genome-www4.stanford.edu/cgi-bin/SMD/source/sourceResult?choice=Gene&option=Name&criteria=LOC400509) | 0.63 | 0.0050 |
| A_32_P142943 | [THC2439682](http://genome-www4.stanford.edu/cgi-bin/SMD/source/sourceResult?choice=Gene&option=Name&criteria=THC2439682) | 0.63 | 0.0050 |
| A_32_P76122 | [A_32_P76122](http://genome-www4.stanford.edu/cgi-bin/SMD/source/sourceResult?choice=Gene&option=Name&criteria=A_32_P76122) | 0.63 | 0.0050 |
| A_23_P51767 | [CD1C](http://genome-www4.stanford.edu/cgi-bin/SMD/source/sourceResult?choice=Gene&option=Name&criteria=CD1C) | 0.64 | 0.0050 |
| A_32_P232035 | [THC2312585](http://genome-www4.stanford.edu/cgi-bin/SMD/source/sourceResult?choice=Gene&option=Name&criteria=THC2312585) | 0.63 | 0.0050 |
| A_23_P251881 | [NCR3](http://genome-www4.stanford.edu/cgi-bin/SMD/source/sourceResult?choice=Gene&option=Name&criteria=NCR3) | 0.65 | 0.0050 |
| A_24_P63468 | [AGXT2](http://genome-www4.stanford.edu/cgi-bin/SMD/source/sourceResult?choice=Gene&option=Name&criteria=AGXT2) | 0.51 | 0.0050 |
| A_32_P152544 | [THC2410524](http://genome-www4.stanford.edu/cgi-bin/SMD/source/sourceResult?choice=Gene&option=Name&criteria=THC2410524) | 0.63 | 0.0050 |
| A_24_P184692 | [TITF1](http://genome-www4.stanford.edu/cgi-bin/SMD/source/sourceResult?choice=Gene&option=Name&criteria=TITF1) | 0.58 | 0.0050 |
| A_32_P224727 | [BE798911](http://genome-www4.stanford.edu/cgi-bin/SMD/source/sourceResult?choice=Gene&option=Name&criteria=BE798911) | 0.63 | 0.0050 |
| A_24_P409182 | [ENST00000303979](http://genome-www4.stanford.edu/cgi-bin/SMD/source/sourceResult?choice=Gene&option=Name&criteria=ENST00000303979) | 0.52 | 0.0050 |
| A_24_P286412 | [RCP9](http://genome-www4.stanford.edu/cgi-bin/SMD/source/sourceResult?choice=Gene&option=Name&criteria=RCP9) | 0.66 | 0.0050 |
| A_24_P155761 | [AKAP13](http://genome-www4.stanford.edu/cgi-bin/SMD/source/sourceResult?choice=Gene&option=Name&criteria=AKAP13) | 0.64 | 0.0050 |
| A_23_P145424 | [KIAA1009](http://genome-www4.stanford.edu/cgi-bin/SMD/source/sourceResult?choice=Gene&option=Name&criteria=KIAA1009) | 0.63 | 0.0050 |
| A_23_P334414 | [TRAF3IP3](http://genome-www4.stanford.edu/cgi-bin/SMD/source/sourceResult?choice=Gene&option=Name&criteria=TRAF3IP3) | 0.66 | 0.0050 |
| A_32_P118325 | [BU567832](http://genome-www4.stanford.edu/cgi-bin/SMD/source/sourceResult?choice=Gene&option=Name&criteria=BU567832) | 0.63 | 0.0050 |
| A_32_P119604 | [THC2300570](http://genome-www4.stanford.edu/cgi-bin/SMD/source/sourceResult?choice=Gene&option=Name&criteria=THC2300570) | 0.63 | 0.0050 |
| A_23_P123853 | [CCL19](http://genome-www4.stanford.edu/cgi-bin/SMD/source/sourceResult?choice=Gene&option=Name&criteria=CCL19) | 0.45 | 0.0050 |
| A_23_P167121 | [GABRA2](http://genome-www4.stanford.edu/cgi-bin/SMD/source/sourceResult?choice=Gene&option=Name&criteria=GABRA2) | 0.65 | 0.0050 |
| A_24_P918137 | [AK096487](http://genome-www4.stanford.edu/cgi-bin/SMD/source/sourceResult?choice=Gene&option=Name&criteria=AK096487) | 0.63 | 0.0050 |
| A_23_P43157 | [MYBL1](http://genome-www4.stanford.edu/cgi-bin/SMD/source/sourceResult?choice=Gene&option=Name&criteria=MYBL1) | 0.66 | 0.0050 |
| A_23_P90333 | [ZNF404](http://genome-www4.stanford.edu/cgi-bin/SMD/source/sourceResult?choice=Gene&option=Name&criteria=ZNF404) | 0.64 | 0.0050 |
| A_32_P137336 | [BC013077](http://genome-www4.stanford.edu/cgi-bin/SMD/source/sourceResult?choice=Gene&option=Name&criteria=BC013077) | 0.58 | 0.0050 |
| A_24_P8165 | [SLC15A1](http://genome-www4.stanford.edu/cgi-bin/SMD/source/sourceResult?choice=Gene&option=Name&criteria=SLC15A1) | 0.64 | 0.0050 |
| A_32_P151152 | [AA451708](http://genome-www4.stanford.edu/cgi-bin/SMD/source/sourceResult?choice=Gene&option=Name&criteria=AA451708) | 0.55 | 0.0050 |
| A_32_P185361 | [AL109784](http://genome-www4.stanford.edu/cgi-bin/SMD/source/sourceResult?choice=Gene&option=Name&criteria=AL109784) | 0.66 | 0.0050 |
| A_32_P61439 | [THC2416008](http://genome-www4.stanford.edu/cgi-bin/SMD/source/sourceResult?choice=Gene&option=Name&criteria=THC2416008) | 0.64 | 0.0050 |
| A_32_P143516 | [C10orf6](http://genome-www4.stanford.edu/cgi-bin/SMD/source/sourceResult?choice=Gene&option=Name&criteria=C10orf6) | 0.66 | 0.0050 |
| A_24_P29733 | [PFTK1](http://genome-www4.stanford.edu/cgi-bin/SMD/source/sourceResult?choice=Gene&option=Name&criteria=PFTK1) | 0.63 | 0.0050 |
| A_24_P23482 | [CLYBL](http://genome-www4.stanford.edu/cgi-bin/SMD/source/sourceResult?choice=Gene&option=Name&criteria=CLYBL) | 0.66 | 0.0050 |
| A_24_P912228 | [AK095564](http://genome-www4.stanford.edu/cgi-bin/SMD/source/sourceResult?choice=Gene&option=Name&criteria=AK095564) | 0.63 | 0.0050 |
| A_23_P44956 | [LOC441136](http://genome-www4.stanford.edu/cgi-bin/SMD/source/sourceResult?choice=Gene&option=Name&criteria=LOC441136) | 0.65 | 0.0050 |
| A_24_P168925 | [ENST00000372045](http://genome-www4.stanford.edu/cgi-bin/SMD/source/sourceResult?choice=Gene&option=Name&criteria=ENST00000372045) | 0.59 | 0.0062 |
| A_32_P81173 | [USP34](http://genome-www4.stanford.edu/cgi-bin/SMD/source/sourceResult?choice=Gene&option=Name&criteria=USP34) | 0.64 | 0.0062 |
| A_32_P60707 | [THC2406514](http://genome-www4.stanford.edu/cgi-bin/SMD/source/sourceResult?choice=Gene&option=Name&criteria=THC2406514) | 0.66 | 0.0062 |
| A_23_P97123 | [RP4-747L4.3](http://genome-www4.stanford.edu/cgi-bin/SMD/source/sourceResult?choice=Gene&option=Name&criteria=RP4-747L4.3) | 0.60 | 0.0062 |
| A_32_P128368 | [H12329](http://genome-www4.stanford.edu/cgi-bin/SMD/source/sourceResult?choice=Gene&option=Name&criteria=H12329) | 0.53 | 0.0062 |
| A_32_P17343 | [THC2347909](http://genome-www4.stanford.edu/cgi-bin/SMD/source/sourceResult?choice=Gene&option=Name&criteria=THC2347909) | 0.55 | 0.0062 |
| A_23_P371410 | [PRKACB](http://genome-www4.stanford.edu/cgi-bin/SMD/source/sourceResult?choice=Gene&option=Name&criteria=PRKACB) | 0.59 | 0.0062 |
| A_24_P921683 | [FOXP2](http://genome-www4.stanford.edu/cgi-bin/SMD/source/sourceResult?choice=Gene&option=Name&criteria=FOXP2) | 0.64 | 0.0062 |
| A_23_P331348 | [DOCK7](http://genome-www4.stanford.edu/cgi-bin/SMD/source/sourceResult?choice=Gene&option=Name&criteria=DOCK7) | 0.65 | 0.0062 |
| A_32_P5040 | [NOTCH2NL](http://genome-www4.stanford.edu/cgi-bin/SMD/source/sourceResult?choice=Gene&option=Name&criteria=NOTCH2NL) | 0.61 | 0.0062 |
| A_32_P155512 | [BM982926](http://genome-www4.stanford.edu/cgi-bin/SMD/source/sourceResult?choice=Gene&option=Name&criteria=BM982926) | 0.58 | 0.0062 |
| A_24_P678741 | [ENST00000358431](http://genome-www4.stanford.edu/cgi-bin/SMD/source/sourceResult?choice=Gene&option=Name&criteria=ENST00000358431) | 0.62 | 0.0062 |
| A_24_P187131 | [FRYL](http://genome-www4.stanford.edu/cgi-bin/SMD/source/sourceResult?choice=Gene&option=Name&criteria=FRYL) | 0.63 | 0.0062 |
| A_24_P162423 | [BC034289](http://genome-www4.stanford.edu/cgi-bin/SMD/source/sourceResult?choice=Gene&option=Name&criteria=BC034289) | 0.65 | 0.0062 |
| A_32_P29442 | [AI911989](http://genome-www4.stanford.edu/cgi-bin/SMD/source/sourceResult?choice=Gene&option=Name&criteria=AI911989) | 0.66 | 0.0062 |
| A_32_P223504 | [AA563626](http://genome-www4.stanford.edu/cgi-bin/SMD/source/sourceResult?choice=Gene&option=Name&criteria=AA563626) | 0.64 | 0.0062 |
| A_32_P163594 | [A_32_P163594](http://genome-www4.stanford.edu/cgi-bin/SMD/source/sourceResult?choice=Gene&option=Name&criteria=A_32_P163594) | 0.62 | 0.0062 |
| A_23_P146997 | [CXorf15](http://genome-www4.stanford.edu/cgi-bin/SMD/source/sourceResult?choice=Gene&option=Name&criteria=CXorf15) | 0.67 | 0.0062 |
| A_24_P115287 | [AK001533](http://genome-www4.stanford.edu/cgi-bin/SMD/source/sourceResult?choice=Gene&option=Name&criteria=AK001533) | 0.61 | 0.0062 |
| A_23_P63032 | [GUCA2B](http://genome-www4.stanford.edu/cgi-bin/SMD/source/sourceResult?choice=Gene&option=Name&criteria=GUCA2B) | 0.62 | 0.0062 |
| A_24_P932016 | [HUWE1](http://genome-www4.stanford.edu/cgi-bin/SMD/source/sourceResult?choice=Gene&option=Name&criteria=HUWE1) | 0.65 | 0.0062 |
| A_32_P210038 | [BC021677](http://genome-www4.stanford.edu/cgi-bin/SMD/source/sourceResult?choice=Gene&option=Name&criteria=BC021677) | 0.66 | 0.0062 |
| A_23_P60006 | [ANXA13](http://genome-www4.stanford.edu/cgi-bin/SMD/source/sourceResult?choice=Gene&option=Name&criteria=ANXA13) | 0.60 | 0.0062 |
| A_32_P51707 | [THC2348290](http://genome-www4.stanford.edu/cgi-bin/SMD/source/sourceResult?choice=Gene&option=Name&criteria=THC2348290) | 0.63 | 0.0062 |
| A_23_P501722 | [TSPAN32](http://genome-www4.stanford.edu/cgi-bin/SMD/source/sourceResult?choice=Gene&option=Name&criteria=TSPAN32) | 0.61 | 0.0062 |
| A_24_P77904 | [HOXA10](http://genome-www4.stanford.edu/cgi-bin/SMD/source/sourceResult?choice=Gene&option=Name&criteria=HOXA10) | 0.54 | 0.0062 |
| A_32_P77571 | [AK024584](http://genome-www4.stanford.edu/cgi-bin/SMD/source/sourceResult?choice=Gene&option=Name&criteria=AK024584) | 0.57 | 0.0062 |
| A_24_P253454 | [RGMA](http://genome-www4.stanford.edu/cgi-bin/SMD/source/sourceResult?choice=Gene&option=Name&criteria=RGMA) | 0.53 | 0.0062 |
| A_23_P38757 | [SLC14A1](http://genome-www4.stanford.edu/cgi-bin/SMD/source/sourceResult?choice=Gene&option=Name&criteria=SLC14A1) | 0.60 | 0.0062 |
| A_24_P933492 | [ZDHHC21](http://genome-www4.stanford.edu/cgi-bin/SMD/source/sourceResult?choice=Gene&option=Name&criteria=ZDHHC21) | 0.66 | 0.0062 |
| A_23_P92928 | [C6](http://genome-www4.stanford.edu/cgi-bin/SMD/source/sourceResult?choice=Gene&option=Name&criteria=C6) | 0.49 | 0.0062 |
| A_23_P123478 | [PDE7A](http://genome-www4.stanford.edu/cgi-bin/SMD/source/sourceResult?choice=Gene&option=Name&criteria=PDE7A) | 0.63 | 0.0062 |
| A_24_P289260 | [DACT2](http://genome-www4.stanford.edu/cgi-bin/SMD/source/sourceResult?choice=Gene&option=Name&criteria=DACT2) | 0.58 | 0.0062 |
| A_32_P185741 | [THC2410251](http://genome-www4.stanford.edu/cgi-bin/SMD/source/sourceResult?choice=Gene&option=Name&criteria=THC2410251) | 0.53 | 0.0062 |
| A_24_P943283 | [FLJ20054](http://genome-www4.stanford.edu/cgi-bin/SMD/source/sourceResult?choice=Gene&option=Name&criteria=FLJ20054) | 0.64 | 0.0062 |
| A_23_P102000 | [CXCR4](http://genome-www4.stanford.edu/cgi-bin/SMD/source/sourceResult?choice=Gene&option=Name&criteria=CXCR4) | 0.56 | 0.0062 |
| A_32_P131342 | [THC2378263](http://genome-www4.stanford.edu/cgi-bin/SMD/source/sourceResult?choice=Gene&option=Name&criteria=THC2378263) | 0.65 | 0.0062 |
| A_23_P54736 | [GNG13](http://genome-www4.stanford.edu/cgi-bin/SMD/source/sourceResult?choice=Gene&option=Name&criteria=GNG13) | 0.62 | 0.0062 |
| A_24_P706983 | [THC2280557](http://genome-www4.stanford.edu/cgi-bin/SMD/source/sourceResult?choice=Gene&option=Name&criteria=THC2280557) | 0.63 | 0.0062 |
| A_24_P822869 | [AK092681](http://genome-www4.stanford.edu/cgi-bin/SMD/source/sourceResult?choice=Gene&option=Name&criteria=AK092681) | 0.67 | 0.0087 |
| A_32_P112531 | [A_32_P112531](http://genome-www4.stanford.edu/cgi-bin/SMD/source/sourceResult?choice=Gene&option=Name&criteria=A_32_P112531) | 0.66 | 0.0087 |
| A_23_P149946 | [PCDH21](http://genome-www4.stanford.edu/cgi-bin/SMD/source/sourceResult?choice=Gene&option=Name&criteria=PCDH21) | 0.63 | 0.0087 |
| A_24_P899020 | [A_24_P899020](http://genome-www4.stanford.edu/cgi-bin/SMD/source/sourceResult?choice=Gene&option=Name&criteria=A_24_P899020) | 0.63 | 0.0087 |
| A_23_P148249 | [THSD4](http://genome-www4.stanford.edu/cgi-bin/SMD/source/sourceResult?choice=Gene&option=Name&criteria=THSD4) | 0.64 | 0.0087 |
| A_23_P124934 | [ZNFN1A1](http://genome-www4.stanford.edu/cgi-bin/SMD/source/sourceResult?choice=Gene&option=Name&criteria=ZNFN1A1) | 0.66 | 0.0087 |
| A_24_P529644 | [H6PD](http://genome-www4.stanford.edu/cgi-bin/SMD/source/sourceResult?choice=Gene&option=Name&criteria=H6PD) | 0.62 | 0.0087 |
| A_23_P8311 | [TTRAP](http://genome-www4.stanford.edu/cgi-bin/SMD/source/sourceResult?choice=Gene&option=Name&criteria=TTRAP) | 0.67 | 0.0087 |
| A_23_P156811 | [LOC389286](http://genome-www4.stanford.edu/cgi-bin/SMD/source/sourceResult?choice=Gene&option=Name&criteria=LOC389286) | 0.65 | 0.0087 |
| A_24_P143492 | [BCAS4](http://genome-www4.stanford.edu/cgi-bin/SMD/source/sourceResult?choice=Gene&option=Name&criteria=BCAS4) | 0.64 | 0.0087 |
| A_32_P146826 | [THC2441367](http://genome-www4.stanford.edu/cgi-bin/SMD/source/sourceResult?choice=Gene&option=Name&criteria=THC2441367) | 0.61 | 0.0087 |
| A_23_P42530 | [DST](http://genome-www4.stanford.edu/cgi-bin/SMD/source/sourceResult?choice=Gene&option=Name&criteria=DST) | 0.65 | 0.0087 |
| A_32_P879150 | [THC2278737](http://genome-www4.stanford.edu/cgi-bin/SMD/source/sourceResult?choice=Gene&option=Name&criteria=THC2278737) | 0.56 | 0.0087 |
| A_32_P194182 | [A_32_P194182](http://genome-www4.stanford.edu/cgi-bin/SMD/source/sourceResult?choice=Gene&option=Name&criteria=A_32_P194182) | 0.66 | 0.0087 |
| A_32_P144390 | [THC2317006](http://genome-www4.stanford.edu/cgi-bin/SMD/source/sourceResult?choice=Gene&option=Name&criteria=THC2317006) | 0.67 | 0.0087 |
| A_24_P341000 | [AK092698](http://genome-www4.stanford.edu/cgi-bin/SMD/source/sourceResult?choice=Gene&option=Name&criteria=AK092698) | 0.50 | 0.0087 |
| A_32_P81806 | [THC2406779](http://genome-www4.stanford.edu/cgi-bin/SMD/source/sourceResult?choice=Gene&option=Name&criteria=THC2406779) | 0.66 | 0.0087 |
| A_24_P111096 | [PFKFB3](http://genome-www4.stanford.edu/cgi-bin/SMD/source/sourceResult?choice=Gene&option=Name&criteria=PFKFB3) | 0.49 | 0.0087 |
| A_32_P108156 | [BIC](http://genome-www4.stanford.edu/cgi-bin/SMD/source/sourceResult?choice=Gene&option=Name&criteria=BIC) | 0.63 | 0.0087 |
| A_32_P33114 | [ENST00000381889](http://genome-www4.stanford.edu/cgi-bin/SMD/source/sourceResult?choice=Gene&option=Name&criteria=ENST00000381889) | 0.65 | 0.0087 |
| A_23_P333218 | [ERGIC1](http://genome-www4.stanford.edu/cgi-bin/SMD/source/sourceResult?choice=Gene&option=Name&criteria=ERGIC1) | 0.66 | 0.0087 |
| A_24_P84719 | [A_24_P84719](http://genome-www4.stanford.edu/cgi-bin/SMD/source/sourceResult?choice=Gene&option=Name&criteria=A_24_P84719) | 0.66 | 0.0087 |
| A_24_P360206 | [PCDHA11](http://genome-www4.stanford.edu/cgi-bin/SMD/source/sourceResult?choice=Gene&option=Name&criteria=PCDHA11) | 0.66 | 0.0087 |
| A_32_P7974 | [TDRD10](http://genome-www4.stanford.edu/cgi-bin/SMD/source/sourceResult?choice=Gene&option=Name&criteria=TDRD10) | 0.56 | 0.0115 |
| A_23_P378555 | [PARP15](http://genome-www4.stanford.edu/cgi-bin/SMD/source/sourceResult?choice=Gene&option=Name&criteria=PARP15) | 0.64 | 0.0115 |
| A_23_P3083 | [THC2331323](http://genome-www4.stanford.edu/cgi-bin/SMD/source/sourceResult?choice=Gene&option=Name&criteria=THC2331323) | 0.57 | 0.0115 |
| A_32_P227648 | [THC2376828](http://genome-www4.stanford.edu/cgi-bin/SMD/source/sourceResult?choice=Gene&option=Name&criteria=THC2376828) | 0.64 | 0.0115 |
| A_24_P321581 | [SLC38A4](http://genome-www4.stanford.edu/cgi-bin/SMD/source/sourceResult?choice=Gene&option=Name&criteria=SLC38A4) | 0.57 | 0.0115 |
| A_23_P159076 | [SLC17A8](http://genome-www4.stanford.edu/cgi-bin/SMD/source/sourceResult?choice=Gene&option=Name&criteria=SLC17A8) | 0.59 | 0.0115 |
| A_24_P50738 | [AF035020](http://genome-www4.stanford.edu/cgi-bin/SMD/source/sourceResult?choice=Gene&option=Name&criteria=AF035020) | 0.66 | 0.0115 |
| A_23_P99386 | [TNFSF11](http://genome-www4.stanford.edu/cgi-bin/SMD/source/sourceResult?choice=Gene&option=Name&criteria=TNFSF11) | 0.59 | 0.0115 |
| A_24_P206604 | [PFKFB3](http://genome-www4.stanford.edu/cgi-bin/SMD/source/sourceResult?choice=Gene&option=Name&criteria=PFKFB3) | 0.63 | 0.0115 |
| A_23_P131074 | [THEG](http://genome-www4.stanford.edu/cgi-bin/SMD/source/sourceResult?choice=Gene&option=Name&criteria=THEG) | 0.64 | 0.0115 |
| A_23_P168288 | [IL22RA2](http://genome-www4.stanford.edu/cgi-bin/SMD/source/sourceResult?choice=Gene&option=Name&criteria=IL22RA2) | 0.63 | 0.0115 |
| A_23_P7262 | MARCH1 | 0.64 | 0.0115 |
| A_23_P12082 | [CHI3L2](http://genome-www4.stanford.edu/cgi-bin/SMD/source/sourceResult?choice=Gene&option=Name&criteria=CHI3L2) | 0.62 | 0.0115 |
| A_23_P70670 | [CD83](http://genome-www4.stanford.edu/cgi-bin/SMD/source/sourceResult?choice=Gene&option=Name&criteria=CD83) | 0.67 | 0.0115 |
| A_24_P332081 | [C10orf39](http://genome-www4.stanford.edu/cgi-bin/SMD/source/sourceResult?choice=Gene&option=Name&criteria=C10orf39) | 0.52 | 0.0115 |
| A_24_P215407 | [DDX6](http://genome-www4.stanford.edu/cgi-bin/SMD/source/sourceResult?choice=Gene&option=Name&criteria=DDX6) | 0.65 | 0.0115 |
| A_24_P194661 | [FLJ38379](http://genome-www4.stanford.edu/cgi-bin/SMD/source/sourceResult?choice=Gene&option=Name&criteria=FLJ38379) | 0.60 | 0.0115 |
| A_23_P205408 | [MIA2](http://genome-www4.stanford.edu/cgi-bin/SMD/source/sourceResult?choice=Gene&option=Name&criteria=MIA2) | 0.64 | 0.0115 |
| A_24_P595223 | [C22orf35](http://genome-www4.stanford.edu/cgi-bin/SMD/source/sourceResult?choice=Gene&option=Name&criteria=C22orf35) | 0.66 | 0.0115 |
| A_24_P153456 | [ZDHHC11](http://genome-www4.stanford.edu/cgi-bin/SMD/source/sourceResult?choice=Gene&option=Name&criteria=ZDHHC11) | 0.58 | 0.0139 |
| A_24_P874508 | [THC2446542](http://genome-www4.stanford.edu/cgi-bin/SMD/source/sourceResult?choice=Gene&option=Name&criteria=THC2446542) | 0.60 | 0.0139 |
| A_32_P45285 | [THC2442277](http://genome-www4.stanford.edu/cgi-bin/SMD/source/sourceResult?choice=Gene&option=Name&criteria=THC2442277) | 0.66 | 0.0139 |
| A_24_P287403 | [HSMPP8](http://genome-www4.stanford.edu/cgi-bin/SMD/source/sourceResult?choice=Gene&option=Name&criteria=HSMPP8) | 0.60 | 0.0139 |
| A_23_P217009 | [C9orf24](http://genome-www4.stanford.edu/cgi-bin/SMD/source/sourceResult?choice=Gene&option=Name&criteria=C9orf24) | 0.64 | 0.0139 |
| A_32_P64263 | [BQ029628](http://genome-www4.stanford.edu/cgi-bin/SMD/source/sourceResult?choice=Gene&option=Name&criteria=BQ029628) | 0.61 | 0.0139 |
| A_32_P160398 | [AI613259](http://genome-www4.stanford.edu/cgi-bin/SMD/source/sourceResult?choice=Gene&option=Name&criteria=AI613259) | 0.61 | 0.0139 |
| A_24_P928031 | [A_24_P928031](http://genome-www4.stanford.edu/cgi-bin/SMD/source/sourceResult?choice=Gene&option=Name&criteria=A_24_P928031) | 0.55 | 0.0139 |
| A_32_P128857 | [LOC286272](http://genome-www4.stanford.edu/cgi-bin/SMD/source/sourceResult?choice=Gene&option=Name&criteria=LOC286272) | 0.66 | 0.0139 |
| A_23_P209055 | [CD22](http://genome-www4.stanford.edu/cgi-bin/SMD/source/sourceResult?choice=Gene&option=Name&criteria=CD22) | 0.60 | 0.0139 |
| A_32_P216015 | [THC2301901](http://genome-www4.stanford.edu/cgi-bin/SMD/source/sourceResult?choice=Gene&option=Name&criteria=THC2301901) | 0.66 | 0.0139 |
| A_32_P100475 | [A_32_P100475](http://genome-www4.stanford.edu/cgi-bin/SMD/source/sourceResult?choice=Gene&option=Name&criteria=A_32_P100475) | 0.67 | 0.0139 |
| A_23_P55281 | [HOXB7](http://genome-www4.stanford.edu/cgi-bin/SMD/source/sourceResult?choice=Gene&option=Name&criteria=HOXB7) | 0.66 | 0.0139 |
| A_24_P580248 | [THC2269852](http://genome-www4.stanford.edu/cgi-bin/SMD/source/sourceResult?choice=Gene&option=Name&criteria=THC2269852) | 0.64 | 0.0139 |
| A_23_P103486 | [CYP2J2](http://genome-www4.stanford.edu/cgi-bin/SMD/source/sourceResult?choice=Gene&option=Name&criteria=CYP2J2) | 0.67 | 0.0164 |
| A_23_P40174 | [MMP9](http://genome-www4.stanford.edu/cgi-bin/SMD/source/sourceResult?choice=Gene&option=Name&criteria=MMP9) | 0.63 | 0.0164 |
| A_23_P131024 | [ZBTB32](http://genome-www4.stanford.edu/cgi-bin/SMD/source/sourceResult?choice=Gene&option=Name&criteria=ZBTB32) | 0.61 | 0.0164 |
| A_23_P207371 | [G6PC](http://genome-www4.stanford.edu/cgi-bin/SMD/source/sourceResult?choice=Gene&option=Name&criteria=G6PC) | 0.64 | 0.0164 |
| A_23_P201211 | [FCRL5](http://genome-www4.stanford.edu/cgi-bin/SMD/source/sourceResult?choice=Gene&option=Name&criteria=FCRL5) | 0.57 | 0.0164 |
| A_32_P225472 | [LOC642398](http://genome-www4.stanford.edu/cgi-bin/SMD/source/sourceResult?choice=Gene&option=Name&criteria=LOC642398) | 0.67 | 0.0164 |
| A_32_P888644 | [MGC32805](http://genome-www4.stanford.edu/cgi-bin/SMD/source/sourceResult?choice=Gene&option=Name&criteria=MGC32805) | 0.61 | 0.0164 |
| A_24_P214598 | [PPM1K](http://genome-www4.stanford.edu/cgi-bin/SMD/source/sourceResult?choice=Gene&option=Name&criteria=PPM1K) | 0.66 | 0.0164 |
| A_23_P149613 | [FMO1](http://genome-www4.stanford.edu/cgi-bin/SMD/source/sourceResult?choice=Gene&option=Name&criteria=FMO1) | 0.60 | 0.0196 |
| A_24_P93656 | [CAND1](http://genome-www4.stanford.edu/cgi-bin/SMD/source/sourceResult?choice=Gene&option=Name&criteria=CAND1) | 0.66 | 0.0196 |
| A_24_P169507 | [MUC17](http://genome-www4.stanford.edu/cgi-bin/SMD/source/sourceResult?choice=Gene&option=Name&criteria=MUC17) | 0.63 | 0.0196 |
| A_23_P49145 | [ZG16](http://genome-www4.stanford.edu/cgi-bin/SMD/source/sourceResult?choice=Gene&option=Name&criteria=ZG16) | 0.63 | 0.0196 |
| A_23_P112726 | [SCN9A](http://genome-www4.stanford.edu/cgi-bin/SMD/source/sourceResult?choice=Gene&option=Name&criteria=SCN9A) | 0.64 | 0.0196 |
| A_32_P34138 | [ENST00000343959](http://genome-www4.stanford.edu/cgi-bin/SMD/source/sourceResult?choice=Gene&option=Name&criteria=ENST00000343959) | 0.63 | 0.0196 |
| A_32_P119704 | [THC2406165](http://genome-www4.stanford.edu/cgi-bin/SMD/source/sourceResult?choice=Gene&option=Name&criteria=THC2406165) | 0.66 | 0.0196 |
| A_24_P375205 | [ENST00000343519](http://genome-www4.stanford.edu/cgi-bin/SMD/source/sourceResult?choice=Gene&option=Name&criteria=ENST00000343519) | 0.60 | 0.0214 |
| A_23_P31858 | [ST18](http://genome-www4.stanford.edu/cgi-bin/SMD/source/sourceResult?choice=Gene&option=Name&criteria=ST18) | 0.64 | 0.0214 |
| A_32_P81282 | [THC2282190](http://genome-www4.stanford.edu/cgi-bin/SMD/source/sourceResult?choice=Gene&option=Name&criteria=THC2282190) | 0.66 | 0.0214 |
| A_23_P312920 | [POU2AF1](http://genome-www4.stanford.edu/cgi-bin/SMD/source/sourceResult?choice=Gene&option=Name&criteria=POU2AF1) | 0.64 | 0.0214 |
| A_23_P256008 | [ZDHHC11](http://genome-www4.stanford.edu/cgi-bin/SMD/source/sourceResult?choice=Gene&option=Name&criteria=ZDHHC11) | 0.63 | 0.0214 |
| A_24_P247643 | [MUC17](http://genome-www4.stanford.edu/cgi-bin/SMD/source/sourceResult?choice=Gene&option=Name&criteria=MUC17) | 0.64 | 0.0214 |
| A_23_P253368 | [HOXA10](http://genome-www4.stanford.edu/cgi-bin/SMD/source/sourceResult?choice=Gene&option=Name&criteria=HOXA10) | 0.61 | 0.0249 |
| A_23_P67367 | [DHDH](http://genome-www4.stanford.edu/cgi-bin/SMD/source/sourceResult?choice=Gene&option=Name&criteria=DHDH) | 0.63 | 0.0249 |
| A_24_P95723 | [KIAA0125](http://genome-www4.stanford.edu/cgi-bin/SMD/source/sourceResult?choice=Gene&option=Name&criteria=KIAA0125) | 0.65 | 0.0249 |
| A_23_P218858 | [ABI3BP](http://genome-www4.stanford.edu/cgi-bin/SMD/source/sourceResult?choice=Gene&option=Name&criteria=ABI3BP) | 0.66 | 0.0249 |
| A_23_P372234 | [CA12](http://genome-www4.stanford.edu/cgi-bin/SMD/source/sourceResult?choice=Gene&option=Name&criteria=CA12) | 0.65 | 0.0249 |
| A_24_P921343 | [BC017851](http://genome-www4.stanford.edu/cgi-bin/SMD/source/sourceResult?choice=Gene&option=Name&criteria=BC017851) | 0.66 | 0.0249 |
| A_23_P71226 | [SLC13A1](http://genome-www4.stanford.edu/cgi-bin/SMD/source/sourceResult?choice=Gene&option=Name&criteria=SLC13A1) | 0.65 | 0.0249 |
| A_32_P133840 | [TMCC2](http://genome-www4.stanford.edu/cgi-bin/SMD/source/sourceResult?choice=Gene&option=Name&criteria=TMCC2) | 0.65 | 0.0249 |
| A_32_P113066 | [LRAT](http://genome-www4.stanford.edu/cgi-bin/SMD/source/sourceResult?choice=Gene&option=Name&criteria=LRAT) | 0.55 | 0.0249 |
| A_32_P201958 | [ENST00000366971](http://genome-www4.stanford.edu/cgi-bin/SMD/source/sourceResult?choice=Gene&option=Name&criteria=ENST00000366971) | 0.65 | 0.0279 |
| A_32_P29551 | [THC2450799](http://genome-www4.stanford.edu/cgi-bin/SMD/source/sourceResult?choice=Gene&option=Name&criteria=THC2450799) | 0.65 | 0.0279 |
| A_24_P60217 | [SLC23A3](http://genome-www4.stanford.edu/cgi-bin/SMD/source/sourceResult?choice=Gene&option=Name&criteria=SLC23A3) | 0.66 | 0.0279 |
| A_23_P163336 | [CA12](http://genome-www4.stanford.edu/cgi-bin/SMD/source/sourceResult?choice=Gene&option=Name&criteria=CA12) | 0.66 | 0.0279 |
| A_24_P725998 | [THC2460656](http://genome-www4.stanford.edu/cgi-bin/SMD/source/sourceResult?choice=Gene&option=Name&criteria=THC2460656) | 0.66 | 0.0344 |
| A_24_P935147 | [AF119905](http://genome-www4.stanford.edu/cgi-bin/SMD/source/sourceResult?choice=Gene&option=Name&criteria=AF119905) | 0.66 | 0.0344 |
| A_24_P355057 | [SLC13A1](http://genome-www4.stanford.edu/cgi-bin/SMD/source/sourceResult?choice=Gene&option=Name&criteria=SLC13A1) | 0.54 | 0.0367 |
| A_24_P124647 | [SLC17A8](http://genome-www4.stanford.edu/cgi-bin/SMD/source/sourceResult?choice=Gene&option=Name&criteria=SLC17A8) | 0.57 | 0.0367 |
| A_23_P102172 | [CPO](http://genome-www4.stanford.edu/cgi-bin/SMD/source/sourceResult?choice=Gene&option=Name&criteria=CPO) | 0.64 | 0.0367 |
| A_23_P407695 | [C1orf179](http://genome-www4.stanford.edu/cgi-bin/SMD/source/sourceResult?choice=Gene&option=Name&criteria=C1orf179) | 0.64 | 0.0367 |
| A_32_P80016 | [N47124](http://genome-www4.stanford.edu/cgi-bin/SMD/source/sourceResult?choice=Gene&option=Name&criteria=N47124) | 0.65 | 0.0367 |
| A_24_P500584 | [XIST](http://genome-www4.stanford.edu/cgi-bin/SMD/source/sourceResult?choice=Gene&option=Name&criteria=XIST) | 0.52 | 0.0384 |
| A_23_P501624 | [UGT2B17](http://genome-www4.stanford.edu/cgi-bin/SMD/source/sourceResult?choice=Gene&option=Name&criteria=UGT2B17) | 0.65 | 0.0421 |
| A_24_P934387 | [AL049270](http://genome-www4.stanford.edu/cgi-bin/SMD/source/sourceResult?choice=Gene&option=Name&criteria=AL049270) | 0.66 | 0.0437 |
|  |  |  |  |
| **Supplementary Table S1C. Gene-probes upregulated in UC compared to Control (n=371)** | | | |
| **Agilent ID** | **Gene Name or Accession Number** | **Fold Change** | **q-value** |
| A_23_P76145 | [ARNTL2](http://genome-www4.stanford.edu/cgi-bin/SMD/source/sourceResult?choice=Gene&option=Name&criteria=ARNTL2) | 2.85 | <0.0001 |
| A_24_P941217 | [SGPP2](http://genome-www4.stanford.edu/cgi-bin/SMD/source/sourceResult?choice=Gene&option=Name&criteria=SGPP2) | 3.16 | <0.0001 |
| A_32_P70519 | [THC2397883](http://genome-www4.stanford.edu/cgi-bin/SMD/source/sourceResult?choice=Gene&option=Name&criteria=THC2397883) | 2.80 | <0.0001 |
| A_23_P101351 | [ZNF426](http://genome-www4.stanford.edu/cgi-bin/SMD/source/sourceResult?choice=Gene&option=Name&criteria=ZNF426) | 2.29 | <0.0001 |
| A_23_P89680 | [THC2305336](http://genome-www4.stanford.edu/cgi-bin/SMD/source/sourceResult?choice=Gene&option=Name&criteria=THC2305336) | 2.17 | <0.0001 |
| A_32_P95147 | [BC038559](http://genome-www4.stanford.edu/cgi-bin/SMD/source/sourceResult?choice=Gene&option=Name&criteria=BC038559) | 1.76 | <0.0001 |
| A_23_P74001 | [S100A12](http://genome-www4.stanford.edu/cgi-bin/SMD/source/sourceResult?choice=Gene&option=Name&criteria=S100A12) | 2.72 | <0.0001 |
| A_24_P323545 | [MYH14](http://genome-www4.stanford.edu/cgi-bin/SMD/source/sourceResult?choice=Gene&option=Name&criteria=MYH14) | 1.82 | <0.0001 |
| A_32_P114615 | [A_32_P114615](http://genome-www4.stanford.edu/cgi-bin/SMD/source/sourceResult?choice=Gene&option=Name&criteria=A_32_P114615) | 2.24 | <0.0001 |
| A_24_P649829 | [AK023338](http://genome-www4.stanford.edu/cgi-bin/SMD/source/sourceResult?choice=Gene&option=Name&criteria=AK023338) | 2.45 | <0.0001 |
| A_32_P72553 | [LOC162073](http://genome-www4.stanford.edu/cgi-bin/SMD/source/sourceResult?choice=Gene&option=Name&criteria=LOC162073) | 2.58 | <0.0001 |
| A_23_P203888 | [MMP19](http://genome-www4.stanford.edu/cgi-bin/SMD/source/sourceResult?choice=Gene&option=Name&criteria=MMP19) | 1.65 | <0.0001 |
| A_23_P434809 | [S100A8](http://genome-www4.stanford.edu/cgi-bin/SMD/source/sourceResult?choice=Gene&option=Name&criteria=S100A8) | 3.65 | <0.0001 |
| A_24_P127701 | [LOC441320](http://genome-www4.stanford.edu/cgi-bin/SMD/source/sourceResult?choice=Gene&option=Name&criteria=LOC441320) | 1.69 | <0.0001 |
| A_32_P142827 | [BF370042](http://genome-www4.stanford.edu/cgi-bin/SMD/source/sourceResult?choice=Gene&option=Name&criteria=BF370042) | 1.89 | <0.0001 |
| A_24_P8257 | [BC009036](http://genome-www4.stanford.edu/cgi-bin/SMD/source/sourceResult?choice=Gene&option=Name&criteria=BC009036) | 1.90 | <0.0001 |
| A_24_P935652 | [NYREN18](http://genome-www4.stanford.edu/cgi-bin/SMD/source/sourceResult?choice=Gene&option=Name&criteria=NYREN18) | 1.78 | <0.0001 |
| A_32_P105469 | [AK074696](http://genome-www4.stanford.edu/cgi-bin/SMD/source/sourceResult?choice=Gene&option=Name&criteria=AK074696) | 1.96 | <0.0001 |
| A_32_P107441 | [AA076992](http://genome-www4.stanford.edu/cgi-bin/SMD/source/sourceResult?choice=Gene&option=Name&criteria=AA076992) | 1.60 | <0.0001 |
| A_23_P93709 | [AK098134](http://genome-www4.stanford.edu/cgi-bin/SMD/source/sourceResult?choice=Gene&option=Name&criteria=AK098134) | 1.77 | <0.0001 |
| A_23_P424712 | [FLJ14397](http://genome-www4.stanford.edu/cgi-bin/SMD/source/sourceResult?choice=Gene&option=Name&criteria=FLJ14397) | 1.61 | <0.0001 |
| A_24_P145035 | [RP11-561O23.4](http://genome-www4.stanford.edu/cgi-bin/SMD/source/sourceResult?choice=Gene&option=Name&criteria=RP11-561O23.4) | 2.16 | <0.0001 |
| A_24_P740022 | [A_24_P740022](http://genome-www4.stanford.edu/cgi-bin/SMD/source/sourceResult?choice=Gene&option=Name&criteria=A_24_P740022) | 1.69 | <0.0001 |
| A_32_P133526 | [A_32_P133526](http://genome-www4.stanford.edu/cgi-bin/SMD/source/sourceResult?choice=Gene&option=Name&criteria=A_32_P133526) | 2.04 | <0.0001 |
| A_32_P88555 | [A_32_P88555](http://genome-www4.stanford.edu/cgi-bin/SMD/source/sourceResult?choice=Gene&option=Name&criteria=A_32_P88555) | 1.91 | <0.0001 |
| A_23_P369666 | [PRKCBP1](http://genome-www4.stanford.edu/cgi-bin/SMD/source/sourceResult?choice=Gene&option=Name&criteria=PRKCBP1) | 1.94 | <0.0001 |
| A_24_P238046 | [AK023376](http://genome-www4.stanford.edu/cgi-bin/SMD/source/sourceResult?choice=Gene&option=Name&criteria=AK023376) | 1.80 | <0.0001 |
| A_23_P364236 | [MGA](http://genome-www4.stanford.edu/cgi-bin/SMD/source/sourceResult?choice=Gene&option=Name&criteria=MGA) | 2.43 | <0.0001 |
| A_24_P450372 | [L40520](http://genome-www4.stanford.edu/cgi-bin/SMD/source/sourceResult?choice=Gene&option=Name&criteria=L40520) | 2.19 | <0.0001 |
| A_32_P181339 | [A_32_P181339](http://genome-www4.stanford.edu/cgi-bin/SMD/source/sourceResult?choice=Gene&option=Name&criteria=A_32_P181339) | 1.88 | <0.0001 |
| A_23_P400036 | [BC012036](http://genome-www4.stanford.edu/cgi-bin/SMD/source/sourceResult?choice=Gene&option=Name&criteria=BC012036) | 2.02 | <0.0001 |
| A_23_P138105 | [MED18](http://genome-www4.stanford.edu/cgi-bin/SMD/source/sourceResult?choice=Gene&option=Name&criteria=MED18) | 2.08 | <0.0001 |
| A_24_P357809 | [C11orf17](http://genome-www4.stanford.edu/cgi-bin/SMD/source/sourceResult?choice=Gene&option=Name&criteria=C11orf17) | 1.65 | <0.0001 |
| A_24_P919733 | [LOC152719](http://genome-www4.stanford.edu/cgi-bin/SMD/source/sourceResult?choice=Gene&option=Name&criteria=LOC152719) | 1.83 | <0.0001 |
| A_23_P57007 | [AF090938](http://genome-www4.stanford.edu/cgi-bin/SMD/source/sourceResult?choice=Gene&option=Name&criteria=AF090938) | 1.50 | <0.0001 |
| A_23_P31816 | [DEFA3](http://genome-www4.stanford.edu/cgi-bin/SMD/source/sourceResult?choice=Gene&option=Name&criteria=DEFA3) | 2.82 | <0.0001 |
| A_24_P608007 | [AK022390](http://genome-www4.stanford.edu/cgi-bin/SMD/source/sourceResult?choice=Gene&option=Name&criteria=AK022390) | 2.03 | <0.0001 |
| A_23_P144704 | [ENST00000330731](http://genome-www4.stanford.edu/cgi-bin/SMD/source/sourceResult?choice=Gene&option=Name&criteria=ENST00000330731) | 1.90 | <0.0001 |
| A_23_P101141 | [RNF125](http://genome-www4.stanford.edu/cgi-bin/SMD/source/sourceResult?choice=Gene&option=Name&criteria=RNF125) | 2.37 | <0.0001 |
| A_23_P151851 | [DUOX2](http://genome-www4.stanford.edu/cgi-bin/SMD/source/sourceResult?choice=Gene&option=Name&criteria=DUOX2) | 3.44 | <0.0001 |
| A_23_P15621 | [PRAC](http://genome-www4.stanford.edu/cgi-bin/SMD/source/sourceResult?choice=Gene&option=Name&criteria=PRAC) | 1.61 | <0.0001 |
| A_32_P44775 | [C9orf85](http://genome-www4.stanford.edu/cgi-bin/SMD/source/sourceResult?choice=Gene&option=Name&criteria=C9orf85) | 1.76 | <0.0001 |
| A_23_P23048 | [S100A9](http://genome-www4.stanford.edu/cgi-bin/SMD/source/sourceResult?choice=Gene&option=Name&criteria=S100A9) | 2.43 | <0.0001 |
| A_23_P157875 | [FCN1](http://genome-www4.stanford.edu/cgi-bin/SMD/source/sourceResult?choice=Gene&option=Name&criteria=FCN1) | 1.81 | <0.0001 |
| A_24_P940615 | [AK090407](http://genome-www4.stanford.edu/cgi-bin/SMD/source/sourceResult?choice=Gene&option=Name&criteria=AK090407) | 2.20 | <0.0001 |
| A_23_P23234 | [MGC5457](http://genome-www4.stanford.edu/cgi-bin/SMD/source/sourceResult?choice=Gene&option=Name&criteria=MGC5457) | 1.85 | <0.0001 |
| A_23_P115902 | [A_23_P115902](http://genome-www4.stanford.edu/cgi-bin/SMD/source/sourceResult?choice=Gene&option=Name&criteria=A_23_P115902) | 1.79 | <0.0001 |
| A_32_P136477 | [A_32_P136477](http://genome-www4.stanford.edu/cgi-bin/SMD/source/sourceResult?choice=Gene&option=Name&criteria=A_32_P136477) | 1.59 | <0.0001 |
| A_24_P111019 | [BC028243](http://genome-www4.stanford.edu/cgi-bin/SMD/source/sourceResult?choice=Gene&option=Name&criteria=BC028243) | 1.81 | <0.0001 |
| A_23_P13929 | [NRIP2](http://genome-www4.stanford.edu/cgi-bin/SMD/source/sourceResult?choice=Gene&option=Name&criteria=NRIP2) | 1.58 | <0.0001 |
| A_23_P49686 | [AF090926](http://genome-www4.stanford.edu/cgi-bin/SMD/source/sourceResult?choice=Gene&option=Name&criteria=AF090926) | 1.96 | <0.0001 |
| A_24_P503731 | [AK024315](http://genome-www4.stanford.edu/cgi-bin/SMD/source/sourceResult?choice=Gene&option=Name&criteria=AK024315) | 1.50 | <0.0001 |
| A_24_P310224 | [ZNF347](http://genome-www4.stanford.edu/cgi-bin/SMD/source/sourceResult?choice=Gene&option=Name&criteria=ZNF347) | 1.80 | <0.0001 |
| A_23_P395954 | [SSH2](http://genome-www4.stanford.edu/cgi-bin/SMD/source/sourceResult?choice=Gene&option=Name&criteria=SSH2) | 1.69 | <0.0001 |
| A_23_P128613 | [KDELC1](http://genome-www4.stanford.edu/cgi-bin/SMD/source/sourceResult?choice=Gene&option=Name&criteria=KDELC1) | 1.67 | <0.0001 |
| A_23_P78975 | [A_23_P78975](http://genome-www4.stanford.edu/cgi-bin/SMD/source/sourceResult?choice=Gene&option=Name&criteria=A_23_P78975) | 1.66 | <0.0001 |
| A_32_P23096 | [A_32_P23096](http://genome-www4.stanford.edu/cgi-bin/SMD/source/sourceResult?choice=Gene&option=Name&criteria=A_32_P23096) | 2.40 | <0.0001 |
| A_24_P307974 | [TBN](http://genome-www4.stanford.edu/cgi-bin/SMD/source/sourceResult?choice=Gene&option=Name&criteria=TBN) | 1.59 | <0.0001 |
| A_32_P140501 | [A_32_P140501](http://genome-www4.stanford.edu/cgi-bin/SMD/source/sourceResult?choice=Gene&option=Name&criteria=A_32_P140501) | 1.99 | <0.0001 |
| A_24_P134942 | [VHL](http://genome-www4.stanford.edu/cgi-bin/SMD/source/sourceResult?choice=Gene&option=Name&criteria=VHL) | 1.74 | <0.0001 |
| A_24_P321715 | [THC2266257](http://genome-www4.stanford.edu/cgi-bin/SMD/source/sourceResult?choice=Gene&option=Name&criteria=THC2266257) | 1.80 | <0.0001 |
| A_23_P35916 | [ATM](http://genome-www4.stanford.edu/cgi-bin/SMD/source/sourceResult?choice=Gene&option=Name&criteria=ATM) | 2.19 | <0.0001 |
| A_32_P222872 | [A_32_P222872](http://genome-www4.stanford.edu/cgi-bin/SMD/source/sourceResult?choice=Gene&option=Name&criteria=A_32_P222872) | 1.64 | <0.0001 |
| A_24_P937435 | [RBED1](http://genome-www4.stanford.edu/cgi-bin/SMD/source/sourceResult?choice=Gene&option=Name&criteria=RBED1) | 1.86 | <0.0001 |
| A_23_P306203 | [SAA2](http://genome-www4.stanford.edu/cgi-bin/SMD/source/sourceResult?choice=Gene&option=Name&criteria=SAA2) | 2.00 | <0.0001 |
| A_32_P50603 | [LOC339778](http://genome-www4.stanford.edu/cgi-bin/SMD/source/sourceResult?choice=Gene&option=Name&criteria=LOC339778) | 1.57 | <0.0001 |
| A_23_P88021 | [GPR180](http://genome-www4.stanford.edu/cgi-bin/SMD/source/sourceResult?choice=Gene&option=Name&criteria=GPR180) | 1.63 | <0.0001 |
| A_23_P211603 | [A_23_P211603](http://genome-www4.stanford.edu/cgi-bin/SMD/source/sourceResult?choice=Gene&option=Name&criteria=A_23_P211603) | 1.53 | <0.0001 |
| A_23_P147106 | [FLJ20245](http://genome-www4.stanford.edu/cgi-bin/SMD/source/sourceResult?choice=Gene&option=Name&criteria=FLJ20245) | 1.80 | <0.0001 |
| A_23_P60627 | [ALOX15B](http://genome-www4.stanford.edu/cgi-bin/SMD/source/sourceResult?choice=Gene&option=Name&criteria=ALOX15B) | 2.37 | <0.0001 |
| A_24_P607195 | [A_24_P607195](http://genome-www4.stanford.edu/cgi-bin/SMD/source/sourceResult?choice=Gene&option=Name&criteria=A_24_P607195) | 1.56 | <0.0001 |
| A_32_P90615 | [A_32_P90615](http://genome-www4.stanford.edu/cgi-bin/SMD/source/sourceResult?choice=Gene&option=Name&criteria=A_32_P90615) | 1.83 | <0.0001 |
| A_24_P201973 | [TEP1](http://genome-www4.stanford.edu/cgi-bin/SMD/source/sourceResult?choice=Gene&option=Name&criteria=TEP1) | 2.14 | <0.0001 |
| A_23_P350895 | [RABGAP1](http://genome-www4.stanford.edu/cgi-bin/SMD/source/sourceResult?choice=Gene&option=Name&criteria=RABGAP1) | 1.99 | <0.0001 |
| A_32_P24741 | [AK093729](http://genome-www4.stanford.edu/cgi-bin/SMD/source/sourceResult?choice=Gene&option=Name&criteria=AK093729) | 1.73 | <0.0001 |
| A_24_P253755 | [PIGL](http://genome-www4.stanford.edu/cgi-bin/SMD/source/sourceResult?choice=Gene&option=Name&criteria=PIGL) | 1.57 | <0.0001 |
| A_32_P35603 | [THC2376568](http://genome-www4.stanford.edu/cgi-bin/SMD/source/sourceResult?choice=Gene&option=Name&criteria=THC2376568) | 1.95 | <0.0001 |
| A_32_P37143 | [GAS2L3](http://genome-www4.stanford.edu/cgi-bin/SMD/source/sourceResult?choice=Gene&option=Name&criteria=GAS2L3) | 1.68 | <0.0001 |
| A_32_P129310 | [THC2449838](http://genome-www4.stanford.edu/cgi-bin/SMD/source/sourceResult?choice=Gene&option=Name&criteria=THC2449838) | 1.85 | <0.0001 |
| A_23_P22499 | [GNL3L](http://genome-www4.stanford.edu/cgi-bin/SMD/source/sourceResult?choice=Gene&option=Name&criteria=GNL3L) | 1.85 | <0.0001 |
| A_23_P320728 | [A_23_P320728](http://genome-www4.stanford.edu/cgi-bin/SMD/source/sourceResult?choice=Gene&option=Name&criteria=A_23_P320728) | 1.79 | <0.0001 |
| A_32_P930953 | [AK024147](http://genome-www4.stanford.edu/cgi-bin/SMD/source/sourceResult?choice=Gene&option=Name&criteria=AK024147) | 1.81 | <0.0001 |
| A_32_P18300 | [A_32_P18300](http://genome-www4.stanford.edu/cgi-bin/SMD/source/sourceResult?choice=Gene&option=Name&criteria=A_32_P18300) | 1.95 | <0.0001 |
| A_24_P336853 | [LOC56902](http://genome-www4.stanford.edu/cgi-bin/SMD/source/sourceResult?choice=Gene&option=Name&criteria=LOC56902) | 1.76 | <0.0001 |
| A_23_P2294 | [HELB](http://genome-www4.stanford.edu/cgi-bin/SMD/source/sourceResult?choice=Gene&option=Name&criteria=HELB) | 1.70 | <0.0001 |
| A_24_P928415 | [VPS29](http://genome-www4.stanford.edu/cgi-bin/SMD/source/sourceResult?choice=Gene&option=Name&criteria=VPS29) | 1.92 | <0.0001 |
| A_23_P330561 | [MCEMP1](http://genome-www4.stanford.edu/cgi-bin/SMD/source/sourceResult?choice=Gene&option=Name&criteria=MCEMP1) | 1.68 | <0.0001 |
| A_23_P214066 | [ARHGAP26](http://genome-www4.stanford.edu/cgi-bin/SMD/source/sourceResult?choice=Gene&option=Name&criteria=ARHGAP26) | 2.03 | <0.0001 |
| A_23_P387691 | [ENST00000228360](http://genome-www4.stanford.edu/cgi-bin/SMD/source/sourceResult?choice=Gene&option=Name&criteria=ENST00000228360) | 1.88 | <0.0001 |
| A_23_P253738 | [CLN8](http://genome-www4.stanford.edu/cgi-bin/SMD/source/sourceResult?choice=Gene&option=Name&criteria=CLN8) | 1.53 | <0.0001 |
| A_23_P61180 | [PLCXD1](http://genome-www4.stanford.edu/cgi-bin/SMD/source/sourceResult?choice=Gene&option=Name&criteria=PLCXD1) | 1.79 | <0.0001 |
| A_24_P179175 | [PHC3](http://genome-www4.stanford.edu/cgi-bin/SMD/source/sourceResult?choice=Gene&option=Name&criteria=PHC3) | 2.07 | <0.0001 |
| A_23_P360754 | [ADAMTS4](http://genome-www4.stanford.edu/cgi-bin/SMD/source/sourceResult?choice=Gene&option=Name&criteria=ADAMTS4) | 1.88 | <0.0001 |
| A_32_P70158 | [LILRB3](http://genome-www4.stanford.edu/cgi-bin/SMD/source/sourceResult?choice=Gene&option=Name&criteria=LILRB3) | 2.04 | <0.0001 |
| A_23_P82651 | [NPTX2](http://genome-www4.stanford.edu/cgi-bin/SMD/source/sourceResult?choice=Gene&option=Name&criteria=NPTX2) | 1.71 | <0.0001 |
| A_24_P411561 | [HAVCR2](http://genome-www4.stanford.edu/cgi-bin/SMD/source/sourceResult?choice=Gene&option=Name&criteria=HAVCR2) | 1.90 | <0.0001 |
| A_24_P670342 | [A_24_P670342](http://genome-www4.stanford.edu/cgi-bin/SMD/source/sourceResult?choice=Gene&option=Name&criteria=A_24_P670342) | 1.67 | <0.0001 |
| A_32_P56874 | [BG502322](http://genome-www4.stanford.edu/cgi-bin/SMD/source/sourceResult?choice=Gene&option=Name&criteria=BG502322) | 1.68 | <0.0001 |
| A_32_P177595 | [A_32_P177595](http://genome-www4.stanford.edu/cgi-bin/SMD/source/sourceResult?choice=Gene&option=Name&criteria=A_32_P177595) | 1.68 | <0.0001 |
| A_23_P136173 | [CSF2RA](http://genome-www4.stanford.edu/cgi-bin/SMD/source/sourceResult?choice=Gene&option=Name&criteria=CSF2RA) | 1.81 | <0.0001 |
| A_32_P22679 | [A_32_P22679](http://genome-www4.stanford.edu/cgi-bin/SMD/source/sourceResult?choice=Gene&option=Name&criteria=A_32_P22679) | 1.61 | <0.0001 |
| A_23_P34510 | [PHC2](http://genome-www4.stanford.edu/cgi-bin/SMD/source/sourceResult?choice=Gene&option=Name&criteria=PHC2) | 1.68 | <0.0001 |
| A_23_P404595 | [ZNF417](http://genome-www4.stanford.edu/cgi-bin/SMD/source/sourceResult?choice=Gene&option=Name&criteria=ZNF417) | 1.73 | <0.0001 |
| A_24_P29665 | [CYCS](http://genome-www4.stanford.edu/cgi-bin/SMD/source/sourceResult?choice=Gene&option=Name&criteria=CYCS) | 2.20 | <0.0001 |
| A_32_P209909 | [BE614051](http://genome-www4.stanford.edu/cgi-bin/SMD/source/sourceResult?choice=Gene&option=Name&criteria=BE614051) | 1.64 | <0.0001 |
| A_24_P941773 | [METTL7A](http://genome-www4.stanford.edu/cgi-bin/SMD/source/sourceResult?choice=Gene&option=Name&criteria=METTL7A) | 2.47 | <0.0001 |
| A_24_P66337 | [LYCAT](http://genome-www4.stanford.edu/cgi-bin/SMD/source/sourceResult?choice=Gene&option=Name&criteria=LYCAT) | 1.82 | <0.0001 |
| A_32_P230465 | [AL133570](http://genome-www4.stanford.edu/cgi-bin/SMD/source/sourceResult?choice=Gene&option=Name&criteria=AL133570) | 1.92 | <0.0001 |
| A_23_P166408 | [OSM](http://genome-www4.stanford.edu/cgi-bin/SMD/source/sourceResult?choice=Gene&option=Name&criteria=OSM) | 2.07 | <0.0001 |
| A_24_P917951 | [NOC2L](http://genome-www4.stanford.edu/cgi-bin/SMD/source/sourceResult?choice=Gene&option=Name&criteria=NOC2L) | 1.72 | <0.0001 |
| A_32_P39963 | [EXOSC6](http://genome-www4.stanford.edu/cgi-bin/SMD/source/sourceResult?choice=Gene&option=Name&criteria=EXOSC6) | 1.76 | <0.0001 |
| A_23_P51926 | [PTAFR](http://genome-www4.stanford.edu/cgi-bin/SMD/source/sourceResult?choice=Gene&option=Name&criteria=PTAFR) | 1.74 | <0.0001 |
| A_24_P459522 | [AK000144](http://genome-www4.stanford.edu/cgi-bin/SMD/source/sourceResult?choice=Gene&option=Name&criteria=AK000144) | 1.73 | <0.0001 |
| A_23_P370574 | [KIR3DL3](http://genome-www4.stanford.edu/cgi-bin/SMD/source/sourceResult?choice=Gene&option=Name&criteria=KIR3DL3) | 1.51 | <0.0001 |
| A_23_P161698 | [MMP3](http://genome-www4.stanford.edu/cgi-bin/SMD/source/sourceResult?choice=Gene&option=Name&criteria=MMP3) | 2.05 | <0.0001 |
| A_23_P126782 | [F3](http://genome-www4.stanford.edu/cgi-bin/SMD/source/sourceResult?choice=Gene&option=Name&criteria=F3) | 1.67 | <0.0001 |
| A_24_P57730 | [MRPL52](http://genome-www4.stanford.edu/cgi-bin/SMD/source/sourceResult?choice=Gene&option=Name&criteria=MRPL52) | 1.65 | <0.0001 |
| A_23_P100341 | [ORC6L](http://genome-www4.stanford.edu/cgi-bin/SMD/source/sourceResult?choice=Gene&option=Name&criteria=ORC6L) | 1.50 | <0.0001 |
| A_24_P68162 | [DIP2B](http://genome-www4.stanford.edu/cgi-bin/SMD/source/sourceResult?choice=Gene&option=Name&criteria=DIP2B) | 2.01 | <0.0001 |
| A_23_P70359 | [C6orf59](http://genome-www4.stanford.edu/cgi-bin/SMD/source/sourceResult?choice=Gene&option=Name&criteria=C6orf59) | 1.73 | <0.0001 |
| A_24_P57170 | [LOC148137](http://genome-www4.stanford.edu/cgi-bin/SMD/source/sourceResult?choice=Gene&option=Name&criteria=LOC148137) | 1.51 | <0.0001 |
| A_24_P102821 | [PTAFR](http://genome-www4.stanford.edu/cgi-bin/SMD/source/sourceResult?choice=Gene&option=Name&criteria=PTAFR) | 1.52 | <0.0001 |
| A_24_P297098 | [PHF20](http://genome-www4.stanford.edu/cgi-bin/SMD/source/sourceResult?choice=Gene&option=Name&criteria=PHF20) | 1.62 | <0.0001 |
| A_23_P115246 | [FCN3](http://genome-www4.stanford.edu/cgi-bin/SMD/source/sourceResult?choice=Gene&option=Name&criteria=FCN3) | 1.51 | <0.0001 |
| A_24_P316495 | [BC033250](http://genome-www4.stanford.edu/cgi-bin/SMD/source/sourceResult?choice=Gene&option=Name&criteria=BC033250) | 1.70 | <0.0001 |
| A_24_P316454 | [BC022826](http://genome-www4.stanford.edu/cgi-bin/SMD/source/sourceResult?choice=Gene&option=Name&criteria=BC022826) | 2.45 | <0.0001 |
| A_24_P845082 | [AY090769](http://genome-www4.stanford.edu/cgi-bin/SMD/source/sourceResult?choice=Gene&option=Name&criteria=AY090769) | 1.58 | <0.0001 |
| A_32_P74942 | [BI024548](http://genome-www4.stanford.edu/cgi-bin/SMD/source/sourceResult?choice=Gene&option=Name&criteria=BI024548) | 1.81 | <0.0001 |
| A_23_P54597 | [RSL1D1](http://genome-www4.stanford.edu/cgi-bin/SMD/source/sourceResult?choice=Gene&option=Name&criteria=RSL1D1) | 1.58 | <0.0001 |
| A_23_P214267 | [GPR110](http://genome-www4.stanford.edu/cgi-bin/SMD/source/sourceResult?choice=Gene&option=Name&criteria=GPR110) | 2.51 | <0.0001 |
| A_24_P268474 | [A_24_P268474](http://genome-www4.stanford.edu/cgi-bin/SMD/source/sourceResult?choice=Gene&option=Name&criteria=A_24_P268474) | 1.56 | <0.0001 |
| A_23_P360777 | [NRG1](http://genome-www4.stanford.edu/cgi-bin/SMD/source/sourceResult?choice=Gene&option=Name&criteria=NRG1) | 1.84 | <0.0001 |
| A_23_P51966 | [A_23_P51966](http://genome-www4.stanford.edu/cgi-bin/SMD/source/sourceResult?choice=Gene&option=Name&criteria=A_23_P51966) | 1.65 | <0.0001 |
| A_24_P881430 | [AI216457](http://genome-www4.stanford.edu/cgi-bin/SMD/source/sourceResult?choice=Gene&option=Name&criteria=AI216457) | 1.69 | <0.0001 |
| A_24_P524452 | [HIST3H2BB](http://genome-www4.stanford.edu/cgi-bin/SMD/source/sourceResult?choice=Gene&option=Name&criteria=HIST3H2BB) | 1.58 | <0.0001 |
| A_23_P2293 | [HELB](http://genome-www4.stanford.edu/cgi-bin/SMD/source/sourceResult?choice=Gene&option=Name&criteria=HELB) | 1.80 | <0.0001 |
| A_24_P142305 | [HBA2](http://genome-www4.stanford.edu/cgi-bin/SMD/source/sourceResult?choice=Gene&option=Name&criteria=HBA2) | 2.31 | <0.0001 |
| A_32_P136588 | [BF928446](http://genome-www4.stanford.edu/cgi-bin/SMD/source/sourceResult?choice=Gene&option=Name&criteria=BF928446) | 1.58 | <0.0001 |
| A_23_P126457 | [ISG20L2](http://genome-www4.stanford.edu/cgi-bin/SMD/source/sourceResult?choice=Gene&option=Name&criteria=ISG20L2) | 1.58 | <0.0001 |
| A_32_P177937 | [ENST00000368918](http://genome-www4.stanford.edu/cgi-bin/SMD/source/sourceResult?choice=Gene&option=Name&criteria=ENST00000368918) | 1.80 | <0.0001 |
| A_23_P91390 | [THBD](http://genome-www4.stanford.edu/cgi-bin/SMD/source/sourceResult?choice=Gene&option=Name&criteria=THBD) | 1.99 | <0.0001 |
| A_24_P51279 | [AL833832](http://genome-www4.stanford.edu/cgi-bin/SMD/source/sourceResult?choice=Gene&option=Name&criteria=AL833832) | 1.77 | <0.0001 |
| A_32_P106944 | [ZNF429](http://genome-www4.stanford.edu/cgi-bin/SMD/source/sourceResult?choice=Gene&option=Name&criteria=ZNF429) | 1.70 | <0.0001 |
| A_24_P94034 | [USP22](http://genome-www4.stanford.edu/cgi-bin/SMD/source/sourceResult?choice=Gene&option=Name&criteria=USP22) | 1.57 | <0.0001 |
| A_32_P111658 | [N91552](http://genome-www4.stanford.edu/cgi-bin/SMD/source/sourceResult?choice=Gene&option=Name&criteria=N91552) | 1.51 | <0.0001 |
| A_32_P98574 | [A_32_P98574](http://genome-www4.stanford.edu/cgi-bin/SMD/source/sourceResult?choice=Gene&option=Name&criteria=A_32_P98574) | 1.95 | <0.0001 |
| A_23_P136849 | [THC2380706](http://genome-www4.stanford.edu/cgi-bin/SMD/source/sourceResult?choice=Gene&option=Name&criteria=THC2380706) | 1.67 | <0.0001 |
| A_23_P169437 | [LCN2](http://genome-www4.stanford.edu/cgi-bin/SMD/source/sourceResult?choice=Gene&option=Name&criteria=LCN2) | 2.44 | <0.0001 |
| A_24_P71280 | [GPR157](http://genome-www4.stanford.edu/cgi-bin/SMD/source/sourceResult?choice=Gene&option=Name&criteria=GPR157) | 1.52 | <0.0001 |
| A_23_P111206 | [FKBP5](http://genome-www4.stanford.edu/cgi-bin/SMD/source/sourceResult?choice=Gene&option=Name&criteria=FKBP5) | 1.95 | <0.0001 |
| A_23_P256158 | [ADRA2C](http://genome-www4.stanford.edu/cgi-bin/SMD/source/sourceResult?choice=Gene&option=Name&criteria=ADRA2C) | 1.70 | <0.0001 |
| A_23_P18055 | [C3orf51](http://genome-www4.stanford.edu/cgi-bin/SMD/source/sourceResult?choice=Gene&option=Name&criteria=C3orf51) | 1.52 | <0.0001 |
| A_32_P233713 | [A_32_P233713](http://genome-www4.stanford.edu/cgi-bin/SMD/source/sourceResult?choice=Gene&option=Name&criteria=A_32_P233713) | 1.87 | <0.0001 |
| A_24_P140171 | [CRTAP](http://genome-www4.stanford.edu/cgi-bin/SMD/source/sourceResult?choice=Gene&option=Name&criteria=CRTAP) | 1.93 | <0.0001 |
| A_32_P157671 | [A_32_P157671](http://genome-www4.stanford.edu/cgi-bin/SMD/source/sourceResult?choice=Gene&option=Name&criteria=A_32_P157671) | 2.08 | <0.0001 |
| A_23_P8754 | [AASS](http://genome-www4.stanford.edu/cgi-bin/SMD/source/sourceResult?choice=Gene&option=Name&criteria=AASS) | 1.52 | <0.0001 |
| A_32_P164916 | [AL833005](http://genome-www4.stanford.edu/cgi-bin/SMD/source/sourceResult?choice=Gene&option=Name&criteria=AL833005) | 2.35 | <0.0001 |
| A_23_P411296 | [CEBPB](http://genome-www4.stanford.edu/cgi-bin/SMD/source/sourceResult?choice=Gene&option=Name&criteria=CEBPB) | 1.54 | <0.0001 |
| A_32_P41375 | [AK093729](http://genome-www4.stanford.edu/cgi-bin/SMD/source/sourceResult?choice=Gene&option=Name&criteria=AK093729) | 1.72 | <0.0001 |
| A_23_P47220 | [A_23_P47220](http://genome-www4.stanford.edu/cgi-bin/SMD/source/sourceResult?choice=Gene&option=Name&criteria=A_23_P47220) | 1.54 | <0.0001 |
| A_23_P213562 | [F2R](http://genome-www4.stanford.edu/cgi-bin/SMD/source/sourceResult?choice=Gene&option=Name&criteria=F2R) | 1.74 | <0.0001 |
| A_24_P486503 | [ENST00000343567](http://genome-www4.stanford.edu/cgi-bin/SMD/source/sourceResult?choice=Gene&option=Name&criteria=ENST00000343567) | 1.59 | <0.0001 |
| A_23_P354170 | [PIGX](http://genome-www4.stanford.edu/cgi-bin/SMD/source/sourceResult?choice=Gene&option=Name&criteria=PIGX) | 1.55 | <0.0001 |
| A_24_P256674 | [ARHGEF10](http://genome-www4.stanford.edu/cgi-bin/SMD/source/sourceResult?choice=Gene&option=Name&criteria=ARHGEF10) | 1.59 | 0.0022 |
| A_23_P210465 | [PI3](http://genome-www4.stanford.edu/cgi-bin/SMD/source/sourceResult?choice=Gene&option=Name&criteria=PI3) | 1.86 | 0.0022 |
| A_24_P769977 | [THC2406011](http://genome-www4.stanford.edu/cgi-bin/SMD/source/sourceResult?choice=Gene&option=Name&criteria=THC2406011) | 1.84 | 0.0022 |
| A_23_P335920 | [RPS6KA2](http://genome-www4.stanford.edu/cgi-bin/SMD/source/sourceResult?choice=Gene&option=Name&criteria=RPS6KA2) | 1.54 | 0.0022 |
| A_32_P206561 | [THC2278097](http://genome-www4.stanford.edu/cgi-bin/SMD/source/sourceResult?choice=Gene&option=Name&criteria=THC2278097) | 1.61 | 0.0022 |
| A_23_P385529 | [POLR3E](http://genome-www4.stanford.edu/cgi-bin/SMD/source/sourceResult?choice=Gene&option=Name&criteria=POLR3E) | 1.75 | 0.0022 |
| A_23_P105815 | [BC008631](http://genome-www4.stanford.edu/cgi-bin/SMD/source/sourceResult?choice=Gene&option=Name&criteria=BC008631) | 1.67 | 0.0022 |
| A_23_P259071 | [AREG](http://genome-www4.stanford.edu/cgi-bin/SMD/source/sourceResult?choice=Gene&option=Name&criteria=AREG) | 2.40 | 0.0022 |
| A_24_P186030 | [PRKY](http://genome-www4.stanford.edu/cgi-bin/SMD/source/sourceResult?choice=Gene&option=Name&criteria=PRKY) | 1.57 | 0.0022 |
| A_24_P826046 | [AK024177](http://genome-www4.stanford.edu/cgi-bin/SMD/source/sourceResult?choice=Gene&option=Name&criteria=AK024177) | 1.54 | 0.0022 |
| A_32_P118010 | [THC2448349](http://genome-www4.stanford.edu/cgi-bin/SMD/source/sourceResult?choice=Gene&option=Name&criteria=THC2448349) | 1.70 | 0.0022 |
| A_32_P144599 | [AW979273](http://genome-www4.stanford.edu/cgi-bin/SMD/source/sourceResult?choice=Gene&option=Name&criteria=AW979273) | 1.83 | 0.0022 |
| A_24_P374863 | [RLTPR](http://genome-www4.stanford.edu/cgi-bin/SMD/source/sourceResult?choice=Gene&option=Name&criteria=RLTPR) | 1.96 | 0.0022 |
| A_32_P67577 | [THC2401493](http://genome-www4.stanford.edu/cgi-bin/SMD/source/sourceResult?choice=Gene&option=Name&criteria=THC2401493) | 1.64 | 0.0022 |
| A_23_P259314 | [RPS4Y1](http://genome-www4.stanford.edu/cgi-bin/SMD/source/sourceResult?choice=Gene&option=Name&criteria=RPS4Y1) | 2.96 | 0.0022 |
| A_23_P124252 | [CAMK1D](http://genome-www4.stanford.edu/cgi-bin/SMD/source/sourceResult?choice=Gene&option=Name&criteria=CAMK1D) | 1.69 | 0.0022 |
| A_32_P47166 | [A_32_P47166](http://genome-www4.stanford.edu/cgi-bin/SMD/source/sourceResult?choice=Gene&option=Name&criteria=A_32_P47166) | 1.79 | 0.0022 |
| A_32_P34167 | [A_32_P34167](http://genome-www4.stanford.edu/cgi-bin/SMD/source/sourceResult?choice=Gene&option=Name&criteria=A_32_P34167) | 1.70 | 0.0022 |
| A_23_P79398 | [IL1R2](http://genome-www4.stanford.edu/cgi-bin/SMD/source/sourceResult?choice=Gene&option=Name&criteria=IL1R2) | 2.57 | 0.0022 |
| A_24_P122636 | [BPNT1](http://genome-www4.stanford.edu/cgi-bin/SMD/source/sourceResult?choice=Gene&option=Name&criteria=BPNT1) | 1.53 | 0.0022 |
| A_24_P649357 | [SMA4](http://genome-www4.stanford.edu/cgi-bin/SMD/source/sourceResult?choice=Gene&option=Name&criteria=SMA4) | 1.78 | 0.0022 |
| A_24_P183150 | [CXCL3](http://genome-www4.stanford.edu/cgi-bin/SMD/source/sourceResult?choice=Gene&option=Name&criteria=CXCL3) | 2.08 | 0.0022 |
| A_23_P76450 | [PHLDA1](http://genome-www4.stanford.edu/cgi-bin/SMD/source/sourceResult?choice=Gene&option=Name&criteria=PHLDA1) | 1.89 | 0.0022 |
| A_24_P931628 | [AY358705](http://genome-www4.stanford.edu/cgi-bin/SMD/source/sourceResult?choice=Gene&option=Name&criteria=AY358705) | 1.59 | 0.0022 |
| A_32_P23209 | [THC2448537](http://genome-www4.stanford.edu/cgi-bin/SMD/source/sourceResult?choice=Gene&option=Name&criteria=THC2448537) | 1.60 | 0.0039 |
| A_24_P419017 | [AK098478](http://genome-www4.stanford.edu/cgi-bin/SMD/source/sourceResult?choice=Gene&option=Name&criteria=AK098478) | 1.95 | 0.0039 |
| A_23_P203558 | [HBB](http://genome-www4.stanford.edu/cgi-bin/SMD/source/sourceResult?choice=Gene&option=Name&criteria=HBB) | 2.61 | 0.0039 |
| A_23_P17706 | [IL17RA](http://genome-www4.stanford.edu/cgi-bin/SMD/source/sourceResult?choice=Gene&option=Name&criteria=IL17RA) | 1.55 | 0.0039 |
| A_24_P535380 | [AK025323](http://genome-www4.stanford.edu/cgi-bin/SMD/source/sourceResult?choice=Gene&option=Name&criteria=AK025323) | 1.59 | 0.0039 |
| A_23_P84860 | [FAM107A](http://genome-www4.stanford.edu/cgi-bin/SMD/source/sourceResult?choice=Gene&option=Name&criteria=FAM107A) | 1.79 | 0.0039 |
| A_24_P718672 | [AK024173](http://genome-www4.stanford.edu/cgi-bin/SMD/source/sourceResult?choice=Gene&option=Name&criteria=AK024173) | 1.78 | 0.0039 |
| A_23_P41824 | [A_23_P41824](http://genome-www4.stanford.edu/cgi-bin/SMD/source/sourceResult?choice=Gene&option=Name&criteria=A_23_P41824) | 1.57 | 0.0039 |
| A_23_P76435 | [15E1.2](http://genome-www4.stanford.edu/cgi-bin/SMD/source/sourceResult?choice=Gene&option=Name&criteria=15E1.2) | 1.53 | 0.0039 |
| A_23_P117602 | [GZMB](http://genome-www4.stanford.edu/cgi-bin/SMD/source/sourceResult?choice=Gene&option=Name&criteria=GZMB) | 1.70 | 0.0039 |
| A_23_P137856 | [MUC1](http://genome-www4.stanford.edu/cgi-bin/SMD/source/sourceResult?choice=Gene&option=Name&criteria=MUC1) | 2.05 | 0.0039 |
| A_23_P131834 | [THC2263651](http://genome-www4.stanford.edu/cgi-bin/SMD/source/sourceResult?choice=Gene&option=Name&criteria=THC2263651) | 1.85 | 0.0039 |
| A_23_P115167 | [LRRC8B](http://genome-www4.stanford.edu/cgi-bin/SMD/source/sourceResult?choice=Gene&option=Name&criteria=LRRC8B) | 1.70 | 0.0039 |
| A_32_P25065 | [A_32_P25065](http://genome-www4.stanford.edu/cgi-bin/SMD/source/sourceResult?choice=Gene&option=Name&criteria=A_32_P25065) | 1.74 | 0.0039 |
| A_23_P127584 | [NNMT](http://genome-www4.stanford.edu/cgi-bin/SMD/source/sourceResult?choice=Gene&option=Name&criteria=NNMT) | 1.90 | 0.0039 |
| A_24_P316234 | [AK090485](http://genome-www4.stanford.edu/cgi-bin/SMD/source/sourceResult?choice=Gene&option=Name&criteria=AK090485) | 1.63 | 0.0039 |
| A_24_P316414 | [LOC646990](http://genome-www4.stanford.edu/cgi-bin/SMD/source/sourceResult?choice=Gene&option=Name&criteria=LOC646990) | 1.51 | 0.0039 |
| A_23_P104798 | [IL18](http://genome-www4.stanford.edu/cgi-bin/SMD/source/sourceResult?choice=Gene&option=Name&criteria=IL18) | 1.56 | 0.0039 |
| A_23_P203882 | [MMP19](http://genome-www4.stanford.edu/cgi-bin/SMD/source/sourceResult?choice=Gene&option=Name&criteria=MMP19) | 1.91 | 0.0039 |
| A_24_P873659 | [MALAT1](http://genome-www4.stanford.edu/cgi-bin/SMD/source/sourceResult?choice=Gene&option=Name&criteria=MALAT1) | 2.18 | 0.0039 |
| A_24_P277367 | [CXCL5](http://genome-www4.stanford.edu/cgi-bin/SMD/source/sourceResult?choice=Gene&option=Name&criteria=CXCL5) | 1.55 | 0.0051 |
| A_32_P217750 | [IL3RA](http://genome-www4.stanford.edu/cgi-bin/SMD/source/sourceResult?choice=Gene&option=Name&criteria=IL3RA) | 1.77 | 0.0051 |
| A_32_P67447 | [THC2377365](http://genome-www4.stanford.edu/cgi-bin/SMD/source/sourceResult?choice=Gene&option=Name&criteria=THC2377365) | 1.57 | 0.0051 |
| A_23_P129903 | [TRIM16](http://genome-www4.stanford.edu/cgi-bin/SMD/source/sourceResult?choice=Gene&option=Name&criteria=TRIM16) | 1.57 | 0.0051 |
| A_24_P537188 | [BC032332](http://genome-www4.stanford.edu/cgi-bin/SMD/source/sourceResult?choice=Gene&option=Name&criteria=BC032332) | 1.61 | 0.0051 |
| A_24_P655888 | [A_24_P655888](http://genome-www4.stanford.edu/cgi-bin/SMD/source/sourceResult?choice=Gene&option=Name&criteria=A_24_P655888) | 2.00 | 0.0051 |
| A_24_P848352 | [THC2319114](http://genome-www4.stanford.edu/cgi-bin/SMD/source/sourceResult?choice=Gene&option=Name&criteria=THC2319114) | 1.54 | 0.0051 |
| A_24_P136155 | [A_24_P136155](http://genome-www4.stanford.edu/cgi-bin/SMD/source/sourceResult?choice=Gene&option=Name&criteria=A_24_P136155) | 1.60 | 0.0051 |
| A_23_P26457 | [HBA2](http://genome-www4.stanford.edu/cgi-bin/SMD/source/sourceResult?choice=Gene&option=Name&criteria=HBA2) | 1.93 | 0.0051 |
| A_24_P314597 | [KIAA0319L](http://genome-www4.stanford.edu/cgi-bin/SMD/source/sourceResult?choice=Gene&option=Name&criteria=KIAA0319L) | 1.53 | 0.0051 |
| A_23_P157628 | [DEFB4](http://genome-www4.stanford.edu/cgi-bin/SMD/source/sourceResult?choice=Gene&option=Name&criteria=DEFB4) | 1.62 | 0.0051 |
| A_24_P59387 | [C8orf60](http://genome-www4.stanford.edu/cgi-bin/SMD/source/sourceResult?choice=Gene&option=Name&criteria=C8orf60) | 1.54 | 0.0051 |
| A_32_P97547 | [AK027393](http://genome-www4.stanford.edu/cgi-bin/SMD/source/sourceResult?choice=Gene&option=Name&criteria=AK027393) | 1.55 | 0.0051 |
| A_23_P35995 | [ASAM](http://genome-www4.stanford.edu/cgi-bin/SMD/source/sourceResult?choice=Gene&option=Name&criteria=ASAM) | 1.85 | 0.0051 |
| A_23_P127948 | [ADM](http://genome-www4.stanford.edu/cgi-bin/SMD/source/sourceResult?choice=Gene&option=Name&criteria=ADM) | 1.67 | 0.0051 |
| A_23_P98580 | [FADS2](http://genome-www4.stanford.edu/cgi-bin/SMD/source/sourceResult?choice=Gene&option=Name&criteria=FADS2) | 1.72 | 0.0062 |
| A_24_P876772 | [BC032332](http://genome-www4.stanford.edu/cgi-bin/SMD/source/sourceResult?choice=Gene&option=Name&criteria=BC032332) | 1.59 | 0.0062 |
| A_24_P214556 | [THC2380706](http://genome-www4.stanford.edu/cgi-bin/SMD/source/sourceResult?choice=Gene&option=Name&criteria=THC2380706) | 1.58 | 0.0062 |
| A_23_P37317 | [AF147723](http://genome-www4.stanford.edu/cgi-bin/SMD/source/sourceResult?choice=Gene&option=Name&criteria=AF147723) | 1.54 | 0.0062 |
| A_32_P27698 | [FLJ44060](http://genome-www4.stanford.edu/cgi-bin/SMD/source/sourceResult?choice=Gene&option=Name&criteria=FLJ44060) | 1.61 | 0.0062 |
| A_23_P7144 | [CXCL1](http://genome-www4.stanford.edu/cgi-bin/SMD/source/sourceResult?choice=Gene&option=Name&criteria=CXCL1) | 2.23 | 0.0062 |
| A_23_P119583 | [PDE4C](http://genome-www4.stanford.edu/cgi-bin/SMD/source/sourceResult?choice=Gene&option=Name&criteria=PDE4C) | 1.50 | 0.0062 |
| A_32_P132883 | [THC2282416](http://genome-www4.stanford.edu/cgi-bin/SMD/source/sourceResult?choice=Gene&option=Name&criteria=THC2282416) | 1.68 | 0.0062 |
| A_24_P75190 | [HBD](http://genome-www4.stanford.edu/cgi-bin/SMD/source/sourceResult?choice=Gene&option=Name&criteria=HBD) | 2.35 | 0.0062 |
| A_24_P683917 | [FLNB](http://genome-www4.stanford.edu/cgi-bin/SMD/source/sourceResult?choice=Gene&option=Name&criteria=FLNB) | 1.61 | 0.0062 |
| A_24_P157087 | [CASP8](http://genome-www4.stanford.edu/cgi-bin/SMD/source/sourceResult?choice=Gene&option=Name&criteria=CASP8) | 2.06 | 0.0062 |
| A_32_P191074 | [THC2394812](http://genome-www4.stanford.edu/cgi-bin/SMD/source/sourceResult?choice=Gene&option=Name&criteria=THC2394812) | 1.56 | 0.0062 |
| A_23_P41917 | [HOMER1](http://genome-www4.stanford.edu/cgi-bin/SMD/source/sourceResult?choice=Gene&option=Name&criteria=HOMER1) | 1.58 | 0.0080 |
| A_24_P24565 | [THC2263894](http://genome-www4.stanford.edu/cgi-bin/SMD/source/sourceResult?choice=Gene&option=Name&criteria=THC2263894) | 1.92 | 0.0080 |
| A_24_P329795 | [C10orf10](http://genome-www4.stanford.edu/cgi-bin/SMD/source/sourceResult?choice=Gene&option=Name&criteria=C10orf10) | 1.94 | 0.0080 |
| A_24_P941309 | [ZNF224](http://genome-www4.stanford.edu/cgi-bin/SMD/source/sourceResult?choice=Gene&option=Name&criteria=ZNF224) | 1.76 | 0.0080 |
| A_24_P856722 | [AI791206](http://genome-www4.stanford.edu/cgi-bin/SMD/source/sourceResult?choice=Gene&option=Name&criteria=AI791206) | 1.72 | 0.0080 |
| A_32_P203615 | [SUZ12P](http://genome-www4.stanford.edu/cgi-bin/SMD/source/sourceResult?choice=Gene&option=Name&criteria=SUZ12P) | 1.65 | 0.0080 |
| A_24_P936171 | [AGMAT](http://genome-www4.stanford.edu/cgi-bin/SMD/source/sourceResult?choice=Gene&option=Name&criteria=AGMAT) | 1.71 | 0.0080 |
| A_23_P3532 | [LITAF](http://genome-www4.stanford.edu/cgi-bin/SMD/source/sourceResult?choice=Gene&option=Name&criteria=LITAF) | 1.74 | 0.0080 |
| A_24_P829934 | [THC2341944](http://genome-www4.stanford.edu/cgi-bin/SMD/source/sourceResult?choice=Gene&option=Name&criteria=THC2341944) | 1.53 | 0.0080 |
| A_23_P37856 | [HBA1](http://genome-www4.stanford.edu/cgi-bin/SMD/source/sourceResult?choice=Gene&option=Name&criteria=HBA1) | 1.81 | 0.0080 |
| A_32_P118013 | [THC2448349](http://genome-www4.stanford.edu/cgi-bin/SMD/source/sourceResult?choice=Gene&option=Name&criteria=THC2448349) | 1.88 | 0.0080 |
| A_23_P203419 | [FADS1](http://genome-www4.stanford.edu/cgi-bin/SMD/source/sourceResult?choice=Gene&option=Name&criteria=FADS1) | 1.68 | 0.0080 |
| A_24_P70480 | [CEACAM4](http://genome-www4.stanford.edu/cgi-bin/SMD/source/sourceResult?choice=Gene&option=Name&criteria=CEACAM4) | 1.65 | 0.0080 |
| A_32_P50406 | [AK093729](http://genome-www4.stanford.edu/cgi-bin/SMD/source/sourceResult?choice=Gene&option=Name&criteria=AK093729) | 1.61 | 0.0080 |
| A_23_P211039 | [ADAMTS1](http://genome-www4.stanford.edu/cgi-bin/SMD/source/sourceResult?choice=Gene&option=Name&criteria=ADAMTS1) | 1.81 | 0.0080 |
| A_32_P151782 | [A_32_P151782](http://genome-www4.stanford.edu/cgi-bin/SMD/source/sourceResult?choice=Gene&option=Name&criteria=A_32_P151782) | 1.59 | 0.0098 |
| A_23_P127663 | [PRRG4](http://genome-www4.stanford.edu/cgi-bin/SMD/source/sourceResult?choice=Gene&option=Name&criteria=PRRG4) | 1.56 | 0.0098 |
| A_23_P1691 | [MMP1](http://genome-www4.stanford.edu/cgi-bin/SMD/source/sourceResult?choice=Gene&option=Name&criteria=MMP1) | 2.03 | 0.0098 |
| A_32_P69386 | [A_32_P69386](http://genome-www4.stanford.edu/cgi-bin/SMD/source/sourceResult?choice=Gene&option=Name&criteria=A_32_P69386) | 1.52 | 0.0098 |
| A_23_P356581 | [ROBO3](http://genome-www4.stanford.edu/cgi-bin/SMD/source/sourceResult?choice=Gene&option=Name&criteria=ROBO3) | 1.52 | 0.0098 |
| A_24_P53976 | [GLUL](http://genome-www4.stanford.edu/cgi-bin/SMD/source/sourceResult?choice=Gene&option=Name&criteria=GLUL) | 1.56 | 0.0098 |
| A_24_P33982 | [ENST00000332935](http://genome-www4.stanford.edu/cgi-bin/SMD/source/sourceResult?choice=Gene&option=Name&criteria=ENST00000332935) | 1.54 | 0.0098 |
| A_32_P215143 | [LOC647022](http://genome-www4.stanford.edu/cgi-bin/SMD/source/sourceResult?choice=Gene&option=Name&criteria=LOC647022) | 2.19 | 0.0098 |
| A_32_P191541 | [THC2345392](http://genome-www4.stanford.edu/cgi-bin/SMD/source/sourceResult?choice=Gene&option=Name&criteria=THC2345392) | 1.96 | 0.0098 |
| A_23_P133036 | [SLC34A2](http://genome-www4.stanford.edu/cgi-bin/SMD/source/sourceResult?choice=Gene&option=Name&criteria=SLC34A2) | 1.63 | 0.0124 |
| A_24_P78531 | [CLEC4E](http://genome-www4.stanford.edu/cgi-bin/SMD/source/sourceResult?choice=Gene&option=Name&criteria=CLEC4E) | 1.79 | 0.0124 |
| A_32_P113404 | [A_32_P113404](http://genome-www4.stanford.edu/cgi-bin/SMD/source/sourceResult?choice=Gene&option=Name&criteria=A_32_P113404) | 1.58 | 0.0124 |
| A_23_P20122 | [ZC3HAV1](http://genome-www4.stanford.edu/cgi-bin/SMD/source/sourceResult?choice=Gene&option=Name&criteria=ZC3HAV1) | 1.52 | 0.0124 |
| A_23_P316410 | [NOX1](http://genome-www4.stanford.edu/cgi-bin/SMD/source/sourceResult?choice=Gene&option=Name&criteria=NOX1) | 1.89 | 0.0124 |
| A_23_P38795 | [FPR1](http://genome-www4.stanford.edu/cgi-bin/SMD/source/sourceResult?choice=Gene&option=Name&criteria=FPR1) | 1.73 | 0.0124 |
| A_24_P136124 | [AF258585](http://genome-www4.stanford.edu/cgi-bin/SMD/source/sourceResult?choice=Gene&option=Name&criteria=AF258585) | 1.56 | 0.0124 |
| A_23_P16225 | [VMD2L1](http://genome-www4.stanford.edu/cgi-bin/SMD/source/sourceResult?choice=Gene&option=Name&criteria=VMD2L1) | 1.99 | 0.0124 |
| A_23_P170908 | [NUBPL](http://genome-www4.stanford.edu/cgi-bin/SMD/source/sourceResult?choice=Gene&option=Name&criteria=NUBPL) | 1.50 | 0.0124 |
| A_24_P341489 | [A_24_P341489](http://genome-www4.stanford.edu/cgi-bin/SMD/source/sourceResult?choice=Gene&option=Name&criteria=A_24_P341489) | 1.52 | 0.0124 |
| A_23_P253661 | [FLJ13236](http://genome-www4.stanford.edu/cgi-bin/SMD/source/sourceResult?choice=Gene&option=Name&criteria=FLJ13236) | 1.59 | 0.0124 |
| A_23_P215956 | [MYC](http://genome-www4.stanford.edu/cgi-bin/SMD/source/sourceResult?choice=Gene&option=Name&criteria=MYC) | 1.57 | 0.0124 |
| A_24_P38081 | [FKBP5](http://genome-www4.stanford.edu/cgi-bin/SMD/source/sourceResult?choice=Gene&option=Name&criteria=FKBP5) | 1.75 | 0.0161 |
| A_23_P71379 | [PSCA](http://genome-www4.stanford.edu/cgi-bin/SMD/source/sourceResult?choice=Gene&option=Name&criteria=PSCA) | 2.12 | 0.0161 |
| A_24_P485105 | [THC2288214](http://genome-www4.stanford.edu/cgi-bin/SMD/source/sourceResult?choice=Gene&option=Name&criteria=THC2288214) | 1.77 | 0.0161 |
| A_32_P164246 | [FOXQ1](http://genome-www4.stanford.edu/cgi-bin/SMD/source/sourceResult?choice=Gene&option=Name&criteria=FOXQ1) | 1.70 | 0.0161 |
| A_32_P184916 | [GNB4](http://genome-www4.stanford.edu/cgi-bin/SMD/source/sourceResult?choice=Gene&option=Name&criteria=GNB4) | 1.50 | 0.0161 |
| A_23_P119353 | [RASIP1](http://genome-www4.stanford.edu/cgi-bin/SMD/source/sourceResult?choice=Gene&option=Name&criteria=RASIP1) | 1.62 | 0.0161 |
| A_24_P942703 | [FLJ11903](http://genome-www4.stanford.edu/cgi-bin/SMD/source/sourceResult?choice=Gene&option=Name&criteria=FLJ11903) | 1.70 | 0.0161 |
| A_23_P342275 | [ADAMTS1](http://genome-www4.stanford.edu/cgi-bin/SMD/source/sourceResult?choice=Gene&option=Name&criteria=ADAMTS1) | 1.75 | 0.0161 |
| A_23_P63390 | [LOC440607](http://genome-www4.stanford.edu/cgi-bin/SMD/source/sourceResult?choice=Gene&option=Name&criteria=LOC440607) | 1.80 | 0.0161 |
| A_32_P139196 | [C13orf25](http://genome-www4.stanford.edu/cgi-bin/SMD/source/sourceResult?choice=Gene&option=Name&criteria=C13orf25) | 1.51 | 0.0161 |
| A_23_P153320 | [ICAM1](http://genome-www4.stanford.edu/cgi-bin/SMD/source/sourceResult?choice=Gene&option=Name&criteria=ICAM1) | 1.64 | 0.0161 |
| A_32_P703 | [LOC646626](http://genome-www4.stanford.edu/cgi-bin/SMD/source/sourceResult?choice=Gene&option=Name&criteria=LOC646626) | 1.64 | 0.0161 |
| A_23_P200728 | [FCGR3A](http://genome-www4.stanford.edu/cgi-bin/SMD/source/sourceResult?choice=Gene&option=Name&criteria=FCGR3A) | 1.83 | 0.0161 |
| A_24_P453740 | [DNAJA5](http://genome-www4.stanford.edu/cgi-bin/SMD/source/sourceResult?choice=Gene&option=Name&criteria=DNAJA5) | 1.66 | 0.0161 |
| A_32_P28712 | [THC2441398](http://genome-www4.stanford.edu/cgi-bin/SMD/source/sourceResult?choice=Gene&option=Name&criteria=THC2441398) | 1.69 | 0.0161 |
| A_23_P153616 | [MADCAM1](http://genome-www4.stanford.edu/cgi-bin/SMD/source/sourceResult?choice=Gene&option=Name&criteria=MADCAM1) | 1.56 | 0.0161 |
| A_24_P282083 | [AK097085](http://genome-www4.stanford.edu/cgi-bin/SMD/source/sourceResult?choice=Gene&option=Name&criteria=AK097085) | 1.60 | 0.0161 |
| A_23_P315364 | [CXCL2](http://genome-www4.stanford.edu/cgi-bin/SMD/source/sourceResult?choice=Gene&option=Name&criteria=CXCL2) | 2.09 | 0.0204 |
| A_23_P63209 | [HSD11B1](http://genome-www4.stanford.edu/cgi-bin/SMD/source/sourceResult?choice=Gene&option=Name&criteria=HSD11B1) | 1.54 | 0.0204 |
| A_23_P143526 | [S100B](http://genome-www4.stanford.edu/cgi-bin/SMD/source/sourceResult?choice=Gene&option=Name&criteria=S100B) | 1.64 | 0.0204 |
| A_24_P257416 | [CXCL2](http://genome-www4.stanford.edu/cgi-bin/SMD/source/sourceResult?choice=Gene&option=Name&criteria=CXCL2) | 1.64 | 0.0204 |
| A_23_P145204 | [HFE](http://genome-www4.stanford.edu/cgi-bin/SMD/source/sourceResult?choice=Gene&option=Name&criteria=HFE) | 1.62 | 0.0204 |
| A_32_P12372 | [THC2355348](http://genome-www4.stanford.edu/cgi-bin/SMD/source/sourceResult?choice=Gene&option=Name&criteria=THC2355348) | 1.54 | 0.0204 |
| A_24_P335092 | [SAA1](http://genome-www4.stanford.edu/cgi-bin/SMD/source/sourceResult?choice=Gene&option=Name&criteria=SAA1) | 1.64 | 0.0204 |
| A_32_P164917 | [AL833005](http://genome-www4.stanford.edu/cgi-bin/SMD/source/sourceResult?choice=Gene&option=Name&criteria=AL833005) | 2.00 | 0.0204 |
| A_24_P830025 | [FNTA](http://genome-www4.stanford.edu/cgi-bin/SMD/source/sourceResult?choice=Gene&option=Name&criteria=FNTA) | 1.52 | 0.0204 |
| A_32_P219704 | [A_32_P219704](http://genome-www4.stanford.edu/cgi-bin/SMD/source/sourceResult?choice=Gene&option=Name&criteria=A_32_P219704) | 1.68 | 0.0204 |
| A_23_P13753 | [NFE2](http://genome-www4.stanford.edu/cgi-bin/SMD/source/sourceResult?choice=Gene&option=Name&criteria=NFE2) | 1.73 | 0.0204 |
| A_23_P104804 | [ZBTB16](http://genome-www4.stanford.edu/cgi-bin/SMD/source/sourceResult?choice=Gene&option=Name&criteria=ZBTB16) | 1.71 | 0.0242 |
| A_24_P147849 | [A_24_P147849](http://genome-www4.stanford.edu/cgi-bin/SMD/source/sourceResult?choice=Gene&option=Name&criteria=A_24_P147849) | 1.66 | 0.0242 |
| A_24_P131622 | [FAM107A](http://genome-www4.stanford.edu/cgi-bin/SMD/source/sourceResult?choice=Gene&option=Name&criteria=FAM107A) | 1.57 | 0.0242 |
| A_24_P273523 | AK097965 | 1.59 | 0.0242 |
| A_32_P161855 | [KIAA1199](http://genome-www4.stanford.edu/cgi-bin/SMD/source/sourceResult?choice=Gene&option=Name&criteria=KIAA1199) | 1.68 | 0.0242 |
| A_32_P195788 | [BX104999](http://genome-www4.stanford.edu/cgi-bin/SMD/source/sourceResult?choice=Gene&option=Name&criteria=BX104999) | 1.52 | 0.0242 |
| A_24_P152743 | [TMC6](http://genome-www4.stanford.edu/cgi-bin/SMD/source/sourceResult?choice=Gene&option=Name&criteria=TMC6) | 1.56 | 0.0242 |
| A_23_P62115 | [TIMP1](http://genome-www4.stanford.edu/cgi-bin/SMD/source/sourceResult?choice=Gene&option=Name&criteria=TIMP1) | 1.58 | 0.0242 |
| A_23_P402164 | [LOC142937](http://genome-www4.stanford.edu/cgi-bin/SMD/source/sourceResult?choice=Gene&option=Name&criteria=LOC142937) | 1.59 | 0.0242 |
| A_23_P18406 | [CAMK2N2](http://genome-www4.stanford.edu/cgi-bin/SMD/source/sourceResult?choice=Gene&option=Name&criteria=CAMK2N2) | 1.51 | 0.0242 |
| A_24_P697437 | [THC2309258](http://genome-www4.stanford.edu/cgi-bin/SMD/source/sourceResult?choice=Gene&option=Name&criteria=THC2309258) | 1.66 | 0.0242 |
| A_24_P319369 | [F11R](http://genome-www4.stanford.edu/cgi-bin/SMD/source/sourceResult?choice=Gene&option=Name&criteria=F11R) | 1.62 | 0.0242 |
| A_23_P324384 | [RPS4Y2](http://genome-www4.stanford.edu/cgi-bin/SMD/source/sourceResult?choice=Gene&option=Name&criteria=RPS4Y2) | 2.29 | 0.0296 |
| A_24_P323916 | [A_24_P323916](http://genome-www4.stanford.edu/cgi-bin/SMD/source/sourceResult?choice=Gene&option=Name&criteria=A_24_P323916) | 1.54 | 0.0296 |
| A_23_P503072 | [CCL28](http://genome-www4.stanford.edu/cgi-bin/SMD/source/sourceResult?choice=Gene&option=Name&criteria=CCL28) | 1.72 | 0.0296 |
| A_23_P24104 | [PLAU](http://genome-www4.stanford.edu/cgi-bin/SMD/source/sourceResult?choice=Gene&option=Name&criteria=PLAU) | 1.52 | 0.0296 |
| A_23_P119196 | [KLF2](http://genome-www4.stanford.edu/cgi-bin/SMD/source/sourceResult?choice=Gene&option=Name&criteria=KLF2) | 1.64 | 0.0296 |
| A_23_P206899 | [TMEM159](http://genome-www4.stanford.edu/cgi-bin/SMD/source/sourceResult?choice=Gene&option=Name&criteria=TMEM159) | 1.53 | 0.0296 |
| A_24_P945165 | [ENST00000371316](http://genome-www4.stanford.edu/cgi-bin/SMD/source/sourceResult?choice=Gene&option=Name&criteria=ENST00000371316) | 1.61 | 0.0296 |
| A_32_P198791 | [THC2397265](http://genome-www4.stanford.edu/cgi-bin/SMD/source/sourceResult?choice=Gene&option=Name&criteria=THC2397265) | 1.57 | 0.0296 |
| A_24_P936911 | [THC2337994](http://genome-www4.stanford.edu/cgi-bin/SMD/source/sourceResult?choice=Gene&option=Name&criteria=THC2337994) | 1.53 | 0.0296 |
| A_24_P934594 | [AKAP8L](http://genome-www4.stanford.edu/cgi-bin/SMD/source/sourceResult?choice=Gene&option=Name&criteria=AKAP8L) | 1.52 | 0.0296 |
| A_24_P176805 | [ZNF264](http://genome-www4.stanford.edu/cgi-bin/SMD/source/sourceResult?choice=Gene&option=Name&criteria=ZNF264) | 1.69 | 0.0296 |
| A_23_P55256 | [ZNF652](http://genome-www4.stanford.edu/cgi-bin/SMD/source/sourceResult?choice=Gene&option=Name&criteria=ZNF652) | 1.59 | 0.0296 |
| A_23_P97517 | [RALGPS2](http://genome-www4.stanford.edu/cgi-bin/SMD/source/sourceResult?choice=Gene&option=Name&criteria=RALGPS2) | 1.59 | 0.0296 |
| A_23_P212779 | [DKFZP564O0823](http://genome-www4.stanford.edu/cgi-bin/SMD/source/sourceResult?choice=Gene&option=Name&criteria=DKFZP564O0823) | 1.70 | 0.0296 |
| A_32_P71310 | [AK093532](http://genome-www4.stanford.edu/cgi-bin/SMD/source/sourceResult?choice=Gene&option=Name&criteria=AK093532) | 1.66 | 0.0296 |
| A_23_P16673 | [CNN2](http://genome-www4.stanford.edu/cgi-bin/SMD/source/sourceResult?choice=Gene&option=Name&criteria=CNN2) | 1.57 | 0.0296 |
| A_23_P207476 | [SPAG9](http://genome-www4.stanford.edu/cgi-bin/SMD/source/sourceResult?choice=Gene&option=Name&criteria=SPAG9) | 1.52 | 0.0364 |
| A_23_P30126 | [FGFBP1](http://genome-www4.stanford.edu/cgi-bin/SMD/source/sourceResult?choice=Gene&option=Name&criteria=FGFBP1) | 2.30 | 0.0364 |
| A_23_P304921 | [NOX1](http://genome-www4.stanford.edu/cgi-bin/SMD/source/sourceResult?choice=Gene&option=Name&criteria=NOX1) | 2.22 | 0.0364 |
| A_24_P79403 | [PF4](http://genome-www4.stanford.edu/cgi-bin/SMD/source/sourceResult?choice=Gene&option=Name&criteria=PF4) | 1.58 | 0.0364 |
| A_23_P55270 | [CCL18](http://genome-www4.stanford.edu/cgi-bin/SMD/source/sourceResult?choice=Gene&option=Name&criteria=CCL18) | 1.82 | 0.0364 |
| A_24_P387321 | [ZNF44](http://genome-www4.stanford.edu/cgi-bin/SMD/source/sourceResult?choice=Gene&option=Name&criteria=ZNF44) | 1.51 | 0.0364 |
| A_24_P299685 | [PDPN](http://genome-www4.stanford.edu/cgi-bin/SMD/source/sourceResult?choice=Gene&option=Name&criteria=PDPN) | 1.57 | 0.0364 |
| A_32_P38323 | [SERPINB9](http://genome-www4.stanford.edu/cgi-bin/SMD/source/sourceResult?choice=Gene&option=Name&criteria=SERPINB9) | 1.86 | 0.0364 |
| A_24_P8524 | [AF119913](http://genome-www4.stanford.edu/cgi-bin/SMD/source/sourceResult?choice=Gene&option=Name&criteria=AF119913) | 1.51 | 0.0364 |
| A_23_P168916 | [CA1](http://genome-www4.stanford.edu/cgi-bin/SMD/source/sourceResult?choice=Gene&option=Name&criteria=CA1) | 2.13 | 0.0364 |
| A_23_P388553 | [ZKSCAN1](http://genome-www4.stanford.edu/cgi-bin/SMD/source/sourceResult?choice=Gene&option=Name&criteria=ZKSCAN1) | 1.60 | 0.0364 |
| A_32_P68142 | [AI470277](http://genome-www4.stanford.edu/cgi-bin/SMD/source/sourceResult?choice=Gene&option=Name&criteria=AI470277) | 1.54 | 0.0364 |
| A_24_P114255 | [MBOAT2](http://genome-www4.stanford.edu/cgi-bin/SMD/source/sourceResult?choice=Gene&option=Name&criteria=MBOAT2) | 1.63 | 0.0364 |
| A_32_P44808 | [AK124941](http://genome-www4.stanford.edu/cgi-bin/SMD/source/sourceResult?choice=Gene&option=Name&criteria=AK124941) | 1.53 | 0.0364 |
| A_23_P362415 | [UBE2B](http://genome-www4.stanford.edu/cgi-bin/SMD/source/sourceResult?choice=Gene&option=Name&criteria=UBE2B) | 1.53 | 0.0405 |
| A_23_P29096 | [PDE9A](http://genome-www4.stanford.edu/cgi-bin/SMD/source/sourceResult?choice=Gene&option=Name&criteria=PDE9A) | 1.69 | 0.0405 |
| A_23_P207850 | [TNS4](http://genome-www4.stanford.edu/cgi-bin/SMD/source/sourceResult?choice=Gene&option=Name&criteria=TNS4) | 1.67 | 0.0405 |
| A_23_P45751 | [CLCA4](http://genome-www4.stanford.edu/cgi-bin/SMD/source/sourceResult?choice=Gene&option=Name&criteria=CLCA4) | 1.77 | 0.0405 |
| A_23_P56938 | [REL](http://genome-www4.stanford.edu/cgi-bin/SMD/source/sourceResult?choice=Gene&option=Name&criteria=REL) | 1.60 | 0.0405 |
| A_24_P931859 | [AK074662](http://genome-www4.stanford.edu/cgi-bin/SMD/source/sourceResult?choice=Gene&option=Name&criteria=AK074662) | 1.58 | 0.0405 |
| A_24_P65597 | [CR616003](http://genome-www4.stanford.edu/cgi-bin/SMD/source/sourceResult?choice=Gene&option=Name&criteria=CR616003) | 1.56 | 0.0405 |
| A_23_P120354 | [ANKRD57](http://genome-www4.stanford.edu/cgi-bin/SMD/source/sourceResult?choice=Gene&option=Name&criteria=ANKRD57) | 1.55 | 0.0405 |
| A_23_P380857 | [APOL4](http://genome-www4.stanford.edu/cgi-bin/SMD/source/sourceResult?choice=Gene&option=Name&criteria=APOL4) | 1.53 | 0.0405 |
| A_32_P26422 | [THC2349739](http://genome-www4.stanford.edu/cgi-bin/SMD/source/sourceResult?choice=Gene&option=Name&criteria=THC2349739) | 1.52 | 0.0405 |
| A_24_P27373 | [PLDN](http://genome-www4.stanford.edu/cgi-bin/SMD/source/sourceResult?choice=Gene&option=Name&criteria=PLDN) | 1.61 | 0.0405 |
| A_23_P254741 | [SOD3](http://genome-www4.stanford.edu/cgi-bin/SMD/source/sourceResult?choice=Gene&option=Name&criteria=SOD3) | 1.58 | 0.0447 |
| A_32_P110086 | [A_32_P110086](http://genome-www4.stanford.edu/cgi-bin/SMD/source/sourceResult?choice=Gene&option=Name&criteria=A_32_P110086) | 1.52 | 0.0447 |
| A_23_P120973 | [FAM118A](http://genome-www4.stanford.edu/cgi-bin/SMD/source/sourceResult?choice=Gene&option=Name&criteria=FAM118A) | 1.66 | 0.0447 |
| A_23_P147109 | [FLJ20245](http://genome-www4.stanford.edu/cgi-bin/SMD/source/sourceResult?choice=Gene&option=Name&criteria=FLJ20245) | 1.67 | 0.0447 |
| A_24_P65098 | [TMEM87A](http://genome-www4.stanford.edu/cgi-bin/SMD/source/sourceResult?choice=Gene&option=Name&criteria=TMEM87A) | 1.64 | 0.0447 |
| A_24_P12438 | [NCOA7](http://genome-www4.stanford.edu/cgi-bin/SMD/source/sourceResult?choice=Gene&option=Name&criteria=NCOA7) | 1.57 | 0.0447 |
| A_23_P85250 | [CD24](http://genome-www4.stanford.edu/cgi-bin/SMD/source/sourceResult?choice=Gene&option=Name&criteria=CD24) | 2.07 | 0.0447 |
| A_24_P191781 | [DKFZP564O0823](http://genome-www4.stanford.edu/cgi-bin/SMD/source/sourceResult?choice=Gene&option=Name&criteria=DKFZP564O0823) | 1.68 | 0.0447 |
| A_23_P571 | [SLC2A1](http://genome-www4.stanford.edu/cgi-bin/SMD/source/sourceResult?choice=Gene&option=Name&criteria=SLC2A1) | 1.56 | 0.0447 |
| A_24_P51375 | [AF351612](http://genome-www4.stanford.edu/cgi-bin/SMD/source/sourceResult?choice=Gene&option=Name&criteria=AF351612) | 1.57 | 0.0447 |
| A_23_P127288 | [IL2RA](http://genome-www4.stanford.edu/cgi-bin/SMD/source/sourceResult?choice=Gene&option=Name&criteria=IL2RA) | 1.71 | 0.0447 |
| A_24_P225679 | [IRS1](http://genome-www4.stanford.edu/cgi-bin/SMD/source/sourceResult?choice=Gene&option=Name&criteria=IRS1) | 1.58 | 0.0477 |
| A_24_P196878 | [AF289567](http://genome-www4.stanford.edu/cgi-bin/SMD/source/sourceResult?choice=Gene&option=Name&criteria=AF289567) | 1.69 | 0.0477 |
| A_23_P217280 | [NOX1](http://genome-www4.stanford.edu/cgi-bin/SMD/source/sourceResult?choice=Gene&option=Name&criteria=NOX1) | 1.96 | 0.0477 |
| A_23_P153256 | [MGC4728](http://genome-www4.stanford.edu/cgi-bin/SMD/source/sourceResult?choice=Gene&option=Name&criteria=MGC4728) | 1.52 | 0.0477 |
| A_32_P196263 | [ADAMTS9](http://genome-www4.stanford.edu/cgi-bin/SMD/source/sourceResult?choice=Gene&option=Name&criteria=ADAMTS9) | 1.54 | 0.0477 |
| A_23_P343221 | [LILRB1](http://genome-www4.stanford.edu/cgi-bin/SMD/source/sourceResult?choice=Gene&option=Name&criteria=LILRB1) | 1.78 | 0.0477 |
| A_24_P945113 | [CR596233](http://genome-www4.stanford.edu/cgi-bin/SMD/source/sourceResult?choice=Gene&option=Name&criteria=CR596233) | 1.62 | 0.0477 |
| A_23_P211468 | [AA837799](http://genome-www4.stanford.edu/cgi-bin/SMD/source/sourceResult?choice=Gene&option=Name&criteria=AA837799) | 1.70 | 0.0477 |
| A_32_P108254 | [FAM20A](http://genome-www4.stanford.edu/cgi-bin/SMD/source/sourceResult?choice=Gene&option=Name&criteria=FAM20A) | 1.52 | 0.0477 |
|  |  |  |  |
| **Supplementary Table S1D. Gene-probes downregulated in UC compared to Control (n=678)** | | | |
| **Agilent ID** | **Gene Name or Accession Number** | **Fold Change** | **q-value** |
| A_23_P35309 | [TAF5L](http://genome-www4.stanford.edu/cgi-bin/SMD/source/sourceResult?choice=Gene&option=Name&criteria=TAF5L) | 0.25 | <0.0001 |
| A_24_P270769 | [VPS35](http://genome-www4.stanford.edu/cgi-bin/SMD/source/sourceResult?choice=Gene&option=Name&criteria=VPS35) | 0.62 | <0.0001 |
| A_23_P159305 | [TAF15](http://genome-www4.stanford.edu/cgi-bin/SMD/source/sourceResult?choice=Gene&option=Name&criteria=TAF15) | 0.46 | <0.0001 |
| A_24_P298495 | [AF289590](http://genome-www4.stanford.edu/cgi-bin/SMD/source/sourceResult?choice=Gene&option=Name&criteria=AF289590) | 0.36 | <0.0001 |
| A_23_P20752 | [CCRK](http://genome-www4.stanford.edu/cgi-bin/SMD/source/sourceResult?choice=Gene&option=Name&criteria=CCRK) | 0.58 | <0.0001 |
| A_32_P517749 | [RPS6KA3](http://genome-www4.stanford.edu/cgi-bin/SMD/source/sourceResult?choice=Gene&option=Name&criteria=RPS6KA3) | 0.58 | <0.0001 |
| A_23_P163380 | [MTHFS](http://genome-www4.stanford.edu/cgi-bin/SMD/source/sourceResult?choice=Gene&option=Name&criteria=MTHFS) | 0.65 | <0.0001 |
| A_23_P309261 | [AKAP9](http://genome-www4.stanford.edu/cgi-bin/SMD/source/sourceResult?choice=Gene&option=Name&criteria=AKAP9) | 0.57 | <0.0001 |
| A_23_P120883 | [HMOX1](http://genome-www4.stanford.edu/cgi-bin/SMD/source/sourceResult?choice=Gene&option=Name&criteria=HMOX1) | 0.49 | <0.0001 |
| A_23_P8311 | [TTRAP](http://genome-www4.stanford.edu/cgi-bin/SMD/source/sourceResult?choice=Gene&option=Name&criteria=TTRAP) | 0.49 | <0.0001 |
| A_23_P211850 | [ABHD6](http://genome-www4.stanford.edu/cgi-bin/SMD/source/sourceResult?choice=Gene&option=Name&criteria=ABHD6) | 0.49 | <0.0001 |
| A_24_P131392 | [FAM82A](http://genome-www4.stanford.edu/cgi-bin/SMD/source/sourceResult?choice=Gene&option=Name&criteria=FAM82A) | 0.58 | <0.0001 |
| A_24_P173823 | [ENST00000328681](http://genome-www4.stanford.edu/cgi-bin/SMD/source/sourceResult?choice=Gene&option=Name&criteria=ENST00000328681) | 0.65 | <0.0001 |
| A_32_P888644 | [MGC32805](http://genome-www4.stanford.edu/cgi-bin/SMD/source/sourceResult?choice=Gene&option=Name&criteria=MGC32805) | 0.40 | <0.0001 |
| A_23_P62128 | [MTM1](http://genome-www4.stanford.edu/cgi-bin/SMD/source/sourceResult?choice=Gene&option=Name&criteria=MTM1) | 0.61 | <0.0001 |
| A_23_P20494 | [NDRG1](http://genome-www4.stanford.edu/cgi-bin/SMD/source/sourceResult?choice=Gene&option=Name&criteria=NDRG1) | 0.58 | <0.0001 |
| A_32_P137632 | [FBXL17](http://genome-www4.stanford.edu/cgi-bin/SMD/source/sourceResult?choice=Gene&option=Name&criteria=FBXL17) | 0.32 | <0.0001 |
| A_23_P164912 | [LIN7B](http://genome-www4.stanford.edu/cgi-bin/SMD/source/sourceResult?choice=Gene&option=Name&criteria=LIN7B) | 0.66 | <0.0001 |
| A_23_P106773 | [SULT1A2](http://genome-www4.stanford.edu/cgi-bin/SMD/source/sourceResult?choice=Gene&option=Name&criteria=SULT1A2) | 0.59 | <0.0001 |
| A_23_P40240 | [CTSZ](http://genome-www4.stanford.edu/cgi-bin/SMD/source/sourceResult?choice=Gene&option=Name&criteria=CTSZ) | 0.41 | <0.0001 |
| A_23_P63032 | [GUCA2B](http://genome-www4.stanford.edu/cgi-bin/SMD/source/sourceResult?choice=Gene&option=Name&criteria=GUCA2B) | 0.47 | <0.0001 |
| A_23_P253012 | [GRAMD1C](http://genome-www4.stanford.edu/cgi-bin/SMD/source/sourceResult?choice=Gene&option=Name&criteria=GRAMD1C) | 0.46 | <0.0001 |
| A_23_P409945 | [OAZ1](http://genome-www4.stanford.edu/cgi-bin/SMD/source/sourceResult?choice=Gene&option=Name&criteria=OAZ1) | 0.63 | <0.0001 |
| A_32_P186921 | [ZNF616](http://genome-www4.stanford.edu/cgi-bin/SMD/source/sourceResult?choice=Gene&option=Name&criteria=ZNF616) | 0.66 | <0.0001 |
| A_32_P219116 | [CENPJ](http://genome-www4.stanford.edu/cgi-bin/SMD/source/sourceResult?choice=Gene&option=Name&criteria=CENPJ) | 0.65 | <0.0001 |
| A_23_P9402 | [CNTFR](http://genome-www4.stanford.edu/cgi-bin/SMD/source/sourceResult?choice=Gene&option=Name&criteria=CNTFR) | 0.53 | <0.0001 |
| A_24_P380679 | [FLJ39575](http://genome-www4.stanford.edu/cgi-bin/SMD/source/sourceResult?choice=Gene&option=Name&criteria=FLJ39575) | 0.61 | <0.0001 |
| A_23_P213050 | [HPGD](http://genome-www4.stanford.edu/cgi-bin/SMD/source/sourceResult?choice=Gene&option=Name&criteria=HPGD) | 0.61 | <0.0001 |
| A_23_P102160 | [FAM82A](http://genome-www4.stanford.edu/cgi-bin/SMD/source/sourceResult?choice=Gene&option=Name&criteria=FAM82A) | 0.59 | <0.0001 |
| A_23_P20285 | [PDLIM2](http://genome-www4.stanford.edu/cgi-bin/SMD/source/sourceResult?choice=Gene&option=Name&criteria=PDLIM2) | 0.54 | <0.0001 |
| A_32_P50670 | [BM807051](http://genome-www4.stanford.edu/cgi-bin/SMD/source/sourceResult?choice=Gene&option=Name&criteria=BM807051) | 0.54 | <0.0001 |
| A_32_P1144 | [AK091357](http://genome-www4.stanford.edu/cgi-bin/SMD/source/sourceResult?choice=Gene&option=Name&criteria=AK091357) | 0.55 | <0.0001 |
| A_24_P208909 | [TRIM2](http://genome-www4.stanford.edu/cgi-bin/SMD/source/sourceResult?choice=Gene&option=Name&criteria=TRIM2) | 0.62 | <0.0001 |
| A_32_P123 | [THC2295911](http://genome-www4.stanford.edu/cgi-bin/SMD/source/sourceResult?choice=Gene&option=Name&criteria=THC2295911) | 0.63 | <0.0001 |
| A_24_P298228 | [A_24_P298228](http://genome-www4.stanford.edu/cgi-bin/SMD/source/sourceResult?choice=Gene&option=Name&criteria=A_24_P298228) | 0.60 | <0.0001 |
| A_24_P930088 | [AK123264](http://genome-www4.stanford.edu/cgi-bin/SMD/source/sourceResult?choice=Gene&option=Name&criteria=AK123264) | 0.60 | <0.0001 |
| A_23_P250294 | [ABHD5](http://genome-www4.stanford.edu/cgi-bin/SMD/source/sourceResult?choice=Gene&option=Name&criteria=ABHD5) | 0.63 | <0.0001 |
| A_32_P227921 | [THC2283605](http://genome-www4.stanford.edu/cgi-bin/SMD/source/sourceResult?choice=Gene&option=Name&criteria=THC2283605) | 0.43 | <0.0001 |
| A_23_P43415 | [HSD17B3](http://genome-www4.stanford.edu/cgi-bin/SMD/source/sourceResult?choice=Gene&option=Name&criteria=HSD17B3) | 0.51 | <0.0001 |
| A_23_P39718 | [FEZ2](http://genome-www4.stanford.edu/cgi-bin/SMD/source/sourceResult?choice=Gene&option=Name&criteria=FEZ2) | 0.66 | <0.0001 |
| A_32_P235796 | [C4orf24](http://genome-www4.stanford.edu/cgi-bin/SMD/source/sourceResult?choice=Gene&option=Name&criteria=C4orf24) | 0.62 | <0.0001 |
| A_23_P136460 | [C5orf5](http://genome-www4.stanford.edu/cgi-bin/SMD/source/sourceResult?choice=Gene&option=Name&criteria=C5orf5) | 0.62 | <0.0001 |
| A_24_P329597 | [UBQLN1](http://genome-www4.stanford.edu/cgi-bin/SMD/source/sourceResult?choice=Gene&option=Name&criteria=UBQLN1) | 0.51 | <0.0001 |
| A_24_P82135 | [KIAA0256](http://genome-www4.stanford.edu/cgi-bin/SMD/source/sourceResult?choice=Gene&option=Name&criteria=KIAA0256) | 0.55 | <0.0001 |
| A_23_P9932 | [PDCD4](http://genome-www4.stanford.edu/cgi-bin/SMD/source/sourceResult?choice=Gene&option=Name&criteria=PDCD4) | 0.65 | <0.0001 |
| A_23_P85441 | [IGSF9](http://genome-www4.stanford.edu/cgi-bin/SMD/source/sourceResult?choice=Gene&option=Name&criteria=IGSF9) | 0.56 | <0.0001 |
| A_24_P294233 | [GLS](http://genome-www4.stanford.edu/cgi-bin/SMD/source/sourceResult?choice=Gene&option=Name&criteria=GLS) | 0.50 | <0.0001 |
| A_23_P31798 | [NAT2](http://genome-www4.stanford.edu/cgi-bin/SMD/source/sourceResult?choice=Gene&option=Name&criteria=NAT2) | 0.59 | <0.0001 |
| A_23_P7282 | [ENST00000323570](http://genome-www4.stanford.edu/cgi-bin/SMD/source/sourceResult?choice=Gene&option=Name&criteria=ENST00000323570) | 0.62 | <0.0001 |
| A_24_P182182 | [SLC25A5](http://genome-www4.stanford.edu/cgi-bin/SMD/source/sourceResult?choice=Gene&option=Name&criteria=SLC25A5) | 0.65 | <0.0001 |
| A_24_P389517 | [HNRPK](http://genome-www4.stanford.edu/cgi-bin/SMD/source/sourceResult?choice=Gene&option=Name&criteria=HNRPK) | 0.64 | <0.0001 |
| A_32_P69783 | [THC2445784](http://genome-www4.stanford.edu/cgi-bin/SMD/source/sourceResult?choice=Gene&option=Name&criteria=THC2445784) | 0.66 | <0.0001 |
| A_32_P217271 | [A_32_P217271](http://genome-www4.stanford.edu/cgi-bin/SMD/source/sourceResult?choice=Gene&option=Name&criteria=A_32_P217271) | 0.60 | <0.0001 |
| A_23_P15889 | [CBLN2](http://genome-www4.stanford.edu/cgi-bin/SMD/source/sourceResult?choice=Gene&option=Name&criteria=CBLN2) | 0.56 | <0.0001 |
| A_23_P379649 | [BMF](http://genome-www4.stanford.edu/cgi-bin/SMD/source/sourceResult?choice=Gene&option=Name&criteria=BMF) | 0.54 | <0.0001 |
| A_24_P8165 | [SLC15A1](http://genome-www4.stanford.edu/cgi-bin/SMD/source/sourceResult?choice=Gene&option=Name&criteria=SLC15A1) | 0.47 | <0.0001 |
| A_24_P318544 | [CSNK1D](http://genome-www4.stanford.edu/cgi-bin/SMD/source/sourceResult?choice=Gene&option=Name&criteria=CSNK1D) | 0.67 | <0.0001 |
| A_24_P23546 | [SP8](http://genome-www4.stanford.edu/cgi-bin/SMD/source/sourceResult?choice=Gene&option=Name&criteria=SP8) | 0.37 | <0.0001 |
| A_23_P75973 | [RNF121](http://genome-www4.stanford.edu/cgi-bin/SMD/source/sourceResult?choice=Gene&option=Name&criteria=RNF121) | 0.64 | <0.0001 |
| A_32_P208654 | [ENST00000353250](http://genome-www4.stanford.edu/cgi-bin/SMD/source/sourceResult?choice=Gene&option=Name&criteria=ENST00000353250) | 0.56 | <0.0001 |
| A_23_P9472 | [VPS13A](http://genome-www4.stanford.edu/cgi-bin/SMD/source/sourceResult?choice=Gene&option=Name&criteria=VPS13A) | 0.64 | <0.0001 |
| A_23_P201551 | [VAV3](http://genome-www4.stanford.edu/cgi-bin/SMD/source/sourceResult?choice=Gene&option=Name&criteria=VAV3) | 0.64 | <0.0001 |
| A_24_P237389 | [EIF1AX](http://genome-www4.stanford.edu/cgi-bin/SMD/source/sourceResult?choice=Gene&option=Name&criteria=EIF1AX) | 0.66 | <0.0001 |
| A_23_P346311 | [BAX](http://genome-www4.stanford.edu/cgi-bin/SMD/source/sourceResult?choice=Gene&option=Name&criteria=BAX) | 0.62 | 0.0028 |
| A_24_P182183 | [SLC25A5](http://genome-www4.stanford.edu/cgi-bin/SMD/source/sourceResult?choice=Gene&option=Name&criteria=SLC25A5) | 0.66 | 0.0028 |
| A_23_P202269 | [ANK3](http://genome-www4.stanford.edu/cgi-bin/SMD/source/sourceResult?choice=Gene&option=Name&criteria=ANK3) | 0.63 | 0.0028 |
| A_23_P146783 | [NEB](http://genome-www4.stanford.edu/cgi-bin/SMD/source/sourceResult?choice=Gene&option=Name&criteria=NEB) | 0.44 | 0.0028 |
| A_24_P603890 | [A_24_P603890](http://genome-www4.stanford.edu/cgi-bin/SMD/source/sourceResult?choice=Gene&option=Name&criteria=A_24_P603890) | 0.65 | 0.0028 |
| A_23_P145644 | [DDC](http://genome-www4.stanford.edu/cgi-bin/SMD/source/sourceResult?choice=Gene&option=Name&criteria=DDC) | 0.57 | 0.0028 |
| A_23_P127279 | [FAM35A](http://genome-www4.stanford.edu/cgi-bin/SMD/source/sourceResult?choice=Gene&option=Name&criteria=FAM35A) | 0.65 | 0.0028 |
| A_23_P152505 | [ABAT](http://genome-www4.stanford.edu/cgi-bin/SMD/source/sourceResult?choice=Gene&option=Name&criteria=ABAT) | 0.61 | 0.0028 |
| A_24_P262201 | [SULT1A4](http://genome-www4.stanford.edu/cgi-bin/SMD/source/sourceResult?choice=Gene&option=Name&criteria=SULT1A4) | 0.66 | 0.0028 |
| A_32_P144326 | [AJ420450](http://genome-www4.stanford.edu/cgi-bin/SMD/source/sourceResult?choice=Gene&option=Name&criteria=AJ420450) | 0.63 | 0.0028 |
| A_32_P83811 | [AK124936](http://genome-www4.stanford.edu/cgi-bin/SMD/source/sourceResult?choice=Gene&option=Name&criteria=AK124936) | 0.43 | 0.0028 |
| A_23_P78018 | [ABCA5](http://genome-www4.stanford.edu/cgi-bin/SMD/source/sourceResult?choice=Gene&option=Name&criteria=ABCA5) | 0.59 | 0.0028 |
| A_24_P925505 | [CD36](http://genome-www4.stanford.edu/cgi-bin/SMD/source/sourceResult?choice=Gene&option=Name&criteria=CD36) | 0.60 | 0.0028 |
| A_24_P260325 | [LRAT](http://genome-www4.stanford.edu/cgi-bin/SMD/source/sourceResult?choice=Gene&option=Name&criteria=LRAT) | 0.57 | 0.0028 |
| A_24_P115621 | [ENST00000373218](http://genome-www4.stanford.edu/cgi-bin/SMD/source/sourceResult?choice=Gene&option=Name&criteria=ENST00000373218) | 0.67 | 0.0028 |
| A_23_P9232 | [GCNT1](http://genome-www4.stanford.edu/cgi-bin/SMD/source/sourceResult?choice=Gene&option=Name&criteria=GCNT1) | 0.66 | 0.0028 |
| A_23_P253301 | [PFN2](http://genome-www4.stanford.edu/cgi-bin/SMD/source/sourceResult?choice=Gene&option=Name&criteria=PFN2) | 0.57 | 0.0028 |
| A_24_P308506 | [CML2](http://genome-www4.stanford.edu/cgi-bin/SMD/source/sourceResult?choice=Gene&option=Name&criteria=CML2) | 0.47 | 0.0028 |
| A_23_P59718 | [SRI](http://genome-www4.stanford.edu/cgi-bin/SMD/source/sourceResult?choice=Gene&option=Name&criteria=SRI) | 0.59 | 0.0028 |
| A_23_P43425 | [C9orf40](http://genome-www4.stanford.edu/cgi-bin/SMD/source/sourceResult?choice=Gene&option=Name&criteria=C9orf40) | 0.66 | 0.0028 |
| A_24_P387609 | [HBLD2](http://genome-www4.stanford.edu/cgi-bin/SMD/source/sourceResult?choice=Gene&option=Name&criteria=HBLD2) | 0.61 | 0.0028 |
| A_24_P114334 | [RMND5A](http://genome-www4.stanford.edu/cgi-bin/SMD/source/sourceResult?choice=Gene&option=Name&criteria=RMND5A) | 0.66 | 0.0028 |
| A_24_P174755 | [SLC22A5](http://genome-www4.stanford.edu/cgi-bin/SMD/source/sourceResult?choice=Gene&option=Name&criteria=SLC22A5) | 0.63 | 0.0028 |
| A_32_P8666 | [LOC644063](http://genome-www4.stanford.edu/cgi-bin/SMD/source/sourceResult?choice=Gene&option=Name&criteria=LOC644063) | 0.64 | 0.0028 |
| A_23_P27795 | [SPINT2](http://genome-www4.stanford.edu/cgi-bin/SMD/source/sourceResult?choice=Gene&option=Name&criteria=SPINT2) | 0.66 | 0.0028 |
| A_23_P214658 | [PBX2](http://genome-www4.stanford.edu/cgi-bin/SMD/source/sourceResult?choice=Gene&option=Name&criteria=PBX2) | 0.53 | 0.0028 |
| A_23_P502654 | [SHMT1](http://genome-www4.stanford.edu/cgi-bin/SMD/source/sourceResult?choice=Gene&option=Name&criteria=SHMT1) | 0.64 | 0.0028 |
| A_32_P211276 | [THC2344886](http://genome-www4.stanford.edu/cgi-bin/SMD/source/sourceResult?choice=Gene&option=Name&criteria=THC2344886) | 0.63 | 0.0028 |
| A_23_P305120 | [ANKRD23](http://genome-www4.stanford.edu/cgi-bin/SMD/source/sourceResult?choice=Gene&option=Name&criteria=ANKRD23) | 0.66 | 0.0028 |
| A_23_P324107 | [RORC](http://genome-www4.stanford.edu/cgi-bin/SMD/source/sourceResult?choice=Gene&option=Name&criteria=RORC) | 0.59 | 0.0028 |
| A_32_P174285 | [THC2442021](http://genome-www4.stanford.edu/cgi-bin/SMD/source/sourceResult?choice=Gene&option=Name&criteria=THC2442021) | 0.53 | 0.0028 |
| A_23_P51996 | [STXBP3](http://genome-www4.stanford.edu/cgi-bin/SMD/source/sourceResult?choice=Gene&option=Name&criteria=STXBP3) | 0.63 | 0.0028 |
| A_23_P160406 | [KCTD3](http://genome-www4.stanford.edu/cgi-bin/SMD/source/sourceResult?choice=Gene&option=Name&criteria=KCTD3) | 0.59 | 0.0028 |
| A_23_P28969 | [CHMP4B](http://genome-www4.stanford.edu/cgi-bin/SMD/source/sourceResult?choice=Gene&option=Name&criteria=CHMP4B) | 0.58 | 0.0028 |
| A_23_P56933 | [RTN4](http://genome-www4.stanford.edu/cgi-bin/SMD/source/sourceResult?choice=Gene&option=Name&criteria=RTN4) | 0.62 | 0.0028 |
| A_23_P146798 | [SEPHS2](http://genome-www4.stanford.edu/cgi-bin/SMD/source/sourceResult?choice=Gene&option=Name&criteria=SEPHS2) | 0.64 | 0.0028 |
| A_23_P54781 | [RBBP6](http://genome-www4.stanford.edu/cgi-bin/SMD/source/sourceResult?choice=Gene&option=Name&criteria=RBBP6) | 0.57 | 0.0028 |
| A_23_P120594 | [ACSS1](http://genome-www4.stanford.edu/cgi-bin/SMD/source/sourceResult?choice=Gene&option=Name&criteria=ACSS1) | 0.66 | 0.0028 |
| A_23_P256391 | [GOLGA4](http://genome-www4.stanford.edu/cgi-bin/SMD/source/sourceResult?choice=Gene&option=Name&criteria=GOLGA4) | 0.60 | 0.0028 |
| A_23_P159764 | [POF1B](http://genome-www4.stanford.edu/cgi-bin/SMD/source/sourceResult?choice=Gene&option=Name&criteria=POF1B) | 0.58 | 0.0028 |
| A_24_P602507 | [LOC123688](http://genome-www4.stanford.edu/cgi-bin/SMD/source/sourceResult?choice=Gene&option=Name&criteria=LOC123688) | 0.60 | 0.0028 |
| A_23_P160582 | [HYI](http://genome-www4.stanford.edu/cgi-bin/SMD/source/sourceResult?choice=Gene&option=Name&criteria=HYI) | 0.65 | 0.0028 |
| A_23_P166306 | [CBS](http://genome-www4.stanford.edu/cgi-bin/SMD/source/sourceResult?choice=Gene&option=Name&criteria=CBS) | 0.65 | 0.0028 |
| A_24_P325533 | [A_24_P325533](http://genome-www4.stanford.edu/cgi-bin/SMD/source/sourceResult?choice=Gene&option=Name&criteria=A_24_P325533) | 0.57 | 0.0028 |
| A_23_P207371 | [G6PC](http://genome-www4.stanford.edu/cgi-bin/SMD/source/sourceResult?choice=Gene&option=Name&criteria=G6PC) | 0.48 | 0.0028 |
| A_23_P217098 | [VPS13A](http://genome-www4.stanford.edu/cgi-bin/SMD/source/sourceResult?choice=Gene&option=Name&criteria=VPS13A) | 0.65 | 0.0028 |
| A_24_P187706 | [GOLGA4](http://genome-www4.stanford.edu/cgi-bin/SMD/source/sourceResult?choice=Gene&option=Name&criteria=GOLGA4) | 0.66 | 0.0028 |
| A_23_P22350 | [GRAMD3](http://genome-www4.stanford.edu/cgi-bin/SMD/source/sourceResult?choice=Gene&option=Name&criteria=GRAMD3) | 0.62 | 0.0028 |
| A_24_P51485 | [CSDE1](http://genome-www4.stanford.edu/cgi-bin/SMD/source/sourceResult?choice=Gene&option=Name&criteria=CSDE1) | 0.65 | 0.0028 |
| A_23_P8961 | [IL7](http://genome-www4.stanford.edu/cgi-bin/SMD/source/sourceResult?choice=Gene&option=Name&criteria=IL7) | 0.61 | 0.0028 |
| A_23_P364625 | [LRRC19](http://genome-www4.stanford.edu/cgi-bin/SMD/source/sourceResult?choice=Gene&option=Name&criteria=LRRC19) | 0.62 | 0.0028 |
| A_23_P103486 | [CYP2J2](http://genome-www4.stanford.edu/cgi-bin/SMD/source/sourceResult?choice=Gene&option=Name&criteria=CYP2J2) | 0.51 | 0.0028 |
| A_24_P225845 | [AK026659](http://genome-www4.stanford.edu/cgi-bin/SMD/source/sourceResult?choice=Gene&option=Name&criteria=AK026659) | 0.56 | 0.0028 |
| A_32_P155811 | [THC2276639](http://genome-www4.stanford.edu/cgi-bin/SMD/source/sourceResult?choice=Gene&option=Name&criteria=THC2276639) | 0.60 | 0.0028 |
| A_32_P230398 | [THC2440435](http://genome-www4.stanford.edu/cgi-bin/SMD/source/sourceResult?choice=Gene&option=Name&criteria=THC2440435) | 0.61 | 0.0028 |
| A_23_P434929 | [CCDC100](http://genome-www4.stanford.edu/cgi-bin/SMD/source/sourceResult?choice=Gene&option=Name&criteria=CCDC100) | 0.59 | 0.0028 |
| A_23_P163408 | [ZNF291](http://genome-www4.stanford.edu/cgi-bin/SMD/source/sourceResult?choice=Gene&option=Name&criteria=ZNF291) | 0.57 | 0.0028 |
| A_23_P336342 | [AK124576](http://genome-www4.stanford.edu/cgi-bin/SMD/source/sourceResult?choice=Gene&option=Name&criteria=AK124576) | 0.65 | 0.0028 |
| A_23_P47565 | [LDHA](http://genome-www4.stanford.edu/cgi-bin/SMD/source/sourceResult?choice=Gene&option=Name&criteria=LDHA) | 0.62 | 0.0028 |
| A_23_P420442 | [SEMA6D](http://genome-www4.stanford.edu/cgi-bin/SMD/source/sourceResult?choice=Gene&option=Name&criteria=SEMA6D) | 0.57 | 0.0028 |
| A_32_P190944 | [A_32_P190944](http://genome-www4.stanford.edu/cgi-bin/SMD/source/sourceResult?choice=Gene&option=Name&criteria=A_32_P190944) | 0.53 | 0.0028 |
| A_23_P140207 | [PCK2](http://genome-www4.stanford.edu/cgi-bin/SMD/source/sourceResult?choice=Gene&option=Name&criteria=PCK2) | 0.55 | 0.0028 |
| A_23_P382835 | [P2RY1](http://genome-www4.stanford.edu/cgi-bin/SMD/source/sourceResult?choice=Gene&option=Name&criteria=P2RY1) | 0.60 | 0.0028 |
| A_23_P52266 | [IFIT1](http://genome-www4.stanford.edu/cgi-bin/SMD/source/sourceResult?choice=Gene&option=Name&criteria=IFIT1) | 0.60 | 0.0028 |
| A_32_P110472 | [C6orf71](http://genome-www4.stanford.edu/cgi-bin/SMD/source/sourceResult?choice=Gene&option=Name&criteria=C6orf71) | 0.55 | 0.0028 |
| A_23_P326852 | [CACNB2](http://genome-www4.stanford.edu/cgi-bin/SMD/source/sourceResult?choice=Gene&option=Name&criteria=CACNB2) | 0.60 | 0.0028 |
| A_23_P31376 | [LRRN3](http://genome-www4.stanford.edu/cgi-bin/SMD/source/sourceResult?choice=Gene&option=Name&criteria=LRRN3) | 0.57 | 0.0028 |
| A_32_P155247 | [FTL](http://genome-www4.stanford.edu/cgi-bin/SMD/source/sourceResult?choice=Gene&option=Name&criteria=FTL) | 0.62 | 0.0028 |
| A_23_P126057 | [SCP2](http://genome-www4.stanford.edu/cgi-bin/SMD/source/sourceResult?choice=Gene&option=Name&criteria=SCP2) | 0.62 | 0.0028 |
| A_23_P7342 | [UGT2B10](http://genome-www4.stanford.edu/cgi-bin/SMD/source/sourceResult?choice=Gene&option=Name&criteria=UGT2B10) | 0.58 | 0.0028 |
| A_23_P16006 | [ZNF600](http://genome-www4.stanford.edu/cgi-bin/SMD/source/sourceResult?choice=Gene&option=Name&criteria=ZNF600) | 0.64 | 0.0028 |
| A_23_P106024 | [JAG2](http://genome-www4.stanford.edu/cgi-bin/SMD/source/sourceResult?choice=Gene&option=Name&criteria=JAG2) | 0.62 | 0.0028 |
| A_23_P103765 | [FCER1A](http://genome-www4.stanford.edu/cgi-bin/SMD/source/sourceResult?choice=Gene&option=Name&criteria=FCER1A) | 0.59 | 0.0028 |
| A_23_P129144 | [Gcom1](http://genome-www4.stanford.edu/cgi-bin/SMD/source/sourceResult?choice=Gene&option=Name&criteria=Gcom1) | 0.58 | 0.0028 |
| A_32_P184394 | [TFEC](http://genome-www4.stanford.edu/cgi-bin/SMD/source/sourceResult?choice=Gene&option=Name&criteria=TFEC) | 0.43 | 0.0028 |
| A_23_P97795 | [ACBD5](http://genome-www4.stanford.edu/cgi-bin/SMD/source/sourceResult?choice=Gene&option=Name&criteria=ACBD5) | 0.64 | 0.0028 |
| A_32_P16258 | [SEC15L2](http://genome-www4.stanford.edu/cgi-bin/SMD/source/sourceResult?choice=Gene&option=Name&criteria=SEC15L2) | 0.61 | 0.0028 |
| A_24_P623814 | [AK023526](http://genome-www4.stanford.edu/cgi-bin/SMD/source/sourceResult?choice=Gene&option=Name&criteria=AK023526) | 0.60 | 0.0028 |
| A_23_P47924 | [PTPRR](http://genome-www4.stanford.edu/cgi-bin/SMD/source/sourceResult?choice=Gene&option=Name&criteria=PTPRR) | 0.55 | 0.0039 |
| A_23_P110492 | [MARCH6](http://genome-www4.stanford.edu/cgi-bin/SMD/source/sourceResult?choice=Gene&option=Name&criteria=3/6/2010) | 0.62 | 0.0039 |
| A_24_P62530 | [RHOU](http://genome-www4.stanford.edu/cgi-bin/SMD/source/sourceResult?choice=Gene&option=Name&criteria=RHOU) | 0.56 | 0.0039 |
| A_23_P418234 | [PHLPPL](http://genome-www4.stanford.edu/cgi-bin/SMD/source/sourceResult?choice=Gene&option=Name&criteria=PHLPPL) | 0.58 | 0.0039 |
| A_32_P23525 | [AK124281](http://genome-www4.stanford.edu/cgi-bin/SMD/source/sourceResult?choice=Gene&option=Name&criteria=AK124281) | 0.61 | 0.0039 |
| A_23_P74794 | [ABCD3](http://genome-www4.stanford.edu/cgi-bin/SMD/source/sourceResult?choice=Gene&option=Name&criteria=ABCD3) | 0.59 | 0.0039 |
| A_23_P379034 | [BAIAP2L2](http://genome-www4.stanford.edu/cgi-bin/SMD/source/sourceResult?choice=Gene&option=Name&criteria=BAIAP2L2) | 0.64 | 0.0039 |
| A_23_P8834 | [EPHX2](http://genome-www4.stanford.edu/cgi-bin/SMD/source/sourceResult?choice=Gene&option=Name&criteria=EPHX2) | 0.65 | 0.0039 |
| A_23_P90542 | [ZNF540](http://genome-www4.stanford.edu/cgi-bin/SMD/source/sourceResult?choice=Gene&option=Name&criteria=ZNF540) | 0.56 | 0.0039 |
| A_24_P538708 | [AK124263](http://genome-www4.stanford.edu/cgi-bin/SMD/source/sourceResult?choice=Gene&option=Name&criteria=AK124263) | 0.65 | 0.0039 |
| A_24_P179183 | [ANKRD12](http://genome-www4.stanford.edu/cgi-bin/SMD/source/sourceResult?choice=Gene&option=Name&criteria=ANKRD12) | 0.66 | 0.0039 |
| A_32_P181826 | [THC2342112](http://genome-www4.stanford.edu/cgi-bin/SMD/source/sourceResult?choice=Gene&option=Name&criteria=THC2342112) | 0.62 | 0.0039 |
| A_23_P148345 | [RNF128](http://genome-www4.stanford.edu/cgi-bin/SMD/source/sourceResult?choice=Gene&option=Name&criteria=RNF128) | 0.60 | 0.0039 |
| A_23_P96369 | [CXorf57](http://genome-www4.stanford.edu/cgi-bin/SMD/source/sourceResult?choice=Gene&option=Name&criteria=CXorf57) | 0.61 | 0.0039 |
| A_24_P374634 | [STAU2](http://genome-www4.stanford.edu/cgi-bin/SMD/source/sourceResult?choice=Gene&option=Name&criteria=STAU2) | 0.56 | 0.0039 |
| A_23_P145711 | [C7orf10](http://genome-www4.stanford.edu/cgi-bin/SMD/source/sourceResult?choice=Gene&option=Name&criteria=C7orf10) | 0.65 | 0.0039 |
| A_24_P405850 | [PGRMC2](http://genome-www4.stanford.edu/cgi-bin/SMD/source/sourceResult?choice=Gene&option=Name&criteria=PGRMC2) | 0.62 | 0.0039 |
| A_23_P50735 | [ZNF181](http://genome-www4.stanford.edu/cgi-bin/SMD/source/sourceResult?choice=Gene&option=Name&criteria=ZNF181) | 0.61 | 0.0039 |
| A_23_P25069 | [OVOS2](http://genome-www4.stanford.edu/cgi-bin/SMD/source/sourceResult?choice=Gene&option=Name&criteria=OVOS2) | 0.60 | 0.0039 |
| A_23_P151198 | [PDAP1](http://genome-www4.stanford.edu/cgi-bin/SMD/source/sourceResult?choice=Gene&option=Name&criteria=PDAP1) | 0.64 | 0.0039 |
| A_24_P941787 | [PRPF4B](http://genome-www4.stanford.edu/cgi-bin/SMD/source/sourceResult?choice=Gene&option=Name&criteria=PRPF4B) | 0.67 | 0.0039 |
| A_32_P104334 | [AW972815](http://genome-www4.stanford.edu/cgi-bin/SMD/source/sourceResult?choice=Gene&option=Name&criteria=AW972815) | 0.50 | 0.0039 |
| A_24_P13790 | [CES2](http://genome-www4.stanford.edu/cgi-bin/SMD/source/sourceResult?choice=Gene&option=Name&criteria=CES2) | 0.60 | 0.0039 |
| A_24_P833256 | [THC2364469](http://genome-www4.stanford.edu/cgi-bin/SMD/source/sourceResult?choice=Gene&option=Name&criteria=THC2364469) | 0.44 | 0.0039 |
| A_32_P125832 | [AK123079](http://genome-www4.stanford.edu/cgi-bin/SMD/source/sourceResult?choice=Gene&option=Name&criteria=AK123079) | 0.59 | 0.0039 |
| A_32_P52911 | [ADI1](http://genome-www4.stanford.edu/cgi-bin/SMD/source/sourceResult?choice=Gene&option=Name&criteria=ADI1) | 0.65 | 0.0051 |
| A_23_P215505 | [RAPGEF5](http://genome-www4.stanford.edu/cgi-bin/SMD/source/sourceResult?choice=Gene&option=Name&criteria=RAPGEF5) | 0.44 | 0.0051 |
| A_23_P77328 | [GCHFR](http://genome-www4.stanford.edu/cgi-bin/SMD/source/sourceResult?choice=Gene&option=Name&criteria=GCHFR) | 0.63 | 0.0051 |
| A_23_P200685 | [MOSC2](http://genome-www4.stanford.edu/cgi-bin/SMD/source/sourceResult?choice=Gene&option=Name&criteria=MOSC2) | 0.61 | 0.0051 |
| A_32_P81173 | [USP34](http://genome-www4.stanford.edu/cgi-bin/SMD/source/sourceResult?choice=Gene&option=Name&criteria=USP34) | 0.61 | 0.0051 |
| A_24_P63468 | [AGXT2](http://genome-www4.stanford.edu/cgi-bin/SMD/source/sourceResult?choice=Gene&option=Name&criteria=AGXT2) | 0.46 | 0.0051 |
| A_23_P10182 | [ACOX2](http://genome-www4.stanford.edu/cgi-bin/SMD/source/sourceResult?choice=Gene&option=Name&criteria=ACOX2) | 0.57 | 0.0051 |
| A_24_P32715 | [A_24_P32715](http://genome-www4.stanford.edu/cgi-bin/SMD/source/sourceResult?choice=Gene&option=Name&criteria=A_24_P32715) | 0.67 | 0.0051 |
| A_24_P192727 | [ENST00000224809](http://genome-www4.stanford.edu/cgi-bin/SMD/source/sourceResult?choice=Gene&option=Name&criteria=ENST00000224809) | 0.61 | 0.0051 |
| A_23_P24004 | [IFIT2](http://genome-www4.stanford.edu/cgi-bin/SMD/source/sourceResult?choice=Gene&option=Name&criteria=IFIT2) | 0.53 | 0.0051 |
| A_23_P214411 | [GLO1](http://genome-www4.stanford.edu/cgi-bin/SMD/source/sourceResult?choice=Gene&option=Name&criteria=GLO1) | 0.62 | 0.0051 |
| A_24_P203696 | [ENST00000301171](http://genome-www4.stanford.edu/cgi-bin/SMD/source/sourceResult?choice=Gene&option=Name&criteria=ENST00000301171) | 0.58 | 0.0051 |
| A_23_P1352 | [SFRP5](http://genome-www4.stanford.edu/cgi-bin/SMD/source/sourceResult?choice=Gene&option=Name&criteria=SFRP5) | 0.56 | 0.0051 |
| A_23_P123315 | [BC067244](http://genome-www4.stanford.edu/cgi-bin/SMD/source/sourceResult?choice=Gene&option=Name&criteria=BC067244) | 0.66 | 0.0051 |
| A_32_P101264 | [ENST00000371408](http://genome-www4.stanford.edu/cgi-bin/SMD/source/sourceResult?choice=Gene&option=Name&criteria=ENST00000371408) | 0.62 | 0.0051 |
| A_24_P71904 | [HPGD](http://genome-www4.stanford.edu/cgi-bin/SMD/source/sourceResult?choice=Gene&option=Name&criteria=HPGD) | 0.58 | 0.0051 |
| A_32_P38228 | [A_32_P38228](http://genome-www4.stanford.edu/cgi-bin/SMD/source/sourceResult?choice=Gene&option=Name&criteria=A_32_P38228) | 0.67 | 0.0051 |
| A_23_P357270 | [SLC17A4](http://genome-www4.stanford.edu/cgi-bin/SMD/source/sourceResult?choice=Gene&option=Name&criteria=SLC17A4) | 0.64 | 0.0051 |
| A_23_P397347 | [MCMDC1](http://genome-www4.stanford.edu/cgi-bin/SMD/source/sourceResult?choice=Gene&option=Name&criteria=MCMDC1) | 0.63 | 0.0051 |
| A_23_P145424 | [KIAA1009](http://genome-www4.stanford.edu/cgi-bin/SMD/source/sourceResult?choice=Gene&option=Name&criteria=KIAA1009) | 0.66 | 0.0051 |
| A_23_P112726 | [SCN9A](http://genome-www4.stanford.edu/cgi-bin/SMD/source/sourceResult?choice=Gene&option=Name&criteria=SCN9A) | 0.44 | 0.0051 |
| A_23_P214821 | [EDN1](http://genome-www4.stanford.edu/cgi-bin/SMD/source/sourceResult?choice=Gene&option=Name&criteria=EDN1) | 0.62 | 0.0051 |
| A_23_P72568 | [SNX4](http://genome-www4.stanford.edu/cgi-bin/SMD/source/sourceResult?choice=Gene&option=Name&criteria=SNX4) | 0.65 | 0.0051 |
| A_23_P167005 | [GPR160](http://genome-www4.stanford.edu/cgi-bin/SMD/source/sourceResult?choice=Gene&option=Name&criteria=GPR160) | 0.63 | 0.0051 |
| A_23_P372834 | [AQP1](http://genome-www4.stanford.edu/cgi-bin/SMD/source/sourceResult?choice=Gene&option=Name&criteria=AQP1) | 0.65 | 0.0051 |
| A_24_P329152 | [CD2AP](http://genome-www4.stanford.edu/cgi-bin/SMD/source/sourceResult?choice=Gene&option=Name&criteria=CD2AP) | 0.56 | 0.0051 |
| A_24_P380330 | [PANK3](http://genome-www4.stanford.edu/cgi-bin/SMD/source/sourceResult?choice=Gene&option=Name&criteria=PANK3) | 0.66 | 0.0051 |
| A_32_P34522 | [BC010544](http://genome-www4.stanford.edu/cgi-bin/SMD/source/sourceResult?choice=Gene&option=Name&criteria=BC010544) | 0.65 | 0.0051 |
| A_32_P66908 | [ENST00000305749](http://genome-www4.stanford.edu/cgi-bin/SMD/source/sourceResult?choice=Gene&option=Name&criteria=ENST00000305749) | 0.57 | 0.0051 |
| A_23_P154379 | [NAT8](http://genome-www4.stanford.edu/cgi-bin/SMD/source/sourceResult?choice=Gene&option=Name&criteria=NAT8) | 0.45 | 0.0051 |
| A_23_P53039 | [LDHC](http://genome-www4.stanford.edu/cgi-bin/SMD/source/sourceResult?choice=Gene&option=Name&criteria=LDHC) | 0.58 | 0.0051 |
| A_23_P105856 | [LOC283537](http://genome-www4.stanford.edu/cgi-bin/SMD/source/sourceResult?choice=Gene&option=Name&criteria=LOC283537) | 0.58 | 0.0051 |
| A_23_P39445 | [RKHD1](http://genome-www4.stanford.edu/cgi-bin/SMD/source/sourceResult?choice=Gene&option=Name&criteria=RKHD1) | 0.66 | 0.0062 |
| A_23_P258037 | [JMJD1A](http://genome-www4.stanford.edu/cgi-bin/SMD/source/sourceResult?choice=Gene&option=Name&criteria=JMJD1A) | 0.65 | 0.0062 |
| A_32_P47643 | [CR601458](http://genome-www4.stanford.edu/cgi-bin/SMD/source/sourceResult?choice=Gene&option=Name&criteria=CR601458) | 0.65 | 0.0062 |
| A_23_P203191 | [APOA1](http://genome-www4.stanford.edu/cgi-bin/SMD/source/sourceResult?choice=Gene&option=Name&criteria=APOA1) | 0.46 | 0.0062 |
| A_23_P48455 | [AMN](http://genome-www4.stanford.edu/cgi-bin/SMD/source/sourceResult?choice=Gene&option=Name&criteria=AMN) | 0.57 | 0.0062 |
| A_23_P111583 | [CD36](http://genome-www4.stanford.edu/cgi-bin/SMD/source/sourceResult?choice=Gene&option=Name&criteria=CD36) | 0.65 | 0.0062 |
| A_23_P118894 | [ATAD4](http://genome-www4.stanford.edu/cgi-bin/SMD/source/sourceResult?choice=Gene&option=Name&criteria=ATAD4) | 0.65 | 0.0062 |
| A_23_P412029 | [FLJ32312](http://genome-www4.stanford.edu/cgi-bin/SMD/source/sourceResult?choice=Gene&option=Name&criteria=FLJ32312) | 0.52 | 0.0062 |
| A_24_P750817 | [LOC147710](http://genome-www4.stanford.edu/cgi-bin/SMD/source/sourceResult?choice=Gene&option=Name&criteria=LOC147710) | 0.59 | 0.0062 |
| A_23_P48747 | [DHRS1](http://genome-www4.stanford.edu/cgi-bin/SMD/source/sourceResult?choice=Gene&option=Name&criteria=DHRS1) | 0.65 | 0.0062 |
| A_23_P62133 | [MTM1](http://genome-www4.stanford.edu/cgi-bin/SMD/source/sourceResult?choice=Gene&option=Name&criteria=MTM1) | 0.65 | 0.0062 |
| A_23_P71790 | [MAMDC4](http://genome-www4.stanford.edu/cgi-bin/SMD/source/sourceResult?choice=Gene&option=Name&criteria=MAMDC4) | 0.66 | 0.0062 |
| A_23_P434430 | [ZNF439](http://genome-www4.stanford.edu/cgi-bin/SMD/source/sourceResult?choice=Gene&option=Name&criteria=ZNF439) | 0.66 | 0.0062 |
| A_24_P880043 | [PCGF5](http://genome-www4.stanford.edu/cgi-bin/SMD/source/sourceResult?choice=Gene&option=Name&criteria=PCGF5) | 0.64 | 0.0062 |
| A_32_P73304 | [THC2360305](http://genome-www4.stanford.edu/cgi-bin/SMD/source/sourceResult?choice=Gene&option=Name&criteria=THC2360305) | 0.63 | 0.0062 |
| A_32_P198029 | [BC031013](http://genome-www4.stanford.edu/cgi-bin/SMD/source/sourceResult?choice=Gene&option=Name&criteria=BC031013) | 0.59 | 0.0062 |
| A_24_P250815 | [POF1B](http://genome-www4.stanford.edu/cgi-bin/SMD/source/sourceResult?choice=Gene&option=Name&criteria=POF1B) | 0.61 | 0.0062 |
| A_24_P566968 | [AK093869](http://genome-www4.stanford.edu/cgi-bin/SMD/source/sourceResult?choice=Gene&option=Name&criteria=AK093869) | 0.66 | 0.0062 |
| A_32_P211188 | [LOC153346](http://genome-www4.stanford.edu/cgi-bin/SMD/source/sourceResult?choice=Gene&option=Name&criteria=LOC153346) | 0.66 | 0.0062 |
| A_23_P92842 | [SAR1B](http://genome-www4.stanford.edu/cgi-bin/SMD/source/sourceResult?choice=Gene&option=Name&criteria=SAR1B) | 0.64 | 0.0062 |
| A_23_P157809 | [LTB4DH](http://genome-www4.stanford.edu/cgi-bin/SMD/source/sourceResult?choice=Gene&option=Name&criteria=LTB4DH) | 0.57 | 0.0062 |
| A_23_P15402 | [SAT2](http://genome-www4.stanford.edu/cgi-bin/SMD/source/sourceResult?choice=Gene&option=Name&criteria=SAT2) | 0.59 | 0.0062 |
| A_23_P28246 | [SLC23A3](http://genome-www4.stanford.edu/cgi-bin/SMD/source/sourceResult?choice=Gene&option=Name&criteria=SLC23A3) | 0.52 | 0.0062 |
| A_24_P136471 | [SLC14A2](http://genome-www4.stanford.edu/cgi-bin/SMD/source/sourceResult?choice=Gene&option=Name&criteria=SLC14A2) | 0.50 | 0.0062 |
| A_32_P24140 | [GAS2](http://genome-www4.stanford.edu/cgi-bin/SMD/source/sourceResult?choice=Gene&option=Name&criteria=GAS2) | 0.63 | 0.0062 |
| A_23_P18713 | [ABCG2](http://genome-www4.stanford.edu/cgi-bin/SMD/source/sourceResult?choice=Gene&option=Name&criteria=ABCG2) | 0.57 | 0.0062 |
| A_32_P140153 | [THC2439228](http://genome-www4.stanford.edu/cgi-bin/SMD/source/sourceResult?choice=Gene&option=Name&criteria=THC2439228) | 0.63 | 0.0062 |
| A_23_P212968 | [UGT2B11](http://genome-www4.stanford.edu/cgi-bin/SMD/source/sourceResult?choice=Gene&option=Name&criteria=UGT2B11) | 0.54 | 0.0062 |
| A_23_P102364 | [NGEF](http://genome-www4.stanford.edu/cgi-bin/SMD/source/sourceResult?choice=Gene&option=Name&criteria=NGEF) | 0.63 | 0.0062 |
| A_23_P421011 | [KAZALD1](http://genome-www4.stanford.edu/cgi-bin/SMD/source/sourceResult?choice=Gene&option=Name&criteria=KAZALD1) | 0.66 | 0.0062 |
| A_24_P253827 | [AP2B1](http://genome-www4.stanford.edu/cgi-bin/SMD/source/sourceResult?choice=Gene&option=Name&criteria=AP2B1) | 0.57 | 0.0062 |
| A_23_P386356 | [KIF12](http://genome-www4.stanford.edu/cgi-bin/SMD/source/sourceResult?choice=Gene&option=Name&criteria=KIF12) | 0.52 | 0.0062 |
| A_23_P60599 | [UGT1A6](http://genome-www4.stanford.edu/cgi-bin/SMD/source/sourceResult?choice=Gene&option=Name&criteria=UGT1A6) | 0.58 | 0.0062 |
| A_23_P152087 | [FAM82C](http://genome-www4.stanford.edu/cgi-bin/SMD/source/sourceResult?choice=Gene&option=Name&criteria=FAM82C) | 0.65 | 0.0062 |
| A_23_P79217 | [LCT](http://genome-www4.stanford.edu/cgi-bin/SMD/source/sourceResult?choice=Gene&option=Name&criteria=LCT) | 0.40 | 0.0062 |
| A_23_P9056 | [RB1CC1](http://genome-www4.stanford.edu/cgi-bin/SMD/source/sourceResult?choice=Gene&option=Name&criteria=RB1CC1) | 0.64 | 0.0062 |
| A_23_P66948 | [FAM59A](http://genome-www4.stanford.edu/cgi-bin/SMD/source/sourceResult?choice=Gene&option=Name&criteria=FAM59A) | 0.66 | 0.0062 |
| A_32_P61439 | [THC2416008](http://genome-www4.stanford.edu/cgi-bin/SMD/source/sourceResult?choice=Gene&option=Name&criteria=THC2416008) | 0.66 | 0.0080 |
| A_23_P14515 | [ACOT4](http://genome-www4.stanford.edu/cgi-bin/SMD/source/sourceResult?choice=Gene&option=Name&criteria=ACOT4) | 0.64 | 0.0080 |
| A_32_P158181 | [THC2437757](http://genome-www4.stanford.edu/cgi-bin/SMD/source/sourceResult?choice=Gene&option=Name&criteria=THC2437757) | 0.49 | 0.0080 |
| A_23_P89422 | [ABCA10](http://genome-www4.stanford.edu/cgi-bin/SMD/source/sourceResult?choice=Gene&option=Name&criteria=ABCA10) | 0.66 | 0.0080 |
| A_23_P118334 | [PAPD5](http://genome-www4.stanford.edu/cgi-bin/SMD/source/sourceResult?choice=Gene&option=Name&criteria=PAPD5) | 0.63 | 0.0080 |
| A_24_P49657 | [ENST00000305402](http://genome-www4.stanford.edu/cgi-bin/SMD/source/sourceResult?choice=Gene&option=Name&criteria=ENST00000305402) | 0.66 | 0.0080 |
| A_23_P362719 | [BC054888](http://genome-www4.stanford.edu/cgi-bin/SMD/source/sourceResult?choice=Gene&option=Name&criteria=BC054888) | 0.66 | 0.0080 |
| A_23_P138524 | [CPXM2](http://genome-www4.stanford.edu/cgi-bin/SMD/source/sourceResult?choice=Gene&option=Name&criteria=CPXM2) | 0.58 | 0.0080 |
| A_23_P12730 | [CSTF2T](http://genome-www4.stanford.edu/cgi-bin/SMD/source/sourceResult?choice=Gene&option=Name&criteria=CSTF2T) | 0.44 | 0.0080 |
| A_23_P92520 | [ANP32C](http://genome-www4.stanford.edu/cgi-bin/SMD/source/sourceResult?choice=Gene&option=Name&criteria=ANP32C) | 0.64 | 0.0080 |
| A_23_P43157 | [MYBL1](http://genome-www4.stanford.edu/cgi-bin/SMD/source/sourceResult?choice=Gene&option=Name&criteria=MYBL1) | 0.58 | 0.0080 |
| A_23_P404606 | [LOC153222](http://genome-www4.stanford.edu/cgi-bin/SMD/source/sourceResult?choice=Gene&option=Name&criteria=LOC153222) | 0.61 | 0.0080 |
| A_24_P508946 | [LOC648674](http://genome-www4.stanford.edu/cgi-bin/SMD/source/sourceResult?choice=Gene&option=Name&criteria=LOC648674) | 0.52 | 0.0080 |
| A_32_P227870 | [BC042520](http://genome-www4.stanford.edu/cgi-bin/SMD/source/sourceResult?choice=Gene&option=Name&criteria=BC042520) | 0.53 | 0.0080 |
| A_23_P50146 | [CD33L3](http://genome-www4.stanford.edu/cgi-bin/SMD/source/sourceResult?choice=Gene&option=Name&criteria=CD33L3) | 0.65 | 0.0080 |
| A_24_P1054 | [NFKBIL2](http://genome-www4.stanford.edu/cgi-bin/SMD/source/sourceResult?choice=Gene&option=Name&criteria=NFKBIL2) | 0.62 | 0.0080 |
| A_32_P78295 | [ATOH7](http://genome-www4.stanford.edu/cgi-bin/SMD/source/sourceResult?choice=Gene&option=Name&criteria=ATOH7) | 0.57 | 0.0080 |
| A_24_P48856 | [CBS](http://genome-www4.stanford.edu/cgi-bin/SMD/source/sourceResult?choice=Gene&option=Name&criteria=CBS) | 0.54 | 0.0080 |
| A_32_P49764 | [CX788817](http://genome-www4.stanford.edu/cgi-bin/SMD/source/sourceResult?choice=Gene&option=Name&criteria=CX788817) | 0.50 | 0.0080 |
| A_32_P167705 | [AGBL2](http://genome-www4.stanford.edu/cgi-bin/SMD/source/sourceResult?choice=Gene&option=Name&criteria=AGBL2) | 0.64 | 0.0080 |
| A_32_P835626 | [FBXO34](http://genome-www4.stanford.edu/cgi-bin/SMD/source/sourceResult?choice=Gene&option=Name&criteria=FBXO34) | 0.66 | 0.0080 |
| A_24_P38754 | [ENST00000333926](http://genome-www4.stanford.edu/cgi-bin/SMD/source/sourceResult?choice=Gene&option=Name&criteria=ENST00000333926) | 0.61 | 0.0080 |
| A_23_P152047 | [SCAMP5](http://genome-www4.stanford.edu/cgi-bin/SMD/source/sourceResult?choice=Gene&option=Name&criteria=SCAMP5) | 0.60 | 0.0080 |
| A_23_P66854 | [KRT20](http://genome-www4.stanford.edu/cgi-bin/SMD/source/sourceResult?choice=Gene&option=Name&criteria=KRT20) | 0.57 | 0.0080 |
| A_32_P204239 | [THC2404488](http://genome-www4.stanford.edu/cgi-bin/SMD/source/sourceResult?choice=Gene&option=Name&criteria=THC2404488) | 0.56 | 0.0080 |
| A_23_P370651 | [FAM13A1](http://genome-www4.stanford.edu/cgi-bin/SMD/source/sourceResult?choice=Gene&option=Name&criteria=FAM13A1) | 0.64 | 0.0080 |
| A_23_P408271 | [DHRS8](http://genome-www4.stanford.edu/cgi-bin/SMD/source/sourceResult?choice=Gene&option=Name&criteria=DHRS8) | 0.60 | 0.0080 |
| A_23_P202004 | [PRTFDC1](http://genome-www4.stanford.edu/cgi-bin/SMD/source/sourceResult?choice=Gene&option=Name&criteria=PRTFDC1) | 0.65 | 0.0080 |
| A_23_P36865 | [CEP290](http://genome-www4.stanford.edu/cgi-bin/SMD/source/sourceResult?choice=Gene&option=Name&criteria=CEP290) | 0.64 | 0.0080 |
| A_23_P258463 | [PROM1](http://genome-www4.stanford.edu/cgi-bin/SMD/source/sourceResult?choice=Gene&option=Name&criteria=PROM1) | 0.62 | 0.0080 |
| A_24_P639679 | [AK095831](http://genome-www4.stanford.edu/cgi-bin/SMD/source/sourceResult?choice=Gene&option=Name&criteria=AK095831) | 0.63 | 0.0080 |
| A_24_P725998 | [THC2460656](http://genome-www4.stanford.edu/cgi-bin/SMD/source/sourceResult?choice=Gene&option=Name&criteria=THC2460656) | 0.46 | 0.0080 |
| A_23_P115022 | [TMEM125](http://genome-www4.stanford.edu/cgi-bin/SMD/source/sourceResult?choice=Gene&option=Name&criteria=TMEM125) | 0.65 | 0.0080 |
| A_23_P55448 | [KRT12](http://genome-www4.stanford.edu/cgi-bin/SMD/source/sourceResult?choice=Gene&option=Name&criteria=KRT12) | 0.56 | 0.0080 |
| A_23_P98092 | [OAT](http://genome-www4.stanford.edu/cgi-bin/SMD/source/sourceResult?choice=Gene&option=Name&criteria=OAT) | 0.43 | 0.0080 |
| A_23_P110569 | [TRIM36](http://genome-www4.stanford.edu/cgi-bin/SMD/source/sourceResult?choice=Gene&option=Name&criteria=TRIM36) | 0.61 | 0.0080 |
| A_23_P359655 | [ZNF664](http://genome-www4.stanford.edu/cgi-bin/SMD/source/sourceResult?choice=Gene&option=Name&criteria=ZNF664) | 0.66 | 0.0080 |
| A_23_P107981 | [SULT2B1](http://genome-www4.stanford.edu/cgi-bin/SMD/source/sourceResult?choice=Gene&option=Name&criteria=SULT2B1) | 0.56 | 0.0080 |
| A_24_P226210 | [CCDC100](http://genome-www4.stanford.edu/cgi-bin/SMD/source/sourceResult?choice=Gene&option=Name&criteria=CCDC100) | 0.63 | 0.0080 |
| A_23_P155688 | [SPINK2](http://genome-www4.stanford.edu/cgi-bin/SMD/source/sourceResult?choice=Gene&option=Name&criteria=SPINK2) | 0.62 | 0.0080 |
| A_24_P333326 | [CTAGE5](http://genome-www4.stanford.edu/cgi-bin/SMD/source/sourceResult?choice=Gene&option=Name&criteria=CTAGE5) | 0.61 | 0.0080 |
| A_32_P113066 | [LRAT](http://genome-www4.stanford.edu/cgi-bin/SMD/source/sourceResult?choice=Gene&option=Name&criteria=LRAT) | 0.35 | 0.0080 |
| A_23_P302914 | [ZFYVE28](http://genome-www4.stanford.edu/cgi-bin/SMD/source/sourceResult?choice=Gene&option=Name&criteria=ZFYVE28) | 0.66 | 0.0080 |
| A_24_P913828 | [MUC3A](http://genome-www4.stanford.edu/cgi-bin/SMD/source/sourceResult?choice=Gene&option=Name&criteria=MUC3A) | 0.62 | 0.0080 |
| A_23_P156076 | [AGXT2](http://genome-www4.stanford.edu/cgi-bin/SMD/source/sourceResult?choice=Gene&option=Name&criteria=AGXT2) | 0.48 | 0.0080 |
| A_23_P257111 | [FBP1](http://genome-www4.stanford.edu/cgi-bin/SMD/source/sourceResult?choice=Gene&option=Name&criteria=FBP1) | 0.60 | 0.0080 |
| A_23_P14708 | [SUHW4](http://genome-www4.stanford.edu/cgi-bin/SMD/source/sourceResult?choice=Gene&option=Name&criteria=SUHW4) | 0.62 | 0.0080 |
| A_24_P366107 | [DNA2L](http://genome-www4.stanford.edu/cgi-bin/SMD/source/sourceResult?choice=Gene&option=Name&criteria=DNA2L) | 0.66 | 0.0080 |
| A_24_P373475 | [ASPA](http://genome-www4.stanford.edu/cgi-bin/SMD/source/sourceResult?choice=Gene&option=Name&criteria=ASPA) | 0.43 | 0.0080 |
| A_23_P73297 | [MAGI1](http://genome-www4.stanford.edu/cgi-bin/SMD/source/sourceResult?choice=Gene&option=Name&criteria=MAGI1) | 0.65 | 0.0098 |
| A_24_P129341 | [AKR1B10](http://genome-www4.stanford.edu/cgi-bin/SMD/source/sourceResult?choice=Gene&option=Name&criteria=AKR1B10) | 0.61 | 0.0098 |
| A_23_P500501 | [FGFR3](http://genome-www4.stanford.edu/cgi-bin/SMD/source/sourceResult?choice=Gene&option=Name&criteria=FGFR3) | 0.66 | 0.0098 |
| A_24_P385313 | [PTPRF](http://genome-www4.stanford.edu/cgi-bin/SMD/source/sourceResult?choice=Gene&option=Name&criteria=PTPRF) | 0.66 | 0.0098 |
| A_23_P501624 | [UGT2B17](http://genome-www4.stanford.edu/cgi-bin/SMD/source/sourceResult?choice=Gene&option=Name&criteria=UGT2B17) | 0.35 | 0.0098 |
| A_23_P404536 | [ENPP3](http://genome-www4.stanford.edu/cgi-bin/SMD/source/sourceResult?choice=Gene&option=Name&criteria=ENPP3) | 0.56 | 0.0098 |
| A_24_P167877 | [LOC440353](http://genome-www4.stanford.edu/cgi-bin/SMD/source/sourceResult?choice=Gene&option=Name&criteria=LOC440353) | 0.64 | 0.0098 |
| A_23_P163390 | [CHRNA7](http://genome-www4.stanford.edu/cgi-bin/SMD/source/sourceResult?choice=Gene&option=Name&criteria=CHRNA7) | 0.64 | 0.0098 |
| A_23_P331813 | [ZNF687](http://genome-www4.stanford.edu/cgi-bin/SMD/source/sourceResult?choice=Gene&option=Name&criteria=ZNF687) | 0.58 | 0.0098 |
| A_23_P114423 | [RGN](http://genome-www4.stanford.edu/cgi-bin/SMD/source/sourceResult?choice=Gene&option=Name&criteria=RGN) | 0.59 | 0.0098 |
| A_32_P44568 | [LDHA](http://genome-www4.stanford.edu/cgi-bin/SMD/source/sourceResult?choice=Gene&option=Name&criteria=LDHA) | 0.64 | 0.0098 |
| A_23_P129064 | [GATM](http://genome-www4.stanford.edu/cgi-bin/SMD/source/sourceResult?choice=Gene&option=Name&criteria=GATM) | 0.53 | 0.0098 |
| A_24_P316046 | [ENST00000342584](http://genome-www4.stanford.edu/cgi-bin/SMD/source/sourceResult?choice=Gene&option=Name&criteria=ENST00000342584) | 0.58 | 0.0098 |
| A_32_P313405 | [LAMA1](http://genome-www4.stanford.edu/cgi-bin/SMD/source/sourceResult?choice=Gene&option=Name&criteria=LAMA1) | 0.61 | 0.0098 |
| A_23_P81721 | [SLC25A27](http://genome-www4.stanford.edu/cgi-bin/SMD/source/sourceResult?choice=Gene&option=Name&criteria=SLC25A27) | 0.63 | 0.0098 |
| A_24_P26792 | [TRPM6](http://genome-www4.stanford.edu/cgi-bin/SMD/source/sourceResult?choice=Gene&option=Name&criteria=TRPM6) | 0.60 | 0.0098 |
| A_23_P118065 | [HSD17B2](http://genome-www4.stanford.edu/cgi-bin/SMD/source/sourceResult?choice=Gene&option=Name&criteria=HSD17B2) | 0.65 | 0.0098 |
| A_24_P290585 | [UACA](http://genome-www4.stanford.edu/cgi-bin/SMD/source/sourceResult?choice=Gene&option=Name&criteria=UACA) | 0.61 | 0.0098 |
| A_23_P93641 | [AKR1B10](http://genome-www4.stanford.edu/cgi-bin/SMD/source/sourceResult?choice=Gene&option=Name&criteria=AKR1B10) | 0.63 | 0.0098 |
| A_23_P36187 | [SYT8](http://genome-www4.stanford.edu/cgi-bin/SMD/source/sourceResult?choice=Gene&option=Name&criteria=SYT8) | 0.59 | 0.0098 |
| A_23_P100754 | [SMURF2](http://genome-www4.stanford.edu/cgi-bin/SMD/source/sourceResult?choice=Gene&option=Name&criteria=SMURF2) | 0.61 | 0.0098 |
| A_23_P17438 | [EDN3](http://genome-www4.stanford.edu/cgi-bin/SMD/source/sourceResult?choice=Gene&option=Name&criteria=EDN3) | 0.61 | 0.0098 |
| A_23_P105803 | [FGF9](http://genome-www4.stanford.edu/cgi-bin/SMD/source/sourceResult?choice=Gene&option=Name&criteria=FGF9) | 0.67 | 0.0098 |
| A_23_P162288 | [MYO1A](http://genome-www4.stanford.edu/cgi-bin/SMD/source/sourceResult?choice=Gene&option=Name&criteria=MYO1A) | 0.64 | 0.0098 |
| A_23_P354341 | [CD160](http://genome-www4.stanford.edu/cgi-bin/SMD/source/sourceResult?choice=Gene&option=Name&criteria=CD160) | 0.67 | 0.0098 |
| A_23_P392544 | [CTAGE3](http://genome-www4.stanford.edu/cgi-bin/SMD/source/sourceResult?choice=Gene&option=Name&criteria=CTAGE3) | 0.66 | 0.0098 |
| A_23_P69791 | [C4orf16](http://genome-www4.stanford.edu/cgi-bin/SMD/source/sourceResult?choice=Gene&option=Name&criteria=C4orf16) | 0.56 | 0.0098 |
| A_24_P118247 | [LOC348180](http://genome-www4.stanford.edu/cgi-bin/SMD/source/sourceResult?choice=Gene&option=Name&criteria=LOC348180) | 0.61 | 0.0098 |
| A_23_P209527 | [THC2310298](http://genome-www4.stanford.edu/cgi-bin/SMD/source/sourceResult?choice=Gene&option=Name&criteria=THC2310298) | 0.63 | 0.0098 |
| A_23_P163567 | [SMPD3](http://genome-www4.stanford.edu/cgi-bin/SMD/source/sourceResult?choice=Gene&option=Name&criteria=SMPD3) | 0.57 | 0.0098 |
| A_24_P105564 | [PRKAB2](http://genome-www4.stanford.edu/cgi-bin/SMD/source/sourceResult?choice=Gene&option=Name&criteria=PRKAB2) | 0.63 | 0.0098 |
| A_23_P430842 | [HAPLN4](http://genome-www4.stanford.edu/cgi-bin/SMD/source/sourceResult?choice=Gene&option=Name&criteria=HAPLN4) | 0.52 | 0.0098 |
| A_23_P104819 | [TREH](http://genome-www4.stanford.edu/cgi-bin/SMD/source/sourceResult?choice=Gene&option=Name&criteria=TREH) | 0.51 | 0.0098 |
| A_32_P132276 | [BE091362](http://genome-www4.stanford.edu/cgi-bin/SMD/source/sourceResult?choice=Gene&option=Name&criteria=BE091362) | 0.67 | 0.0098 |
| A_23_P69908 | [GLRX](http://genome-www4.stanford.edu/cgi-bin/SMD/source/sourceResult?choice=Gene&option=Name&criteria=GLRX) | 0.66 | 0.0098 |
| A_32_P211363 | [CTGLF1](http://genome-www4.stanford.edu/cgi-bin/SMD/source/sourceResult?choice=Gene&option=Name&criteria=CTGLF1) | 0.67 | 0.0098 |
| A_23_P27005 | [MGC4172](http://genome-www4.stanford.edu/cgi-bin/SMD/source/sourceResult?choice=Gene&option=Name&criteria=MGC4172) | 0.64 | 0.0098 |
| A_23_P74900 | [ESRRG](http://genome-www4.stanford.edu/cgi-bin/SMD/source/sourceResult?choice=Gene&option=Name&criteria=ESRRG) | 0.47 | 0.0098 |
| A_23_P425925 | [KA21](http://genome-www4.stanford.edu/cgi-bin/SMD/source/sourceResult?choice=Gene&option=Name&criteria=KA21) | 0.62 | 0.0124 |
| A_32_P220762 | [AK123248](http://genome-www4.stanford.edu/cgi-bin/SMD/source/sourceResult?choice=Gene&option=Name&criteria=AK123248) | 0.59 | 0.0124 |
| A_23_P30175 | [ERBB2IP](http://genome-www4.stanford.edu/cgi-bin/SMD/source/sourceResult?choice=Gene&option=Name&criteria=ERBB2IP) | 0.65 | 0.0124 |
| A_23_P65629 | [KCNK10](http://genome-www4.stanford.edu/cgi-bin/SMD/source/sourceResult?choice=Gene&option=Name&criteria=KCNK10) | 0.65 | 0.0124 |
| A_23_P309207 | [ZNF577](http://genome-www4.stanford.edu/cgi-bin/SMD/source/sourceResult?choice=Gene&option=Name&criteria=ZNF577) | 0.66 | 0.0124 |
| A_23_P33583 | [DNAH7](http://genome-www4.stanford.edu/cgi-bin/SMD/source/sourceResult?choice=Gene&option=Name&criteria=DNAH7) | 0.67 | 0.0124 |
| A_23_P58993 | [MOCS1](http://genome-www4.stanford.edu/cgi-bin/SMD/source/sourceResult?choice=Gene&option=Name&criteria=MOCS1) | 0.61 | 0.0124 |
| A_32_P121978 | [THC2346243](http://genome-www4.stanford.edu/cgi-bin/SMD/source/sourceResult?choice=Gene&option=Name&criteria=THC2346243) | 0.64 | 0.0124 |
| A_24_P636882 | [THC2337176](http://genome-www4.stanford.edu/cgi-bin/SMD/source/sourceResult?choice=Gene&option=Name&criteria=THC2337176) | 0.63 | 0.0124 |
| A_32_P201958 | [ENST00000366971](http://genome-www4.stanford.edu/cgi-bin/SMD/source/sourceResult?choice=Gene&option=Name&criteria=ENST00000366971) | 0.52 | 0.0124 |
| A_24_P303080 | [THC2370211](http://genome-www4.stanford.edu/cgi-bin/SMD/source/sourceResult?choice=Gene&option=Name&criteria=THC2370211) | 0.65 | 0.0124 |
| A_24_P937029 | [AI697848](http://genome-www4.stanford.edu/cgi-bin/SMD/source/sourceResult?choice=Gene&option=Name&criteria=AI697848) | 0.63 | 0.0124 |
| A_23_P79591 | [APOB](http://genome-www4.stanford.edu/cgi-bin/SMD/source/sourceResult?choice=Gene&option=Name&criteria=APOB) | 0.45 | 0.0124 |
| A_24_P910381 | [THC2428320](http://genome-www4.stanford.edu/cgi-bin/SMD/source/sourceResult?choice=Gene&option=Name&criteria=THC2428320) | 0.61 | 0.0124 |
| A_23_P148969 | [LRRC40](http://genome-www4.stanford.edu/cgi-bin/SMD/source/sourceResult?choice=Gene&option=Name&criteria=LRRC40) | 0.63 | 0.0124 |
| A_23_P112162 | [DGAT1](http://genome-www4.stanford.edu/cgi-bin/SMD/source/sourceResult?choice=Gene&option=Name&criteria=DGAT1) | 0.61 | 0.0124 |
| A_32_P169679 | [LCORL](http://genome-www4.stanford.edu/cgi-bin/SMD/source/sourceResult?choice=Gene&option=Name&criteria=LCORL) | 0.63 | 0.0124 |
| A_23_P19894 | [AQP1](http://genome-www4.stanford.edu/cgi-bin/SMD/source/sourceResult?choice=Gene&option=Name&criteria=AQP1) | 0.65 | 0.0124 |
| A_23_P152262 | [DPEP1](http://genome-www4.stanford.edu/cgi-bin/SMD/source/sourceResult?choice=Gene&option=Name&criteria=DPEP1) | 0.50 | 0.0124 |
| A_23_P31041 | [MYLIP](http://genome-www4.stanford.edu/cgi-bin/SMD/source/sourceResult?choice=Gene&option=Name&criteria=MYLIP) | 0.66 | 0.0124 |
| A_23_P349463 | [LOC63928](http://genome-www4.stanford.edu/cgi-bin/SMD/source/sourceResult?choice=Gene&option=Name&criteria=LOC63928) | 0.63 | 0.0124 |
| A_23_P210060 | [MGC13057](http://genome-www4.stanford.edu/cgi-bin/SMD/source/sourceResult?choice=Gene&option=Name&criteria=MGC13057) | 0.61 | 0.0124 |
| A_23_P155123 | [CYP2D6](http://genome-www4.stanford.edu/cgi-bin/SMD/source/sourceResult?choice=Gene&option=Name&criteria=CYP2D6) | 0.51 | 0.0124 |
| A_23_P27107 | [TM4SF5](http://genome-www4.stanford.edu/cgi-bin/SMD/source/sourceResult?choice=Gene&option=Name&criteria=TM4SF5) | 0.61 | 0.0124 |
| A_24_P934704 | [A_24_P934704](http://genome-www4.stanford.edu/cgi-bin/SMD/source/sourceResult?choice=Gene&option=Name&criteria=A_24_P934704) | 0.58 | 0.0124 |
| A_23_P67367 | [DHDH](http://genome-www4.stanford.edu/cgi-bin/SMD/source/sourceResult?choice=Gene&option=Name&criteria=DHDH) | 0.49 | 0.0124 |
| A_23_P210675 | [SYCP2](http://genome-www4.stanford.edu/cgi-bin/SMD/source/sourceResult?choice=Gene&option=Name&criteria=SYCP2) | 0.66 | 0.0124 |
| A_24_P759477 | [ITGB8](http://genome-www4.stanford.edu/cgi-bin/SMD/source/sourceResult?choice=Gene&option=Name&criteria=ITGB8) | 0.58 | 0.0124 |
| A_23_P216556 | [EPB41L4B](http://genome-www4.stanford.edu/cgi-bin/SMD/source/sourceResult?choice=Gene&option=Name&criteria=EPB41L4B) | 0.63 | 0.0124 |
| A_24_P453970 | [THC2284081](http://genome-www4.stanford.edu/cgi-bin/SMD/source/sourceResult?choice=Gene&option=Name&criteria=THC2284081) | 0.52 | 0.0124 |
| A_24_P915095 | [GRAMD1C](http://genome-www4.stanford.edu/cgi-bin/SMD/source/sourceResult?choice=Gene&option=Name&criteria=GRAMD1C) | 0.60 | 0.0124 |
| A_23_P11968 | [GUCA2A](http://genome-www4.stanford.edu/cgi-bin/SMD/source/sourceResult?choice=Gene&option=Name&criteria=GUCA2A) | 0.60 | 0.0124 |
| A_24_P300394 | [GSTA2](http://genome-www4.stanford.edu/cgi-bin/SMD/source/sourceResult?choice=Gene&option=Name&criteria=GSTA2) | 0.53 | 0.0124 |
| A_23_P385126 | [DEPDC7](http://genome-www4.stanford.edu/cgi-bin/SMD/source/sourceResult?choice=Gene&option=Name&criteria=DEPDC7) | 0.55 | 0.0124 |
| A_24_P303052 | [PPARGC1A](http://genome-www4.stanford.edu/cgi-bin/SMD/source/sourceResult?choice=Gene&option=Name&criteria=PPARGC1A) | 0.59 | 0.0124 |
| A_23_P112481 | [AQP3](http://genome-www4.stanford.edu/cgi-bin/SMD/source/sourceResult?choice=Gene&option=Name&criteria=AQP3) | 0.60 | 0.0124 |
| A_24_P419300 | [ENST00000342584](http://genome-www4.stanford.edu/cgi-bin/SMD/source/sourceResult?choice=Gene&option=Name&criteria=ENST00000342584) | 0.61 | 0.0124 |
| A_23_P15692 | [GPR172B](http://genome-www4.stanford.edu/cgi-bin/SMD/source/sourceResult?choice=Gene&option=Name&criteria=GPR172B) | 0.62 | 0.0124 |
| A_24_P340036 | [RNF128](http://genome-www4.stanford.edu/cgi-bin/SMD/source/sourceResult?choice=Gene&option=Name&criteria=RNF128) | 0.60 | 0.0124 |
| A_32_P78285 | [LOC649542](http://genome-www4.stanford.edu/cgi-bin/SMD/source/sourceResult?choice=Gene&option=Name&criteria=LOC649542) | 0.65 | 0.0124 |
| A_32_P191735 | [CK820941](http://genome-www4.stanford.edu/cgi-bin/SMD/source/sourceResult?choice=Gene&option=Name&criteria=CK820941) | 0.65 | 0.0124 |
| A_24_P594721 | [BC062753](http://genome-www4.stanford.edu/cgi-bin/SMD/source/sourceResult?choice=Gene&option=Name&criteria=BC062753) | 0.66 | 0.0124 |
| A_24_P935852 | [A_24_P935852](http://genome-www4.stanford.edu/cgi-bin/SMD/source/sourceResult?choice=Gene&option=Name&criteria=A_24_P935852) | 0.49 | 0.0124 |
| A_23_P212061 | [MME](http://genome-www4.stanford.edu/cgi-bin/SMD/source/sourceResult?choice=Gene&option=Name&criteria=MME) | 0.51 | 0.0124 |
| A_23_P164436 | [ASPA](http://genome-www4.stanford.edu/cgi-bin/SMD/source/sourceResult?choice=Gene&option=Name&criteria=ASPA) | 0.52 | 0.0124 |
| A_23_P205408 | [MIA2](http://genome-www4.stanford.edu/cgi-bin/SMD/source/sourceResult?choice=Gene&option=Name&criteria=MIA2) | 0.51 | 0.0124 |
| A_24_P238525 | [ENST00000324709](http://genome-www4.stanford.edu/cgi-bin/SMD/source/sourceResult?choice=Gene&option=Name&criteria=ENST00000324709) | 0.66 | 0.0124 |
| A_32_P72541 | [BF803942](http://genome-www4.stanford.edu/cgi-bin/SMD/source/sourceResult?choice=Gene&option=Name&criteria=BF803942) | 0.65 | 0.0124 |
| A_24_P541576 | [LOC389831](http://genome-www4.stanford.edu/cgi-bin/SMD/source/sourceResult?choice=Gene&option=Name&criteria=LOC389831) | 0.61 | 0.0124 |
| A_23_P342641 | [SLC44A5](http://genome-www4.stanford.edu/cgi-bin/SMD/source/sourceResult?choice=Gene&option=Name&criteria=SLC44A5) | 0.63 | 0.0124 |
| A_23_P52121 | [PDZK1](http://genome-www4.stanford.edu/cgi-bin/SMD/source/sourceResult?choice=Gene&option=Name&criteria=PDZK1) | 0.47 | 0.0124 |
| A_23_P208706 | [BAX](http://genome-www4.stanford.edu/cgi-bin/SMD/source/sourceResult?choice=Gene&option=Name&criteria=BAX) | 0.64 | 0.0124 |
| A_32_P205637 | [PARD6B](http://genome-www4.stanford.edu/cgi-bin/SMD/source/sourceResult?choice=Gene&option=Name&criteria=PARD6B) | 0.63 | 0.0124 |
| A_24_P795662 | [THC2447296](http://genome-www4.stanford.edu/cgi-bin/SMD/source/sourceResult?choice=Gene&option=Name&criteria=THC2447296) | 0.48 | 0.0124 |
| A_23_P21644 | [DHRS8](http://genome-www4.stanford.edu/cgi-bin/SMD/source/sourceResult?choice=Gene&option=Name&criteria=DHRS8) | 0.64 | 0.0124 |
| A_24_P154868 | [MEP1A](http://genome-www4.stanford.edu/cgi-bin/SMD/source/sourceResult?choice=Gene&option=Name&criteria=MEP1A) | 0.61 | 0.0161 |
| A_32_P9737 | [A_32_P9737](http://genome-www4.stanford.edu/cgi-bin/SMD/source/sourceResult?choice=Gene&option=Name&criteria=A_32_P9737) | 0.50 | 0.0161 |
| A_24_P334378 | [UGT2A3](http://genome-www4.stanford.edu/cgi-bin/SMD/source/sourceResult?choice=Gene&option=Name&criteria=UGT2A3) | 0.51 | 0.0161 |
| A_23_P149613 | [FMO1](http://genome-www4.stanford.edu/cgi-bin/SMD/source/sourceResult?choice=Gene&option=Name&criteria=FMO1) | 0.50 | 0.0161 |
| A_24_P98047 | [SLC16A10](http://genome-www4.stanford.edu/cgi-bin/SMD/source/sourceResult?choice=Gene&option=Name&criteria=SLC16A10) | 0.63 | 0.0161 |
| A_32_P139391 | [BI759100](http://genome-www4.stanford.edu/cgi-bin/SMD/source/sourceResult?choice=Gene&option=Name&criteria=BI759100) | 0.66 | 0.0161 |
| A_24_P363134 | [TRIM36](http://genome-www4.stanford.edu/cgi-bin/SMD/source/sourceResult?choice=Gene&option=Name&criteria=TRIM36) | 0.66 | 0.0161 |
| A_23_P327361 | [DMXL2](http://genome-www4.stanford.edu/cgi-bin/SMD/source/sourceResult?choice=Gene&option=Name&criteria=DMXL2) | 0.66 | 0.0161 |
| A_23_P254512 | [EFNA1](http://genome-www4.stanford.edu/cgi-bin/SMD/source/sourceResult?choice=Gene&option=Name&criteria=EFNA1) | 0.62 | 0.0161 |
| A_24_P83758 | [ENST00000292728](http://genome-www4.stanford.edu/cgi-bin/SMD/source/sourceResult?choice=Gene&option=Name&criteria=ENST00000292728) | 0.64 | 0.0161 |
| A_24_P290999 | [TncRNA](http://genome-www4.stanford.edu/cgi-bin/SMD/source/sourceResult?choice=Gene&option=Name&criteria=TncRNA) | 0.65 | 0.0161 |
| A_24_P846755 | [A_24_P846755](http://genome-www4.stanford.edu/cgi-bin/SMD/source/sourceResult?choice=Gene&option=Name&criteria=A_24_P846755) | 0.62 | 0.0161 |
| A_23_P12767 | [CYP2C9](http://genome-www4.stanford.edu/cgi-bin/SMD/source/sourceResult?choice=Gene&option=Name&criteria=CYP2C9) | 0.56 | 0.0161 |
| A_23_P202683 | [MUCDHL](http://genome-www4.stanford.edu/cgi-bin/SMD/source/sourceResult?choice=Gene&option=Name&criteria=MUCDHL) | 0.61 | 0.0161 |
| A_24_P933675 | [ENST00000380749](http://genome-www4.stanford.edu/cgi-bin/SMD/source/sourceResult?choice=Gene&option=Name&criteria=ENST00000380749) | 0.66 | 0.0161 |
| A_23_P88626 | [ANPEP](http://genome-www4.stanford.edu/cgi-bin/SMD/source/sourceResult?choice=Gene&option=Name&criteria=ANPEP) | 0.58 | 0.0161 |
| A_24_P287974 | [CUEDC1](http://genome-www4.stanford.edu/cgi-bin/SMD/source/sourceResult?choice=Gene&option=Name&criteria=CUEDC1) | 0.65 | 0.0161 |
| A_23_P211047 | [BACH1](http://genome-www4.stanford.edu/cgi-bin/SMD/source/sourceResult?choice=Gene&option=Name&criteria=BACH1) | 0.66 | 0.0161 |
| A_23_P93141 | [GSTA5](http://genome-www4.stanford.edu/cgi-bin/SMD/source/sourceResult?choice=Gene&option=Name&criteria=GSTA5) | 0.53 | 0.0161 |
| A_24_P61864 | [CCDC47](http://genome-www4.stanford.edu/cgi-bin/SMD/source/sourceResult?choice=Gene&option=Name&criteria=CCDC47) | 0.66 | 0.0161 |
| A_23_P16866 | [VIL1](http://genome-www4.stanford.edu/cgi-bin/SMD/source/sourceResult?choice=Gene&option=Name&criteria=VIL1) | 0.66 | 0.0161 |
| A_32_P856518 | [C20orf74](http://genome-www4.stanford.edu/cgi-bin/SMD/source/sourceResult?choice=Gene&option=Name&criteria=C20orf74) | 0.50 | 0.0161 |
| A_24_P3005 | [SCN9A](http://genome-www4.stanford.edu/cgi-bin/SMD/source/sourceResult?choice=Gene&option=Name&criteria=SCN9A) | 0.64 | 0.0161 |
| A_23_P325562 | [SLC1A7](http://genome-www4.stanford.edu/cgi-bin/SMD/source/sourceResult?choice=Gene&option=Name&criteria=SLC1A7) | 0.57 | 0.0161 |
| A_23_P131825 | [TNNC2](http://genome-www4.stanford.edu/cgi-bin/SMD/source/sourceResult?choice=Gene&option=Name&criteria=TNNC2) | 0.65 | 0.0161 |
| A_23_P328729 | [KLHL8](http://genome-www4.stanford.edu/cgi-bin/SMD/source/sourceResult?choice=Gene&option=Name&criteria=KLHL8) | 0.61 | 0.0161 |
| A_24_P186664 | [A_24_P186664](http://genome-www4.stanford.edu/cgi-bin/SMD/source/sourceResult?choice=Gene&option=Name&criteria=A_24_P186664) | 0.65 | 0.0161 |
| A_32_P202703 | [LOC389831](http://genome-www4.stanford.edu/cgi-bin/SMD/source/sourceResult?choice=Gene&option=Name&criteria=LOC389831) | 0.64 | 0.0161 |
| A_24_P575267 | [A_24_P575267](http://genome-www4.stanford.edu/cgi-bin/SMD/source/sourceResult?choice=Gene&option=Name&criteria=A_24_P575267) | 0.59 | 0.0161 |
| A_24_P108863 | [SCML1](http://genome-www4.stanford.edu/cgi-bin/SMD/source/sourceResult?choice=Gene&option=Name&criteria=SCML1) | 0.62 | 0.0161 |
| A_24_P762613 | [AK021543](http://genome-www4.stanford.edu/cgi-bin/SMD/source/sourceResult?choice=Gene&option=Name&criteria=AK021543) | 0.58 | 0.0161 |
| A_23_P66311 | [DNASE1](http://genome-www4.stanford.edu/cgi-bin/SMD/source/sourceResult?choice=Gene&option=Name&criteria=DNASE1) | 0.55 | 0.0161 |
| A_23_P167367 | [PITX2](http://genome-www4.stanford.edu/cgi-bin/SMD/source/sourceResult?choice=Gene&option=Name&criteria=PITX2) | 0.49 | 0.0161 |
| A_23_P8801 | [CYP3A5](http://genome-www4.stanford.edu/cgi-bin/SMD/source/sourceResult?choice=Gene&option=Name&criteria=CYP3A5) | 0.60 | 0.0161 |
| A_23_P312901 | [GPR112](http://genome-www4.stanford.edu/cgi-bin/SMD/source/sourceResult?choice=Gene&option=Name&criteria=GPR112) | 0.52 | 0.0161 |
| A_24_P940725 | [ENST00000369239](http://genome-www4.stanford.edu/cgi-bin/SMD/source/sourceResult?choice=Gene&option=Name&criteria=ENST00000369239) | 0.65 | 0.0204 |
| A_23_P170719 | [A_23_P170719](http://genome-www4.stanford.edu/cgi-bin/SMD/source/sourceResult?choice=Gene&option=Name&criteria=A_23_P170719) | 0.59 | 0.0204 |
| A_24_P17691 | [UGT2B17](http://genome-www4.stanford.edu/cgi-bin/SMD/source/sourceResult?choice=Gene&option=Name&criteria=UGT2B17) | 0.50 | 0.0204 |
| A_32_P184220 | [THC2412604](http://genome-www4.stanford.edu/cgi-bin/SMD/source/sourceResult?choice=Gene&option=Name&criteria=THC2412604) | 0.59 | 0.0204 |
| A_23_P80570 | [AADAC](http://genome-www4.stanford.edu/cgi-bin/SMD/source/sourceResult?choice=Gene&option=Name&criteria=AADAC) | 0.44 | 0.0204 |
| A_23_P360340 | [UACA](http://genome-www4.stanford.edu/cgi-bin/SMD/source/sourceResult?choice=Gene&option=Name&criteria=UACA) | 0.64 | 0.0204 |
| A_23_P10025 | [NELL2](http://genome-www4.stanford.edu/cgi-bin/SMD/source/sourceResult?choice=Gene&option=Name&criteria=NELL2) | 0.63 | 0.0204 |
| A_24_P362904 | [PFKFB4](http://genome-www4.stanford.edu/cgi-bin/SMD/source/sourceResult?choice=Gene&option=Name&criteria=PFKFB4) | 0.59 | 0.0204 |
| A_32_P2883 | [THC2269190](http://genome-www4.stanford.edu/cgi-bin/SMD/source/sourceResult?choice=Gene&option=Name&criteria=THC2269190) | 0.63 | 0.0204 |
| A_23_P149998 | [MAWBP](http://genome-www4.stanford.edu/cgi-bin/SMD/source/sourceResult?choice=Gene&option=Name&criteria=MAWBP) | 0.64 | 0.0204 |
| A_23_P131060 | [CYP4F8](http://genome-www4.stanford.edu/cgi-bin/SMD/source/sourceResult?choice=Gene&option=Name&criteria=CYP4F8) | 0.66 | 0.0204 |
| A_24_P867111 | [LOC283177](http://genome-www4.stanford.edu/cgi-bin/SMD/source/sourceResult?choice=Gene&option=Name&criteria=LOC283177) | 0.67 | 0.0204 |
| A_23_P216712 | [TRPM6](http://genome-www4.stanford.edu/cgi-bin/SMD/source/sourceResult?choice=Gene&option=Name&criteria=TRPM6) | 0.59 | 0.0204 |
| A_24_P194313 | [C21orf66](http://genome-www4.stanford.edu/cgi-bin/SMD/source/sourceResult?choice=Gene&option=Name&criteria=C21orf66) | 0.66 | 0.0204 |
| A_23_P140830 | [ELMO3](http://genome-www4.stanford.edu/cgi-bin/SMD/source/sourceResult?choice=Gene&option=Name&criteria=ELMO3) | 0.67 | 0.0204 |
| A_23_P133120 | [TMEM144](http://genome-www4.stanford.edu/cgi-bin/SMD/source/sourceResult?choice=Gene&option=Name&criteria=TMEM144) | 0.63 | 0.0204 |
| A_23_P85008 | [MAOB](http://genome-www4.stanford.edu/cgi-bin/SMD/source/sourceResult?choice=Gene&option=Name&criteria=MAOB) | 0.52 | 0.0204 |
| A_23_P209116 | [CYP4F3](http://genome-www4.stanford.edu/cgi-bin/SMD/source/sourceResult?choice=Gene&option=Name&criteria=CYP4F3) | 0.57 | 0.0204 |
| A_32_P96748 | [CR744880](http://genome-www4.stanford.edu/cgi-bin/SMD/source/sourceResult?choice=Gene&option=Name&criteria=CR744880) | 0.59 | 0.0204 |
| A_23_P377141 | [VPS13A](http://genome-www4.stanford.edu/cgi-bin/SMD/source/sourceResult?choice=Gene&option=Name&criteria=VPS13A) | 0.64 | 0.0204 |
| A_23_P328836 | [LCOR](http://genome-www4.stanford.edu/cgi-bin/SMD/source/sourceResult?choice=Gene&option=Name&criteria=LCOR) | 0.66 | 0.0204 |
| A_23_P324718 | [SYNJ1](http://genome-www4.stanford.edu/cgi-bin/SMD/source/sourceResult?choice=Gene&option=Name&criteria=SYNJ1) | 0.65 | 0.0204 |
| A_24_P548453 | [ENST00000370871](http://genome-www4.stanford.edu/cgi-bin/SMD/source/sourceResult?choice=Gene&option=Name&criteria=ENST00000370871) | 0.61 | 0.0204 |
| A_24_P912799 | [ENST00000382496](http://genome-www4.stanford.edu/cgi-bin/SMD/source/sourceResult?choice=Gene&option=Name&criteria=ENST00000382496) | 0.66 | 0.0204 |
| A_24_P277875 | [CHN2](http://genome-www4.stanford.edu/cgi-bin/SMD/source/sourceResult?choice=Gene&option=Name&criteria=CHN2) | 0.67 | 0.0204 |
| A_24_P500584 | [XIST](http://genome-www4.stanford.edu/cgi-bin/SMD/source/sourceResult?choice=Gene&option=Name&criteria=XIST) | 0.29 | 0.0204 |
| A_23_P42897 | [MGAM](http://genome-www4.stanford.edu/cgi-bin/SMD/source/sourceResult?choice=Gene&option=Name&criteria=MGAM) | 0.55 | 0.0204 |
| A_23_P135132 | [FRMD3](http://genome-www4.stanford.edu/cgi-bin/SMD/source/sourceResult?choice=Gene&option=Name&criteria=FRMD3) | 0.59 | 0.0204 |
| A_23_P343719 | [PLCB3](http://genome-www4.stanford.edu/cgi-bin/SMD/source/sourceResult?choice=Gene&option=Name&criteria=PLCB3) | 0.63 | 0.0204 |
| A_32_P228341 | [LOC149703](http://genome-www4.stanford.edu/cgi-bin/SMD/source/sourceResult?choice=Gene&option=Name&criteria=LOC149703) | 0.52 | 0.0204 |
| A_24_P940197 | [PAPD5](http://genome-www4.stanford.edu/cgi-bin/SMD/source/sourceResult?choice=Gene&option=Name&criteria=PAPD5) | 0.63 | 0.0204 |
| A_24_P409182 | [ENST00000303979](http://genome-www4.stanford.edu/cgi-bin/SMD/source/sourceResult?choice=Gene&option=Name&criteria=ENST00000303979) | 0.50 | 0.0204 |
| A_32_P214011 | [THC2277187](http://genome-www4.stanford.edu/cgi-bin/SMD/source/sourceResult?choice=Gene&option=Name&criteria=THC2277187) | 0.65 | 0.0204 |
| A_23_P328740 | [LINCR](http://genome-www4.stanford.edu/cgi-bin/SMD/source/sourceResult?choice=Gene&option=Name&criteria=LINCR) | 0.65 | 0.0204 |
| A_32_P199998 | [C10orf75](http://genome-www4.stanford.edu/cgi-bin/SMD/source/sourceResult?choice=Gene&option=Name&criteria=C10orf75) | 0.65 | 0.0204 |
| A_32_P224840 | [FTS](http://genome-www4.stanford.edu/cgi-bin/SMD/source/sourceResult?choice=Gene&option=Name&criteria=FTS) | 0.65 | 0.0204 |
| A_23_P211909 | [PLS1](http://genome-www4.stanford.edu/cgi-bin/SMD/source/sourceResult?choice=Gene&option=Name&criteria=PLS1) | 0.61 | 0.0204 |
| A_23_P163238 | [STRC](http://genome-www4.stanford.edu/cgi-bin/SMD/source/sourceResult?choice=Gene&option=Name&criteria=STRC) | 0.64 | 0.0204 |
| A_24_P200831 | [MLXIPL](http://genome-www4.stanford.edu/cgi-bin/SMD/source/sourceResult?choice=Gene&option=Name&criteria=MLXIPL) | 0.58 | 0.0204 |
| A_32_P69333 | [BM717049](http://genome-www4.stanford.edu/cgi-bin/SMD/source/sourceResult?choice=Gene&option=Name&criteria=BM717049) | 0.64 | 0.0204 |
| A_23_P135417 | [GSTA1](http://genome-www4.stanford.edu/cgi-bin/SMD/source/sourceResult?choice=Gene&option=Name&criteria=GSTA1) | 0.53 | 0.0204 |
| A_32_P105397 | [THC2373712](http://genome-www4.stanford.edu/cgi-bin/SMD/source/sourceResult?choice=Gene&option=Name&criteria=THC2373712) | 0.64 | 0.0204 |
| A_23_P117082 | [HEBP1](http://genome-www4.stanford.edu/cgi-bin/SMD/source/sourceResult?choice=Gene&option=Name&criteria=HEBP1) | 0.58 | 0.0204 |
| A_23_P312150 | [EDN2](http://genome-www4.stanford.edu/cgi-bin/SMD/source/sourceResult?choice=Gene&option=Name&criteria=EDN2) | 0.56 | 0.0204 |
| A_32_P879150 | [THC2278737](http://genome-www4.stanford.edu/cgi-bin/SMD/source/sourceResult?choice=Gene&option=Name&criteria=THC2278737) | 0.55 | 0.0204 |
| A_32_P146635 | [CR603982](http://genome-www4.stanford.edu/cgi-bin/SMD/source/sourceResult?choice=Gene&option=Name&criteria=CR603982) | 0.63 | 0.0242 |
| A_24_P924816 | [SLC2A13](http://genome-www4.stanford.edu/cgi-bin/SMD/source/sourceResult?choice=Gene&option=Name&criteria=SLC2A13) | 0.62 | 0.0242 |
| A_24_P921897 | [HOOK1](http://genome-www4.stanford.edu/cgi-bin/SMD/source/sourceResult?choice=Gene&option=Name&criteria=HOOK1) | 0.65 | 0.0242 |
| A_23_P83436 | [PEPD](http://genome-www4.stanford.edu/cgi-bin/SMD/source/sourceResult?choice=Gene&option=Name&criteria=PEPD) | 0.58 | 0.0242 |
| A_24_P89426 | [APOM](http://genome-www4.stanford.edu/cgi-bin/SMD/source/sourceResult?choice=Gene&option=Name&criteria=APOM) | 0.61 | 0.0242 |
| A_23_P358917 | [CYP3A7](http://genome-www4.stanford.edu/cgi-bin/SMD/source/sourceResult?choice=Gene&option=Name&criteria=CYP3A7) | 0.56 | 0.0242 |
| A_23_P252236 | [KLKB1](http://genome-www4.stanford.edu/cgi-bin/SMD/source/sourceResult?choice=Gene&option=Name&criteria=KLKB1) | 0.64 | 0.0242 |
| A_23_P108823 | [OSBPL6](http://genome-www4.stanford.edu/cgi-bin/SMD/source/sourceResult?choice=Gene&option=Name&criteria=OSBPL6) | 0.65 | 0.0242 |
| A_24_P410776 | [DNAH7](http://genome-www4.stanford.edu/cgi-bin/SMD/source/sourceResult?choice=Gene&option=Name&criteria=DNAH7) | 0.57 | 0.0242 |
| A_32_P31827 | [THC2279918](http://genome-www4.stanford.edu/cgi-bin/SMD/source/sourceResult?choice=Gene&option=Name&criteria=THC2279918) | 0.66 | 0.0242 |
| A_23_P12928 | [SLC5A12](http://genome-www4.stanford.edu/cgi-bin/SMD/source/sourceResult?choice=Gene&option=Name&criteria=SLC5A12) | 0.49 | 0.0242 |
| A_23_P133338 | [PCLKC](http://genome-www4.stanford.edu/cgi-bin/SMD/source/sourceResult?choice=Gene&option=Name&criteria=PCLKC) | 0.58 | 0.0242 |
| A_32_P177040 | [WBSCR19](http://genome-www4.stanford.edu/cgi-bin/SMD/source/sourceResult?choice=Gene&option=Name&criteria=WBSCR19) | 0.65 | 0.0242 |
| A_24_P340112 | [THC2405729](http://genome-www4.stanford.edu/cgi-bin/SMD/source/sourceResult?choice=Gene&option=Name&criteria=THC2405729) | 0.65 | 0.0242 |
| A_23_P308800 | [GLS](http://genome-www4.stanford.edu/cgi-bin/SMD/source/sourceResult?choice=Gene&option=Name&criteria=GLS) | 0.66 | 0.0242 |
| A_24_P695306 | [LOC402689](http://genome-www4.stanford.edu/cgi-bin/SMD/source/sourceResult?choice=Gene&option=Name&criteria=LOC402689) | 0.55 | 0.0242 |
| A_23_P139146 | [MS4A8B](http://genome-www4.stanford.edu/cgi-bin/SMD/source/sourceResult?choice=Gene&option=Name&criteria=MS4A8B) | 0.65 | 0.0242 |
| A_23_P20713 | [C8G](http://genome-www4.stanford.edu/cgi-bin/SMD/source/sourceResult?choice=Gene&option=Name&criteria=C8G) | 0.62 | 0.0242 |
| A_23_P252981 | [ACE2](http://genome-www4.stanford.edu/cgi-bin/SMD/source/sourceResult?choice=Gene&option=Name&criteria=ACE2) | 0.51 | 0.0242 |
| A_23_P143734 | [CYP2D6](http://genome-www4.stanford.edu/cgi-bin/SMD/source/sourceResult?choice=Gene&option=Name&criteria=CYP2D6) | 0.62 | 0.0242 |
| A_23_P29257 | [H1F0](http://genome-www4.stanford.edu/cgi-bin/SMD/source/sourceResult?choice=Gene&option=Name&criteria=H1F0) | 0.67 | 0.0242 |
| A_32_P158272 | [AL359055](http://genome-www4.stanford.edu/cgi-bin/SMD/source/sourceResult?choice=Gene&option=Name&criteria=AL359055) | 0.65 | 0.0242 |
| A_23_P70813 | [ENST00000366822](http://genome-www4.stanford.edu/cgi-bin/SMD/source/sourceResult?choice=Gene&option=Name&criteria=ENST00000366822) | 0.66 | 0.0242 |
| A_24_P263144 | [BMX](http://genome-www4.stanford.edu/cgi-bin/SMD/source/sourceResult?choice=Gene&option=Name&criteria=BMX) | 0.66 | 0.0242 |
| A_23_P52480 | [CYP2C18](http://genome-www4.stanford.edu/cgi-bin/SMD/source/sourceResult?choice=Gene&option=Name&criteria=CYP2C18) | 0.65 | 0.0242 |
| A_23_P167182 | [LRAT](http://genome-www4.stanford.edu/cgi-bin/SMD/source/sourceResult?choice=Gene&option=Name&criteria=LRAT) | 0.53 | 0.0242 |
| A_23_P20316 | [CA3](http://genome-www4.stanford.edu/cgi-bin/SMD/source/sourceResult?choice=Gene&option=Name&criteria=CA3) | 0.57 | 0.0242 |
| A_23_P331598 | [IPO7](http://genome-www4.stanford.edu/cgi-bin/SMD/source/sourceResult?choice=Gene&option=Name&criteria=IPO7) | 0.65 | 0.0242 |
| A_23_P85015 | [MAOB](http://genome-www4.stanford.edu/cgi-bin/SMD/source/sourceResult?choice=Gene&option=Name&criteria=MAOB) | 0.62 | 0.0242 |
| A_23_P158297 | [BTNL3](http://genome-www4.stanford.edu/cgi-bin/SMD/source/sourceResult?choice=Gene&option=Name&criteria=BTNL3) | 0.65 | 0.0242 |
| A_24_P393571 | [GDA](http://genome-www4.stanford.edu/cgi-bin/SMD/source/sourceResult?choice=Gene&option=Name&criteria=GDA) | 0.62 | 0.0242 |
| A_23_P341065 | [RP3-402G11.5](http://genome-www4.stanford.edu/cgi-bin/SMD/source/sourceResult?choice=Gene&option=Name&criteria=RP3-402G11.5) | 0.66 | 0.0242 |
| A_23_P108514 | [STK16](http://genome-www4.stanford.edu/cgi-bin/SMD/source/sourceResult?choice=Gene&option=Name&criteria=STK16) | 0.65 | 0.0242 |
| A_23_P202275 | [PRAP1](http://genome-www4.stanford.edu/cgi-bin/SMD/source/sourceResult?choice=Gene&option=Name&criteria=PRAP1) | 0.57 | 0.0242 |
| A_24_P511877 | [AK128714](http://genome-www4.stanford.edu/cgi-bin/SMD/source/sourceResult?choice=Gene&option=Name&criteria=AK128714) | 0.58 | 0.0242 |
| A_23_P93122 | [MEP1A](http://genome-www4.stanford.edu/cgi-bin/SMD/source/sourceResult?choice=Gene&option=Name&criteria=MEP1A) | 0.62 | 0.0242 |
| A_23_P117387 | [MIA2](http://genome-www4.stanford.edu/cgi-bin/SMD/source/sourceResult?choice=Gene&option=Name&criteria=MIA2) | 0.58 | 0.0242 |
| A_24_P396980 | [PFN2](http://genome-www4.stanford.edu/cgi-bin/SMD/source/sourceResult?choice=Gene&option=Name&criteria=PFN2) | 0.64 | 0.0242 |
| A_23_P58407 | [UGT2B15](http://genome-www4.stanford.edu/cgi-bin/SMD/source/sourceResult?choice=Gene&option=Name&criteria=UGT2B15) | 0.43 | 0.0242 |
| A_23_P157371 | [FAM3C](http://genome-www4.stanford.edu/cgi-bin/SMD/source/sourceResult?choice=Gene&option=Name&criteria=FAM3C) | 0.60 | 0.0242 |
| A_24_P156922 | [SCP2](http://genome-www4.stanford.edu/cgi-bin/SMD/source/sourceResult?choice=Gene&option=Name&criteria=SCP2) | 0.60 | 0.0242 |
| A_23_P168818 | [IMPA1](http://genome-www4.stanford.edu/cgi-bin/SMD/source/sourceResult?choice=Gene&option=Name&criteria=IMPA1) | 0.65 | 0.0242 |
| A_24_P190424 | [RAB8A](http://genome-www4.stanford.edu/cgi-bin/SMD/source/sourceResult?choice=Gene&option=Name&criteria=RAB8A) | 0.65 | 0.0242 |
| A_23_P87036 | [APOA4](http://genome-www4.stanford.edu/cgi-bin/SMD/source/sourceResult?choice=Gene&option=Name&criteria=APOA4) | 0.53 | 0.0242 |
| A_23_P335388 | [C17orf78](http://genome-www4.stanford.edu/cgi-bin/SMD/source/sourceResult?choice=Gene&option=Name&criteria=C17orf78) | 0.59 | 0.0242 |
| A_23_P21990 | [SLC23A1](http://genome-www4.stanford.edu/cgi-bin/SMD/source/sourceResult?choice=Gene&option=Name&criteria=SLC23A1) | 0.54 | 0.0242 |
| A_32_P81282 | [THC2282190](http://genome-www4.stanford.edu/cgi-bin/SMD/source/sourceResult?choice=Gene&option=Name&criteria=THC2282190) | 0.51 | 0.0242 |
| A_24_P63537 | [ARTS-1](http://genome-www4.stanford.edu/cgi-bin/SMD/source/sourceResult?choice=Gene&option=Name&criteria=ARTS-1) | 0.66 | 0.0242 |
| A_24_P103952 | [DGKZ](http://genome-www4.stanford.edu/cgi-bin/SMD/source/sourceResult?choice=Gene&option=Name&criteria=DGKZ) | 0.64 | 0.0242 |
| A_23_P397376 | [MAF](http://genome-www4.stanford.edu/cgi-bin/SMD/source/sourceResult?choice=Gene&option=Name&criteria=MAF) | 0.57 | 0.0242 |
| A_23_P159325 | [ANGPTL4](http://genome-www4.stanford.edu/cgi-bin/SMD/source/sourceResult?choice=Gene&option=Name&criteria=ANGPTL4) | 0.60 | 0.0296 |
| A_24_P365129 | [THC2364018](http://genome-www4.stanford.edu/cgi-bin/SMD/source/sourceResult?choice=Gene&option=Name&criteria=THC2364018) | 0.53 | 0.0296 |
| A_24_P374427 | [ZDHHC21](http://genome-www4.stanford.edu/cgi-bin/SMD/source/sourceResult?choice=Gene&option=Name&criteria=ZDHHC21) | 0.67 | 0.0296 |
| A_24_P566916 | [TncRNA](http://genome-www4.stanford.edu/cgi-bin/SMD/source/sourceResult?choice=Gene&option=Name&criteria=TncRNA) | 0.61 | 0.0296 |
| A_32_P185741 | [THC2410251](http://genome-www4.stanford.edu/cgi-bin/SMD/source/sourceResult?choice=Gene&option=Name&criteria=THC2410251) | 0.51 | 0.0296 |
| A_32_P309929 | [ENST00000373264](http://genome-www4.stanford.edu/cgi-bin/SMD/source/sourceResult?choice=Gene&option=Name&criteria=ENST00000373264) | 0.66 | 0.0296 |
| A_23_P42265 | [APOM](http://genome-www4.stanford.edu/cgi-bin/SMD/source/sourceResult?choice=Gene&option=Name&criteria=APOM) | 0.64 | 0.0296 |
| A_23_P54736 | [GNG13](http://genome-www4.stanford.edu/cgi-bin/SMD/source/sourceResult?choice=Gene&option=Name&criteria=GNG13) | 0.62 | 0.0296 |
| A_23_P128728 | [ARG2](http://genome-www4.stanford.edu/cgi-bin/SMD/source/sourceResult?choice=Gene&option=Name&criteria=ARG2) | 0.56 | 0.0296 |
| A_24_P852601 | [HNF4G](http://genome-www4.stanford.edu/cgi-bin/SMD/source/sourceResult?choice=Gene&option=Name&criteria=HNF4G) | 0.59 | 0.0296 |
| A_23_P128817 | [PCK2](http://genome-www4.stanford.edu/cgi-bin/SMD/source/sourceResult?choice=Gene&option=Name&criteria=PCK2) | 0.67 | 0.0296 |
| A_23_P430839 | [HAPLN4](http://genome-www4.stanford.edu/cgi-bin/SMD/source/sourceResult?choice=Gene&option=Name&criteria=HAPLN4) | 0.62 | 0.0296 |
| A_23_P366983 | [TRHDE](http://genome-www4.stanford.edu/cgi-bin/SMD/source/sourceResult?choice=Gene&option=Name&criteria=TRHDE) | 0.60 | 0.0296 |
| A_32_P183598 | [LOC645904](http://genome-www4.stanford.edu/cgi-bin/SMD/source/sourceResult?choice=Gene&option=Name&criteria=LOC645904) | 0.61 | 0.0296 |
| A_24_P678741 | [ENST00000358431](http://genome-www4.stanford.edu/cgi-bin/SMD/source/sourceResult?choice=Gene&option=Name&criteria=ENST00000358431) | 0.63 | 0.0296 |
| A_23_P160433 | [C1orf115](http://genome-www4.stanford.edu/cgi-bin/SMD/source/sourceResult?choice=Gene&option=Name&criteria=C1orf115) | 0.66 | 0.0296 |
| A_24_P311845 | [CR612518](http://genome-www4.stanford.edu/cgi-bin/SMD/source/sourceResult?choice=Gene&option=Name&criteria=CR612518) | 0.65 | 0.0296 |
| A_23_P209251 | [THC2316768](http://genome-www4.stanford.edu/cgi-bin/SMD/source/sourceResult?choice=Gene&option=Name&criteria=THC2316768) | 0.60 | 0.0296 |
| A_23_P314805 | [TMEM56](http://genome-www4.stanford.edu/cgi-bin/SMD/source/sourceResult?choice=Gene&option=Name&criteria=TMEM56) | 0.59 | 0.0296 |
| A_32_P180435 | [WBSCR19](http://genome-www4.stanford.edu/cgi-bin/SMD/source/sourceResult?choice=Gene&option=Name&criteria=WBSCR19) | 0.66 | 0.0296 |
| A_23_P372478 | [SERPINA9](http://genome-www4.stanford.edu/cgi-bin/SMD/source/sourceResult?choice=Gene&option=Name&criteria=SERPINA9) | 0.56 | 0.0296 |
| A_23_P105012 | [HRASLS2](http://genome-www4.stanford.edu/cgi-bin/SMD/source/sourceResult?choice=Gene&option=Name&criteria=HRASLS2) | 0.66 | 0.0296 |
| A_23_P71226 | [SLC13A1](http://genome-www4.stanford.edu/cgi-bin/SMD/source/sourceResult?choice=Gene&option=Name&criteria=SLC13A1) | 0.58 | 0.0296 |
| A_32_P131583 | [THC2314886](http://genome-www4.stanford.edu/cgi-bin/SMD/source/sourceResult?choice=Gene&option=Name&criteria=THC2314886) | 0.55 | 0.0296 |
| A_32_P66843 | [THC2340177](http://genome-www4.stanford.edu/cgi-bin/SMD/source/sourceResult?choice=Gene&option=Name&criteria=THC2340177) | 0.54 | 0.0296 |
| A_23_P205074 | [LOC283537](http://genome-www4.stanford.edu/cgi-bin/SMD/source/sourceResult?choice=Gene&option=Name&criteria=LOC283537) | 0.66 | 0.0296 |
| A_23_P304110 | [ANKRD43](http://genome-www4.stanford.edu/cgi-bin/SMD/source/sourceResult?choice=Gene&option=Name&criteria=ANKRD43) | 0.66 | 0.0296 |
| A_23_P112482 | [AQP3](http://genome-www4.stanford.edu/cgi-bin/SMD/source/sourceResult?choice=Gene&option=Name&criteria=AQP3) | 0.63 | 0.0296 |
| A_24_P260101 | [MME](http://genome-www4.stanford.edu/cgi-bin/SMD/source/sourceResult?choice=Gene&option=Name&criteria=MME) | 0.49 | 0.0296 |
| A_23_P413693 | [C21orf129](http://genome-www4.stanford.edu/cgi-bin/SMD/source/sourceResult?choice=Gene&option=Name&criteria=C21orf129) | 0.53 | 0.0296 |
| A_23_P214300 | [GSTA2](http://genome-www4.stanford.edu/cgi-bin/SMD/source/sourceResult?choice=Gene&option=Name&criteria=GSTA2) | 0.57 | 0.0296 |
| A_24_P461998 | [AA889382](http://genome-www4.stanford.edu/cgi-bin/SMD/source/sourceResult?choice=Gene&option=Name&criteria=AA889382) | 0.64 | 0.0296 |
| A_24_P321581 | [SLC38A4](http://genome-www4.stanford.edu/cgi-bin/SMD/source/sourceResult?choice=Gene&option=Name&criteria=SLC38A4) | 0.58 | 0.0296 |
| A_23_P167276 | [PAQR3](http://genome-www4.stanford.edu/cgi-bin/SMD/source/sourceResult?choice=Gene&option=Name&criteria=PAQR3) | 0.65 | 0.0296 |
| A_23_P382302 | [RIF1](http://genome-www4.stanford.edu/cgi-bin/SMD/source/sourceResult?choice=Gene&option=Name&criteria=RIF1) | 0.65 | 0.0296 |
| A_24_P671115 | [BC018675](http://genome-www4.stanford.edu/cgi-bin/SMD/source/sourceResult?choice=Gene&option=Name&criteria=BC018675) | 0.67 | 0.0296 |
| A_23_P397120 | [ENST00000211092](http://genome-www4.stanford.edu/cgi-bin/SMD/source/sourceResult?choice=Gene&option=Name&criteria=ENST00000211092) | 0.62 | 0.0296 |
| A_23_P44207 | [ACOT12](http://genome-www4.stanford.edu/cgi-bin/SMD/source/sourceResult?choice=Gene&option=Name&criteria=ACOT12) | 0.66 | 0.0296 |
| A_23_P131308 | [CYP27A1](http://genome-www4.stanford.edu/cgi-bin/SMD/source/sourceResult?choice=Gene&option=Name&criteria=CYP27A1) | 0.65 | 0.0364 |
| A_24_P10226 | [SEMA6D](http://genome-www4.stanford.edu/cgi-bin/SMD/source/sourceResult?choice=Gene&option=Name&criteria=SEMA6D) | 0.64 | 0.0364 |
| A_23_P78353 | [MEP1B](http://genome-www4.stanford.edu/cgi-bin/SMD/source/sourceResult?choice=Gene&option=Name&criteria=MEP1B) | 0.53 | 0.0364 |
| A_23_P90510 | [REEP6](http://genome-www4.stanford.edu/cgi-bin/SMD/source/sourceResult?choice=Gene&option=Name&criteria=REEP6) | 0.60 | 0.0364 |
| A_23_P501193 | [KCNJ16](http://genome-www4.stanford.edu/cgi-bin/SMD/source/sourceResult?choice=Gene&option=Name&criteria=KCNJ16) | 0.49 | 0.0364 |
| A_23_P108082 | [CREB3L3](http://genome-www4.stanford.edu/cgi-bin/SMD/source/sourceResult?choice=Gene&option=Name&criteria=CREB3L3) | 0.54 | 0.0364 |
| A_24_P253454 | [RGMA](http://genome-www4.stanford.edu/cgi-bin/SMD/source/sourceResult?choice=Gene&option=Name&criteria=RGMA) | 0.55 | 0.0364 |
| A_24_P305223 | [CTAGE1](http://genome-www4.stanford.edu/cgi-bin/SMD/source/sourceResult?choice=Gene&option=Name&criteria=CTAGE1) | 0.67 | 0.0364 |
| A_32_P208200 | [THC2393794](http://genome-www4.stanford.edu/cgi-bin/SMD/source/sourceResult?choice=Gene&option=Name&criteria=THC2393794) | 0.67 | 0.0364 |
| A_23_P5845 | [KHK](http://genome-www4.stanford.edu/cgi-bin/SMD/source/sourceResult?choice=Gene&option=Name&criteria=KHK) | 0.63 | 0.0364 |
| A_24_P53778 | [ITLN2](http://genome-www4.stanford.edu/cgi-bin/SMD/source/sourceResult?choice=Gene&option=Name&criteria=ITLN2) | 0.57 | 0.0364 |
| A_23_P119763 | [ABCG5](http://genome-www4.stanford.edu/cgi-bin/SMD/source/sourceResult?choice=Gene&option=Name&criteria=ABCG5) | 0.53 | 0.0364 |
| A_23_P254756 | [CD164](http://genome-www4.stanford.edu/cgi-bin/SMD/source/sourceResult?choice=Gene&option=Name&criteria=CD164) | 0.65 | 0.0364 |
| A_23_P258493 | [LMNB1](http://genome-www4.stanford.edu/cgi-bin/SMD/source/sourceResult?choice=Gene&option=Name&criteria=LMNB1) | 0.66 | 0.0364 |
| A_23_P145786 | [MLXIPL](http://genome-www4.stanford.edu/cgi-bin/SMD/source/sourceResult?choice=Gene&option=Name&criteria=MLXIPL) | 0.62 | 0.0364 |
| A_23_P312358 | [C10orf30](http://genome-www4.stanford.edu/cgi-bin/SMD/source/sourceResult?choice=Gene&option=Name&criteria=C10orf30) | 0.66 | 0.0364 |
| A_23_P390032 | [TMEM20](http://genome-www4.stanford.edu/cgi-bin/SMD/source/sourceResult?choice=Gene&option=Name&criteria=TMEM20) | 0.64 | 0.0364 |
| A_23_P5778 | [RAB17](http://genome-www4.stanford.edu/cgi-bin/SMD/source/sourceResult?choice=Gene&option=Name&criteria=RAB17) | 0.64 | 0.0364 |
| A_24_P334208 | [A_24_P334208](http://genome-www4.stanford.edu/cgi-bin/SMD/source/sourceResult?choice=Gene&option=Name&criteria=A_24_P334208) | 0.66 | 0.0364 |
| A_32_P13168 | [BX115853](http://genome-www4.stanford.edu/cgi-bin/SMD/source/sourceResult?choice=Gene&option=Name&criteria=BX115853) | 0.60 | 0.0364 |
| A_23_P20427 | [RHOBTB2](http://genome-www4.stanford.edu/cgi-bin/SMD/source/sourceResult?choice=Gene&option=Name&criteria=RHOBTB2) | 0.65 | 0.0364 |
| A_23_P10559 | [AATK](http://genome-www4.stanford.edu/cgi-bin/SMD/source/sourceResult?choice=Gene&option=Name&criteria=AATK) | 0.60 | 0.0364 |
| A_24_P917711 | [PRKAB2](http://genome-www4.stanford.edu/cgi-bin/SMD/source/sourceResult?choice=Gene&option=Name&criteria=PRKAB2) | 0.63 | 0.0364 |
| A_24_P678743 | [ENST00000358431](http://genome-www4.stanford.edu/cgi-bin/SMD/source/sourceResult?choice=Gene&option=Name&criteria=ENST00000358431) | 0.58 | 0.0364 |
| A_23_P136116 | [FLJ16237](http://genome-www4.stanford.edu/cgi-bin/SMD/source/sourceResult?choice=Gene&option=Name&criteria=FLJ16237) | 0.60 | 0.0364 |
| A_24_P7584 | [LY6G5C](http://genome-www4.stanford.edu/cgi-bin/SMD/source/sourceResult?choice=Gene&option=Name&criteria=LY6G5C) | 0.63 | 0.0364 |
| A_23_P154938 | [HIRA](http://genome-www4.stanford.edu/cgi-bin/SMD/source/sourceResult?choice=Gene&option=Name&criteria=HIRA) | 0.60 | 0.0364 |
| A_23_P158481 | [CYP2C19](http://genome-www4.stanford.edu/cgi-bin/SMD/source/sourceResult?choice=Gene&option=Name&criteria=CYP2C19) | 0.60 | 0.0364 |
| A_23_P40611 | [TCN2](http://genome-www4.stanford.edu/cgi-bin/SMD/source/sourceResult?choice=Gene&option=Name&criteria=TCN2) | 0.66 | 0.0364 |
| A_24_P245838 | [AK125361](http://genome-www4.stanford.edu/cgi-bin/SMD/source/sourceResult?choice=Gene&option=Name&criteria=AK125361) | 0.62 | 0.0364 |
| A_23_P218584 | [BCL11A](http://genome-www4.stanford.edu/cgi-bin/SMD/source/sourceResult?choice=Gene&option=Name&criteria=BCL11A) | 0.66 | 0.0405 |
| A_23_P323943 | [SLC5A12](http://genome-www4.stanford.edu/cgi-bin/SMD/source/sourceResult?choice=Gene&option=Name&criteria=SLC5A12) | 0.65 | 0.0405 |
| A_23_P359052 | [FAM44A](http://genome-www4.stanford.edu/cgi-bin/SMD/source/sourceResult?choice=Gene&option=Name&criteria=FAM44A) | 0.64 | 0.0405 |
| A_23_P49192 | [CES2](http://genome-www4.stanford.edu/cgi-bin/SMD/source/sourceResult?choice=Gene&option=Name&criteria=CES2) | 0.66 | 0.0405 |
| A_32_P26103 | [AGPAT2](http://genome-www4.stanford.edu/cgi-bin/SMD/source/sourceResult?choice=Gene&option=Name&criteria=AGPAT2) | 0.60 | 0.0405 |
| A_23_P402313 | [FLJ35424](http://genome-www4.stanford.edu/cgi-bin/SMD/source/sourceResult?choice=Gene&option=Name&criteria=FLJ35424) | 0.62 | 0.0405 |
| A_23_P38235 | [ACE](http://genome-www4.stanford.edu/cgi-bin/SMD/source/sourceResult?choice=Gene&option=Name&criteria=ACE) | 0.66 | 0.0405 |
| A_23_P319572 | [NR1I3](http://genome-www4.stanford.edu/cgi-bin/SMD/source/sourceResult?choice=Gene&option=Name&criteria=NR1I3) | 0.65 | 0.0405 |
| A_23_P390722 | [IFRG15](http://genome-www4.stanford.edu/cgi-bin/SMD/source/sourceResult?choice=Gene&option=Name&criteria=IFRG15) | 0.67 | 0.0405 |
| A_24_P80500 | [BDH2](http://genome-www4.stanford.edu/cgi-bin/SMD/source/sourceResult?choice=Gene&option=Name&criteria=BDH2) | 0.65 | 0.0405 |
| A_32_P182812 | [BX100997](http://genome-www4.stanford.edu/cgi-bin/SMD/source/sourceResult?choice=Gene&option=Name&criteria=BX100997) | 0.49 | 0.0405 |
| A_23_P207905 | [SECTM1](http://genome-www4.stanford.edu/cgi-bin/SMD/source/sourceResult?choice=Gene&option=Name&criteria=SECTM1) | 0.66 | 0.0405 |
| A_24_P935147 | [AF119905](http://genome-www4.stanford.edu/cgi-bin/SMD/source/sourceResult?choice=Gene&option=Name&criteria=AF119905) | 0.54 | 0.0405 |
| A_24_P303454 | [TIAM2](http://genome-www4.stanford.edu/cgi-bin/SMD/source/sourceResult?choice=Gene&option=Name&criteria=TIAM2) | 0.65 | 0.0405 |
| A_23_P253495 | [GSTA3](http://genome-www4.stanford.edu/cgi-bin/SMD/source/sourceResult?choice=Gene&option=Name&criteria=GSTA3) | 0.58 | 0.0405 |
| A_23_P500093 | [RGS13](http://genome-www4.stanford.edu/cgi-bin/SMD/source/sourceResult?choice=Gene&option=Name&criteria=RGS13) | 0.58 | 0.0405 |
| A_24_P379616 | [PLB1](http://genome-www4.stanford.edu/cgi-bin/SMD/source/sourceResult?choice=Gene&option=Name&criteria=PLB1) | 0.60 | 0.0405 |
| A_23_P18672 | [GBA3](http://genome-www4.stanford.edu/cgi-bin/SMD/source/sourceResult?choice=Gene&option=Name&criteria=GBA3) | 0.56 | 0.0405 |
| A_23_P155351 | [BTD](http://genome-www4.stanford.edu/cgi-bin/SMD/source/sourceResult?choice=Gene&option=Name&criteria=BTD) | 0.66 | 0.0405 |
| A_23_P216468 | [SLC1A1](http://genome-www4.stanford.edu/cgi-bin/SMD/source/sourceResult?choice=Gene&option=Name&criteria=SLC1A1) | 0.63 | 0.0405 |
| A_24_P285522 | [MAP4K3](http://genome-www4.stanford.edu/cgi-bin/SMD/source/sourceResult?choice=Gene&option=Name&criteria=MAP4K3) | 0.65 | 0.0405 |
| A_23_P67381 | [SULT2A1](http://genome-www4.stanford.edu/cgi-bin/SMD/source/sourceResult?choice=Gene&option=Name&criteria=SULT2A1) | 0.50 | 0.0447 |
| A_23_P314101 | [SUSD2](http://genome-www4.stanford.edu/cgi-bin/SMD/source/sourceResult?choice=Gene&option=Name&criteria=SUSD2) | 0.61 | 0.0447 |
| A_24_P224684 | [SULT2A1](http://genome-www4.stanford.edu/cgi-bin/SMD/source/sourceResult?choice=Gene&option=Name&criteria=SULT2A1) | 0.48 | 0.0447 |
| A_32_P491499 | [BC025775](http://genome-www4.stanford.edu/cgi-bin/SMD/source/sourceResult?choice=Gene&option=Name&criteria=BC025775) | 0.61 | 0.0447 |
| A_23_P120902 | [LGALS2](http://genome-www4.stanford.edu/cgi-bin/SMD/source/sourceResult?choice=Gene&option=Name&criteria=LGALS2) | 0.65 | 0.0447 |
| A_24_P740620 | [ENST00000382184](http://genome-www4.stanford.edu/cgi-bin/SMD/source/sourceResult?choice=Gene&option=Name&criteria=ENST00000382184) | 0.62 | 0.0447 |
| A_23_P130158 | [WNT3](http://genome-www4.stanford.edu/cgi-bin/SMD/source/sourceResult?choice=Gene&option=Name&criteria=WNT3) | 0.65 | 0.0447 |
| A_32_P206541 | [AK128714](http://genome-www4.stanford.edu/cgi-bin/SMD/source/sourceResult?choice=Gene&option=Name&criteria=AK128714) | 0.61 | 0.0447 |
| A_32_P34920 | [FOXD1](http://genome-www4.stanford.edu/cgi-bin/SMD/source/sourceResult?choice=Gene&option=Name&criteria=FOXD1) | 0.67 | 0.0447 |
| A_24_P272761 | [DENND1A](http://genome-www4.stanford.edu/cgi-bin/SMD/source/sourceResult?choice=Gene&option=Name&criteria=DENND1A) | 0.66 | 0.0447 |
| A_23_P413923 | [DMRTA1](http://genome-www4.stanford.edu/cgi-bin/SMD/source/sourceResult?choice=Gene&option=Name&criteria=DMRTA1) | 0.65 | 0.0447 |
| A_23_P390984 | [KCNH6](http://genome-www4.stanford.edu/cgi-bin/SMD/source/sourceResult?choice=Gene&option=Name&criteria=KCNH6) | 0.66 | 0.0447 |
| A_23_P25475 | [SOAT2](http://genome-www4.stanford.edu/cgi-bin/SMD/source/sourceResult?choice=Gene&option=Name&criteria=SOAT2) | 0.59 | 0.0447 |
| A_32_P166653 | [A_32_P166653](http://genome-www4.stanford.edu/cgi-bin/SMD/source/sourceResult?choice=Gene&option=Name&criteria=A_32_P166653) | 0.64 | 0.0447 |
| A_32_P47027 | [CD678339](http://genome-www4.stanford.edu/cgi-bin/SMD/source/sourceResult?choice=Gene&option=Name&criteria=CD678339) | 0.52 | 0.0447 |
| A_24_P341000 | [AK092698](http://genome-www4.stanford.edu/cgi-bin/SMD/source/sourceResult?choice=Gene&option=Name&criteria=AK092698) | 0.53 | 0.0447 |
| A_32_P40145 | [THC2345778](http://genome-www4.stanford.edu/cgi-bin/SMD/source/sourceResult?choice=Gene&option=Name&criteria=THC2345778) | 0.66 | 0.0447 |
| A_24_P111096 | [PFKFB3](http://genome-www4.stanford.edu/cgi-bin/SMD/source/sourceResult?choice=Gene&option=Name&criteria=PFKFB3) | 0.52 | 0.0447 |
| A_23_P110175 | [CTSO](http://genome-www4.stanford.edu/cgi-bin/SMD/source/sourceResult?choice=Gene&option=Name&criteria=CTSO) | 0.66 | 0.0447 |
| A_32_P101689 | [FAM3C](http://genome-www4.stanford.edu/cgi-bin/SMD/source/sourceResult?choice=Gene&option=Name&criteria=FAM3C) | 0.63 | 0.0447 |
| A_23_P251453 | [HNF4G](http://genome-www4.stanford.edu/cgi-bin/SMD/source/sourceResult?choice=Gene&option=Name&criteria=HNF4G) | 0.61 | 0.0447 |
| A_23_P44569 | [ABCC2](http://genome-www4.stanford.edu/cgi-bin/SMD/source/sourceResult?choice=Gene&option=Name&criteria=ABCC2) | 0.59 | 0.0477 |
| A_23_P56356 | [PLB1](http://genome-www4.stanford.edu/cgi-bin/SMD/source/sourceResult?choice=Gene&option=Name&criteria=PLB1) | 0.59 | 0.0477 |
| A_23_P209564 | [CYBRD1](http://genome-www4.stanford.edu/cgi-bin/SMD/source/sourceResult?choice=Gene&option=Name&criteria=CYBRD1) | 0.52 | 0.0477 |
| A_23_P76322 | [PIK3C2G](http://genome-www4.stanford.edu/cgi-bin/SMD/source/sourceResult?choice=Gene&option=Name&criteria=PIK3C2G) | 0.59 | 0.0477 |
| A_23_P52668 | [NAALADL1](http://genome-www4.stanford.edu/cgi-bin/SMD/source/sourceResult?choice=Gene&option=Name&criteria=NAALADL1) | 0.66 | 0.0477 |
| A_23_P158976 | [ABCC2](http://genome-www4.stanford.edu/cgi-bin/SMD/source/sourceResult?choice=Gene&option=Name&criteria=ABCC2) | 0.55 | 0.0477 |
| A_24_P59062 | [GGTL4](http://genome-www4.stanford.edu/cgi-bin/SMD/source/sourceResult?choice=Gene&option=Name&criteria=GGTL4) | 0.66 | 0.0477 |
| A_23_P389118 | [TMEM16F](http://genome-www4.stanford.edu/cgi-bin/SMD/source/sourceResult?choice=Gene&option=Name&criteria=TMEM16F) | 0.66 | 0.0477 |
| A_24_P85258 | [KIAA1751](http://genome-www4.stanford.edu/cgi-bin/SMD/source/sourceResult?choice=Gene&option=Name&criteria=KIAA1751) | 0.66 | 0.0477 |
| A_23_P37914 | [SLC5A11](http://genome-www4.stanford.edu/cgi-bin/SMD/source/sourceResult?choice=Gene&option=Name&criteria=SLC5A11) | 0.64 | 0.0477 |
| A_24_P294419 | [SEMA3B](http://genome-www4.stanford.edu/cgi-bin/SMD/source/sourceResult?choice=Gene&option=Name&criteria=SEMA3B) | 0.65 | 0.0477 |
|  |  |  |  |
| **Supplementary Table S1E. Gene-probes upregulated in CD compared to UC (n=946)** | | | |
| **Agilent ID** | **Gene Name or Accession Number** | **Fold Change** | **q-value** |
| A_23_P47616 | [FOLH1](http://genome-www4.stanford.edu/cgi-bin/SMD/source/sourceResult?choice=Gene&option=Name&criteria=FOLH1) | 8.67 | <0.0001 |
| A_32_P157391 | FOLH1B | 7.05 | <0.0001 |
| A_23_P20075 | [NPC1L1](http://genome-www4.stanford.edu/cgi-bin/SMD/source/sourceResult?choice=Gene&option=Name&criteria=NPC1L1) | 3.64 | <0.0001 |
| A_23_P75973 | [RNF121](http://genome-www4.stanford.edu/cgi-bin/SMD/source/sourceResult?choice=Gene&option=Name&criteria=RNF121) | 1.96 | <0.0001 |
| A_23_P395001 | [SLC2A12](http://genome-www4.stanford.edu/cgi-bin/SMD/source/sourceResult?choice=Gene&option=Name&criteria=SLC2A12) | 3.06 | <0.0001 |
| A_24_P698136 | [LOC344967](http://genome-www4.stanford.edu/cgi-bin/SMD/source/sourceResult?choice=Gene&option=Name&criteria=LOC344967) | 2.04 | <0.0001 |
| A_23_P208706 | [BAX](http://genome-www4.stanford.edu/cgi-bin/SMD/source/sourceResult?choice=Gene&option=Name&criteria=BAX) | 2.28 | <0.0001 |
| A_23_P346311 | [BAX](http://genome-www4.stanford.edu/cgi-bin/SMD/source/sourceResult?choice=Gene&option=Name&criteria=BAX) | 1.99 | <0.0001 |
| A_23_P35309 | [TAF5L](http://genome-www4.stanford.edu/cgi-bin/SMD/source/sourceResult?choice=Gene&option=Name&criteria=TAF5L) | 2.82 | <0.0001 |
| A_24_P389517 | [HNRPK](http://genome-www4.stanford.edu/cgi-bin/SMD/source/sourceResult?choice=Gene&option=Name&criteria=HNRPK) | 1.82 | <0.0001 |
| A_24_P714134 | [AK126814](http://genome-www4.stanford.edu/cgi-bin/SMD/source/sourceResult?choice=Gene&option=Name&criteria=AK126814) | 2.19 | <0.0001 |
| A_23_P156890 | [TCF21](http://genome-www4.stanford.edu/cgi-bin/SMD/source/sourceResult?choice=Gene&option=Name&criteria=TCF21) | 2.30 | <0.0001 |
| A_23_P120883 | [HMOX1](http://genome-www4.stanford.edu/cgi-bin/SMD/source/sourceResult?choice=Gene&option=Name&criteria=HMOX1) | 2.15 | <0.0001 |
| A_23_P79217 | [LCT](http://genome-www4.stanford.edu/cgi-bin/SMD/source/sourceResult?choice=Gene&option=Name&criteria=LCT) | 6.41 | <0.0001 |
| A_32_P8666 | [LOC644063](http://genome-www4.stanford.edu/cgi-bin/SMD/source/sourceResult?choice=Gene&option=Name&criteria=LOC644063) | 1.84 | <0.0001 |
| A_23_P29257 | [H1F0](http://genome-www4.stanford.edu/cgi-bin/SMD/source/sourceResult?choice=Gene&option=Name&criteria=H1F0) | 2.02 | <0.0001 |
| A_24_P673209 | [A_24_P673209](http://genome-www4.stanford.edu/cgi-bin/SMD/source/sourceResult?choice=Gene&option=Name&criteria=A_24_P673209) | 1.79 | <0.0001 |
| A_23_P353742 | [FLJ20294](http://genome-www4.stanford.edu/cgi-bin/SMD/source/sourceResult?choice=Gene&option=Name&criteria=FLJ20294) | 1.72 | <0.0001 |
| A_23_P15727 | [FKBP10](http://genome-www4.stanford.edu/cgi-bin/SMD/source/sourceResult?choice=Gene&option=Name&criteria=FKBP10) | 2.21 | <0.0001 |
| A_23_P9232 | [GCNT1](http://genome-www4.stanford.edu/cgi-bin/SMD/source/sourceResult?choice=Gene&option=Name&criteria=GCNT1) | 1.73 | <0.0001 |
| A_23_P52425 | [NKX2-3](http://genome-www4.stanford.edu/cgi-bin/SMD/source/sourceResult?choice=Gene&option=Name&criteria=NKX2-3) | 2.22 | <0.0001 |
| A_24_P538478 | [MED28](http://genome-www4.stanford.edu/cgi-bin/SMD/source/sourceResult?choice=Gene&option=Name&criteria=MED28) | 1.70 | <0.0001 |
| A_32_P178513 | [FOLH1](http://genome-www4.stanford.edu/cgi-bin/SMD/source/sourceResult?choice=Gene&option=Name&criteria=FOLH1) | 1.97 | <0.0001 |
| A_24_P332721 | [A_24_P332721](http://genome-www4.stanford.edu/cgi-bin/SMD/source/sourceResult?choice=Gene&option=Name&criteria=A_24_P332721) | 1.96 | <0.0001 |
| A_23_P253896 | [NPNT](http://genome-www4.stanford.edu/cgi-bin/SMD/source/sourceResult?choice=Gene&option=Name&criteria=NPNT) | 1.94 | <0.0001 |
| A_24_P183664 | [KIAA0644](http://genome-www4.stanford.edu/cgi-bin/SMD/source/sourceResult?choice=Gene&option=Name&criteria=KIAA0644) | 1.71 | <0.0001 |
| A_24_P925505 | [CD36](http://genome-www4.stanford.edu/cgi-bin/SMD/source/sourceResult?choice=Gene&option=Name&criteria=CD36) | 2.31 | <0.0001 |
| A_23_P39445 | [RKHD1](http://genome-www4.stanford.edu/cgi-bin/SMD/source/sourceResult?choice=Gene&option=Name&criteria=RKHD1) | 1.83 | <0.0001 |
| A_23_P157404 | [AP1S1](http://genome-www4.stanford.edu/cgi-bin/SMD/source/sourceResult?choice=Gene&option=Name&criteria=AP1S1) | 1.81 | <0.0001 |
| A_32_P133670 | [ANP32A](http://genome-www4.stanford.edu/cgi-bin/SMD/source/sourceResult?choice=Gene&option=Name&criteria=ANP32A) | 2.10 | <0.0001 |
| A_32_P125832 | [AK123079](http://genome-www4.stanford.edu/cgi-bin/SMD/source/sourceResult?choice=Gene&option=Name&criteria=AK123079) | 2.03 | <0.0001 |
| A_23_P20285 | [PDLIM2](http://genome-www4.stanford.edu/cgi-bin/SMD/source/sourceResult?choice=Gene&option=Name&criteria=PDLIM2) | 1.93 | <0.0001 |
| A_23_P28969 | [CHMP4B](http://genome-www4.stanford.edu/cgi-bin/SMD/source/sourceResult?choice=Gene&option=Name&criteria=CHMP4B) | 1.90 | <0.0001 |
| A_32_P137632 | [FBXL17](http://genome-www4.stanford.edu/cgi-bin/SMD/source/sourceResult?choice=Gene&option=Name&criteria=FBXL17) | 2.81 | <0.0001 |
| A_23_P362719 | [BC054888](http://genome-www4.stanford.edu/cgi-bin/SMD/source/sourceResult?choice=Gene&option=Name&criteria=BC054888) | 1.89 | <0.0001 |
| A_24_P296772 | [PPP1R14A](http://genome-www4.stanford.edu/cgi-bin/SMD/source/sourceResult?choice=Gene&option=Name&criteria=PPP1R14A) | 1.81 | <0.0001 |
| A_23_P151198 | [PDAP1](http://genome-www4.stanford.edu/cgi-bin/SMD/source/sourceResult?choice=Gene&option=Name&criteria=PDAP1) | 1.83 | <0.0001 |
| A_23_P72025 | [SLC25A20](http://genome-www4.stanford.edu/cgi-bin/SMD/source/sourceResult?choice=Gene&option=Name&criteria=SLC25A20) | 1.61 | <0.0001 |
| A_23_P343104 | [FLJ30901](http://genome-www4.stanford.edu/cgi-bin/SMD/source/sourceResult?choice=Gene&option=Name&criteria=FLJ30901) | 1.64 | <0.0001 |
| A_23_P331813 | [ZNF687](http://genome-www4.stanford.edu/cgi-bin/SMD/source/sourceResult?choice=Gene&option=Name&criteria=ZNF687) | 2.03 | <0.0001 |
| A_23_P107211 | [RAB5C](http://genome-www4.stanford.edu/cgi-bin/SMD/source/sourceResult?choice=Gene&option=Name&criteria=RAB5C) | 1.68 | <0.0001 |
| A_23_P499 | [B3GALT2](http://genome-www4.stanford.edu/cgi-bin/SMD/source/sourceResult?choice=Gene&option=Name&criteria=B3GALT2) | 1.65 | <0.0001 |
| A_24_P49267 | [A_24_P49267](http://genome-www4.stanford.edu/cgi-bin/SMD/source/sourceResult?choice=Gene&option=Name&criteria=A_24_P49267) | 1.64 | <0.0001 |
| A_23_P258982 | [PKN3](http://genome-www4.stanford.edu/cgi-bin/SMD/source/sourceResult?choice=Gene&option=Name&criteria=PKN3) | 1.58 | <0.0001 |
| A_32_P78295 | [ATOH7](http://genome-www4.stanford.edu/cgi-bin/SMD/source/sourceResult?choice=Gene&option=Name&criteria=ATOH7) | 2.12 | <0.0001 |
| A_24_P253827 | [AP2B1](http://genome-www4.stanford.edu/cgi-bin/SMD/source/sourceResult?choice=Gene&option=Name&criteria=AP2B1) | 2.03 | <0.0001 |
| A_23_P87580 | [ANP32D](http://genome-www4.stanford.edu/cgi-bin/SMD/source/sourceResult?choice=Gene&option=Name&criteria=ANP32D) | 1.89 | <0.0001 |
| A_23_P31844 | [ATP6V1B2](http://genome-www4.stanford.edu/cgi-bin/SMD/source/sourceResult?choice=Gene&option=Name&criteria=ATP6V1B2) | 1.76 | <0.0001 |
| A_23_P131202 | [HES6](http://genome-www4.stanford.edu/cgi-bin/SMD/source/sourceResult?choice=Gene&option=Name&criteria=HES6) | 1.78 | <0.0001 |
| A_23_P250629 | [PSMB8](http://genome-www4.stanford.edu/cgi-bin/SMD/source/sourceResult?choice=Gene&option=Name&criteria=PSMB8) | 1.52 | <0.0001 |
| A_23_P40240 | [CTSZ](http://genome-www4.stanford.edu/cgi-bin/SMD/source/sourceResult?choice=Gene&option=Name&criteria=CTSZ) | 2.47 | <0.0001 |
| A_32_P100830 | [A_32_P100830](http://genome-www4.stanford.edu/cgi-bin/SMD/source/sourceResult?choice=Gene&option=Name&criteria=A_32_P100830) | 1.63 | <0.0001 |
| A_24_P205589 | [ACOT7](http://genome-www4.stanford.edu/cgi-bin/SMD/source/sourceResult?choice=Gene&option=Name&criteria=ACOT7) | 1.80 | <0.0001 |
| A_24_P633686 | [A_24_P633686](http://genome-www4.stanford.edu/cgi-bin/SMD/source/sourceResult?choice=Gene&option=Name&criteria=A_24_P633686) | 1.56 | <0.0001 |
| A_23_P162739 | [TSC22D1](http://genome-www4.stanford.edu/cgi-bin/SMD/source/sourceResult?choice=Gene&option=Name&criteria=TSC22D1) | 1.95 | <0.0001 |
| A_23_P337849 | [TNRC4](http://genome-www4.stanford.edu/cgi-bin/SMD/source/sourceResult?choice=Gene&option=Name&criteria=TNRC4) | 1.92 | <0.0001 |
| A_24_P518369 | [A_24_P518369](http://genome-www4.stanford.edu/cgi-bin/SMD/source/sourceResult?choice=Gene&option=Name&criteria=A_24_P518369) | 2.13 | <0.0001 |
| A_23_P258234 | [ENST00000368503](http://genome-www4.stanford.edu/cgi-bin/SMD/source/sourceResult?choice=Gene&option=Name&criteria=ENST00000368503) | 1.63 | <0.0001 |
| A_24_P144314 | [A_24_P144314](http://genome-www4.stanford.edu/cgi-bin/SMD/source/sourceResult?choice=Gene&option=Name&criteria=A_24_P144314) | 1.88 | <0.0001 |
| A_23_P111583 | [CD36](http://genome-www4.stanford.edu/cgi-bin/SMD/source/sourceResult?choice=Gene&option=Name&criteria=CD36) | 2.01 | <0.0001 |
| A_23_P106194 | [FOS](http://genome-www4.stanford.edu/cgi-bin/SMD/source/sourceResult?choice=Gene&option=Name&criteria=FOS) | 2.21 | <0.0001 |
| A_24_P388322 | [COLEC11](http://genome-www4.stanford.edu/cgi-bin/SMD/source/sourceResult?choice=Gene&option=Name&criteria=COLEC11) | 1.93 | <0.0001 |
| A_23_P214658 | [PBX2](http://genome-www4.stanford.edu/cgi-bin/SMD/source/sourceResult?choice=Gene&option=Name&criteria=PBX2) | 2.01 | <0.0001 |
| A_23_P114423 | [RGN](http://genome-www4.stanford.edu/cgi-bin/SMD/source/sourceResult?choice=Gene&option=Name&criteria=RGN) | 2.50 | <0.0001 |
| A_23_P92520 | [ANP32C](http://genome-www4.stanford.edu/cgi-bin/SMD/source/sourceResult?choice=Gene&option=Name&criteria=ANP32C) | 1.76 | <0.0001 |
| A_23_P50399 | [LOC90379](http://genome-www4.stanford.edu/cgi-bin/SMD/source/sourceResult?choice=Gene&option=Name&criteria=LOC90379) | 1.59 | <0.0001 |
| A_23_P94689 | [C9orf74](http://genome-www4.stanford.edu/cgi-bin/SMD/source/sourceResult?choice=Gene&option=Name&criteria=C9orf74) | 1.58 | <0.0001 |
| A_23_P400406 | [ANKRD43](http://genome-www4.stanford.edu/cgi-bin/SMD/source/sourceResult?choice=Gene&option=Name&criteria=ANKRD43) | 1.51 | <0.0001 |
| A_24_P115932 | [GPR44](http://genome-www4.stanford.edu/cgi-bin/SMD/source/sourceResult?choice=Gene&option=Name&criteria=GPR44) | 1.80 | <0.0001 |
| A_24_P233850 | [SDHC](http://genome-www4.stanford.edu/cgi-bin/SMD/source/sourceResult?choice=Gene&option=Name&criteria=SDHC) | 1.74 | <0.0001 |
| A_24_P370472 | [HLA-DRB4](http://genome-www4.stanford.edu/cgi-bin/SMD/source/sourceResult?choice=Gene&option=Name&criteria=HLA-DRB4) | 1.80 | <0.0001 |
| A_24_P409816 | [A_24_P409816](http://genome-www4.stanford.edu/cgi-bin/SMD/source/sourceResult?choice=Gene&option=Name&criteria=A_24_P409816) | 1.68 | <0.0001 |
| A_23_P141508 | [CLEC10A](http://genome-www4.stanford.edu/cgi-bin/SMD/source/sourceResult?choice=Gene&option=Name&criteria=CLEC10A) | 1.57 | <0.0001 |
| A_23_P404698 | [FLJ35880](http://genome-www4.stanford.edu/cgi-bin/SMD/source/sourceResult?choice=Gene&option=Name&criteria=FLJ35880) | 2.16 | <0.0001 |
| A_24_P100228 | [XBP1](http://genome-www4.stanford.edu/cgi-bin/SMD/source/sourceResult?choice=Gene&option=Name&criteria=XBP1) | 1.87 | <0.0001 |
| A_23_P217114 | [ALAD](http://genome-www4.stanford.edu/cgi-bin/SMD/source/sourceResult?choice=Gene&option=Name&criteria=ALAD) | 1.52 | <0.0001 |
| A_23_P19523 | [MLN](http://genome-www4.stanford.edu/cgi-bin/SMD/source/sourceResult?choice=Gene&option=Name&criteria=MLN) | 2.38 | <0.0001 |
| A_32_P45009 | [IDH1](http://genome-www4.stanford.edu/cgi-bin/SMD/source/sourceResult?choice=Gene&option=Name&criteria=IDH1) | 1.57 | <0.0001 |
| A_24_P190424 | [RAB8A](http://genome-www4.stanford.edu/cgi-bin/SMD/source/sourceResult?choice=Gene&option=Name&criteria=RAB8A) | 2.01 | <0.0001 |
| A_24_P53778 | [ITLN2](http://genome-www4.stanford.edu/cgi-bin/SMD/source/sourceResult?choice=Gene&option=Name&criteria=ITLN2) | 2.76 | <0.0001 |
| A_23_P386356 | [KIF12](http://genome-www4.stanford.edu/cgi-bin/SMD/source/sourceResult?choice=Gene&option=Name&criteria=KIF12) | 2.14 | <0.0001 |
| A_23_P149019 | [BAI2](http://genome-www4.stanford.edu/cgi-bin/SMD/source/sourceResult?choice=Gene&option=Name&criteria=BAI2) | 1.80 | <0.0001 |
| A_24_P329597 | [UBQLN1](http://genome-www4.stanford.edu/cgi-bin/SMD/source/sourceResult?choice=Gene&option=Name&criteria=UBQLN1) | 1.94 | <0.0001 |
| A_24_P322191 | [CALCOCO2](http://genome-www4.stanford.edu/cgi-bin/SMD/source/sourceResult?choice=Gene&option=Name&criteria=CALCOCO2) | 1.51 | <0.0001 |
| A_24_P54485 | [CCDC115](http://genome-www4.stanford.edu/cgi-bin/SMD/source/sourceResult?choice=Gene&option=Name&criteria=CCDC115) | 1.83 | <0.0001 |
| A_23_P2789 | [OLFM4](http://genome-www4.stanford.edu/cgi-bin/SMD/source/sourceResult?choice=Gene&option=Name&criteria=OLFM4) | 2.40 | <0.0001 |
| A_24_P68649 | [RNPEP](http://genome-www4.stanford.edu/cgi-bin/SMD/source/sourceResult?choice=Gene&option=Name&criteria=RNPEP) | 1.89 | <0.0001 |
| A_23_P47102 | [ACY3](http://genome-www4.stanford.edu/cgi-bin/SMD/source/sourceResult?choice=Gene&option=Name&criteria=ACY3) | 2.22 | <0.0001 |
| A_24_P698141 | [LOC344967](http://genome-www4.stanford.edu/cgi-bin/SMD/source/sourceResult?choice=Gene&option=Name&criteria=LOC344967) | 1.69 | <0.0001 |
| A_24_P402779 | [PARP3](http://genome-www4.stanford.edu/cgi-bin/SMD/source/sourceResult?choice=Gene&option=Name&criteria=PARP3) | 1.71 | <0.0001 |
| A_24_P372901 | [MVK](http://genome-www4.stanford.edu/cgi-bin/SMD/source/sourceResult?choice=Gene&option=Name&criteria=MVK) | 1.54 | <0.0001 |
| A_24_P103952 | [DGKZ](http://genome-www4.stanford.edu/cgi-bin/SMD/source/sourceResult?choice=Gene&option=Name&criteria=DGKZ) | 1.78 | <0.0001 |
| A_23_P137157 | [RENBP](http://genome-www4.stanford.edu/cgi-bin/SMD/source/sourceResult?choice=Gene&option=Name&criteria=RENBP) | 1.59 | <0.0001 |
| A_32_P211188 | [LOC153346](http://genome-www4.stanford.edu/cgi-bin/SMD/source/sourceResult?choice=Gene&option=Name&criteria=LOC153346) | 1.70 | <0.0001 |
| A_23_P155755 | [CXCL6](http://genome-www4.stanford.edu/cgi-bin/SMD/source/sourceResult?choice=Gene&option=Name&criteria=CXCL6) | 2.43 | <0.0001 |
| A_24_P152793 | [A_24_P152793](http://genome-www4.stanford.edu/cgi-bin/SMD/source/sourceResult?choice=Gene&option=Name&criteria=A_24_P152793) | 1.53 | <0.0001 |
| A_24_P603890 | [A_24_P603890](http://genome-www4.stanford.edu/cgi-bin/SMD/source/sourceResult?choice=Gene&option=Name&criteria=A_24_P603890) | 1.61 | <0.0001 |
| A_23_P103968 | [AKR7A3](http://genome-www4.stanford.edu/cgi-bin/SMD/source/sourceResult?choice=Gene&option=Name&criteria=AKR7A3) | 1.74 | <0.0001 |
| A_23_P42353 | [ETV7](http://genome-www4.stanford.edu/cgi-bin/SMD/source/sourceResult?choice=Gene&option=Name&criteria=ETV7) | 1.91 | <0.0001 |
| A_23_P119763 | [ABCG5](http://genome-www4.stanford.edu/cgi-bin/SMD/source/sourceResult?choice=Gene&option=Name&criteria=ABCG5) | 3.00 | <0.0001 |
| A_23_P66854 | [KRT20](http://genome-www4.stanford.edu/cgi-bin/SMD/source/sourceResult?choice=Gene&option=Name&criteria=KRT20) | 2.01 | <0.0001 |
| A_23_P395438 | [HTRA3](http://genome-www4.stanford.edu/cgi-bin/SMD/source/sourceResult?choice=Gene&option=Name&criteria=HTRA3) | 1.81 | <0.0001 |
| A_23_P366828 | [ABCC6](http://genome-www4.stanford.edu/cgi-bin/SMD/source/sourceResult?choice=Gene&option=Name&criteria=ABCC6) | 1.60 | <0.0001 |
| A_24_P42264 | [LYZ](http://genome-www4.stanford.edu/cgi-bin/SMD/source/sourceResult?choice=Gene&option=Name&criteria=LYZ) | 2.11 | <0.0001 |
| A_23_P370707 | [A_23_P370707](http://genome-www4.stanford.edu/cgi-bin/SMD/source/sourceResult?choice=Gene&option=Name&criteria=A_23_P370707) | 1.56 | <0.0001 |
| A_23_P71268 | [AZGP1](http://genome-www4.stanford.edu/cgi-bin/SMD/source/sourceResult?choice=Gene&option=Name&criteria=AZGP1) | 1.54 | <0.0001 |
| A_23_P15889 | [CBLN2](http://genome-www4.stanford.edu/cgi-bin/SMD/source/sourceResult?choice=Gene&option=Name&criteria=CBLN2) | 1.76 | <0.0001 |
| A_23_P12620 | [TNKS2](http://genome-www4.stanford.edu/cgi-bin/SMD/source/sourceResult?choice=Gene&option=Name&criteria=TNKS2) | 1.60 | <0.0001 |
| A_24_P253003 | [WNT11](http://genome-www4.stanford.edu/cgi-bin/SMD/source/sourceResult?choice=Gene&option=Name&criteria=WNT11) | 2.07 | <0.0001 |
| A_23_P28878 | [C20orf27](http://genome-www4.stanford.edu/cgi-bin/SMD/source/sourceResult?choice=Gene&option=Name&criteria=C20orf27) | 1.69 | <0.0001 |
| A_23_P10121 | [SFRP1](http://genome-www4.stanford.edu/cgi-bin/SMD/source/sourceResult?choice=Gene&option=Name&criteria=SFRP1) | 1.81 | <0.0001 |
| A_32_P835626 | [FBXO34](http://genome-www4.stanford.edu/cgi-bin/SMD/source/sourceResult?choice=Gene&option=Name&criteria=FBXO34) | 1.65 | <0.0001 |
| A_23_P29621 | [GLYCTK](http://genome-www4.stanford.edu/cgi-bin/SMD/source/sourceResult?choice=Gene&option=Name&criteria=GLYCTK) | 1.57 | <0.0001 |
| A_24_P333479 | [SLC39A14](http://genome-www4.stanford.edu/cgi-bin/SMD/source/sourceResult?choice=Gene&option=Name&criteria=SLC39A14) | 1.89 | <0.0001 |
| A_23_P12730 | [CSTF2T](http://genome-www4.stanford.edu/cgi-bin/SMD/source/sourceResult?choice=Gene&option=Name&criteria=CSTF2T) | 2.32 | <0.0001 |
| A_24_P98047 | [SLC16A10](http://genome-www4.stanford.edu/cgi-bin/SMD/source/sourceResult?choice=Gene&option=Name&criteria=SLC16A10) | 1.95 | <0.0001 |
| A_24_P82135 | [KIAA0256](http://genome-www4.stanford.edu/cgi-bin/SMD/source/sourceResult?choice=Gene&option=Name&criteria=KIAA0256) | 1.69 | <0.0001 |
| A_23_P9875 | [TESK2](http://genome-www4.stanford.edu/cgi-bin/SMD/source/sourceResult?choice=Gene&option=Name&criteria=TESK2) | 1.57 | <0.0001 |
| A_23_P434212 | [SULT1A1](http://genome-www4.stanford.edu/cgi-bin/SMD/source/sourceResult?choice=Gene&option=Name&criteria=SULT1A1) | 1.87 | <0.0001 |
| A_23_P135787 | [BX537520](http://genome-www4.stanford.edu/cgi-bin/SMD/source/sourceResult?choice=Gene&option=Name&criteria=BX537520) | 1.63 | <0.0001 |
| A_23_P121533 | [SPON2](http://genome-www4.stanford.edu/cgi-bin/SMD/source/sourceResult?choice=Gene&option=Name&criteria=SPON2) | 1.67 | <0.0001 |
| A_23_P22350 | [GRAMD3](http://genome-www4.stanford.edu/cgi-bin/SMD/source/sourceResult?choice=Gene&option=Name&criteria=GRAMD3) | 1.66 | <0.0001 |
| A_23_P1759 | [AMICA1](http://genome-www4.stanford.edu/cgi-bin/SMD/source/sourceResult?choice=Gene&option=Name&criteria=AMICA1) | 1.61 | <0.0001 |
| A_23_P154938 | [HIRA](http://genome-www4.stanford.edu/cgi-bin/SMD/source/sourceResult?choice=Gene&option=Name&criteria=HIRA) | 1.96 | <0.0001 |
| A_24_P12413 | [TRAM2](http://genome-www4.stanford.edu/cgi-bin/SMD/source/sourceResult?choice=Gene&option=Name&criteria=TRAM2) | 1.61 | <0.0001 |
| A_23_P84475 | [ENST00000360178](http://genome-www4.stanford.edu/cgi-bin/SMD/source/sourceResult?choice=Gene&option=Name&criteria=ENST00000360178) | 1.52 | <0.0001 |
| A_23_P210100 | [CYP26B1](http://genome-www4.stanford.edu/cgi-bin/SMD/source/sourceResult?choice=Gene&option=Name&criteria=CYP26B1) | 1.82 | <0.0001 |
| A_24_P283000 | [DVL3](http://genome-www4.stanford.edu/cgi-bin/SMD/source/sourceResult?choice=Gene&option=Name&criteria=DVL3) | 1.56 | <0.0001 |
| A_23_P105794 | [EPSTI1](http://genome-www4.stanford.edu/cgi-bin/SMD/source/sourceResult?choice=Gene&option=Name&criteria=EPSTI1) | 1.69 | <0.0001 |
| A_23_P407142 | [LUZP1](http://genome-www4.stanford.edu/cgi-bin/SMD/source/sourceResult?choice=Gene&option=Name&criteria=LUZP1) | 1.74 | <0.0001 |
| A_23_P19529 | [MLN](http://genome-www4.stanford.edu/cgi-bin/SMD/source/sourceResult?choice=Gene&option=Name&criteria=MLN) | 2.18 | <0.0001 |
| A_32_P29408 | [BX337332](http://genome-www4.stanford.edu/cgi-bin/SMD/source/sourceResult?choice=Gene&option=Name&criteria=BX337332) | 1.83 | <0.0001 |
| A_23_P12767 | [CYP2C9](http://genome-www4.stanford.edu/cgi-bin/SMD/source/sourceResult?choice=Gene&option=Name&criteria=CYP2C9) | 2.13 | <0.0001 |
| A_24_P167806 | [IDH3A](http://genome-www4.stanford.edu/cgi-bin/SMD/source/sourceResult?choice=Gene&option=Name&criteria=IDH3A) | 1.57 | <0.0001 |
| A_23_P10127 | [SFRP1](http://genome-www4.stanford.edu/cgi-bin/SMD/source/sourceResult?choice=Gene&option=Name&criteria=SFRP1) | 1.89 | <0.0001 |
| A_24_P470754 | [A_24_P470754](http://genome-www4.stanford.edu/cgi-bin/SMD/source/sourceResult?choice=Gene&option=Name&criteria=A_24_P470754) | 1.59 | <0.0001 |
| A_23_P426809 | [ARHGEF11](http://genome-www4.stanford.edu/cgi-bin/SMD/source/sourceResult?choice=Gene&option=Name&criteria=ARHGEF11) | 1.60 | <0.0001 |
| A_24_P933675 | [ENST00000380749](http://genome-www4.stanford.edu/cgi-bin/SMD/source/sourceResult?choice=Gene&option=Name&criteria=ENST00000380749) | 1.92 | <0.0001 |
| A_23_P160940 | [ABCA4](http://genome-www4.stanford.edu/cgi-bin/SMD/source/sourceResult?choice=Gene&option=Name&criteria=ABCA4) | 2.19 | <0.0001 |
| A_23_P12514 | [RHOC](http://genome-www4.stanford.edu/cgi-bin/SMD/source/sourceResult?choice=Gene&option=Name&criteria=RHOC) | 1.59 | <0.0001 |
| A_23_P125107 | [HLA-B](http://genome-www4.stanford.edu/cgi-bin/SMD/source/sourceResult?choice=Gene&option=Name&criteria=HLA-B) | 1.64 | <0.0001 |
| A_24_P89887 | [C9orf3](http://genome-www4.stanford.edu/cgi-bin/SMD/source/sourceResult?choice=Gene&option=Name&criteria=C9orf3) | 1.51 | <0.0001 |
| A_24_P188218 | [MYL4](http://genome-www4.stanford.edu/cgi-bin/SMD/source/sourceResult?choice=Gene&option=Name&criteria=MYL4) | 1.73 | <0.0001 |
| A_32_P234459 | [HLA-H](http://genome-www4.stanford.edu/cgi-bin/SMD/source/sourceResult?choice=Gene&option=Name&criteria=HLA-H) | 2.07 | <0.0001 |
| A_32_P69536 | [THC2381210](http://genome-www4.stanford.edu/cgi-bin/SMD/source/sourceResult?choice=Gene&option=Name&criteria=THC2381210) | 1.65 | <0.0001 |
| A_23_P214408 | [UNC93A](http://genome-www4.stanford.edu/cgi-bin/SMD/source/sourceResult?choice=Gene&option=Name&criteria=UNC93A) | 2.40 | <0.0001 |
| A_24_P212539 | [GALM](http://genome-www4.stanford.edu/cgi-bin/SMD/source/sourceResult?choice=Gene&option=Name&criteria=GALM) | 1.52 | <0.0001 |
| A_23_P15692 | [GPR172B](http://genome-www4.stanford.edu/cgi-bin/SMD/source/sourceResult?choice=Gene&option=Name&criteria=GPR172B) | 1.87 | <0.0001 |
| A_23_P12950 | [KBTBD4](http://genome-www4.stanford.edu/cgi-bin/SMD/source/sourceResult?choice=Gene&option=Name&criteria=KBTBD4) | 1.73 | <0.0001 |
| A_24_P262201 | [SULT1A4](http://genome-www4.stanford.edu/cgi-bin/SMD/source/sourceResult?choice=Gene&option=Name&criteria=SULT1A4) | 1.55 | 0.0006 |
| A_24_P376483 | [HLA-A](http://genome-www4.stanford.edu/cgi-bin/SMD/source/sourceResult?choice=Gene&option=Name&criteria=HLA-A) | 2.06 | 0.0006 |
| A_23_P314024 | [HLA-F](http://genome-www4.stanford.edu/cgi-bin/SMD/source/sourceResult?choice=Gene&option=Name&criteria=HLA-F) | 1.54 | 0.0006 |
| A_23_P66454 | [GSDML](http://genome-www4.stanford.edu/cgi-bin/SMD/source/sourceResult?choice=Gene&option=Name&criteria=GSDML) | 1.53 | 0.0006 |
| A_23_P112548 | [HDHD3](http://genome-www4.stanford.edu/cgi-bin/SMD/source/sourceResult?choice=Gene&option=Name&criteria=HDHD3) | 1.88 | 0.0006 |
| A_23_P101297 | [EML2](http://genome-www4.stanford.edu/cgi-bin/SMD/source/sourceResult?choice=Gene&option=Name&criteria=EML2) | 1.61 | 0.0006 |
| A_23_P70539 | [HLA-C](http://genome-www4.stanford.edu/cgi-bin/SMD/source/sourceResult?choice=Gene&option=Name&criteria=HLA-C) | 2.06 | 0.0006 |
| A_23_P146981 | [A_23_P146981](http://genome-www4.stanford.edu/cgi-bin/SMD/source/sourceResult?choice=Gene&option=Name&criteria=A_23_P146981) | 1.84 | 0.0006 |
| A_24_P103434 | [UNC93A](http://genome-www4.stanford.edu/cgi-bin/SMD/source/sourceResult?choice=Gene&option=Name&criteria=UNC93A) | 1.65 | 0.0006 |
| A_24_P156922 | [SCP2](http://genome-www4.stanford.edu/cgi-bin/SMD/source/sourceResult?choice=Gene&option=Name&criteria=SCP2) | 2.16 | 0.0006 |
| A_23_P159211 | [ENST00000360178](http://genome-www4.stanford.edu/cgi-bin/SMD/source/sourceResult?choice=Gene&option=Name&criteria=ENST00000360178) | 1.51 | 0.0006 |
| A_24_P221974 | [A_24_P221974](http://genome-www4.stanford.edu/cgi-bin/SMD/source/sourceResult?choice=Gene&option=Name&criteria=A_24_P221974) | 1.51 | 0.0006 |
| A_23_P160800 | [NR0B2](http://genome-www4.stanford.edu/cgi-bin/SMD/source/sourceResult?choice=Gene&option=Name&criteria=NR0B2) | 2.00 | 0.0006 |
| A_23_P502654 | [SHMT1](http://genome-www4.stanford.edu/cgi-bin/SMD/source/sourceResult?choice=Gene&option=Name&criteria=SHMT1) | 1.59 | 0.0006 |
| A_24_P273679 | [YAP1](http://genome-www4.stanford.edu/cgi-bin/SMD/source/sourceResult?choice=Gene&option=Name&criteria=YAP1) | 2.05 | 0.0006 |
| A_23_P124300 | [BCMO1](http://genome-www4.stanford.edu/cgi-bin/SMD/source/sourceResult?choice=Gene&option=Name&criteria=BCMO1) | 1.76 | 0.0006 |
| A_24_P182182 | [SLC25A5](http://genome-www4.stanford.edu/cgi-bin/SMD/source/sourceResult?choice=Gene&option=Name&criteria=SLC25A5) | 1.52 | 0.0006 |
| A_24_P679796 | [A_24_P679796](http://genome-www4.stanford.edu/cgi-bin/SMD/source/sourceResult?choice=Gene&option=Name&criteria=A_24_P679796) | 1.53 | 0.0006 |
| A_24_P392925 | [LOC388323](http://genome-www4.stanford.edu/cgi-bin/SMD/source/sourceResult?choice=Gene&option=Name&criteria=LOC388323) | 1.74 | 0.0006 |
| A_24_P314159 | [APP](http://genome-www4.stanford.edu/cgi-bin/SMD/source/sourceResult?choice=Gene&option=Name&criteria=APP) | 1.81 | 0.0006 |
| A_23_P86599 | [DMBT1](http://genome-www4.stanford.edu/cgi-bin/SMD/source/sourceResult?choice=Gene&option=Name&criteria=DMBT1) | 1.94 | 0.0006 |
| A_24_P263767 | [ENST00000376793](http://genome-www4.stanford.edu/cgi-bin/SMD/source/sourceResult?choice=Gene&option=Name&criteria=ENST00000376793) | 2.01 | 0.0006 |
| A_32_P102252 | [CR591764](http://genome-www4.stanford.edu/cgi-bin/SMD/source/sourceResult?choice=Gene&option=Name&criteria=CR591764) | 1.54 | 0.0006 |
| A_24_P326082 | [HLA-E](http://genome-www4.stanford.edu/cgi-bin/SMD/source/sourceResult?choice=Gene&option=Name&criteria=HLA-E) | 1.55 | 0.0006 |
| A_23_P51690 | [RHBG](http://genome-www4.stanford.edu/cgi-bin/SMD/source/sourceResult?choice=Gene&option=Name&criteria=RHBG) | 1.59 | 0.0006 |
| A_23_P19936 | [KDELR2](http://genome-www4.stanford.edu/cgi-bin/SMD/source/sourceResult?choice=Gene&option=Name&criteria=KDELR2) | 1.65 | 0.0006 |
| A_24_P148521 | [TMBIM1](http://genome-www4.stanford.edu/cgi-bin/SMD/source/sourceResult?choice=Gene&option=Name&criteria=TMBIM1) | 1.63 | 0.0006 |
| A_23_P36700 | [TAPBPL](http://genome-www4.stanford.edu/cgi-bin/SMD/source/sourceResult?choice=Gene&option=Name&criteria=TAPBPL) | 1.52 | 0.0006 |
| A_23_P206474 | [TRAF7](http://genome-www4.stanford.edu/cgi-bin/SMD/source/sourceResult?choice=Gene&option=Name&criteria=TRAF7) | 1.61 | 0.0006 |
| A_23_P117424 | [WDR23](http://genome-www4.stanford.edu/cgi-bin/SMD/source/sourceResult?choice=Gene&option=Name&criteria=WDR23) | 1.61 | 0.0006 |
| A_23_P406424 | [RHOC](http://genome-www4.stanford.edu/cgi-bin/SMD/source/sourceResult?choice=Gene&option=Name&criteria=RHOC) | 1.58 | 0.0006 |
| A_23_P90014 | [AX775899](http://genome-www4.stanford.edu/cgi-bin/SMD/source/sourceResult?choice=Gene&option=Name&criteria=AX775899) | 1.53 | 0.0006 |
| A_23_P359131 | [NIPA1](http://genome-www4.stanford.edu/cgi-bin/SMD/source/sourceResult?choice=Gene&option=Name&criteria=NIPA1) | 1.55 | 0.0006 |
| A_23_P45361 | [GLUD2](http://genome-www4.stanford.edu/cgi-bin/SMD/source/sourceResult?choice=Gene&option=Name&criteria=GLUD2) | 1.66 | 0.0006 |
| A_23_P20494 | [NDRG1](http://genome-www4.stanford.edu/cgi-bin/SMD/source/sourceResult?choice=Gene&option=Name&criteria=NDRG1) | 1.53 | 0.0006 |
| A_23_P373126 | [BC035647](http://genome-www4.stanford.edu/cgi-bin/SMD/source/sourceResult?choice=Gene&option=Name&criteria=BC035647) | 1.58 | 0.0006 |
| A_24_P80532 | [CCNG2](http://genome-www4.stanford.edu/cgi-bin/SMD/source/sourceResult?choice=Gene&option=Name&criteria=CCNG2) | 1.93 | 0.0006 |
| A_24_P32646 | [A_24_P32646](http://genome-www4.stanford.edu/cgi-bin/SMD/source/sourceResult?choice=Gene&option=Name&criteria=A_24_P32646) | 1.71 | 0.0006 |
| A_23_P100539 | [CR609146](http://genome-www4.stanford.edu/cgi-bin/SMD/source/sourceResult?choice=Gene&option=Name&criteria=CR609146) | 1.64 | 0.0006 |
| A_23_P391980 | [CMTM4](http://genome-www4.stanford.edu/cgi-bin/SMD/source/sourceResult?choice=Gene&option=Name&criteria=CMTM4) | 1.54 | 0.0006 |
| A_23_P55828 | [CCL25](http://genome-www4.stanford.edu/cgi-bin/SMD/source/sourceResult?choice=Gene&option=Name&criteria=CCL25) | 2.33 | 0.0006 |
| A_23_P163787 | [MMP2](http://genome-www4.stanford.edu/cgi-bin/SMD/source/sourceResult?choice=Gene&option=Name&criteria=MMP2) | 1.73 | 0.0006 |
| A_23_P85201 | [PLP1](http://genome-www4.stanford.edu/cgi-bin/SMD/source/sourceResult?choice=Gene&option=Name&criteria=PLP1) | 1.77 | 0.0006 |
| A_23_P94795 | [TEAD4](http://genome-www4.stanford.edu/cgi-bin/SMD/source/sourceResult?choice=Gene&option=Name&criteria=TEAD4) | 1.52 | 0.0006 |
| A_32_P170481 | [ENST00000377548](http://genome-www4.stanford.edu/cgi-bin/SMD/source/sourceResult?choice=Gene&option=Name&criteria=ENST00000377548) | 1.80 | 0.0006 |
| A_23_P77328 | [GCHFR](http://genome-www4.stanford.edu/cgi-bin/SMD/source/sourceResult?choice=Gene&option=Name&criteria=GCHFR) | 1.64 | 0.0006 |
| A_32_P117338 | [AFG3L2](http://genome-www4.stanford.edu/cgi-bin/SMD/source/sourceResult?choice=Gene&option=Name&criteria=AFG3L2) | 1.86 | 0.0006 |
| A_32_P155247 | [FTL](http://genome-www4.stanford.edu/cgi-bin/SMD/source/sourceResult?choice=Gene&option=Name&criteria=FTL) | 1.60 | 0.0006 |
| A_23_P134347 | [CPVL](http://genome-www4.stanford.edu/cgi-bin/SMD/source/sourceResult?choice=Gene&option=Name&criteria=CPVL) | 1.82 | 0.0006 |
| A_23_P57868 | [ACY1](http://genome-www4.stanford.edu/cgi-bin/SMD/source/sourceResult?choice=Gene&option=Name&criteria=ACY1) | 1.60 | 0.0006 |
| A_23_P209251 | [THC2316768](http://genome-www4.stanford.edu/cgi-bin/SMD/source/sourceResult?choice=Gene&option=Name&criteria=THC2316768) | 2.07 | 0.0006 |
| A_23_P143218 | [ACOT8](http://genome-www4.stanford.edu/cgi-bin/SMD/source/sourceResult?choice=Gene&option=Name&criteria=ACOT8) | 1.63 | 0.0006 |
| A_24_P246591 | [ENST00000305820](http://genome-www4.stanford.edu/cgi-bin/SMD/source/sourceResult?choice=Gene&option=Name&criteria=ENST00000305820) | 1.72 | 0.0006 |
| A_24_P151692 | [POF1B](http://genome-www4.stanford.edu/cgi-bin/SMD/source/sourceResult?choice=Gene&option=Name&criteria=POF1B) | 1.67 | 0.0006 |
| A_24_P11315 | [OLFML3](http://genome-www4.stanford.edu/cgi-bin/SMD/source/sourceResult?choice=Gene&option=Name&criteria=OLFML3) | 1.58 | 0.0006 |
| A_24_P161933 | [CR608347](http://genome-www4.stanford.edu/cgi-bin/SMD/source/sourceResult?choice=Gene&option=Name&criteria=CR608347) | 1.88 | 0.0006 |
| A_23_P70127 | [TMED9](http://genome-www4.stanford.edu/cgi-bin/SMD/source/sourceResult?choice=Gene&option=Name&criteria=TMED9) | 1.79 | 0.0006 |
| A_23_P162982 | [DHRS4](http://genome-www4.stanford.edu/cgi-bin/SMD/source/sourceResult?choice=Gene&option=Name&criteria=DHRS4) | 1.52 | 0.0006 |
| A_23_P145264 | [HLA-F](http://genome-www4.stanford.edu/cgi-bin/SMD/source/sourceResult?choice=Gene&option=Name&criteria=HLA-F) | 1.55 | 0.0006 |
| A_23_P112481 | [AQP3](http://genome-www4.stanford.edu/cgi-bin/SMD/source/sourceResult?choice=Gene&option=Name&criteria=AQP3) | 1.80 | 0.0006 |
| A_32_P107746 | [ENSA](http://genome-www4.stanford.edu/cgi-bin/SMD/source/sourceResult?choice=Gene&option=Name&criteria=ENSA) | 1.51 | 0.0006 |
| A_32_P24832 | [OLFML3](http://genome-www4.stanford.edu/cgi-bin/SMD/source/sourceResult?choice=Gene&option=Name&criteria=OLFML3) | 1.53 | 0.0006 |
| A_23_P5610 | [DOK1](http://genome-www4.stanford.edu/cgi-bin/SMD/source/sourceResult?choice=Gene&option=Name&criteria=DOK1) | 1.51 | 0.0006 |
| A_23_P161171 | [ASAH2](http://genome-www4.stanford.edu/cgi-bin/SMD/source/sourceResult?choice=Gene&option=Name&criteria=ASAH2) | 1.78 | 0.0010 |
| A_24_P61864 | [CCDC47](http://genome-www4.stanford.edu/cgi-bin/SMD/source/sourceResult?choice=Gene&option=Name&criteria=CCDC47) | 1.67 | 0.0010 |
| A_23_P99249 | [SUCLG2](http://genome-www4.stanford.edu/cgi-bin/SMD/source/sourceResult?choice=Gene&option=Name&criteria=SUCLG2) | 1.58 | 0.0010 |
| A_23_P43988 | [DPYD](http://genome-www4.stanford.edu/cgi-bin/SMD/source/sourceResult?choice=Gene&option=Name&criteria=DPYD) | 1.89 | 0.0010 |
| A_24_P508946 | [LOC648674](http://genome-www4.stanford.edu/cgi-bin/SMD/source/sourceResult?choice=Gene&option=Name&criteria=LOC648674) | 2.11 | 0.0010 |
| A_23_P27005 | [MGC4172](http://genome-www4.stanford.edu/cgi-bin/SMD/source/sourceResult?choice=Gene&option=Name&criteria=MGC4172) | 1.72 | 0.0010 |
| A_23_P14853 | [LTK](http://genome-www4.stanford.edu/cgi-bin/SMD/source/sourceResult?choice=Gene&option=Name&criteria=LTK) | 1.68 | 0.0010 |
| A_23_P311468 | [ZNF428](http://genome-www4.stanford.edu/cgi-bin/SMD/source/sourceResult?choice=Gene&option=Name&criteria=ZNF428) | 1.61 | 0.0010 |
| A_23_P26771 | [CD300C](http://genome-www4.stanford.edu/cgi-bin/SMD/source/sourceResult?choice=Gene&option=Name&criteria=CD300C) | 1.52 | 0.0010 |
| A_23_P144179 | [CPOX](http://genome-www4.stanford.edu/cgi-bin/SMD/source/sourceResult?choice=Gene&option=Name&criteria=CPOX) | 1.63 | 0.0010 |
| A_24_P762613 | [AK021543](http://genome-www4.stanford.edu/cgi-bin/SMD/source/sourceResult?choice=Gene&option=Name&criteria=AK021543) | 1.98 | 0.0010 |
| A_23_P72068 | [GMDS](http://genome-www4.stanford.edu/cgi-bin/SMD/source/sourceResult?choice=Gene&option=Name&criteria=GMDS) | 1.71 | 0.0010 |
| A_24_P123190 | [PLD1](http://genome-www4.stanford.edu/cgi-bin/SMD/source/sourceResult?choice=Gene&option=Name&criteria=PLD1) | 1.75 | 0.0010 |
| A_23_P31006 | [HLA-DRB5](http://genome-www4.stanford.edu/cgi-bin/SMD/source/sourceResult?choice=Gene&option=Name&criteria=HLA-DRB5) | 1.67 | 0.0010 |
| A_23_P37702 | [TPSB2](http://genome-www4.stanford.edu/cgi-bin/SMD/source/sourceResult?choice=Gene&option=Name&criteria=TPSB2) | 1.80 | 0.0010 |
| A_23_P31041 | [MYLIP](http://genome-www4.stanford.edu/cgi-bin/SMD/source/sourceResult?choice=Gene&option=Name&criteria=MYLIP) | 1.65 | 0.0010 |
| A_23_P380754 | [PRSS1](http://genome-www4.stanford.edu/cgi-bin/SMD/source/sourceResult?choice=Gene&option=Name&criteria=PRSS1) | 1.59 | 0.0010 |
| A_23_P20713 | [C8G](http://genome-www4.stanford.edu/cgi-bin/SMD/source/sourceResult?choice=Gene&option=Name&criteria=C8G) | 1.91 | 0.0010 |
| A_23_P7727 | [HAPLN1](http://genome-www4.stanford.edu/cgi-bin/SMD/source/sourceResult?choice=Gene&option=Name&criteria=HAPLN1) | 1.90 | 0.0010 |
| A_23_P21316 | [PRUNE](http://genome-www4.stanford.edu/cgi-bin/SMD/source/sourceResult?choice=Gene&option=Name&criteria=PRUNE) | 1.61 | 0.0010 |
| A_23_P133916 | [C2](http://genome-www4.stanford.edu/cgi-bin/SMD/source/sourceResult?choice=Gene&option=Name&criteria=C2) | 1.69 | 0.0010 |
| A_24_P846755 | [A_24_P846755](http://genome-www4.stanford.edu/cgi-bin/SMD/source/sourceResult?choice=Gene&option=Name&criteria=A_24_P846755) | 1.91 | 0.0010 |
| A_24_P363134 | [TRIM36](http://genome-www4.stanford.edu/cgi-bin/SMD/source/sourceResult?choice=Gene&option=Name&criteria=TRIM36) | 1.66 | 0.0010 |
| A_23_P80491 | [RBP2](http://genome-www4.stanford.edu/cgi-bin/SMD/source/sourceResult?choice=Gene&option=Name&criteria=RBP2) | 2.67 | 0.0010 |
| A_23_P147641 | [TCEA2](http://genome-www4.stanford.edu/cgi-bin/SMD/source/sourceResult?choice=Gene&option=Name&criteria=TCEA2) | 1.69 | 0.0010 |
| A_23_P30848 | [HLA-E](http://genome-www4.stanford.edu/cgi-bin/SMD/source/sourceResult?choice=Gene&option=Name&criteria=HLA-E) | 1.94 | 0.0010 |
| A_24_P33444 | [YWHAE](http://genome-www4.stanford.edu/cgi-bin/SMD/source/sourceResult?choice=Gene&option=Name&criteria=YWHAE) | 1.71 | 0.0010 |
| A_23_P158481 | [CYP2C19](http://genome-www4.stanford.edu/cgi-bin/SMD/source/sourceResult?choice=Gene&option=Name&criteria=CYP2C19) | 2.32 | 0.0010 |
| A_24_P174550 | [RHOA](http://genome-www4.stanford.edu/cgi-bin/SMD/source/sourceResult?choice=Gene&option=Name&criteria=RHOA) | 1.61 | 0.0010 |
| A_23_P78342 | [LMAN1](http://genome-www4.stanford.edu/cgi-bin/SMD/source/sourceResult?choice=Gene&option=Name&criteria=LMAN1) | 1.53 | 0.0010 |
| A_23_P27040 | [TMEM98](http://genome-www4.stanford.edu/cgi-bin/SMD/source/sourceResult?choice=Gene&option=Name&criteria=TMEM98) | 1.52 | 0.0010 |
| A_23_P43415 | [HSD17B3](http://genome-www4.stanford.edu/cgi-bin/SMD/source/sourceResult?choice=Gene&option=Name&criteria=HSD17B3) | 1.75 | 0.0010 |
| A_23_P5778 | [RAB17](http://genome-www4.stanford.edu/cgi-bin/SMD/source/sourceResult?choice=Gene&option=Name&criteria=RAB17) | 1.95 | 0.0010 |
| A_24_P639679 | [AK095831](http://genome-www4.stanford.edu/cgi-bin/SMD/source/sourceResult?choice=Gene&option=Name&criteria=AK095831) | 1.68 | 0.0010 |
| A_23_P218247 | [FLJ21736](http://genome-www4.stanford.edu/cgi-bin/SMD/source/sourceResult?choice=Gene&option=Name&criteria=FLJ21736) | 1.55 | 0.0010 |
| A_23_P154801 | [TRPC4AP](http://genome-www4.stanford.edu/cgi-bin/SMD/source/sourceResult?choice=Gene&option=Name&criteria=TRPC4AP) | 1.57 | 0.0010 |
| A_23_P252808 | [WBP1](http://genome-www4.stanford.edu/cgi-bin/SMD/source/sourceResult?choice=Gene&option=Name&criteria=WBP1) | 1.52 | 0.0010 |
| A_23_P61042 | [ENST00000322032](http://genome-www4.stanford.edu/cgi-bin/SMD/source/sourceResult?choice=Gene&option=Name&criteria=ENST00000322032) | 1.69 | 0.0010 |
| A_23_P51217 | [CLCA1](http://genome-www4.stanford.edu/cgi-bin/SMD/source/sourceResult?choice=Gene&option=Name&criteria=CLCA1) | 1.57 | 0.0010 |
| A_23_P402952 | [LOC441294](http://genome-www4.stanford.edu/cgi-bin/SMD/source/sourceResult?choice=Gene&option=Name&criteria=LOC441294) | 1.65 | 0.0010 |
| A_32_P34522 | [BC010544](http://genome-www4.stanford.edu/cgi-bin/SMD/source/sourceResult?choice=Gene&option=Name&criteria=BC010544) | 1.55 | 0.0010 |
| A_32_P221966 | [AY102069](http://genome-www4.stanford.edu/cgi-bin/SMD/source/sourceResult?choice=Gene&option=Name&criteria=AY102069) | 1.76 | 0.0010 |
| A_23_P258493 | [LMNB1](http://genome-www4.stanford.edu/cgi-bin/SMD/source/sourceResult?choice=Gene&option=Name&criteria=LMNB1) | 1.81 | 0.0010 |
| A_23_P152047 | [SCAMP5](http://genome-www4.stanford.edu/cgi-bin/SMD/source/sourceResult?choice=Gene&option=Name&criteria=SCAMP5) | 1.73 | 0.0010 |
| A_24_P276628 | [PPT1](http://genome-www4.stanford.edu/cgi-bin/SMD/source/sourceResult?choice=Gene&option=Name&criteria=PPT1) | 1.53 | 0.0010 |
| A_23_P86421 | [NCOA4](http://genome-www4.stanford.edu/cgi-bin/SMD/source/sourceResult?choice=Gene&option=Name&criteria=NCOA4) | 1.70 | 0.0010 |
| A_23_P43276 | [GPR124](http://genome-www4.stanford.edu/cgi-bin/SMD/source/sourceResult?choice=Gene&option=Name&criteria=GPR124) | 1.60 | 0.0010 |
| A_23_P28090 | [STX10](http://genome-www4.stanford.edu/cgi-bin/SMD/source/sourceResult?choice=Gene&option=Name&criteria=STX10) | 1.54 | 0.0010 |
| A_24_P89457 | [CDKN1A](http://genome-www4.stanford.edu/cgi-bin/SMD/source/sourceResult?choice=Gene&option=Name&criteria=CDKN1A) | 1.67 | 0.0010 |
| A_23_P29851 | [LRPAP1](http://genome-www4.stanford.edu/cgi-bin/SMD/source/sourceResult?choice=Gene&option=Name&criteria=LRPAP1) | 1.55 | 0.0010 |
| A_23_P83098 | [ALDH1A1](http://genome-www4.stanford.edu/cgi-bin/SMD/source/sourceResult?choice=Gene&option=Name&criteria=ALDH1A1) | 1.63 | 0.0010 |
| A_24_P135748 | [GRTP1](http://genome-www4.stanford.edu/cgi-bin/SMD/source/sourceResult?choice=Gene&option=Name&criteria=GRTP1) | 1.54 | 0.0010 |
| A_24_P101771 | [A_24_P101771](http://genome-www4.stanford.edu/cgi-bin/SMD/source/sourceResult?choice=Gene&option=Name&criteria=A_24_P101771) | 1.98 | 0.0010 |
| A_24_P295745 | [HSPA8](http://genome-www4.stanford.edu/cgi-bin/SMD/source/sourceResult?choice=Gene&option=Name&criteria=HSPA8) | 1.54 | 0.0010 |
| A_24_P164731 | [TMED1](http://genome-www4.stanford.edu/cgi-bin/SMD/source/sourceResult?choice=Gene&option=Name&criteria=TMED1) | 1.56 | 0.0010 |
| A_23_P150018 | [DUSP5](http://genome-www4.stanford.edu/cgi-bin/SMD/source/sourceResult?choice=Gene&option=Name&criteria=DUSP5) | 1.56 | 0.0010 |
| A_24_P398585 | [UNG](http://genome-www4.stanford.edu/cgi-bin/SMD/source/sourceResult?choice=Gene&option=Name&criteria=UNG) | 1.56 | 0.0010 |
| A_23_P104199 | [ITGB1](http://genome-www4.stanford.edu/cgi-bin/SMD/source/sourceResult?choice=Gene&option=Name&criteria=ITGB1) | 1.53 | 0.0010 |
| A_23_P155351 | [BTD](http://genome-www4.stanford.edu/cgi-bin/SMD/source/sourceResult?choice=Gene&option=Name&criteria=BTD) | 1.87 | 0.0010 |
| A_24_P401381 | [A_24_P401381](http://genome-www4.stanford.edu/cgi-bin/SMD/source/sourceResult?choice=Gene&option=Name&criteria=A_24_P401381) | 1.50 | 0.0010 |
| A_23_P110175 | [CTSO](http://genome-www4.stanford.edu/cgi-bin/SMD/source/sourceResult?choice=Gene&option=Name&criteria=CTSO) | 2.03 | 0.0010 |
| A_24_P106297 | [AMACR](http://genome-www4.stanford.edu/cgi-bin/SMD/source/sourceResult?choice=Gene&option=Name&criteria=AMACR) | 1.52 | 0.0010 |
| A_23_P159305 | [TAF15](http://genome-www4.stanford.edu/cgi-bin/SMD/source/sourceResult?choice=Gene&option=Name&criteria=TAF15) | 1.61 | 0.0010 |
| A_23_P119562 | [CFD](http://genome-www4.stanford.edu/cgi-bin/SMD/source/sourceResult?choice=Gene&option=Name&criteria=CFD) | 1.73 | 0.0010 |
| A_24_P379858 | [ACAA1](http://genome-www4.stanford.edu/cgi-bin/SMD/source/sourceResult?choice=Gene&option=Name&criteria=ACAA1) | 1.72 | 0.0010 |
| A_24_P315873 | [ENST00000354937](http://genome-www4.stanford.edu/cgi-bin/SMD/source/sourceResult?choice=Gene&option=Name&criteria=ENST00000354937) | 1.62 | 0.0010 |
| A_24_P305223 | [CTAGE1](http://genome-www4.stanford.edu/cgi-bin/SMD/source/sourceResult?choice=Gene&option=Name&criteria=CTAGE1) | 1.82 | 0.0010 |
| A_32_P85978 | [ZNF414](http://genome-www4.stanford.edu/cgi-bin/SMD/source/sourceResult?choice=Gene&option=Name&criteria=ZNF414) | 1.51 | 0.0010 |
| A_24_P298495 | [AF289590](http://genome-www4.stanford.edu/cgi-bin/SMD/source/sourceResult?choice=Gene&option=Name&criteria=AF289590) | 1.71 | 0.0010 |
| A_23_P76322 | [PIK3C2G](http://genome-www4.stanford.edu/cgi-bin/SMD/source/sourceResult?choice=Gene&option=Name&criteria=PIK3C2G) | 2.32 | 0.0010 |
| A_24_P79755 | [AKR1A1](http://genome-www4.stanford.edu/cgi-bin/SMD/source/sourceResult?choice=Gene&option=Name&criteria=AKR1A1) | 1.56 | 0.0010 |
| A_23_P158593 | [COL5A1](http://genome-www4.stanford.edu/cgi-bin/SMD/source/sourceResult?choice=Gene&option=Name&criteria=COL5A1) | 1.56 | 0.0010 |
| A_24_P89257 | [ERGIC1](http://genome-www4.stanford.edu/cgi-bin/SMD/source/sourceResult?choice=Gene&option=Name&criteria=ERGIC1) | 1.54 | 0.0010 |
| A_23_P95917 | [HLA-C](http://genome-www4.stanford.edu/cgi-bin/SMD/source/sourceResult?choice=Gene&option=Name&criteria=HLA-C) | 1.57 | 0.0010 |
| A_24_P200652 | [C6orf62](http://genome-www4.stanford.edu/cgi-bin/SMD/source/sourceResult?choice=Gene&option=Name&criteria=C6orf62) | 1.72 | 0.0010 |
| A_23_P112162 | [DGAT1](http://genome-www4.stanford.edu/cgi-bin/SMD/source/sourceResult?choice=Gene&option=Name&criteria=DGAT1) | 1.76 | 0.0010 |
| A_23_P316812 | [AFARP1](http://genome-www4.stanford.edu/cgi-bin/SMD/source/sourceResult?choice=Gene&option=Name&criteria=AFARP1) | 1.59 | 0.0010 |
| A_24_P622697 | [A_24_P622697](http://genome-www4.stanford.edu/cgi-bin/SMD/source/sourceResult?choice=Gene&option=Name&criteria=A_24_P622697) | 1.51 | 0.0010 |
| A_23_P211926 | [WNT5A](http://genome-www4.stanford.edu/cgi-bin/SMD/source/sourceResult?choice=Gene&option=Name&criteria=WNT5A) | 1.71 | 0.0016 |
| A_24_P681011 | [BC041926](http://genome-www4.stanford.edu/cgi-bin/SMD/source/sourceResult?choice=Gene&option=Name&criteria=BC041926) | 1.52 | 0.0016 |
| A_23_P129312 | [PPP1R14D](http://genome-www4.stanford.edu/cgi-bin/SMD/source/sourceResult?choice=Gene&option=Name&criteria=PPP1R14D) | 1.62 | 0.0016 |
| A_23_P408353 | [HLA-A](http://genome-www4.stanford.edu/cgi-bin/SMD/source/sourceResult?choice=Gene&option=Name&criteria=HLA-A) | 1.51 | 0.0016 |
| A_24_P100266 | [ST13](http://genome-www4.stanford.edu/cgi-bin/SMD/source/sourceResult?choice=Gene&option=Name&criteria=ST13) | 1.55 | 0.0016 |
| A_24_P9605 | [PPP1R16A](http://genome-www4.stanford.edu/cgi-bin/SMD/source/sourceResult?choice=Gene&option=Name&criteria=PPP1R16A) | 1.56 | 0.0016 |
| A_32_P230398 | [THC2440435](http://genome-www4.stanford.edu/cgi-bin/SMD/source/sourceResult?choice=Gene&option=Name&criteria=THC2440435) | 1.56 | 0.0016 |
| A_23_P140830 | [ELMO3](http://genome-www4.stanford.edu/cgi-bin/SMD/source/sourceResult?choice=Gene&option=Name&criteria=ELMO3) | 1.64 | 0.0016 |
| A_23_P430842 | [HAPLN4](http://genome-www4.stanford.edu/cgi-bin/SMD/source/sourceResult?choice=Gene&option=Name&criteria=HAPLN4) | 1.95 | 0.0016 |
| A_23_P147995 | [PICALM](http://genome-www4.stanford.edu/cgi-bin/SMD/source/sourceResult?choice=Gene&option=Name&criteria=PICALM) | 1.58 | 0.0016 |
| A_23_P70355 | [SERPINB6](http://genome-www4.stanford.edu/cgi-bin/SMD/source/sourceResult?choice=Gene&option=Name&criteria=SERPINB6) | 1.51 | 0.0016 |
| A_23_P49351 | [AMDHD2](http://genome-www4.stanford.edu/cgi-bin/SMD/source/sourceResult?choice=Gene&option=Name&criteria=AMDHD2) | 1.76 | 0.0016 |
| A_32_P19135 | [RAB4B](http://genome-www4.stanford.edu/cgi-bin/SMD/source/sourceResult?choice=Gene&option=Name&criteria=RAB4B) | 1.56 | 0.0016 |
| A_23_P19061 | [DC-UbP](http://genome-www4.stanford.edu/cgi-bin/SMD/source/sourceResult?choice=Gene&option=Name&criteria=DC-UbP) | 1.63 | 0.0016 |
| A_23_P343719 | [PLCB3](http://genome-www4.stanford.edu/cgi-bin/SMD/source/sourceResult?choice=Gene&option=Name&criteria=PLCB3) | 1.76 | 0.0016 |
| A_24_P68814 | [DQX1](http://genome-www4.stanford.edu/cgi-bin/SMD/source/sourceResult?choice=Gene&option=Name&criteria=DQX1) | 1.61 | 0.0016 |
| A_23_P392544 | [CTAGE3](http://genome-www4.stanford.edu/cgi-bin/SMD/source/sourceResult?choice=Gene&option=Name&criteria=CTAGE3) | 1.53 | 0.0016 |
| A_23_P95330 | [ZDHHC9](http://genome-www4.stanford.edu/cgi-bin/SMD/source/sourceResult?choice=Gene&option=Name&criteria=ZDHHC9) | 1.67 | 0.0016 |
| A_23_P99974 | [CLK3](http://genome-www4.stanford.edu/cgi-bin/SMD/source/sourceResult?choice=Gene&option=Name&criteria=CLK3) | 1.50 | 0.0016 |
| A_24_P740620 | [ENST00000382184](http://genome-www4.stanford.edu/cgi-bin/SMD/source/sourceResult?choice=Gene&option=Name&criteria=ENST00000382184) | 1.92 | 0.0016 |
| A_23_P126248 | [RNF186](http://genome-www4.stanford.edu/cgi-bin/SMD/source/sourceResult?choice=Gene&option=Name&criteria=RNF186) | 1.83 | 0.0016 |
| A_24_P399622 | [COPE](http://genome-www4.stanford.edu/cgi-bin/SMD/source/sourceResult?choice=Gene&option=Name&criteria=COPE) | 1.65 | 0.0016 |
| A_23_P106773 | [SULT1A2](http://genome-www4.stanford.edu/cgi-bin/SMD/source/sourceResult?choice=Gene&option=Name&criteria=SULT1A2) | 1.55 | 0.0016 |
| A_23_P428298 | [UNC5CL](http://genome-www4.stanford.edu/cgi-bin/SMD/source/sourceResult?choice=Gene&option=Name&criteria=UNC5CL) | 1.88 | 0.0016 |
| A_23_P128817 | [PCK2](http://genome-www4.stanford.edu/cgi-bin/SMD/source/sourceResult?choice=Gene&option=Name&criteria=PCK2) | 1.79 | 0.0016 |
| A_23_P250035 | [SDHA](http://genome-www4.stanford.edu/cgi-bin/SMD/source/sourceResult?choice=Gene&option=Name&criteria=SDHA) | 1.59 | 0.0016 |
| A_23_P37910 | [MAPK3](http://genome-www4.stanford.edu/cgi-bin/SMD/source/sourceResult?choice=Gene&option=Name&criteria=MAPK3) | 1.65 | 0.0016 |
| A_23_P310274 | [PRSS2](http://genome-www4.stanford.edu/cgi-bin/SMD/source/sourceResult?choice=Gene&option=Name&criteria=PRSS2) | 1.61 | 0.0016 |
| A_24_P526190 | [LOC643201](http://genome-www4.stanford.edu/cgi-bin/SMD/source/sourceResult?choice=Gene&option=Name&criteria=LOC643201) | 1.60 | 0.0016 |
| A_24_P169574 | [A_24_P169574](http://genome-www4.stanford.edu/cgi-bin/SMD/source/sourceResult?choice=Gene&option=Name&criteria=A_24_P169574) | 1.72 | 0.0016 |
| A_23_P321388 | [IBRDC3](http://genome-www4.stanford.edu/cgi-bin/SMD/source/sourceResult?choice=Gene&option=Name&criteria=IBRDC3) | 1.66 | 0.0016 |
| A_24_P302332 | [NECAP2](http://genome-www4.stanford.edu/cgi-bin/SMD/source/sourceResult?choice=Gene&option=Name&criteria=NECAP2) | 1.54 | 0.0016 |
| A_23_P91859 | [ENST00000301807](http://genome-www4.stanford.edu/cgi-bin/SMD/source/sourceResult?choice=Gene&option=Name&criteria=ENST00000301807) | 1.58 | 0.0016 |
| A_23_P155624 | [AP2M1](http://genome-www4.stanford.edu/cgi-bin/SMD/source/sourceResult?choice=Gene&option=Name&criteria=AP2M1) | 1.61 | 0.0016 |
| A_24_P24645 | [A_24_P24645](http://genome-www4.stanford.edu/cgi-bin/SMD/source/sourceResult?choice=Gene&option=Name&criteria=A_24_P24645) | 1.56 | 0.0016 |
| A_23_P167367 | [PITX2](http://genome-www4.stanford.edu/cgi-bin/SMD/source/sourceResult?choice=Gene&option=Name&criteria=PITX2) | 1.97 | 0.0016 |
| A_23_P209944 | [RETSAT](http://genome-www4.stanford.edu/cgi-bin/SMD/source/sourceResult?choice=Gene&option=Name&criteria=RETSAT) | 1.65 | 0.0016 |
| A_24_P362904 | [PFKFB4](http://genome-www4.stanford.edu/cgi-bin/SMD/source/sourceResult?choice=Gene&option=Name&criteria=PFKFB4) | 1.81 | 0.0016 |
| A_23_P200928 | [NID1](http://genome-www4.stanford.edu/cgi-bin/SMD/source/sourceResult?choice=Gene&option=Name&criteria=NID1) | 1.56 | 0.0016 |
| A_23_P168864 | [ZNF16](http://genome-www4.stanford.edu/cgi-bin/SMD/source/sourceResult?choice=Gene&option=Name&criteria=ZNF16) | 1.58 | 0.0016 |
| A_23_P106024 | [JAG2](http://genome-www4.stanford.edu/cgi-bin/SMD/source/sourceResult?choice=Gene&option=Name&criteria=JAG2) | 1.54 | 0.0016 |
| A_23_P252449 | [HNRPA0](http://genome-www4.stanford.edu/cgi-bin/SMD/source/sourceResult?choice=Gene&option=Name&criteria=HNRPA0) | 1.74 | 0.0016 |
| A_23_P155868 | [PGRMC2](http://genome-www4.stanford.edu/cgi-bin/SMD/source/sourceResult?choice=Gene&option=Name&criteria=PGRMC2) | 1.58 | 0.0016 |
| A_23_P253012 | [GRAMD1C](http://genome-www4.stanford.edu/cgi-bin/SMD/source/sourceResult?choice=Gene&option=Name&criteria=GRAMD1C) | 1.71 | 0.0016 |
| A_23_P217326 | [FHL1](http://genome-www4.stanford.edu/cgi-bin/SMD/source/sourceResult?choice=Gene&option=Name&criteria=FHL1) | 1.56 | 0.0016 |
| A_24_P137602 | [CCDC100](http://genome-www4.stanford.edu/cgi-bin/SMD/source/sourceResult?choice=Gene&option=Name&criteria=CCDC100) | 1.57 | 0.0016 |
| A_23_P127054 | [PANK1](http://genome-www4.stanford.edu/cgi-bin/SMD/source/sourceResult?choice=Gene&option=Name&criteria=PANK1) | 1.64 | 0.0016 |
| A_24_P330263 | [EDNRB](http://genome-www4.stanford.edu/cgi-bin/SMD/source/sourceResult?choice=Gene&option=Name&criteria=EDNRB) | 2.24 | 0.0016 |
| A_23_P131299 | [ZFAND2B](http://genome-www4.stanford.edu/cgi-bin/SMD/source/sourceResult?choice=Gene&option=Name&criteria=ZFAND2B) | 1.57 | 0.0016 |
| A_24_P230675 | [SOCS2](http://genome-www4.stanford.edu/cgi-bin/SMD/source/sourceResult?choice=Gene&option=Name&criteria=SOCS2) | 1.59 | 0.0016 |
| A_24_P404245 | [PCYT2](http://genome-www4.stanford.edu/cgi-bin/SMD/source/sourceResult?choice=Gene&option=Name&criteria=PCYT2) | 1.52 | 0.0016 |
| A_23_P43763 | [PLLP](http://genome-www4.stanford.edu/cgi-bin/SMD/source/sourceResult?choice=Gene&option=Name&criteria=PLLP) | 1.60 | 0.0016 |
| A_23_P112482 | [AQP3](http://genome-www4.stanford.edu/cgi-bin/SMD/source/sourceResult?choice=Gene&option=Name&criteria=AQP3) | 1.85 | 0.0016 |
| A_24_P89987 | [EPB41L4B](http://genome-www4.stanford.edu/cgi-bin/SMD/source/sourceResult?choice=Gene&option=Name&criteria=EPB41L4B) | 1.69 | 0.0016 |
| A_24_P208909 | [TRIM2](http://genome-www4.stanford.edu/cgi-bin/SMD/source/sourceResult?choice=Gene&option=Name&criteria=TRIM2) | 1.51 | 0.0016 |
| A_23_P14939 | [TXNDC11](http://genome-www4.stanford.edu/cgi-bin/SMD/source/sourceResult?choice=Gene&option=Name&criteria=TXNDC11) | 1.56 | 0.0016 |
| A_24_P231494 | [DNPEP](http://genome-www4.stanford.edu/cgi-bin/SMD/source/sourceResult?choice=Gene&option=Name&criteria=DNPEP) | 1.54 | 0.0016 |
| A_23_P158484 | [CYP2C19](http://genome-www4.stanford.edu/cgi-bin/SMD/source/sourceResult?choice=Gene&option=Name&criteria=CYP2C19) | 2.03 | 0.0016 |
| A_23_P203658 | [PICALM](http://genome-www4.stanford.edu/cgi-bin/SMD/source/sourceResult?choice=Gene&option=Name&criteria=PICALM) | 1.64 | 0.0016 |
| A_23_P105856 | [LOC283537](http://genome-www4.stanford.edu/cgi-bin/SMD/source/sourceResult?choice=Gene&option=Name&criteria=LOC283537) | 1.76 | 0.0016 |
| A_24_P123012 | [YWHAE](http://genome-www4.stanford.edu/cgi-bin/SMD/source/sourceResult?choice=Gene&option=Name&criteria=YWHAE) | 1.68 | 0.0016 |
| A_24_P65199 | [CDK10](http://genome-www4.stanford.edu/cgi-bin/SMD/source/sourceResult?choice=Gene&option=Name&criteria=CDK10) | 1.55 | 0.0016 |
| A_23_P88626 | [ANPEP](http://genome-www4.stanford.edu/cgi-bin/SMD/source/sourceResult?choice=Gene&option=Name&criteria=ANPEP) | 1.89 | 0.0016 |
| A_23_P6433 | [MB](http://genome-www4.stanford.edu/cgi-bin/SMD/source/sourceResult?choice=Gene&option=Name&criteria=MB) | 1.66 | 0.0016 |
| A_24_P383660 | [A_24_P383660](http://genome-www4.stanford.edu/cgi-bin/SMD/source/sourceResult?choice=Gene&option=Name&criteria=A_24_P383660) | 1.61 | 0.0016 |
| A_24_P243528 | [HLA-DPA1](http://genome-www4.stanford.edu/cgi-bin/SMD/source/sourceResult?choice=Gene&option=Name&criteria=HLA-DPA1) | 1.58 | 0.0016 |
| A_23_P58993 | [MOCS1](http://genome-www4.stanford.edu/cgi-bin/SMD/source/sourceResult?choice=Gene&option=Name&criteria=MOCS1) | 1.68 | 0.0016 |
| A_23_P379649 | [BMF](http://genome-www4.stanford.edu/cgi-bin/SMD/source/sourceResult?choice=Gene&option=Name&criteria=BMF) | 1.69 | 0.0016 |
| A_24_P222126 | [TH1L](http://genome-www4.stanford.edu/cgi-bin/SMD/source/sourceResult?choice=Gene&option=Name&criteria=TH1L) | 1.53 | 0.0016 |
| A_23_P200001 | [NEXN](http://genome-www4.stanford.edu/cgi-bin/SMD/source/sourceResult?choice=Gene&option=Name&criteria=NEXN) | 1.53 | 0.0016 |
| A_23_P47818 | [CS](http://genome-www4.stanford.edu/cgi-bin/SMD/source/sourceResult?choice=Gene&option=Name&criteria=CS) | 1.56 | 0.0016 |
| A_23_P207456 | [CCL8](http://genome-www4.stanford.edu/cgi-bin/SMD/source/sourceResult?choice=Gene&option=Name&criteria=CCL8) | 1.83 | 0.0016 |
| A_23_P138776 | [ACP2](http://genome-www4.stanford.edu/cgi-bin/SMD/source/sourceResult?choice=Gene&option=Name&criteria=ACP2) | 1.56 | 0.0016 |
| A_23_P402313 | [FLJ35424](http://genome-www4.stanford.edu/cgi-bin/SMD/source/sourceResult?choice=Gene&option=Name&criteria=FLJ35424) | 1.86 | 0.0016 |
| A_24_P384604 | [A_24_P384604](http://genome-www4.stanford.edu/cgi-bin/SMD/source/sourceResult?choice=Gene&option=Name&criteria=A_24_P384604) | 1.97 | 0.0016 |
| A_23_P138782 | [ACP2](http://genome-www4.stanford.edu/cgi-bin/SMD/source/sourceResult?choice=Gene&option=Name&criteria=ACP2) | 1.52 | 0.0016 |
| A_23_P15146 | [IL32](http://genome-www4.stanford.edu/cgi-bin/SMD/source/sourceResult?choice=Gene&option=Name&criteria=IL32) | 1.51 | 0.0016 |
| A_23_P5983 | [PLTP](http://genome-www4.stanford.edu/cgi-bin/SMD/source/sourceResult?choice=Gene&option=Name&criteria=PLTP) | 1.53 | 0.0016 |
| A_24_P383704 | [LOC389599](http://genome-www4.stanford.edu/cgi-bin/SMD/source/sourceResult?choice=Gene&option=Name&criteria=LOC389599) | 1.79 | 0.0016 |
| A_23_P125109 | [A_23_P125109](http://genome-www4.stanford.edu/cgi-bin/SMD/source/sourceResult?choice=Gene&option=Name&criteria=A_23_P125109) | 1.51 | 0.0016 |
| A_24_P219769 | [FLJ10241](http://genome-www4.stanford.edu/cgi-bin/SMD/source/sourceResult?choice=Gene&option=Name&criteria=FLJ10241) | 1.52 | 0.0016 |
| A_23_P131308 | [CYP27A1](http://genome-www4.stanford.edu/cgi-bin/SMD/source/sourceResult?choice=Gene&option=Name&criteria=CYP27A1) | 1.83 | 0.0016 |
| A_24_P179244 | [AK092594](http://genome-www4.stanford.edu/cgi-bin/SMD/source/sourceResult?choice=Gene&option=Name&criteria=AK092594) | 1.50 | 0.0016 |
| A_24_P277875 | [CHN2](http://genome-www4.stanford.edu/cgi-bin/SMD/source/sourceResult?choice=Gene&option=Name&criteria=CHN2) | 1.64 | 0.0016 |
| A_23_P19894 | [AQP1](http://genome-www4.stanford.edu/cgi-bin/SMD/source/sourceResult?choice=Gene&option=Name&criteria=AQP1) | 1.54 | 0.0016 |
| A_24_P944570 | [PXDN](http://genome-www4.stanford.edu/cgi-bin/SMD/source/sourceResult?choice=Gene&option=Name&criteria=PXDN) | 1.54 | 0.0016 |
| A_32_P26103 | [AGPAT2](http://genome-www4.stanford.edu/cgi-bin/SMD/source/sourceResult?choice=Gene&option=Name&criteria=AGPAT2) | 1.96 | 0.0016 |
| A_23_P28238 | [SNX17](http://genome-www4.stanford.edu/cgi-bin/SMD/source/sourceResult?choice=Gene&option=Name&criteria=SNX17) | 1.71 | 0.0016 |
| A_23_P135454 | [AFG3L2](http://genome-www4.stanford.edu/cgi-bin/SMD/source/sourceResult?choice=Gene&option=Name&criteria=AFG3L2) | 1.59 | 0.0016 |
| A_23_P214554 | [TRIM15](http://genome-www4.stanford.edu/cgi-bin/SMD/source/sourceResult?choice=Gene&option=Name&criteria=TRIM15) | 1.75 | 0.0016 |
| A_23_P434929 | [CCDC100](http://genome-www4.stanford.edu/cgi-bin/SMD/source/sourceResult?choice=Gene&option=Name&criteria=CCDC100) | 1.64 | 0.0016 |
| A_23_P140207 | [PCK2](http://genome-www4.stanford.edu/cgi-bin/SMD/source/sourceResult?choice=Gene&option=Name&criteria=PCK2) | 1.63 | 0.0016 |
| A_23_P45999 | [FBXO2](http://genome-www4.stanford.edu/cgi-bin/SMD/source/sourceResult?choice=Gene&option=Name&criteria=FBXO2) | 1.74 | 0.0016 |
| A_23_P116235 | [MDK](http://genome-www4.stanford.edu/cgi-bin/SMD/source/sourceResult?choice=Gene&option=Name&criteria=MDK) | 1.58 | 0.0016 |
| A_24_P417352 | [IGHM](http://genome-www4.stanford.edu/cgi-bin/SMD/source/sourceResult?choice=Gene&option=Name&criteria=IGHM) | 1.77 | 0.0016 |
| A_24_P340036 | [RNF128](http://genome-www4.stanford.edu/cgi-bin/SMD/source/sourceResult?choice=Gene&option=Name&criteria=RNF128) | 1.82 | 0.0016 |
| A_24_P401392 | [A_24_P401392](http://genome-www4.stanford.edu/cgi-bin/SMD/source/sourceResult?choice=Gene&option=Name&criteria=A_24_P401392) | 1.59 | 0.0016 |
| A_24_P161733 | [A_24_P161733](http://genome-www4.stanford.edu/cgi-bin/SMD/source/sourceResult?choice=Gene&option=Name&criteria=A_24_P161733) | 1.59 | 0.0016 |
| A_24_P246626 | [ENST00000383097](http://genome-www4.stanford.edu/cgi-bin/SMD/source/sourceResult?choice=Gene&option=Name&criteria=ENST00000383097) | 1.58 | 0.0016 |
| A_24_P110012 | [FLJ45422](http://genome-www4.stanford.edu/cgi-bin/SMD/source/sourceResult?choice=Gene&option=Name&criteria=FLJ45422) | 1.83 | 0.0016 |
| A_24_P1054 | [NFKBIL2](http://genome-www4.stanford.edu/cgi-bin/SMD/source/sourceResult?choice=Gene&option=Name&criteria=NFKBIL2) | 1.56 | 0.0021 |
| A_23_P55990 | [NAPA](http://genome-www4.stanford.edu/cgi-bin/SMD/source/sourceResult?choice=Gene&option=Name&criteria=NAPA) | 1.56 | 0.0021 |
| A_23_P434710 | [PPP1CA](http://genome-www4.stanford.edu/cgi-bin/SMD/source/sourceResult?choice=Gene&option=Name&criteria=PPP1CA) | 1.59 | 0.0021 |
| A_23_P99320 | [KRT18](http://genome-www4.stanford.edu/cgi-bin/SMD/source/sourceResult?choice=Gene&option=Name&criteria=KRT18) | 1.54 | 0.0021 |
| A_23_P77103 | [SORD](http://genome-www4.stanford.edu/cgi-bin/SMD/source/sourceResult?choice=Gene&option=Name&criteria=SORD) | 1.57 | 0.0021 |
| A_24_P185117 | [RILP](http://genome-www4.stanford.edu/cgi-bin/SMD/source/sourceResult?choice=Gene&option=Name&criteria=RILP) | 1.51 | 0.0021 |
| A_32_P77762 | [LOC642680](http://genome-www4.stanford.edu/cgi-bin/SMD/source/sourceResult?choice=Gene&option=Name&criteria=LOC642680) | 1.64 | 0.0021 |
| A_23_P202510 | [ASB13](http://genome-www4.stanford.edu/cgi-bin/SMD/source/sourceResult?choice=Gene&option=Name&criteria=ASB13) | 1.50 | 0.0021 |
| A_23_P1352 | [SFRP5](http://genome-www4.stanford.edu/cgi-bin/SMD/source/sourceResult?choice=Gene&option=Name&criteria=SFRP5) | 1.69 | 0.0021 |
| A_23_P34478 | [GIPC2](http://genome-www4.stanford.edu/cgi-bin/SMD/source/sourceResult?choice=Gene&option=Name&criteria=GIPC2) | 1.52 | 0.0021 |
| A_24_P75979 | [LOC402643](http://genome-www4.stanford.edu/cgi-bin/SMD/source/sourceResult?choice=Gene&option=Name&criteria=LOC402643) | 1.50 | 0.0021 |
| A_24_P917711 | [PRKAB2](http://genome-www4.stanford.edu/cgi-bin/SMD/source/sourceResult?choice=Gene&option=Name&criteria=PRKAB2) | 1.77 | 0.0021 |
| A_23_P122563 | [PFDN6](http://genome-www4.stanford.edu/cgi-bin/SMD/source/sourceResult?choice=Gene&option=Name&criteria=PFDN6) | 1.57 | 0.0021 |
| A_23_P310590 | [OAF](http://genome-www4.stanford.edu/cgi-bin/SMD/source/sourceResult?choice=Gene&option=Name&criteria=OAF) | 1.61 | 0.0021 |
| A_24_P595369 | [LOC440895](http://genome-www4.stanford.edu/cgi-bin/SMD/source/sourceResult?choice=Gene&option=Name&criteria=LOC440895) | 1.62 | 0.0021 |
| A_24_P385134 | [SCD5](http://genome-www4.stanford.edu/cgi-bin/SMD/source/sourceResult?choice=Gene&option=Name&criteria=SCD5) | 1.56 | 0.0021 |
| A_23_P363831 | [FBXO3](http://genome-www4.stanford.edu/cgi-bin/SMD/source/sourceResult?choice=Gene&option=Name&criteria=FBXO3) | 1.58 | 0.0021 |
| A_23_P156431 | [MAN1A1](http://genome-www4.stanford.edu/cgi-bin/SMD/source/sourceResult?choice=Gene&option=Name&criteria=MAN1A1) | 1.64 | 0.0021 |
| A_32_P202703 | [LOC389831](http://genome-www4.stanford.edu/cgi-bin/SMD/source/sourceResult?choice=Gene&option=Name&criteria=LOC389831) | 1.73 | 0.0021 |
| A_23_P338113 | [NP101106](http://genome-www4.stanford.edu/cgi-bin/SMD/source/sourceResult?choice=Gene&option=Name&criteria=NP101106) | 1.53 | 0.0021 |
| A_24_P260325 | [LRAT](http://genome-www4.stanford.edu/cgi-bin/SMD/source/sourceResult?choice=Gene&option=Name&criteria=LRAT) | 1.59 | 0.0021 |
| A_23_P12405 | [ESPN](http://genome-www4.stanford.edu/cgi-bin/SMD/source/sourceResult?choice=Gene&option=Name&criteria=ESPN) | 1.57 | 0.0021 |
| A_23_P130619 | [STXBP2](http://genome-www4.stanford.edu/cgi-bin/SMD/source/sourceResult?choice=Gene&option=Name&criteria=STXBP2) | 1.61 | 0.0021 |
| A_24_P229726 | [A_24_P229726](http://genome-www4.stanford.edu/cgi-bin/SMD/source/sourceResult?choice=Gene&option=Name&criteria=A_24_P229726) | 1.68 | 0.0021 |
| A_24_P15702 | [A_24_P15702](http://genome-www4.stanford.edu/cgi-bin/SMD/source/sourceResult?choice=Gene&option=Name&criteria=A_24_P15702) | 1.52 | 0.0021 |
| A_23_P251453 | [HNF4G](http://genome-www4.stanford.edu/cgi-bin/SMD/source/sourceResult?choice=Gene&option=Name&criteria=HNF4G) | 2.09 | 0.0021 |
| A_23_P37598 | [NPTN](http://genome-www4.stanford.edu/cgi-bin/SMD/source/sourceResult?choice=Gene&option=Name&criteria=NPTN) | 1.57 | 0.0021 |
| A_24_P195164 | [A_24_P195164](http://genome-www4.stanford.edu/cgi-bin/SMD/source/sourceResult?choice=Gene&option=Name&criteria=A_24_P195164) | 1.59 | 0.0021 |
| A_24_P99795 | [ISOC2](http://genome-www4.stanford.edu/cgi-bin/SMD/source/sourceResult?choice=Gene&option=Name&criteria=ISOC2) | 1.58 | 0.0021 |
| A_24_P22488 | [AXIN1](http://genome-www4.stanford.edu/cgi-bin/SMD/source/sourceResult?choice=Gene&option=Name&criteria=AXIN1) | 1.66 | 0.0021 |
| A_24_P418717 | [BMPR1A](http://genome-www4.stanford.edu/cgi-bin/SMD/source/sourceResult?choice=Gene&option=Name&criteria=BMPR1A) | 1.73 | 0.0021 |
| A_23_P43238 | [NAPRT1](http://genome-www4.stanford.edu/cgi-bin/SMD/source/sourceResult?choice=Gene&option=Name&criteria=NAPRT1) | 1.70 | 0.0021 |
| A_23_P86424 | [NCOA4](http://genome-www4.stanford.edu/cgi-bin/SMD/source/sourceResult?choice=Gene&option=Name&criteria=NCOA4) | 1.64 | 0.0021 |
| A_23_P3963 | [CDR2L](http://genome-www4.stanford.edu/cgi-bin/SMD/source/sourceResult?choice=Gene&option=Name&criteria=CDR2L) | 1.57 | 0.0021 |
| A_23_P67913 | [GMPPA](http://genome-www4.stanford.edu/cgi-bin/SMD/source/sourceResult?choice=Gene&option=Name&criteria=GMPPA) | 1.59 | 0.0021 |
| A_23_P133799 | [KLC4](http://genome-www4.stanford.edu/cgi-bin/SMD/source/sourceResult?choice=Gene&option=Name&criteria=KLC4) | 1.51 | 0.0021 |
| A_23_P54963 | [MRPL38](http://genome-www4.stanford.edu/cgi-bin/SMD/source/sourceResult?choice=Gene&option=Name&criteria=MRPL38) | 1.52 | 0.0021 |
| A_24_P15550 | [AF035034](http://genome-www4.stanford.edu/cgi-bin/SMD/source/sourceResult?choice=Gene&option=Name&criteria=AF035034) | 1.77 | 0.0021 |
| A_23_P217917 | [GSTM4](http://genome-www4.stanford.edu/cgi-bin/SMD/source/sourceResult?choice=Gene&option=Name&criteria=GSTM4) | 1.53 | 0.0021 |
| A_23_P36448 | [TMED2](http://genome-www4.stanford.edu/cgi-bin/SMD/source/sourceResult?choice=Gene&option=Name&criteria=TMED2) | 1.64 | 0.0021 |
| A_23_P62890 | [GBP1](http://genome-www4.stanford.edu/cgi-bin/SMD/source/sourceResult?choice=Gene&option=Name&criteria=GBP1) | 1.71 | 0.0021 |
| A_23_P54781 | [RBBP6](http://genome-www4.stanford.edu/cgi-bin/SMD/source/sourceResult?choice=Gene&option=Name&criteria=RBBP6) | 1.59 | 0.0021 |
| A_23_P145336 | [HLA-DRB3](http://genome-www4.stanford.edu/cgi-bin/SMD/source/sourceResult?choice=Gene&option=Name&criteria=HLA-DRB3) | 1.74 | 0.0021 |
| A_24_P10226 | [SEMA6D](http://genome-www4.stanford.edu/cgi-bin/SMD/source/sourceResult?choice=Gene&option=Name&criteria=SEMA6D) | 1.73 | 0.0021 |
| A_24_P205364 | [SHMT1](http://genome-www4.stanford.edu/cgi-bin/SMD/source/sourceResult?choice=Gene&option=Name&criteria=SHMT1) | 1.53 | 0.0021 |
| A_24_P263786 | [BC022362](http://genome-www4.stanford.edu/cgi-bin/SMD/source/sourceResult?choice=Gene&option=Name&criteria=BC022362) | 1.99 | 0.0021 |
| A_23_P160433 | [C1orf115](http://genome-www4.stanford.edu/cgi-bin/SMD/source/sourceResult?choice=Gene&option=Name&criteria=C1orf115) | 1.65 | 0.0021 |
| A_23_P118615 | [ABCA8](http://genome-www4.stanford.edu/cgi-bin/SMD/source/sourceResult?choice=Gene&option=Name&criteria=ABCA8) | 1.90 | 0.0021 |
| A_23_P146631 | [ZYG11BL](http://genome-www4.stanford.edu/cgi-bin/SMD/source/sourceResult?choice=Gene&option=Name&criteria=ZYG11BL) | 1.51 | 0.0021 |
| A_23_P201483 | [MAPKAPK2](http://genome-www4.stanford.edu/cgi-bin/SMD/source/sourceResult?choice=Gene&option=Name&criteria=MAPKAPK2) | 1.57 | 0.0021 |
| A_23_P429998 | [FOSB](http://genome-www4.stanford.edu/cgi-bin/SMD/source/sourceResult?choice=Gene&option=Name&criteria=FOSB) | 1.95 | 0.0021 |
| A_23_P136026 | [IGHA1](http://genome-www4.stanford.edu/cgi-bin/SMD/source/sourceResult?choice=Gene&option=Name&criteria=IGHA1) | 1.80 | 0.0021 |
| A_24_P186379 | [C10orf125](http://genome-www4.stanford.edu/cgi-bin/SMD/source/sourceResult?choice=Gene&option=Name&criteria=C10orf125) | 1.55 | 0.0021 |
| A_24_P396662 | [GSTM4](http://genome-www4.stanford.edu/cgi-bin/SMD/source/sourceResult?choice=Gene&option=Name&criteria=GSTM4) | 1.62 | 0.0021 |
| A_24_P328320 | [GORASP2](http://genome-www4.stanford.edu/cgi-bin/SMD/source/sourceResult?choice=Gene&option=Name&criteria=GORASP2) | 1.71 | 0.0021 |
| A_23_P129064 | [GATM](http://genome-www4.stanford.edu/cgi-bin/SMD/source/sourceResult?choice=Gene&option=Name&criteria=GATM) | 1.86 | 0.0021 |
| A_24_P193570 | [CNOT1](http://genome-www4.stanford.edu/cgi-bin/SMD/source/sourceResult?choice=Gene&option=Name&criteria=CNOT1) | 1.52 | 0.0021 |
| A_23_P211267 | [RIPK4](http://genome-www4.stanford.edu/cgi-bin/SMD/source/sourceResult?choice=Gene&option=Name&criteria=RIPK4) | 1.53 | 0.0021 |
| A_24_P541576 | [LOC389831](http://genome-www4.stanford.edu/cgi-bin/SMD/source/sourceResult?choice=Gene&option=Name&criteria=LOC389831) | 1.70 | 0.0021 |
| A_24_P57207 | [CREB3L3](http://genome-www4.stanford.edu/cgi-bin/SMD/source/sourceResult?choice=Gene&option=Name&criteria=CREB3L3) | 1.58 | 0.0021 |
| A_23_P63447 | [THC2373845](http://genome-www4.stanford.edu/cgi-bin/SMD/source/sourceResult?choice=Gene&option=Name&criteria=THC2373845) | 1.54 | 0.0021 |
| A_24_P100301 | [GIPC2](http://genome-www4.stanford.edu/cgi-bin/SMD/source/sourceResult?choice=Gene&option=Name&criteria=GIPC2) | 1.57 | 0.0021 |
| A_24_P29260 | [MGAT4B](http://genome-www4.stanford.edu/cgi-bin/SMD/source/sourceResult?choice=Gene&option=Name&criteria=MGAT4B) | 1.68 | 0.0021 |
| A_23_P308519 | [SLC9A3R1](http://genome-www4.stanford.edu/cgi-bin/SMD/source/sourceResult?choice=Gene&option=Name&criteria=SLC9A3R1) | 1.56 | 0.0021 |
| A_24_P49183 | [FLJ20433](http://genome-www4.stanford.edu/cgi-bin/SMD/source/sourceResult?choice=Gene&option=Name&criteria=FLJ20433) | 1.58 | 0.0021 |
| A_23_P51918 | [LZTR2](http://genome-www4.stanford.edu/cgi-bin/SMD/source/sourceResult?choice=Gene&option=Name&criteria=LZTR2) | 1.54 | 0.0021 |
| A_23_P146922 | [GAS6](http://genome-www4.stanford.edu/cgi-bin/SMD/source/sourceResult?choice=Gene&option=Name&criteria=GAS6) | 1.83 | 0.0021 |
| A_23_P65022 | [ACADS](http://genome-www4.stanford.edu/cgi-bin/SMD/source/sourceResult?choice=Gene&option=Name&criteria=ACADS) | 1.77 | 0.0021 |
| A_23_P111766 | [A_23_P111766](http://genome-www4.stanford.edu/cgi-bin/SMD/source/sourceResult?choice=Gene&option=Name&criteria=A_23_P111766) | 1.71 | 0.0021 |
| A_24_P833256 | [THC2364469](http://genome-www4.stanford.edu/cgi-bin/SMD/source/sourceResult?choice=Gene&option=Name&criteria=THC2364469) | 2.01 | 0.0021 |
| A_23_P201086 | [ARF1](http://genome-www4.stanford.edu/cgi-bin/SMD/source/sourceResult?choice=Gene&option=Name&criteria=ARF1) | 1.69 | 0.0021 |
| A_24_P296280 | [FAM82C](http://genome-www4.stanford.edu/cgi-bin/SMD/source/sourceResult?choice=Gene&option=Name&criteria=FAM82C) | 1.66 | 0.0028 |
| A_23_P434421 | [C10orf9](http://genome-www4.stanford.edu/cgi-bin/SMD/source/sourceResult?choice=Gene&option=Name&criteria=C10orf9) | 1.55 | 0.0028 |
| A_23_P43296 | [FBXL6](http://genome-www4.stanford.edu/cgi-bin/SMD/source/sourceResult?choice=Gene&option=Name&criteria=FBXL6) | 1.51 | 0.0028 |
| A_23_P102364 | [NGEF](http://genome-www4.stanford.edu/cgi-bin/SMD/source/sourceResult?choice=Gene&option=Name&criteria=NGEF) | 1.51 | 0.0028 |
| A_23_P163079 | [GCH1](http://genome-www4.stanford.edu/cgi-bin/SMD/source/sourceResult?choice=Gene&option=Name&criteria=GCH1) | 1.62 | 0.0028 |
| A_23_P62133 | [MTM1](http://genome-www4.stanford.edu/cgi-bin/SMD/source/sourceResult?choice=Gene&option=Name&criteria=MTM1) | 1.55 | 0.0028 |
| A_32_P190944 | [A_32_P190944](http://genome-www4.stanford.edu/cgi-bin/SMD/source/sourceResult?choice=Gene&option=Name&criteria=A_32_P190944) | 1.74 | 0.0028 |
| A_23_P109508 | [NCF4](http://genome-www4.stanford.edu/cgi-bin/SMD/source/sourceResult?choice=Gene&option=Name&criteria=NCF4) | 1.59 | 0.0028 |
| A_23_P120125 | [COLEC11](http://genome-www4.stanford.edu/cgi-bin/SMD/source/sourceResult?choice=Gene&option=Name&criteria=COLEC11) | 1.63 | 0.0028 |
| A_32_P44568 | [LDHA](http://genome-www4.stanford.edu/cgi-bin/SMD/source/sourceResult?choice=Gene&option=Name&criteria=LDHA) | 1.54 | 0.0028 |
| A_24_P66578 | [ENST00000310579](http://genome-www4.stanford.edu/cgi-bin/SMD/source/sourceResult?choice=Gene&option=Name&criteria=ENST00000310579) | 1.75 | 0.0028 |
| A_32_P39216 | [YWHAE](http://genome-www4.stanford.edu/cgi-bin/SMD/source/sourceResult?choice=Gene&option=Name&criteria=YWHAE) | 1.56 | 0.0028 |
| A_24_P14634 | [EMID1](http://genome-www4.stanford.edu/cgi-bin/SMD/source/sourceResult?choice=Gene&option=Name&criteria=EMID1) | 1.50 | 0.0028 |
| A_24_P63950 | [AP1S1](http://genome-www4.stanford.edu/cgi-bin/SMD/source/sourceResult?choice=Gene&option=Name&criteria=AP1S1) | 1.63 | 0.0028 |
| A_23_P97339 | [SLC16A4](http://genome-www4.stanford.edu/cgi-bin/SMD/source/sourceResult?choice=Gene&option=Name&criteria=SLC16A4) | 1.67 | 0.0028 |
| A_23_P98092 | [OAT](http://genome-www4.stanford.edu/cgi-bin/SMD/source/sourceResult?choice=Gene&option=Name&criteria=OAT) | 2.14 | 0.0028 |
| A_23_P112554 | [COL15A1](http://genome-www4.stanford.edu/cgi-bin/SMD/source/sourceResult?choice=Gene&option=Name&criteria=COL15A1) | 1.55 | 0.0028 |
| A_24_P451992 | [A_24_P451992](http://genome-www4.stanford.edu/cgi-bin/SMD/source/sourceResult?choice=Gene&option=Name&criteria=A_24_P451992) | 1.51 | 0.0028 |
| A_23_P113682 | [SLC34A3](http://genome-www4.stanford.edu/cgi-bin/SMD/source/sourceResult?choice=Gene&option=Name&criteria=SLC34A3) | 1.56 | 0.0028 |
| A_32_P7015 | [TSPAN15](http://genome-www4.stanford.edu/cgi-bin/SMD/source/sourceResult?choice=Gene&option=Name&criteria=TSPAN15) | 1.63 | 0.0028 |
| A_23_P10077 | [PNPLA2](http://genome-www4.stanford.edu/cgi-bin/SMD/source/sourceResult?choice=Gene&option=Name&criteria=PNPLA2) | 1.59 | 0.0028 |
| A_23_P203841 | [BAZ2A](http://genome-www4.stanford.edu/cgi-bin/SMD/source/sourceResult?choice=Gene&option=Name&criteria=BAZ2A) | 1.53 | 0.0028 |
| A_24_P337380 | [HNRPH1](http://genome-www4.stanford.edu/cgi-bin/SMD/source/sourceResult?choice=Gene&option=Name&criteria=HNRPH1) | 1.54 | 0.0028 |
| A_24_P402222 | [HLA-DRB3](http://genome-www4.stanford.edu/cgi-bin/SMD/source/sourceResult?choice=Gene&option=Name&criteria=HLA-DRB3) | 1.62 | 0.0028 |
| A_32_P183904 | [ENST00000361989](http://genome-www4.stanford.edu/cgi-bin/SMD/source/sourceResult?choice=Gene&option=Name&criteria=ENST00000361989) | 1.64 | 0.0028 |
| A_23_P107412 | [P4HB](http://genome-www4.stanford.edu/cgi-bin/SMD/source/sourceResult?choice=Gene&option=Name&criteria=P4HB) | 1.65 | 0.0028 |
| A_23_P211850 | [ABHD6](http://genome-www4.stanford.edu/cgi-bin/SMD/source/sourceResult?choice=Gene&option=Name&criteria=ABHD6) | 1.60 | 0.0028 |
| A_23_P15857 | [PPP4R1](http://genome-www4.stanford.edu/cgi-bin/SMD/source/sourceResult?choice=Gene&option=Name&criteria=PPP4R1) | 1.54 | 0.0028 |
| A_32_P163169 | [VDAC1](http://genome-www4.stanford.edu/cgi-bin/SMD/source/sourceResult?choice=Gene&option=Name&criteria=VDAC1) | 1.52 | 0.0028 |
| A_23_P32500 | [STAB1](http://genome-www4.stanford.edu/cgi-bin/SMD/source/sourceResult?choice=Gene&option=Name&criteria=STAB1) | 1.56 | 0.0028 |
| A_32_P148710 | [CFL1](http://genome-www4.stanford.edu/cgi-bin/SMD/source/sourceResult?choice=Gene&option=Name&criteria=CFL1) | 1.60 | 0.0028 |
| A_24_P328471 | [PLEKHA3](http://genome-www4.stanford.edu/cgi-bin/SMD/source/sourceResult?choice=Gene&option=Name&criteria=PLEKHA3) | 1.66 | 0.0028 |
| A_23_P218111 | [SERPINA1](http://genome-www4.stanford.edu/cgi-bin/SMD/source/sourceResult?choice=Gene&option=Name&criteria=SERPINA1) | 1.66 | 0.0028 |
| A_32_P13392 | [THC2340668](http://genome-www4.stanford.edu/cgi-bin/SMD/source/sourceResult?choice=Gene&option=Name&criteria=THC2340668) | 1.52 | 0.0028 |
| A_23_P319572 | [NR1I3](http://genome-www4.stanford.edu/cgi-bin/SMD/source/sourceResult?choice=Gene&option=Name&criteria=NR1I3) | 1.78 | 0.0028 |
| A_23_P108082 | [CREB3L3](http://genome-www4.stanford.edu/cgi-bin/SMD/source/sourceResult?choice=Gene&option=Name&criteria=CREB3L3) | 2.09 | 0.0028 |
| A_23_P152245 | [CDK10](http://genome-www4.stanford.edu/cgi-bin/SMD/source/sourceResult?choice=Gene&option=Name&criteria=CDK10) | 1.58 | 0.0028 |
| A_23_P211212 | [COL18A1](http://genome-www4.stanford.edu/cgi-bin/SMD/source/sourceResult?choice=Gene&option=Name&criteria=COL18A1) | 1.51 | 0.0028 |
| A_24_P702749 | [AF471454](http://genome-www4.stanford.edu/cgi-bin/SMD/source/sourceResult?choice=Gene&option=Name&criteria=AF471454) | 1.88 | 0.0028 |
| A_23_P121051 | [PCCB](http://genome-www4.stanford.edu/cgi-bin/SMD/source/sourceResult?choice=Gene&option=Name&criteria=PCCB) | 1.51 | 0.0028 |
| A_24_P46066 | [CXCR6](http://genome-www4.stanford.edu/cgi-bin/SMD/source/sourceResult?choice=Gene&option=Name&criteria=CXCR6) | 1.56 | 0.0028 |
| A_24_P843309 | [A_24_P843309](http://genome-www4.stanford.edu/cgi-bin/SMD/source/sourceResult?choice=Gene&option=Name&criteria=A_24_P843309) | 1.57 | 0.0028 |
| A_23_P39590 | [XDH](http://genome-www4.stanford.edu/cgi-bin/SMD/source/sourceResult?choice=Gene&option=Name&criteria=XDH) | 1.64 | 0.0028 |
| A_23_P147326 | [SERINC2](http://genome-www4.stanford.edu/cgi-bin/SMD/source/sourceResult?choice=Gene&option=Name&criteria=SERINC2) | 1.64 | 0.0028 |
| A_32_P532216 | [QARS](http://genome-www4.stanford.edu/cgi-bin/SMD/source/sourceResult?choice=Gene&option=Name&criteria=QARS) | 1.56 | 0.0028 |
| A_24_P315941 | [D83692](http://genome-www4.stanford.edu/cgi-bin/SMD/source/sourceResult?choice=Gene&option=Name&criteria=D83692) | 1.98 | 0.0028 |
| A_24_P396660 | [GSTM4](http://genome-www4.stanford.edu/cgi-bin/SMD/source/sourceResult?choice=Gene&option=Name&criteria=GSTM4) | 1.62 | 0.0028 |
| A_23_P108294 | [PPAP2C](http://genome-www4.stanford.edu/cgi-bin/SMD/source/sourceResult?choice=Gene&option=Name&criteria=PPAP2C) | 1.59 | 0.0028 |
| A_23_P326963 | [C3orf59](http://genome-www4.stanford.edu/cgi-bin/SMD/source/sourceResult?choice=Gene&option=Name&criteria=C3orf59) | 1.55 | 0.0028 |
| A_23_P396062 | [RAB40C](http://genome-www4.stanford.edu/cgi-bin/SMD/source/sourceResult?choice=Gene&option=Name&criteria=RAB40C) | 1.55 | 0.0028 |
| A_24_P750817 | [LOC147710](http://genome-www4.stanford.edu/cgi-bin/SMD/source/sourceResult?choice=Gene&option=Name&criteria=LOC147710) | 1.63 | 0.0028 |
| A_24_P281374 | [A_24_P281374](http://genome-www4.stanford.edu/cgi-bin/SMD/source/sourceResult?choice=Gene&option=Name&criteria=A_24_P281374) | 1.50 | 0.0028 |
| A_23_P39465 | [BST2](http://genome-www4.stanford.edu/cgi-bin/SMD/source/sourceResult?choice=Gene&option=Name&criteria=BST2) | 1.59 | 0.0028 |
| A_23_P373708 | [FLJ40504](http://genome-www4.stanford.edu/cgi-bin/SMD/source/sourceResult?choice=Gene&option=Name&criteria=FLJ40504) | 1.54 | 0.0028 |
| A_24_P170283 | [A_24_P170283](http://genome-www4.stanford.edu/cgi-bin/SMD/source/sourceResult?choice=Gene&option=Name&criteria=A_24_P170283) | 1.58 | 0.0028 |
| A_32_P214011 | [THC2277187](http://genome-www4.stanford.edu/cgi-bin/SMD/source/sourceResult?choice=Gene&option=Name&criteria=THC2277187) | 1.66 | 0.0028 |
| A_23_P141126 | [GALK1](http://genome-www4.stanford.edu/cgi-bin/SMD/source/sourceResult?choice=Gene&option=Name&criteria=GALK1) | 1.63 | 0.0028 |
| A_23_P215505 | [RAPGEF5](http://genome-www4.stanford.edu/cgi-bin/SMD/source/sourceResult?choice=Gene&option=Name&criteria=RAPGEF5) | 1.98 | 0.0028 |
| A_24_P830696 | [THC2373624](http://genome-www4.stanford.edu/cgi-bin/SMD/source/sourceResult?choice=Gene&option=Name&criteria=THC2373624) | 1.55 | 0.0028 |
| A_23_P201619 | [ENST00000367385](http://genome-www4.stanford.edu/cgi-bin/SMD/source/sourceResult?choice=Gene&option=Name&criteria=ENST00000367385) | 1.65 | 0.0028 |
| A_23_P48455 | [AMN](http://genome-www4.stanford.edu/cgi-bin/SMD/source/sourceResult?choice=Gene&option=Name&criteria=AMN) | 1.62 | 0.0028 |
| A_23_P347432 | [DVL1](http://genome-www4.stanford.edu/cgi-bin/SMD/source/sourceResult?choice=Gene&option=Name&criteria=DVL1) | 1.63 | 0.0028 |
| A_23_P58953 | [NQO2](http://genome-www4.stanford.edu/cgi-bin/SMD/source/sourceResult?choice=Gene&option=Name&criteria=NQO2) | 1.52 | 0.0028 |
| A_23_P167168 | [IGJ](http://genome-www4.stanford.edu/cgi-bin/SMD/source/sourceResult?choice=Gene&option=Name&criteria=IGJ) | 1.82 | 0.0028 |
| A_24_P764598 | [PTP4A2](http://genome-www4.stanford.edu/cgi-bin/SMD/source/sourceResult?choice=Gene&option=Name&criteria=PTP4A2) | 1.57 | 0.0028 |
| A_23_P7562 | [ACSL6](http://genome-www4.stanford.edu/cgi-bin/SMD/source/sourceResult?choice=Gene&option=Name&criteria=ACSL6) | 1.55 | 0.0028 |
| A_23_P354609 | [AK095583](http://genome-www4.stanford.edu/cgi-bin/SMD/source/sourceResult?choice=Gene&option=Name&criteria=AK095583) | 1.57 | 0.0028 |
| A_23_P5845 | [KHK](http://genome-www4.stanford.edu/cgi-bin/SMD/source/sourceResult?choice=Gene&option=Name&criteria=KHK) | 1.76 | 0.0028 |
| A_23_P255345 | [VNN1](http://genome-www4.stanford.edu/cgi-bin/SMD/source/sourceResult?choice=Gene&option=Name&criteria=VNN1) | 2.29 | 0.0028 |
| A_24_P387609 | [HBLD2](http://genome-www4.stanford.edu/cgi-bin/SMD/source/sourceResult?choice=Gene&option=Name&criteria=HBLD2) | 1.54 | 0.0028 |
| A_24_P300952 | [APLP2](http://genome-www4.stanford.edu/cgi-bin/SMD/source/sourceResult?choice=Gene&option=Name&criteria=APLP2) | 1.58 | 0.0028 |
| A_23_P66635 | [CCL11](http://genome-www4.stanford.edu/cgi-bin/SMD/source/sourceResult?choice=Gene&option=Name&criteria=CCL11) | 1.66 | 0.0028 |
| A_32_P94444 | [PRSS2](http://genome-www4.stanford.edu/cgi-bin/SMD/source/sourceResult?choice=Gene&option=Name&criteria=PRSS2) | 1.76 | 0.0028 |
| A_23_P305759 | [ABHD3](http://genome-www4.stanford.edu/cgi-bin/SMD/source/sourceResult?choice=Gene&option=Name&criteria=ABHD3) | 1.56 | 0.0028 |
| A_32_P722809 | [IGKV1-5](http://genome-www4.stanford.edu/cgi-bin/SMD/source/sourceResult?choice=Gene&option=Name&criteria=IGKV1-5) | 1.78 | 0.0028 |
| A_24_P67395 | [KRT8](http://genome-www4.stanford.edu/cgi-bin/SMD/source/sourceResult?choice=Gene&option=Name&criteria=KRT8) | 1.61 | 0.0028 |
| A_24_P323682 | [ENST00000361859](http://genome-www4.stanford.edu/cgi-bin/SMD/source/sourceResult?choice=Gene&option=Name&criteria=ENST00000361859) | 1.67 | 0.0028 |
| A_24_P226198 | [RAD50](http://genome-www4.stanford.edu/cgi-bin/SMD/source/sourceResult?choice=Gene&option=Name&criteria=RAD50) | 1.54 | 0.0028 |
| A_23_P156687 | [CFB](http://genome-www4.stanford.edu/cgi-bin/SMD/source/sourceResult?choice=Gene&option=Name&criteria=CFB) | 1.65 | 0.0036 |
| A_23_P383009 | [IGFBP5](http://genome-www4.stanford.edu/cgi-bin/SMD/source/sourceResult?choice=Gene&option=Name&criteria=IGFBP5) | 1.61 | 0.0036 |
| A_23_P125977 | [C1QC](http://genome-www4.stanford.edu/cgi-bin/SMD/source/sourceResult?choice=Gene&option=Name&criteria=C1QC) | 1.60 | 0.0036 |
| A_23_P202275 | [PRAP1](http://genome-www4.stanford.edu/cgi-bin/SMD/source/sourceResult?choice=Gene&option=Name&criteria=PRAP1) | 1.87 | 0.0036 |
| A_23_P170719 | [A_23_P170719](http://genome-www4.stanford.edu/cgi-bin/SMD/source/sourceResult?choice=Gene&option=Name&criteria=A_23_P170719) | 1.75 | 0.0036 |
| A_24_P686014 | [A_24_P686014](http://genome-www4.stanford.edu/cgi-bin/SMD/source/sourceResult?choice=Gene&option=Name&criteria=A_24_P686014) | 1.54 | 0.0036 |
| A_23_P98876 | [SLC39A5](http://genome-www4.stanford.edu/cgi-bin/SMD/source/sourceResult?choice=Gene&option=Name&criteria=SLC39A5) | 1.57 | 0.0036 |
| A_24_P386622 | [ARRB1](http://genome-www4.stanford.edu/cgi-bin/SMD/source/sourceResult?choice=Gene&option=Name&criteria=ARRB1) | 1.51 | 0.0036 |
| A_23_P100355 | [PPP4C](http://genome-www4.stanford.edu/cgi-bin/SMD/source/sourceResult?choice=Gene&option=Name&criteria=PPP4C) | 1.55 | 0.0036 |
| A_23_P205959 | [ALDH1A3](http://genome-www4.stanford.edu/cgi-bin/SMD/source/sourceResult?choice=Gene&option=Name&criteria=ALDH1A3) | 1.88 | 0.0036 |
| A_32_P128258 | [SIGLECP3](http://genome-www4.stanford.edu/cgi-bin/SMD/source/sourceResult?choice=Gene&option=Name&criteria=SIGLECP3) | 1.61 | 0.0036 |
| A_32_P856518 | [C20orf74](http://genome-www4.stanford.edu/cgi-bin/SMD/source/sourceResult?choice=Gene&option=Name&criteria=C20orf74) | 2.03 | 0.0036 |
| A_24_P307126 | [LOC442013](http://genome-www4.stanford.edu/cgi-bin/SMD/source/sourceResult?choice=Gene&option=Name&criteria=LOC442013) | 1.57 | 0.0036 |
| A_24_P169843 | [A_24_P169843](http://genome-www4.stanford.edu/cgi-bin/SMD/source/sourceResult?choice=Gene&option=Name&criteria=A_24_P169843) | 1.51 | 0.0036 |
| A_24_P403168 | [PRPF4B](http://genome-www4.stanford.edu/cgi-bin/SMD/source/sourceResult?choice=Gene&option=Name&criteria=PRPF4B) | 1.57 | 0.0036 |
| A_24_P358054 | [CTAGE3](http://genome-www4.stanford.edu/cgi-bin/SMD/source/sourceResult?choice=Gene&option=Name&criteria=CTAGE3) | 1.58 | 0.0036 |
| A_23_P212968 | [UGT2B11](http://genome-www4.stanford.edu/cgi-bin/SMD/source/sourceResult?choice=Gene&option=Name&criteria=UGT2B11) | 1.83 | 0.0036 |
| A_23_P133120 | [TMEM144](http://genome-www4.stanford.edu/cgi-bin/SMD/source/sourceResult?choice=Gene&option=Name&criteria=TMEM144) | 1.62 | 0.0036 |
| A_32_P101264 | [ENST00000371408](http://genome-www4.stanford.edu/cgi-bin/SMD/source/sourceResult?choice=Gene&option=Name&criteria=ENST00000371408) | 1.59 | 0.0036 |
[truncated: 213,967 more chars]
