# Supplementary material for: Host Genes Related to Paneth Cells and Xenobiotic Metabolism Are Associated with Shifts in Human Ileum-Associated Microbial Composition
Source: PLoS One. 2012 Jun 13;7(6):e30044. doi: 10.1371/journal.pone.0030044 (PMC3374611; doi:10.1371/journal.pone.0030044)
Supplement: Table S2 — Gene-probes in the 43 clusters. (DOCX) [file pone.0030044.s002.docx]

**Supplementary Table S2. Gene-probes in the 43 clusters**

| **Agilent_ID** | **Gene Name or Accession Number** | **Cluster** |
| --- | --- | --- |
| A_23_P100022 | SV2B | 1 |
| A_23_P101407 | C3 | 1 |
| A_23_P102000 | CXCR4 | 1 |
| A_23_P10232 | BANK1 | 1 |
| A_23_P103522 | SELL | 1 |
| A_23_P10356 | ADAM28 | 1 |
| A_23_P111402 | RSPO3 | 1 |
| A_23_P112470 | CCL21 | 1 |
| A_23_P113572 | CD19 | 1 |
| A_23_P113793 | ZBED2 | 1 |
| A_23_P115192 | FCRL4 | 1 |
| A_23_P115200 | FCRL4 | 1 |
| A_23_P115201 | FCRL4 | 1 |
| A_23_P116371 | MS4A1 | 1 |
| A_23_P12082 | CHI3L2 | 1 |
| A_23_P121695 | CXCL13 | 1 |
| A_23_P122127 | FYB | 1 |
| A_23_P123853 | CCL19 | 1 |
| A_23_P124335 | | 1 |
| A_23_P124542 | CR2 | 1 |
| A_23_P131024 | ZBTB32 | 1 |
| A_23_P132378 | CELSR1 | 1 |
| A_23_P132956 | UCHL1 | 1 |
| A_23_P13548 | CHRDL2 | 1 |
| A_23_P136753 | | 1 |
| A_23_P143047 | ATP6V1E2 | 1 |
| A_23_P145657 | STAG3 | 1 |
| A_23_P147578 | | 1 |
| A_23_P149368 | FCRL1 | 1 |
| A_23_P152926 | GP1BA | 1 |
| A_23_P156218 | GZMK | 1 |
| A_23_P15876 | ALPK2 | 1 |
| A_23_P159316 | BFSP2 | 1 |
| A_23_P160751 | FCRL2 | 1 |
| A_23_P166087 | RASSF2 | 1 |
| A_23_P166371 | VPREB3 | 1 |
| A_23_P166848 | LTF | 1 |
| A_23_P168288 | IL22RA2 | 1 |
| A_23_P170233 | CSTA | 1 |
| A_23_P170679 | COL4A3 | 1 |
| A_23_P17134 | MAL | 1 |
| A_23_P201211 | FCRL5 | 1 |
| A_23_P201510 | FCAMR | 1 |
| A_23_P201731 | TRAF5 | 1 |
| A_23_P204689 | CLEC2D | 1 |
| A_23_P205355 | SERPINA5 | 1 |
| A_23_P206585 | PRKCB | 1 |
| A_23_P207201 | CD79B | 1 |
| A_23_P209055 | CD22 | 1 |
| A_23_P212500 | TF | 1 |
| A_23_P214208 | CNR1 | 1 |
| A_23_P217187 | | 1 |
| A_23_P23639 | MCOLN2 | 1 |
| A_23_P250245 | CD72 | 1 |
| A_23_P251881 | NCR3 | 1 |
| A_23_P253791 | CAMP | 1 |
| A_23_P25587 | LECT1 | 1 |
| A_23_P258088 | PACSIN1 | 1 |
| A_23_P26325 | CCL17 | 1 |
| A_23_P29773 | LAMP3 | 1 |
| A_23_P300150 | NFATC1 | 1 |
| A_23_P30634 | BACH2 | 1 |
| A_23_P310931 | CNR2 | 1 |
| A_23_P312920 | POU2AF1 | 1 |
| A_23_P31725 | BLK | 1 |
| A_23_P317591 | SEMA3A | 1 |
| A_23_P321984 | CLECL1 | 1 |
| A_23_P323930 | TSPAN5 | 1 |
| A_23_P333640 | PAPLN | 1 |
| A_23_P334414 | TRAF3IP3 | 1 |
| A_23_P343398 | CCR7 | 1 |
| A_23_P345799 | FAM129C | 1 |
| A_23_P351286 | CD22 | 1 |
| A_23_P352266 | BCL2 | 1 |
| A_23_P354705 | ST8SIA1 | 1 |
| A_23_P357717 | TCL1A | 1 |
| A_23_P358438 | FCRL3 | 1 |
| A_23_P362694 | C4ORF7 | 1 |
| A_23_P36641 | AICDA | 1 |
| A_23_P366453 | KHDRBS2 | 1 |
| A_23_P36888 | FAM113B | 1 |
| A_23_P370830 | KLHL14 | 1 |
| A_23_P371076 | KLF12 | 1 |
| A_23_P372478 | SERPINA9 | 1 |
| A_23_P384965 | | 1 |
| A_23_P39067 | SPIB | 1 |
| A_23_P391344 | RASGEF1A | 1 |
| A_23_P40174 | MMP9 | 1 |
| A_23_P41114 | CSTA | 1 |
| A_23_P432947 | GREM1 | 1 |
| A_23_P433785 | P2RX5 | 1 |
| A_23_P434398 | TXLNB | 1 |
| A_23_P45248 | SH2D1A | 1 |
| A_23_P4551 | SETBP1 | 1 |
| A_23_P46039 | FCRLA | 1 |
| A_23_P4714 | MIA | 1 |
| A_23_P51767 | CD1C | 1 |
| A_23_P56578 | VIT | 1 |
| A_23_P57709 | PCOLCE2 | 1 |
| A_23_P65000 | C12ORF24 | 1 |
| A_23_P66137 | SOX8 | 1 |
| A_23_P6822 | ITIH3 | 1 |
| A_23_P70670 | CD83 | 1 |
| A_23_P7185 | STAP1 | 1 |
| A_23_P7262 | MARCH1 | 1 |
| A_23_P74145 | CD48 | 1 |
| A_23_P7503 | TIMD4 | 1 |
| A_23_P75915 | RIC3 | 1 |
| A_23_P76078 | IL23A | 1 |
| A_23_P7932 | TREML2 | 1 |
| A_23_P80528 | TIGIT | 1 |
| A_23_P80883 | BTLA | 1 |
| A_23_P85269 | TTN | 1 |
| A_23_P85800 | CD52 | 1 |
| A_23_P90419 | PBX4 | 1 |
| A_23_P98910 | LRMP | 1 |
| A_23_P99386 | TNFSF11 | 1 |
| A_24_P106542 | RSPO3 | 1 |
| A_24_P133905 | CCL23 | 1 |
| A_24_P143492 | BCAS4 | 1 |
| A_24_P174353 | NPPC | 1 |
| A_24_P182947 | GCET2 | 1 |
| A_24_P184803 | COCH | 1 |
| A_24_P20630 | LEF1 | 1 |
| A_24_P227927 | IL21R | 1 |
| A_24_P252945 | CXCR5 | 1 |
| A_24_P254106 | CD22 | 1 |
| A_24_P256073 | | 1 |
| A_24_P272451 | C17ORF87 | 1 |
| A_24_P276576 | FCRLA | 1 |
| A_24_P295010 | SERPINB9 | 1 |
| A_24_P29733 | CDK14 | 1 |
| A_24_P314477 | TUBB2B | 1 |
| A_24_P319647 | FCRL2 | 1 |
| A_24_P324838 | IGHD | 1 |
| A_24_P328504 | SP140 | 1 |
| A_24_P348326 | IL27RA | 1 |
| A_24_P365975 | COL8A2 | 1 |
| A_24_P37020 | | 1 |
| A_24_P388528 | ST6GAL1 | 1 |
| A_24_P395415 | | 1 |
| A_24_P418816 | GPX7 | 1 |
| A_24_P465879 | | 1 |
| A_24_P50738 | | 1 |
| A_24_P595223 | MIAT | 1 |
| A_24_P621701 | | 1 |
| A_24_P67980 | LOC100129447 | 1 |
| A_24_P73599 | IL16 | 1 |
| A_24_P822869 | | 1 |
| A_24_P827096 | | 1 |
| A_24_P916853 | | 1 |
| A_24_P930963 | | 1 |
| A_24_P937546 | CMTM7 | 1 |
| A_24_P940079 | | 1 |
| A_24_P940348 | FAM129C | 1 |
| A_24_P941359 | FAM65B | 1 |
| A_24_P95723 | KIAA0125 | 1 |
| A_24_P98210 | TFEC | 1 |
| A_32_P107002 | RUNDC2A | 1 |
| A_32_P107029 | NAPSA | 1 |
| A_32_P108156 | MIR155HG | 1 |
| A_32_P111394 | | 1 |
| A_32_P111492 | | 1 |
| A_32_P113472 | | 1 |
| A_32_P125402 | | 1 |
| A_32_P125589 | | 1 |
| A_32_P137604 | ANKRD33B | 1 |
| A_32_P139260 | | 1 |
| A_32_P153361 | | 1 |
| A_32_P162862 | | 1 |
| A_32_P17343 | | 1 |
| A_32_P177725 | | 1 |
| A_32_P179998 | DMRTC1 | 1 |
| A_32_P184039 | | 1 |
| A_32_P197561 | EBF1 | 1 |
| A_32_P202502 | EFHA2 | 1 |
| A_32_P206479 | ZNF831 | 1 |
| A_32_P219520 | TNFAIP8 | 1 |
| A_32_P228886 | | 1 |
| A_32_P30905 | WDFY4 | 1 |
| A_32_P34703 | | 1 |
| A_32_P39944 | | 1 |
| A_32_P41267 | | 1 |
| A_32_P42149 | | 1 |
| A_32_P44394 | AIM2 | 1 |
| A_32_P453321 | C1ORF228 | 1 |
| A_32_P46510 | ZC3H6 | 1 |
| A_32_P48054 | CNR2 | 1 |
| A_32_P49728 | LOC339803 | 1 |
| A_32_P62371 | | 1 |
| A_32_P64016 | RUNDC2C | 1 |
| A_32_P703 | LOC646626 | 1 |
| A_32_P71858 | | 1 |
| A_32_P71876 | | 1 |
| A_32_P72067 | ARHGAP24 | 1 |
| A_32_P75399 | | 1 |
| A_32_P8813 | LOC283663 | 1 |
| A_32_P92445 | LSAMP | 1 |
| A_32_P94176 | | 1 |
| A_23_P10025 | NELL2 | 2 |
| A_23_P10182 | ACOX2 | 2 |
| A_23_P103486 | CYP2J2 | 2 |
| A_23_P106773 | SULT1A2 | 2 |
| A_23_P118894 | PRR15L | 2 |
| A_23_P11968 | GUCA2A | 2 |
| A_23_P120902 | LGALS2 | 2 |
| A_23_P140207 | PCK2 | 2 |
| A_23_P145644 | DDC | 2 |
| A_23_P148345 | RNF128 | 2 |
| A_23_P149613 | FMO1 | 2 |
| A_23_P152087 | FAM82A2 | 2 |
| A_23_P152505 | ABAT | 2 |
| A_23_P15402 | SAT2 | 2 |
| A_23_P154379 | NAT8 | 2 |
| A_23_P156076 | AGXT2 | 2 |
| A_23_P158297 | BTNL3 | 2 |
| A_23_P162288 | MYO1A | 2 |
| A_23_P163380 | MTHFS | 2 |
| A_23_P163567 | SMPD3 | 2 |
| A_23_P166306 | CBS | 2 |
| A_23_P167182 | LRAT | 2 |
| A_23_P16866 | VIL1 | 2 |
| A_23_P17438 | EDN3 | 2 |
| A_23_P18713 | ABCG2 | 2 |
| A_23_P200685 | MOSC2 | 2 |
| A_23_P207371 | G6PC | 2 |
| A_23_P20752 | CDK20 | 2 |
| A_23_P209116 | CYP4F3 | 2 |
| A_23_P209527 | VIL1 | 2 |
| A_23_P211850 | ABHD6 | 2 |
| A_23_P21644 | HSD17B11 | 2 |
| A_23_P216712 | TRPM6 | 2 |
| A_23_P217009 | C9ORF24 | 2 |
| A_23_P252236 | KLKB1 | 2 |
| A_23_P252432 | TM4SF4 | 2 |
| A_23_P254512 | EFNA1 | 2 |
| A_23_P29046 | CBR1 | 2 |
| A_23_P304110 | ANKRD43 | 2 |
| A_23_P308519 | SLC9A3R1 | 2 |
| A_23_P314101 | SUSD2 | 2 |
| A_23_P325562 | SLC1A7 | 2 |
| A_23_P328740 | LINCR | 2 |
| A_23_P349463 | CHP2 | 2 |
| A_23_P357270 | SLC17A4 | 2 |
| A_23_P397376 | MAF | 2 |
| A_23_P407695 | FAM151A | 2 |
| A_23_P409945 | OAZ1 | 2 |
| A_23_P421011 | KAZALD1 | 2 |
| A_23_P42265 | APOM | 2 |
| A_23_P43425 | C9ORF40 | 2 |
| A_23_P60006 | ANXA13 | 2 |
| A_23_P63032 | GUCA2B | 2 |
| A_23_P69908 | GLRX | 2 |
| A_23_P70813 | C6ORF123 | 2 |
| A_23_P8834 | EPHX2 | 2 |
| A_24_P118247 | CTU2 | 2 |
| A_24_P13790 | CES2 | 2 |
| A_24_P173823 | PBX1 | 2 |
| A_24_P192727 | KAZALD1 | 2 |
| A_24_P23546 | SP8 | 2 |
| A_24_P260325 | LRAT | 2 |
| A_24_P262201 | SULT1A4 | 2 |
| A_24_P26792 | TRPM6 | 2 |
| A_24_P270769 | VPS35 | 2 |
| A_24_P308506 | NAT8B | 2 |
| A_24_P318544 | CSNK1D | 2 |
| A_24_P374634 | STAU2 | 2 |
| A_24_P385313 | PTPRF | 2 |
| A_24_P405850 | PGRMC2 | 2 |
| A_24_P49657 | | 2 |
| A_24_P602507 | AGPHD1 | 2 |
| A_24_P63468 | AGXT2 | 2 |
| A_24_P725998 | | 2 |
| A_24_P89426 | APOM | 2 |
| A_32_P104334 | | 2 |
| A_32_P110472 | IYD | 2 |
| A_32_P113066 | LRAT | 2 |
| A_32_P139391 | | 2 |
| A_32_P2883 | | 2 |
| A_32_P313405 | LAMA1 | 2 |
| A_32_P52911 | ADI1 | 2 |
| A_32_P69333 | | 2 |
| A_32_P856518 | | 2 |
| A_23_P100341 | ORC6L | 3 |
| A_23_P108103 | | 3 |
| A_23_P119583 | PDE4C | 3 |
| A_23_P123018 | | 3 |
| A_23_P137786 | ADAMTSL4 | 3 |
| A_23_P13929 | NRIP2 | 3 |
| A_23_P14649 | C15ORF28 | 3 |
| A_23_P15621 | PRAC | 3 |
| A_23_P18055 | C3ORF51 | 3 |
| A_23_P203391 | ASRGL1 | 3 |
| A_23_P206110 | CYP1A2 | 3 |
| A_23_P209459 | | 3 |
| A_23_P211603 | | 3 |
| A_23_P251232 | TTTY14 | 3 |
| A_23_P309545 | MDM2 | 3 |
| A_23_P370574 | KIR3DL3 | 3 |
| A_23_P395954 | SSH2 | 3 |
| A_23_P410736 | GPR135 | 3 |
| A_23_P47220 | | 3 |
| A_23_P68539 | MAVS | 3 |
| A_23_P78975 | | 3 |
| A_23_P93709 | | 3 |
| A_24_P109417 | C1ORF187 | 3 |
| A_24_P111271 | PLA2G2D | 3 |
| A_24_P129588 | GPR120 | 3 |
| A_24_P152937 | TNRC18 | 3 |
| A_24_P238046 | LOC399491 | 3 |
| A_24_P251040 | ARTN | 3 |
| A_24_P251866 | ADAMTS2 | 3 |
| A_24_P323545 | MYH14 | 3 |
| A_24_P33785 | | 3 |
| A_24_P403459 | IFNA4 | 3 |
| A_24_P503731 | | 3 |
| A_24_P612200 | | 3 |
| A_24_P647507 | | 3 |
| A_24_P693448 | ZNF552 | 3 |
| A_24_P740022 | | 3 |
| A_24_P765053 | | 3 |
| A_24_P83075 | C21ORF86 | 3 |
| A_24_P843921 | | 3 |
| A_24_P936282 | | 3 |
| A_32_P107441 | | 3 |
| A_32_P13442 | | 3 |
| A_32_P139909 | | 3 |
| A_32_P22014 | | 3 |
| A_32_P22989 | | 3 |
| A_32_P235185 | | 3 |
| A_32_P45894 | STAG3L1 | 3 |
| A_32_P50603 | C2ORF70 | 3 |
| A_32_P85230 | | 3 |
| A_32_P88555 | | 3 |
| A_23_P100355 | PPP4C | 4 |
| A_23_P102551 | MALL | 4 |
| A_23_P104188 | ELF3 | 4 |
| A_23_P112548 | HDHD3 | 4 |
| A_23_P121051 | PCCB | 4 |
| A_23_P122375 | ZFAND3 | 4 |
| A_23_P123265 | SUMF2 | 4 |
| A_23_P129209 | IDH2 | 4 |
| A_23_P129312 | PPP1R14D | 4 |
| A_23_P130619 | STXBP2 | 4 |
| A_23_P138835 | CAPN1 | 4 |
| A_23_P147109 | C9ORF167 | 4 |
| A_23_P149517 | PIGR | 4 |
| A_23_P152620 | TNFSF13 | 4 |
| A_23_P155624 | AP2M1 | 4 |
| A_23_P162739 | TSC22D1 | 4 |
| A_23_P165280 | POLR2E | 4 |
| A_23_P201086 | ARF1 | 4 |
| A_23_P202837 | CCND1 | 4 |
| A_23_P206474 | TRAF7 | 4 |
| A_23_P214554 | TRIM15 | 4 |
| A_23_P218247 | CES3 | 4 |
| A_23_P28238 | SNX17 | 4 |
| A_23_P28878 | C20ORF27 | 4 |
| A_23_P31921 | ASS1 | 4 |
| A_23_P35820 | CFL1 | 4 |
| A_23_P35883 | FOXRED1 | 4 |
| A_23_P36700 | TAPBPL | 4 |
| A_23_P373708 | FLJ40504 | 4 |
| A_23_P37910 | MAPK3 | 4 |
| A_23_P434710 | PPP1CA | 4 |
| A_23_P47818 | CS | 4 |
| A_23_P54963 | MRPL38 | 4 |
| A_23_P55990 | NAPA | 4 |
| A_23_P64938 | LPCAT3 | 4 |
| A_23_P6596 | HES1 | 4 |
| A_23_P72068 | GMDS | 4 |
| A_23_P93349 | SLC44A4 | 4 |
| A_23_P99320 | KRT18 | 4 |
| A_24_P118341 | | 4 |
| A_24_P15973 | | 4 |
| A_24_P161733 | | 4 |
| A_24_P161809 | | 4 |
| A_24_P161827 | | 4 |
| A_24_P169843 | | 4 |
| A_24_P178834 | | 4 |
| A_24_P195164 | | 4 |
| A_24_P195974 | | 4 |
| A_24_P199774 | TINAGL1 | 4 |
| A_24_P213336 | | 4 |
| A_24_P233850 | SDHC | 4 |
| A_24_P244800 | NDRG2 | 4 |
| A_24_P24645 | | 4 |
| A_24_P247074 | | 4 |
| A_24_P281374 | | 4 |
| A_24_P281605 | | 4 |
| A_24_P283000 | DVL3 | 4 |
| A_24_P295601 | KIAA0652 | 4 |
| A_24_P298894 | | 4 |
| A_24_P302332 | NECAP2 | 4 |
| A_24_P303770 | CTSB | 4 |
| A_24_P304449 | MLEC | 4 |
| A_24_P309317 | PSAP | 4 |
| A_24_P332595 | | 4 |
| A_24_P341546 | | 4 |
| A_24_P347854 | SYNGR2 | 4 |
| A_24_P349590 | | 4 |
| A_24_P357836 | | 4 |
| A_24_P358474 | | 4 |
| A_24_P383660 | | 4 |
| A_24_P399622 | COPE | 4 |
| A_24_P409420 | | 4 |
| A_24_P418687 | | 4 |
| A_24_P451992 | | 4 |
| A_24_P54485 | CCDC115 | 4 |
| A_24_P578445 | | 4 |
| A_24_P633686 | | 4 |
| A_24_P67395 | KRT8 | 4 |
| A_24_P6850 | | 4 |
| A_24_P686014 | | 4 |
| A_24_P68649 | RNPEP | 4 |
| A_24_P700170 | | 4 |
| A_24_P745352 | | 4 |
| A_24_P75708 | | 4 |
| A_24_P7750 | | 4 |
| A_24_P792748 | | 4 |
| A_24_P843309 | | 4 |
| A_24_P890995 | | 4 |
| A_24_P89887 | C9ORF3 | 4 |
| A_24_P93321 | | 4 |
| A_32_P148710 | CFL1 | 4 |
| A_32_P41254 | MRAP2 | 4 |
| A_32_P532216 | QARS | 4 |
| A_23_P100539 | ABCC6 | 5 |
| A_23_P100754 | SMURF2 | 5 |
| A_23_P102364 | NGEF | 5 |
| A_23_P104819 | TREH | 5 |
| A_23_P105803 | FGF9 | 5 |
| A_23_P106024 | JAG2 | 5 |
| A_23_P107981 | SULT2B1 | 5 |
| A_23_P108082 | CREB3L3 | 5 |
| A_23_P112162 | DGAT1 | 5 |
| A_23_P112481 | AQP3 | 5 |
| A_23_P112482 | AQP3 | 5 |
| A_23_P113682 | SLC34A3 | 5 |
| A_23_P118334 | PAPD5 | 5 |
| A_23_P120594 | ACSS1 | 5 |
| A_23_P130158 | WNT3 | 5 |
| A_23_P133338 | CDHR2 | 5 |
| A_23_P1352 | SFRP5 | 5 |
| A_23_P143734 | CYP2D6 | 5 |
| A_23_P145786 | MLXIPL | 5 |
| A_23_P146798 | SEPHS2 | 5 |
| A_23_P148249 | THSD4 | 5 |
| A_23_P15146 | IL32 | 5 |
| A_23_P152047 | SCAMP5 | 5 |
| A_23_P152262 | DPEP1 | 5 |
| A_23_P155123 | CYP2D6 | 5 |
| A_23_P155351 | BTD | 5 |
| A_23_P160433 | C1ORF115 | 5 |
| A_23_P162660 | GPD1 | 5 |
| A_23_P163390 | CHRNA7 | 5 |
| A_23_P17811 | SEC14L2 | 5 |
| A_23_P19894 | AQP1 | 5 |
| A_23_P202275 | PRAP1 | 5 |
| A_23_P202683 | CDHR5 | 5 |
| A_23_P203191 | APOA1 | 5 |
| A_23_P20427 | RHOBTB2 | 5 |
| A_23_P20713 | C8G | 5 |
| A_23_P252163 | DAPK1 | 5 |
| A_23_P29621 | GLYCTK | 5 |
| A_23_P302914 | ZFYVE28 | 5 |
| A_23_P31041 | MYLIP | 5 |
| A_23_P312358 | BEND7 | 5 |
| A_23_P323943 | SLC5A12 | 5 |
| A_23_P324107 | RORC | 5 |
| A_23_P354609 | | 5 |
| A_23_P35970 | SLC37A4 | 5 |
| A_23_P360340 | UACA | 5 |
| A_23_P36129 | DAK | 5 |
| A_23_P372834 | AQP1 | 5 |
| A_23_P377141 | VPS13A | 5 |
| A_23_P379034 | BAIAP2L2 | 5 |
| A_23_P38235 | ACE | 5 |
| A_23_P392544 | CTAGE3 | 5 |
| A_23_P3963 | CDR2L | 5 |
| A_23_P402313 | FLJ35424 | 5 |
| A_23_P402952 | LOC441294 | 5 |
| A_23_P40611 | TCN2 | 5 |
| A_23_P413721 | GPD1 | 5 |
| A_23_P430839 | HAPLN4 | 5 |
| A_23_P430842 | HAPLN4 | 5 |
| A_23_P43415 | HSD17B3 | 5 |
| A_23_P43484 | CDKN2A | 5 |
| A_23_P44663 | SERPINA2 | 5 |
| A_23_P47102 | ACY3 | 5 |
| A_23_P48455 | AMN | 5 |
| A_23_P52668 | NAALADL1 | 5 |
| A_23_P56356 | PLB1 | 5 |
| A_23_P57868 | ACY1 | 5 |
| A_23_P5845 | KHK | 5 |
| A_23_P77328 | GCHFR | 5 |
| A_23_P88626 | ANPEP | 5 |
| A_23_P9472 | VPS13A | 5 |
| A_24_P10226 | SEMA6D | 5 |
| A_24_P169507 | MUC17 | 5 |
| A_24_P200831 | MLXIPL | 5 |
| A_24_P247643 | MUC17 | 5 |
| A_24_P252934 | APOA4 | 5 |
| A_24_P256219 | MAF | 5 |
| A_24_P290585 | UACA | 5 |
| A_24_P305223 | CTAGE1 | 5 |
| A_24_P311845 | PANK3 | 5 |
| A_24_P312519 | PBLD | 5 |
| A_24_P325533 | PKLR | 5 |
| A_24_P333326 | CTAGE5 | 5 |
| A_24_P340112 | | 5 |
| A_24_P34865 | BTNL3 | 5 |
| A_24_P358054 | | 5 |
| A_24_P362904 | PFKFB4 | 5 |
| A_24_P379616 | PLB1 | 5 |
| A_24_P48856 | CBS | 5 |
| A_24_P51485 | CSDE1 | 5 |
| A_24_P57207 | CREB3L3 | 5 |
| A_24_P681011 | HIPK2 | 5 |
| A_24_P695306 | TMEM229A | 5 |
| A_24_P740620 | LOC197350 | 5 |
| A_24_P750817 | LOC147710 | 5 |
| A_24_P795662 | | 5 |
| A_24_P8165 | SLC15A1 | 5 |
| A_24_P867111 | LOC283177 | 5 |
| A_24_P913828 | MUC3A | 5 |
| A_24_P915095 | GRAMD1C | 5 |
| A_24_P934704 | | 5 |
| A_24_P935147 | | 5 |
| A_32_P125832 | LOC100128893 | 5 |
| A_32_P16258 | EXOC6B | 5 |
| A_32_P208654 | PIWIL2 | 5 |
| A_32_P211363 | AGAP8 | 5 |
| A_32_P31618 | GSR | 5 |
| A_32_P491499 | | 5 |
| A_32_P78295 | ATOH7 | 5 |
| A_32_P888644 | | 5 |
| A_23_P10077 | PNPLA2 | 6 |
| A_23_P103968 | AKR7A3 | 6 |
| A_23_P107211 | RAB5C | 6 |
| A_23_P107412 | P4HB | 6 |
| A_23_P108280 | CYP4F12 | 6 |
| A_23_P108294 | PPAP2C | 6 |
| A_23_P108326 | NR2F6 | 6 |
| A_23_P112103 | GSDMD | 6 |
| A_23_P117424 | DCAF11 | 6 |
| A_23_P119377 | CYTH2 | 6 |
| A_23_P12405 | ESPN | 6 |
| A_23_P128817 | PCK2 | 6 |
| A_23_P131060 | CYP4F8 | 6 |
| A_23_P131202 | HES6 | 6 |
| A_23_P131299 | ZFAND2B | 6 |
| A_23_P131308 | CYP27A1 | 6 |
| A_23_P131899 | SDCBP2 | 6 |
| A_23_P133799 | KLC4 | 6 |
| A_23_P135454 | AFG3L2 | 6 |
| A_23_P138776 | ACP2 | 6 |
| A_23_P138782 | ACP2 | 6 |
| A_23_P140830 | ELMO3 | 6 |
| A_23_P141126 | GALK1 | 6 |
| A_23_P142403 | TM6SF2 | 6 |
| A_23_P143218 | ACOT8 | 6 |
| A_23_P146631 | ZER1 | 6 |
| A_23_P146922 | GAS6 | 6 |
| A_23_P147326 | SERINC2 | 6 |
| A_23_P14853 | LTK | 6 |
| A_23_P14939 | TXNDC11 | 6 |
| A_23_P1519 | PITPNM1 | 6 |
| A_23_P15218 | ZNF768 | 6 |
| A_23_P152245 | CDK10 | 6 |
| A_23_P154245 | GPR35 | 6 |
| A_23_P154801 | TRPC4AP | 6 |
| A_23_P160154 | GALE | 6 |
| A_23_P160582 | HYI | 6 |
| A_23_P161125 | MOV10 | 6 |
| A_23_P162982 | DHRS4 | 6 |
| A_23_P168864 | ZNF16 | 6 |
| A_23_P201483 | MAPKAPK2 | 6 |
| A_23_P202510 | ASB13 | 6 |
| A_23_P207905 | SECTM1 | 6 |
| A_23_P209944 | RETSAT | 6 |
| A_23_P210623 | PCK1 | 6 |
| A_23_P211267 | RIPK4 | 6 |
| A_23_P216739 | SURF6 | 6 |
| A_23_P216996 | SH3GLB2 | 6 |
| A_23_P217917 | GSTM4 | 6 |
| A_23_P250035 | SDHA | 6 |
| A_23_P252671 | CLTB | 6 |
| A_23_P252808 | WBP1 | 6 |
| A_23_P258982 | PKN3 | 6 |
| A_23_P27005 | DHRS11 | 6 |
| A_23_P28090 | STX10 | 6 |
| A_23_P29851 | LRPAP1 | 6 |
| A_23_P2990 | CEBPE | 6 |
| A_23_P307525 | ANKRD9 | 6 |
| A_23_P30848 | HLA-E | 6 |
| A_23_P310590 | OAF | 6 |
| A_23_P316812 | | 6 |
| A_23_P320553 | PPFIA3 | 6 |
| A_23_P321388 | RNF19B | 6 |
| A_23_P323836 | C9ORF75 | 6 |
| A_23_P327370 | PGPEP1 | 6 |
| A_23_P337141 | GLI4 | 6 |
| A_23_P341065 | RP3-402G11.5 | 6 |
| A_23_P343719 | PLCB3 | 6 |
| A_23_P347432 | DVL1 | 6 |
| A_23_P366682 | C19ORF20 | 6 |
| A_23_P366828 | ABCC6 | 6 |
| A_23_P379649 | BMF | 6 |
| A_23_P386356 | KIF12 | 6 |
| A_23_P391980 | CMTM4 | 6 |
| A_23_P394947 | CLDN2 | 6 |
| A_23_P396062 | RAB40C | 6 |
| A_23_P397910 | CBLC | 6 |
| A_23_P406424 | RHOC | 6 |
| A_23_P407142 | LUZP1 | 6 |
| A_23_P41765 | IRF1 | 6 |
| A_23_P42353 | ETV7 | 6 |
| A_23_P426809 | ARHGEF11 | 6 |
| A_23_P431346 | PRR15 | 6 |
| A_23_P43238 | NAPRT1 | 6 |
| A_23_P43296 | FBXL6 | 6 |
| A_23_P434212 | SULT1A1 | 6 |
| A_23_P434421 | CCNY | 6 |
| A_23_P43763 | PLLP | 6 |
| A_23_P48747 | DHRS1 | 6 |
| A_23_P49192 | CES2 | 6 |
| A_23_P49351 | AMDHD2 | 6 |
| A_23_P500886 | CLDN15 | 6 |
| A_23_P501547 | ADCY6 | 6 |
| A_23_P50399 | DCAF15 | 6 |
| A_23_P50710 | CYP4F2 | 6 |
| A_23_P52425 | NKX2-3 | 6 |
| A_23_P5610 | DOK1 | 6 |
| A_23_P5778 | RAB17 | 6 |
| A_23_P58993 | MOCS1 | 6 |
| A_23_P65022 | ACADS | 6 |
| A_23_P66454 | GSDMB | 6 |
| A_23_P67339 | RCN3 | 6 |
| A_23_P70539 | HLA-C | 6 |
| A_23_P72025 | SLC25A20 | 6 |
| A_23_P89113 | NAT15 | 6 |
| A_23_P90014 | GPR108 | 6 |
| A_23_P94365 | ADCK5 | 6 |
| A_23_P94689 | URM1 | 6 |
| A_23_P94795 | TEAD4 | 6 |
| A_23_P95330 | ZDHHC9 | 6 |
| A_23_P98876 | SLC39A5 | 6 |
| A_23_P99974 | CLK3 | 6 |
| A_24_P101771 | | 6 |
| A_24_P103952 | DGKZ | 6 |
| A_24_P110012 | HLA-L | 6 |
| A_24_P115932 | GPR44 | 6 |
| A_24_P127159 | | 6 |
| A_24_P148521 | TMBIM1 | 6 |
| A_24_P151084 | CNDP2 | 6 |
| A_24_P151582 | TEF | 6 |
| A_24_P161933 | HLA-B | 6 |
| A_24_P164731 | TMED1 | 6 |
| A_24_P185117 | RILP | 6 |
| A_24_P186379 | C10ORF125 | 6 |
| A_24_P198844 | MPDU1 | 6 |
| A_24_P205589 | ACOT7 | 6 |
| A_24_P219769 | ATP5SL | 6 |
| A_24_P221974 | | 6 |
| A_24_P22488 | AXIN1 | 6 |
| A_24_P229766 | | 6 |
| A_24_P231494 | DNPEP | 6 |
| A_24_P257971 | SLC27A4 | 6 |
| A_24_P263767 | | 6 |
| A_24_P272761 | DENND1A | 6 |
| A_24_P276490 | LYPLA2 | 6 |
| A_24_P278637 | FADD | 6 |
| A_24_P287974 | CUEDC1 | 6 |
| A_24_P288890 | FAM101A | 6 |
| A_24_P29260 | MGAT4B | 6 |
| A_24_P294419 | SEMA3B | 6 |
| A_24_P300952 | APLP2 | 6 |
| A_24_P307626 | ATP1A4 | 6 |
| A_24_P315873 | SDHAL1 | 6 |
| A_24_P32646 | | 6 |
| A_24_P32715 | | 6 |
| A_24_P367289 | DDR1 | 6 |
| A_24_P36890 | RAP1GAP | 6 |
| A_24_P372901 | MVK | 6 |
| A_24_P376483 | HLA-A | 6 |
| A_24_P379858 | ACAA1 | 6 |
| A_24_P382661 | ETS2 | 6 |
| A_24_P386622 | ARRB1 | 6 |
| A_24_P392151 | C11ORF86 | 6 |
| A_24_P392925 | GLTPD2 | 6 |
| A_24_P396660 | GSTM4 | 6 |
| A_24_P396662 | GSTM4 | 6 |
| A_24_P398432 | ARAP1 | 6 |
| A_24_P401381 | | 6 |
| A_24_P404245 | PCYT2 | 6 |
| A_24_P404840 | GJB1 | 6 |
| A_24_P48204 | SECTM1 | 6 |
| A_24_P49183 | EXD3 | 6 |
| A_24_P538478 | MED28 | 6 |
| A_24_P59062 | GGTLC2 | 6 |
| A_24_P595369 | LOC100288570 | 6 |
| A_24_P64653 | METTL7B | 6 |
| A_24_P65199 | CDK10 | 6 |
| A_24_P68814 | DQX1 | 6 |
| A_24_P698136 | | 6 |
| A_24_P698141 | | 6 |
| A_24_P771278 | | 6 |
| A_24_P79755 | AKR1A1 | 6 |
| A_24_P83758 | | 6 |
| A_24_P936272 | | 6 |
| A_24_P938236 | | 6 |
| A_24_P9605 | PPP1R16A | 6 |
| A_24_P99216 | LRP10 | 6 |
| A_24_P99795 | ISOC2 | 6 |
| A_32_P117338 | AFG3L2 | 6 |
| A_32_P155247 | FTL | 6 |
| A_32_P183904 | SHF | 6 |
| A_32_P19135 | RAB4B | 6 |
| A_32_P234459 | | 6 |
| A_32_P26103 | AGPAT2 | 6 |
| A_32_P475513 | | 6 |
| A_32_P7015 | TSPAN15 | 6 |
| A_32_P86763 | TGM2 | 6 |
| A_23_P101141 | RNF125 | 7 |
| A_23_P101351 | ZNF426 | 7 |
| A_23_P105815 | | 7 |
| A_23_P110896 | SLC17A5 | 7 |
| A_23_P115167 | LRRC8B | 7 |
| A_23_P126457 | ISG20L2 | 7 |
| A_23_P127663 | PRRG4 | 7 |
| A_23_P129903 | TRIM16L | 7 |
| A_23_P131834 | | 7 |
| A_23_P138105 | MED18 | 7 |
| A_23_P144704 | | 7 |
| A_23_P145204 | HFE | 7 |
| A_23_P153256 | ZNF773 | 7 |
| A_23_P156185 | SHROOM1 | 7 |
| A_23_P16673 | CNN2 | 7 |
| A_23_P17706 | IL17RA | 7 |
| A_23_P20122 | ZC3HAV1 | 7 |
| A_23_P206899 | TMEM159 | 7 |
| A_23_P214066 | ARHGAP26 | 7 |
| A_23_P22499 | GNL3L | 7 |
| A_23_P253661 | DNAJC22 | 7 |
| A_23_P253738 | CLN8 | 7 |
| A_23_P34510 | PHC2 | 7 |
| A_23_P354170 | PIGX | 7 |
| A_23_P364236 | MGA | 7 |
| A_23_P369666 | ZMYND8 | 7 |
| A_23_P385529 | POLR3E | 7 |
| A_23_P388553 | ZKSCAN1 | 7 |
| A_23_P400036 | | 7 |
| A_23_P404595 | ZNF417 | 7 |
| A_23_P41824 | | 7 |
| A_23_P424712 | CCDC142 | 7 |
| A_23_P55256 | ZNF652 | 7 |
| A_23_P57007 | PRO0628 | 7 |
| A_23_P76145 | ARNTL2 | 7 |
| A_23_P89680 | | 7 |
| A_24_P102151 | SNRK | 7 |
| A_24_P114255 | MBOAT2 | 7 |
| A_24_P122636 | BPNT1 | 7 |
| A_24_P134942 | VHL | 7 |
| A_24_P136124 | LOC100131642 | 7 |
| A_24_P136155 | | 7 |
| A_24_P145035 | CBWD5 | 7 |
| A_24_P152743 | TMC6 | 7 |
| A_24_P179175 | PHC3 | 7 |
| A_24_P191884 | PHAX | 7 |
| A_24_P201973 | TEP1 | 7 |
| A_24_P273523 | | 7 |
| A_24_P282083 | | 7 |
| A_24_P297098 | PHF20 | 7 |
| A_24_P314597 | KIAA0319L | 7 |
| A_24_P316414 | LOC100289058 | 7 |
| A_24_P316454 | | 7 |
| A_24_P319369 | F11R | 7 |
| A_24_P323916 | | 7 |
| A_24_P341489 | | 7 |
| A_24_P374863 | RLTPR | 7 |
| A_24_P450372 | | 7 |
| A_24_P453740 | DNAJC21 | 7 |
| A_24_P486503 | | 7 |
| A_24_P649357 | LOC653188 | 7 |
| A_24_P655888 | | 7 |
| A_24_P683917 | FLNB | 7 |
| A_24_P71280 | GPR157 | 7 |
| A_24_P8257 | | 7 |
| A_24_P826046 | | 7 |
| A_24_P8524 | | 7 |
| A_24_P873659 | MALAT1 | 7 |
| A_24_P917951 | NOC2L | 7 |
| A_24_P919733 | ZNF721 | 7 |
| A_24_P924932 | BCL2L15 | 7 |
| A_24_P928415 | VPS29 | 7 |
| A_24_P931628 | | 7 |
| A_24_P935652 | NUB1 | 7 |
| A_24_P937435 | ELMOD3 | 7 |
| A_24_P940615 | | 7 |
| A_24_P941217 | SGPP2 | 7 |
| A_24_P941309 | ZNF224 | 7 |
| A_24_P941773 | METTL7A | 7 |
| A_24_P942703 | LOC728153 | 7 |
| A_32_P105469 | | 7 |
| A_32_P106944 | ZNF429 | 7 |
| A_32_P114615 | | 7 |
| A_32_P133526 | | 7 |
| A_32_P136477 | | 7 |
| A_32_P142827 | | 7 |
| A_32_P144599 | MACC1 | 7 |
| A_32_P147500 | | 7 |
| A_32_P157671 | | 7 |
| A_32_P177937 | GABPB2 | 7 |
| A_32_P181339 | | 7 |
| A_32_P18300 | | 7 |
| A_32_P198791 | | 7 |
| A_32_P22679 | | 7 |
| A_32_P230465 | | 7 |
| A_32_P24741 | TCAG7.907 | 7 |
| A_32_P35603 | | 7 |
| A_32_P41375 | TCAG7.907 | 7 |
| A_32_P44775 | C9ORF85 | 7 |
| A_32_P50406 | TCAG7.907 | 7 |
| A_32_P56874 | LPP | 7 |
| A_32_P61708 | | 7 |
| A_32_P70519 | LPP | 7 |
| A_32_P72553 | ITPRIPL2 | 7 |
| A_32_P90615 | | 7 |
| A_32_P930953 | | 7 |
| A_32_P95147 | | 7 |
| A_32_P97547 | | 7 |
| A_32_P98574 | | 7 |
| A_23_P10121 | SFRP1 | 8 |
| A_23_P10127 | SFRP1 | 8 |
| A_23_P103765 | FCER1A | 8 |
| A_23_P111583 | CD36 | 8 |
| A_23_P112554 | COL15A1 | 8 |
| A_23_P117662 | HDC | 8 |
| A_23_P118615 | ABCA8 | 8 |
| A_23_P119562 | CFD | 8 |
| A_23_P120103 | KCNS3 | 8 |
| A_23_P120125 | COLEC11 | 8 |
| A_23_P12746 | MRC1L1 | 8 |
| A_23_P128235 | KRT1 | 8 |
| A_23_P134347 | CPVL | 8 |
| A_23_P135548 | DPYD | 8 |
| A_23_P145841 | SOSTDC1 | 8 |
| A_23_P149562 | ARHGAP29 | 8 |
| A_23_P150457 | LYVE1 | 8 |
| A_23_P157914 | MAMDC2 | 8 |
| A_23_P15889 | CBLN2 | 8 |
| A_23_P18649 | FAT4 | 8 |
| A_23_P205959 | ALDH1A3 | 8 |
| A_23_P207456 | CCL8 | 8 |
| A_23_P210100 | CYP26B1 | 8 |
| A_23_P216429 | ASPN | 8 |
| A_23_P250607 | PLS3 | 8 |
| A_23_P26965 | CCL13 | 8 |
| A_23_P343104 | FLJ30901 | 8 |
| A_23_P360605 | KIAA0802 | 8 |
| A_23_P401606 | EDIL3 | 8 |
| A_23_P425925 | KRT222 | 8 |
| A_23_P43988 | DPYD | 8 |
| A_23_P51518 | RGS5 | 8 |
| A_23_P63447 | | 8 |
| A_23_P71855 | C5 | 8 |
| A_23_P72668 | SDPR | 8 |
| A_23_P7727 | HAPLN1 | 8 |
| A_23_P85201 | PLP1 | 8 |
| A_23_P88347 | FERMT2 | 8 |
| A_24_P125335 | CCL13 | 8 |
| A_24_P183664 | TRIL | 8 |
| A_24_P284959 | PCDH18 | 8 |
| A_24_P330263 | EDNRB | 8 |
| A_24_P385134 | SCD5 | 8 |
| A_24_P388322 | COLEC11 | 8 |
| A_24_P762613 | | 8 |
| A_24_P925505 | CD36 | 8 |
| A_24_P943781 | C7ORF58 | 8 |
| A_32_P128258 | SIGLECP3 | 8 |
| A_32_P13392 | | 8 |
| A_32_P221966 | SFTPF | 8 |
| A_32_P225816 | PRDM16 | 8 |
| A_32_P24140 | GAS2 | 8 |
| A_32_P56001 | CD93 | 8 |
| A_23_P101297 | EML2 | 9 |
| A_23_P120883 | HMOX1 | 9 |
| A_23_P122563 | PFDN6 | 9 |
| A_23_P12329 | APH1A | 9 |
| A_23_P12730 | CSTF2T | 9 |
| A_23_P136460 | FAM13B | 9 |
| A_23_P146981 | | 9 |
| A_23_P151198 | PDAP1 | 9 |
| A_23_P154938 | HIRA | 9 |
| A_23_P157404 | AP1S1 | 9 |
| A_23_P159211 | | 9 |
| A_23_P159305 | TAF15 | 9 |
| A_23_P20285 | PDLIM2 | 9 |
| A_23_P208706 | BAX | 9 |
| A_23_P214658 | PBX2 | 9 |
| A_23_P215505 | RAPGEF5 | 9 |
| A_23_P252449 | HNRNPA0 | 9 |
| A_23_P258493 | LMNB1 | 9 |
| A_23_P28969 | CHMP4B | 9 |
| A_23_P311468 | ZNF428 | 9 |
| A_23_P331598 | IPO7 | 9 |
| A_23_P331813 | ZNF687 | 9 |
| A_23_P337849 | CELF3 | 9 |
| A_23_P346311 | BAX | 9 |
| A_23_P35309 | TAF5L | 9 |
| A_23_P353742 | AMBRA1 | 9 |
| A_23_P362719 | LSM14B | 9 |
| A_23_P370588 | HOXB8 | 9 |
| A_23_P39445 | MEX3D | 9 |
| A_23_P40240 | CTSZ | 9 |
| A_23_P500206 | IL17RE | 9 |
| A_23_P54781 | RBBP6 | 9 |
| A_23_P66854 | KRT20 | 9 |
| A_23_P70571 | SLC39A7 | 9 |
| A_23_P84475 | | 9 |
| A_23_P87580 | ANP32D | 9 |
| A_23_P92520 | ANP32C | 9 |
| A_24_P12413 | TRAM2 | 9 |
| A_24_P222126 | TH1L | 9 |
| A_24_P253827 | AP2B1 | 9 |
| A_24_P273679 | YAP1 | 9 |
| A_24_P329597 | UBQLN1 | 9 |
| A_24_P389517 | HNRNPK | 9 |
| A_24_P63950 | AP1S1 | 9 |
| A_24_P673209 | | 9 |
| A_24_P89257 | ERGIC1 | 9 |
| A_24_P910381 | | 9 |
| A_32_P107746 | ENSA | 9 |
| A_32_P133670 | ANP32A | 9 |
| A_32_P137632 | FBXL17 | 9 |
| A_32_P835626 | FBXO34 | 9 |
| A_32_P85978 | ZNF414 | 9 |
| A_32_P8666 | | 9 |
| A_23_P1014 | C1ORF97 | 10 |
| A_23_P105794 | EPSTI1 | 10 |
| A_23_P108673 | FAM176A | 10 |
| A_23_P111000 | PSMB9 | 10 |
| A_23_P126613 | AQP10 | 10 |
| A_23_P146233 | LPL | 10 |
| A_23_P155755 | CXCL6 | 10 |
| A_23_P19523 | MLN | 10 |
| A_23_P19529 | MLN | 10 |
| A_23_P200001 | NEXN | 10 |
| A_23_P211926 | WNT5A | 10 |
| A_23_P258136 | MXRA5 | 10 |
| A_23_P2789 | OLFM4 | 10 |
| A_23_P31161 | CPA2 | 10 |
| A_23_P404698 | COL29A1 | 10 |
| A_23_P41854 | CARD6 | 10 |
| A_23_P47616 | FOLH1 | 10 |
| A_23_P56630 | STAT1 | 10 |
| A_23_P62890 | GBP1 | 10 |
| A_23_P6909 | CCRL1 | 10 |
| A_23_P7212 | CFI | 10 |
| A_23_P7562 | ACSL6 | 10 |
| A_23_P78342 | LMAN1 | 10 |
| A_24_P181254 | OLFM4 | 10 |
| A_24_P188218 | MYL4 | 10 |
| A_24_P274270 | STAT1 | 10 |
| A_24_P311917 | BTN3A3 | 10 |
| A_24_P329065 | BTN3A1 | 10 |
| A_24_P416997 | APOL3 | 10 |
| A_24_P693986 | TRNP1 | 10 |
| A_24_P92472 | CFI | 10 |
| A_32_P107372 | GBP1 | 10 |
| A_32_P157391 | FOLH1B | 10 |
| A_32_P178513 | FOLH1 | 10 |
| A_32_P190864 | | 10 |
| A_23_P101683 | CLC | 11 |
| A_23_P105562 | VWF | 11 |
| A_23_P111701 | GNG11 | 11 |
| A_23_P119353 | RASIP1 | 11 |
| A_23_P121533 | SPON2 | 11 |
| A_23_P142533 | COL3A1 | 11 |
| A_23_P151805 | FBLN5 | 11 |
| A_23_P158593 | COL5A1 | 11 |
| A_23_P160559 | ECM1 | 11 |
| A_23_P203475 | PRKCDBP | 11 |
| A_23_P203957 | TMTC1 | 11 |
| A_23_P20566 | TPM2 | 11 |
| A_23_P207520 | COL1A1 | 11 |
| A_23_P211212 | COL18A1 | 11 |
| A_23_P211468 | | 11 |
| A_23_P216501 | TPM2 | 11 |
| A_23_P217428 | ARHGAP6 | 11 |
| A_23_P255111 | | 11 |
| A_23_P257144 | PXDN | 11 |
| A_23_P32444 | MXRA8 | 11 |
| A_23_P331748 | CD33 | 11 |
| A_23_P33196 | COL5A2 | 11 |
| A_23_P344421 | ROBO4 | 11 |
| A_23_P353035 | IGFBP7 | 11 |
| A_23_P365267 | SNED1 | 11 |
| A_23_P365614 | NOTCH4 | 11 |
| A_23_P383009 | IGFBP5 | 11 |
| A_23_P395438 | HTRA3 | 11 |
| A_23_P426305 | AOC3 | 11 |
| A_23_P432573 | MRGPRF | 11 |
| A_23_P43276 | GPR124 | 11 |
| A_23_P50175 | SIGLEC7 | 11 |
| A_23_P65388 | CLEC14A | 11 |
| A_23_P65518 | DACT1 | 11 |
| A_23_P66635 | CCL11 | 11 |
| A_23_P71316 | RBPMS | 11 |
| A_23_P82775 | SOX17 | 11 |
| A_23_P86470 | CH25H | 11 |
| A_23_P89431 | CCL2 | 11 |
| A_24_P156113 | EHD2 | 11 |
| A_24_P261417 | DKK3 | 11 |
| A_24_P277934 | COL1A2 | 11 |
| A_24_P291814 | COL12A1 | 11 |
| A_24_P376391 | PLXND1 | 11 |
| A_24_P389916 | LRRC32 | 11 |
| A_24_P935491 | COL3A1 | 11 |
| A_24_P944570 | PXDN | 11 |
| A_32_P220798 | CD34 | 11 |
| A_32_P74409 | AG2 | 11 |
| A_32_P97169 | GPC6 | 11 |
| A_23_P102160 | FAM82A1 | 12 |
| A_23_P110492 | MARCH6 | 12 |
| A_23_P123315 | | 12 |
| A_23_P127279 | FAM35A | 12 |
| A_23_P135123 | | 12 |
| A_23_P135132 | FRMD3 | 12 |
| A_23_P148969 | LRRC40 | 12 |
| A_23_P202269 | ANK3 | 12 |
| A_23_P214411 | GLO1 | 12 |
| A_23_P309261 | AKAP9 | 12 |
| A_23_P31798 | NAT2 | 12 |
| A_23_P347070 | PAG1 | 12 |
| A_23_P364625 | LRRC19 | 12 |
| A_23_P39718 | FEZ2 | 12 |
| A_23_P39766 | GLS | 12 |
| A_23_P428468 | ZNF292 | 12 |
| A_23_P50735 | ZNF181 | 12 |
| A_23_P51996 | STXBP3 | 12 |
| A_23_P62128 | MTM1 | 12 |
| A_23_P7282 | ELMOD2 | 12 |
| A_23_P73297 | MAGI1 | 12 |
| A_23_P92842 | SAR1B | 12 |
| A_24_P114334 | RMND5A | 12 |
| A_24_P131392 | FAM82A1 | 12 |
| A_24_P208909 | TRIM2 | 12 |
| A_24_P225845 | | 12 |
| A_24_P363134 | TRIM36 | 12 |
| A_24_P380330 | PANK3 | 12 |
| A_24_P594721 | | 12 |
| A_24_P623814 | | 12 |
| A_24_P67096 | ABCA5 | 12 |
| A_24_P880043 | PCGF5 | 12 |
| A_24_P921683 | FOXP2 | 12 |
| A_24_P924589 | | 12 |
| A_32_P1144 | | 12 |
| A_32_P132276 | | 12 |
| A_32_P155811 | CD2AP | 12 |
| A_32_P163996 | | 12 |
| A_32_P184220 | | 12 |
| A_32_P211276 | | 12 |
| A_32_P23525 | | 12 |
| A_32_P235796 | BBS12 | 12 |
| A_32_P33114 | KLB | 12 |
| A_32_P47643 | FAM110C | 12 |
| A_32_P61684 | PAG1 | 12 |
| A_32_P83811 | FAM47E | 12 |
| A_32_P87074 | | 12 |
| A_32_P98059 | | 12 |
| A_23_P102172 | CPO | 13 |
| A_23_P102864 | PRSS7 | 13 |
| A_23_P10559 | AATK | 13 |
| A_23_P117082 | HEBP1 | 13 |
| A_23_P119763 | ABCG5 | 13 |
| A_23_P12767 | CYP2C9 | 13 |
| A_23_P129064 | GATM | 13 |
| A_23_P12928 | SLC5A12 | 13 |
| A_23_P135417 | GSTA1 | 13 |
| A_23_P139146 | MS4A8B | 13 |
| A_23_P155786 | SULT1E1 | 13 |
| A_23_P157809 | PTGR1 | 13 |
| A_23_P158481 | CYP2C19 | 13 |
| A_23_P158484 | CYP2C19 | 13 |
| A_23_P158976 | ABCC2 | 13 |
| A_23_P160800 | NR0B2 | 13 |
| A_23_P160940 | ABCA4 | 13 |
| A_23_P203183 | APOC3 | 13 |
| A_23_P209251 | | 13 |
| A_23_P209564 | CYBRD1 | 13 |
| A_23_P212061 | MME | 13 |
| A_23_P213171 | MTTP | 13 |
| A_23_P214300 | GSTA2 | 13 |
| A_23_P214408 | UNC93A | 13 |
| A_23_P252817 | SST | 13 |
| A_23_P252981 | ACE2 | 13 |
| A_23_P253495 | GSTA3 | 13 |
| A_23_P25475 | SOAT2 | 13 |
| A_23_P257111 | FBP1 | 13 |
| A_23_P27107 | TM4SF5 | 13 |
| A_23_P28246 | SLC23A3 | 13 |
| A_23_P312150 | EDN2 | 13 |
| A_23_P319572 | NR1I3 | 13 |
| A_23_P325642 | PDIA2 | 13 |
| A_23_P333605 | ENPEP | 13 |
| A_23_P335214 | UGT2B4 | 13 |
| A_23_P335388 | C17ORF78 | 13 |
| A_23_P37914 | SLC5A11 | 13 |
| A_23_P389118 | ANO6 | 13 |
| A_23_P390984 | KCNH6 | 13 |
| A_23_P397120 | C19ORF77 | 13 |
| A_23_P413693 | C21ORF129 | 13 |
| A_23_P42897 | MGAM | 13 |
| A_23_P44569 | ABCC2 | 13 |
| A_23_P52121 | PDZK1 | 13 |
| A_23_P52480 | CYP2C18 | 13 |
| A_23_P5300 | CPS1 | 13 |
| A_23_P58953 | NQO2 | 13 |
| A_23_P66311 | DNASE1 | 13 |
| A_23_P67367 | DHDH | 13 |
| A_23_P67381 | SULT2A1 | 13 |
| A_23_P71570 | OSR2 | 13 |
| A_23_P7325 | BST1 | 13 |
| A_23_P76322 | PIK3C2G | 13 |
| A_23_P78353 | MEP1B | 13 |
| A_23_P79591 | APOB | 13 |
| A_23_P80491 | RBP2 | 13 |
| A_23_P80570 | AADAC | 13 |
| A_23_P83436 | PEPD | 13 |
| A_23_P85008 | MAOB | 13 |
| A_23_P85015 | MAOB | 13 |
| A_23_P87036 | APOA4 | 13 |
| A_23_P90510 | REEP6 | 13 |
| A_23_P93122 | MEP1A | 13 |
| A_23_P93141 | GSTA5 | 13 |
| A_23_P97923 | PHYHIPL | 13 |
| A_23_P98092 | OAT | 13 |
| A_24_P154868 | MEP1A | 13 |
| A_24_P186664 | | 13 |
| A_24_P224684 | SULT2A1 | 13 |
| A_24_P245838 | MGAT3 | 13 |
| A_24_P260101 | MME | 13 |
| A_24_P300394 | GSTA2 | 13 |
| A_24_P303454 | TIAM2 | 13 |
| A_24_P365129 | | 13 |
| A_24_P463929 | LOC285733 | 13 |
| A_24_P508946 | | 13 |
| A_24_P684186 | EMB | 13 |
| A_24_P98047 | SLC16A10 | 13 |
| A_32_P182812 | SLC6A4 | 13 |
| A_32_P206541 | EMB | 13 |
| A_32_P228341 | LOC149703 | 13 |
| A_32_P47027 | TMEM229A | 13 |
| A_32_P66843 | C19ORF69 | 13 |
| A_32_P81282 | | 13 |
| A_32_P96748 | | 13 |
| A_23_P103110 | MAFF | 14 |
| A_23_P106194 | FOS | 14 |
| A_23_P110712 | DUSP1 | 14 |
| A_23_P119943 | IGFBP2 | 14 |
| A_23_P121011 | CSRNP1 | 14 |
| A_23_P123503 | TRIB1 | 14 |
| A_23_P126248 | RNF186 | 14 |
| A_23_P126593 | S100A11 | 14 |
| A_23_P128230 | NR4A1 | 14 |
| A_23_P132115 | SIK1 | 14 |
| A_23_P13222 | RCN1 | 14 |
| A_23_P144959 | VCAN | 14 |
| A_23_P147106 | C9ORF167 | 14 |
| A_23_P154358 | PROM2 | 14 |
| A_23_P15727 | FKBP10 | 14 |
| A_23_P19663 | CTGF | 14 |
| A_23_P211039 | ADAMTS1 | 14 |
| A_23_P214079 | SPINK1 | 14 |
| A_23_P214080 | EGR1 | 14 |
| A_23_P217088 | AK1 | 14 |
| A_23_P24948 | KCNE3 | 14 |
| A_23_P26854 | RICH2 | 14 |
| A_23_P3038 | GPX2 | 14 |
| A_23_P342275 | ADAMTS1 | 14 |
| A_23_P34915 | ATF3 | 14 |
| A_23_P357207 | MRAP2 | 14 |
| A_23_P376449 | | 14 |
| A_23_P379475 | DHCR24 | 14 |
| A_23_P393620 | TFPI2 | 14 |
| A_23_P429998 | FOSB | 14 |
| A_23_P436259 | ERN1 | 14 |
| A_23_P46426 | CYR61 | 14 |
| A_23_P46429 | CYR61 | 14 |
| A_23_P50638 | LRG1 | 14 |
| A_23_P61180 | PLCXD1 | 14 |
| A_23_P70127 | TMED9 | 14 |
| A_23_P71379 | PSCA | 14 |
| A_23_P77103 | SORD | 14 |
| A_23_P87545 | IFITM3 | 14 |
| A_24_P140608 | HBEGF | 14 |
| A_24_P152325 | | 14 |
| A_24_P212997 | | 14 |
| A_24_P281264 | | 14 |
| A_24_P287043 | IFITM2 | 14 |
| A_24_P296772 | PPP1R14A | 14 |
| A_24_P314159 | APP | 14 |
| A_24_P319635 | MCL1 | 14 |
| A_24_P33895 | ATF3 | 14 |
| A_24_P357809 | C11ORF17 | 14 |
| A_24_P370946 | CYR61 | 14 |
| A_24_P53976 | GLUL | 14 |
| A_24_P57730 | MRPL52 | 14 |
| A_24_P68162 | DIP2B | 14 |
| A_24_P7040 | | 14 |
| A_24_P868905 | | 14 |
| A_24_P915692 | PHLDA1 | 14 |
| A_32_P100830 | KIF19 | 14 |
| A_32_P127153 | SORD | 14 |
| A_32_P142440 | PCSK9 | 14 |
| A_32_P151782 | | 14 |
| A_32_P163858 | SCD | 14 |
| A_32_P167592 | | 14 |
| A_32_P231617 | TM4SF1 | 14 |
| A_32_P234405 | | 14 |
| A_32_P25065 | | 14 |
| A_32_P327750 | | 14 |
| A_32_P331700 | | 14 |
| A_32_P69386 | | 14 |
| A_23_P103496 | GBP4 | 15 |
| A_23_P111888 | CTHRC1 | 15 |
| A_23_P112026 | IDO1 | 15 |
| A_23_P119196 | KLF2 | 15 |
| A_23_P122924 | INHBA | 15 |
| A_23_P127288 | IL2RA | 15 |
| A_23_P127584 | NNMT | 15 |
| A_23_P136173 | CSF2RA | 15 |
| A_23_P137697 | SELP | 15 |
| A_23_P143526 | S100B | 15 |
| A_23_P153320 | ICAM1 | 15 |
| A_23_P153616 | MADCAM1 | 15 |
| A_23_P18452 | CXCL9 | 15 |
| A_23_P200741 | DPT | 15 |
| A_23_P203419 | FADS1 | 15 |
| A_23_P203882 | MMP19 | 15 |
| A_23_P203888 | MMP19 | 15 |
| A_23_P207058 | SOCS3 | 15 |
| A_23_P215956 | MYC | 15 |
| A_23_P24104 | PLAU | 15 |
| A_23_P254741 | SOD3 | 15 |
| A_23_P2920 | SERPINA3 | 15 |
| A_23_P343221 | LILRB1 | 15 |
| A_23_P356581 | ROBO3 | 15 |
| A_23_P360754 | ADAMTS4 | 15 |
| A_23_P360777 | NRG1 | 15 |
| A_23_P369899 | TMEM158 | 15 |
| A_23_P51926 | PTAFR | 15 |
| A_23_P55270 | CCL18 | 15 |
| A_23_P62115 | TIMP1 | 15 |
| A_23_P63209 | HSD11B1 | 15 |
| A_23_P7144 | CXCL1 | 15 |
| A_23_P74290 | GBP5 | 15 |
| A_23_P76450 | PHLDA1 | 15 |
| A_23_P7827 | FAM26F | 15 |
| A_23_P84860 | FAM107A | 15 |
| A_23_P91390 | THBD | 15 |
| A_23_P98580 | FADS2 | 15 |
| A_24_P102821 | PTAFR | 15 |
| A_24_P131622 | FAM107A | 15 |
| A_24_P286114 | SLC1A3 | 15 |
| A_24_P299685 | PDPN | 15 |
| A_24_P329795 | C10ORF10 | 15 |
| A_24_P411561 | HAVCR2 | 15 |
| A_32_P108254 | FAM20A | 15 |
| A_32_P12372 | | 15 |
| A_32_P161855 | KIAA1199 | 15 |
| A_32_P164246 | FOXQ1 | 15 |
| A_32_P184916 | GNB4 | 15 |
| A_32_P196263 | ADAMTS9 | 15 |
| A_32_P217750 | IL3RA | 15 |
| A_32_P38323 | SERPINB9 | 15 |
| A_23_P103787 | HIPK1 | 16 |
| A_23_P121939 | PTCD2 | 16 |
| A_23_P146325 | ASAP1IT1 | 16 |
| A_23_P156809 | FAM119A | 16 |
| A_23_P156811 | FAM119A | 16 |
| A_23_P18406 | CAMK2N2 | 16 |
| A_23_P251216 | VPS13D | 16 |
| A_23_P326142 | C7ORF54 | 16 |
| A_23_P331348 | DOCK7 | 16 |
| A_23_P3413 | OTUD7A | 16 |
| A_23_P359052 | BOD1L | 16 |
| A_23_P362415 | UBE2B | 16 |
| A_23_P435183 | LRRFIP1 | 16 |
| A_23_P70161 | ITGA2 | 16 |
| A_24_P109432 | NBEAL1 | 16 |
| A_24_P117782 | SCRT2 | 16 |
| A_24_P118171 | MAGI3 | 16 |
| A_24_P128057 | MBNL1 | 16 |
| A_24_P140204 | PXK | 16 |
| A_24_P148263 | | 16 |
| A_24_P179183 | ANKRD12 | 16 |
| A_24_P194886 | EHBP1 | 16 |
| A_24_P195749 | | 16 |
| A_24_P25252 | ANKRD12 | 16 |
| A_24_P288915 | CCDC144B | 16 |
| A_24_P299007 | | 16 |
| A_24_P307195 | AASDH | 16 |
| A_24_P375592 | PHF20 | 16 |
| A_24_P384990 | LOC642852 | 16 |
| A_24_P42569 | | 16 |
| A_24_P462330 | | 16 |
| A_24_P469641 | RNF216 | 16 |
| A_24_P567944 | HCG18 | 16 |
| A_24_P579984 | | 16 |
| A_24_P636834 | | 16 |
| A_24_P643041 | RNF216L | 16 |
| A_24_P715530 | | 16 |
| A_24_P741023 | | 16 |
| A_24_P786172 | TNRC6B | 16 |
| A_24_P883109 | | 16 |
| A_24_P910372 | | 16 |
| A_24_P913216 | | 16 |
| A_24_P914102 | | 16 |
| A_24_P924521 | | 16 |
| A_24_P926025 | LOC100190986 | 16 |
| A_24_P930337 | | 16 |
| A_24_P932388 | | 16 |
| A_24_P934594 | AKAP8L | 16 |
| A_24_P936537 | LOC730658 | 16 |
| A_24_P942715 | | 16 |
| A_32_P110485 | | 16 |
| A_32_P118556 | | 16 |
| A_32_P120454 | | 16 |
| A_32_P122529 | SPDYE3 | 16 |
| A_32_P12703 | | 16 |
| A_32_P127978 | IKZF2 | 16 |
| A_32_P128857 | LOC286272 | 16 |
| A_32_P132766 | | 16 |
| A_32_P13565 | | 16 |
| A_32_P138503 | | 16 |
| A_32_P145385 | | 16 |
| A_32_P147969 | | 16 |
| A_32_P151244 | | 16 |
| A_32_P152544 | | 16 |
| A_32_P155512 | | 16 |
| A_32_P160615 | | 16 |
| A_32_P164378 | | 16 |
| A_32_P172545 | | 16 |
| A_32_P178537 | | 16 |
| A_32_P181131 | | 16 |
| A_32_P184746 | | 16 |
| A_32_P185530 | | 16 |
| A_32_P191074 | | 16 |
| A_32_P193908 | | 16 |
| A_32_P200429 | | 16 |
| A_32_P201150 | | 16 |
| A_32_P208200 | | 16 |
| A_32_P211026 | | 16 |
| A_32_P212108 | | 16 |
| A_32_P216004 | | 16 |
| A_32_P216369 | CCDC144A | 16 |
| A_32_P220580 | | 16 |
| A_32_P220700 | | 16 |
| A_32_P224638 | | 16 |
| A_32_P225301 | | 16 |
| A_32_P233250 | | 16 |
| A_32_P33304 | ANK3 | 16 |
| A_32_P42666 | | 16 |
| A_32_P45285 | | 16 |
| A_32_P48198 | RUNDC2B | 16 |
| A_32_P5542 | | 16 |
| A_32_P57013 | | 16 |
| A_32_P65067 | | 16 |
| A_32_P6972 | | 16 |
| A_32_P77665 | | 16 |
| A_32_P85880 | | 16 |
| A_32_P89646 | | 16 |
| A_32_P95541 | | 16 |
| A_32_P974 | | 16 |
| A_23_P104199 | ITGB1 | 17 |
| A_23_P110175 | CTSO | 17 |
| A_23_P110569 | TRIM36 | 17 |
| A_23_P123763 | C9ORF82 | 17 |
| A_23_P126057 | SCP2 | 17 |
| A_23_P127054 | PANK1 | 17 |
| A_23_P130429 | ROCK1 | 17 |
| A_23_P133120 | TMEM144 | 17 |
| A_23_P144179 | CPOX | 17 |
| A_23_P147995 | PICALM | 17 |
| A_23_P154130 | MAP4K3 | 17 |
| A_23_P156425 | MAN1A1 | 17 |
| A_23_P156431 | MAN1A1 | 17 |
| A_23_P158330 | UGT1A8 | 17 |
| A_23_P159764 | POF1B | 17 |
| A_23_P163079 | GCH1 | 17 |
| A_23_P167005 | GPR160 | 17 |
| A_23_P168818 | IMPA1 | 17 |
| A_23_P19061 | UBTD2 | 17 |
| A_23_P201619 | NEK7 | 17 |
| A_23_P201758 | CD46 | 17 |
| A_23_P203658 | PICALM | 17 |
| A_23_P203751 | TMEM135 | 17 |
| A_23_P204564 | PPP1R12A | 17 |
| A_23_P211909 | PLS1 | 17 |
| A_23_P22350 | GRAMD3 | 17 |
| A_23_P254756 | CD164 | 17 |
| A_23_P258234 | DCBLD1 | 17 |
| A_23_P30175 | ERBB2IP | 17 |
| A_23_P328729 | KLHL8 | 17 |
| A_23_P328836 | LCOR | 17 |
| A_23_P337875 | PAQR3 | 17 |
| A_23_P34460 | SLC35A3 | 17 |
| A_23_P348894 | SEC23A | 17 |
| A_23_P363831 | FBXO3 | 17 |
| A_23_P37598 | NPTN | 17 |
| A_23_P382460 | NIPA1 | 17 |
| A_23_P412029 | PUS10 | 17 |
| A_23_P45361 | GLUD2 | 17 |
| A_23_P62133 | MTM1 | 17 |
| A_23_P69791 | AP1AR | 17 |
| A_23_P72568 | SNX4 | 17 |
| A_23_P74794 | ABCD3 | 17 |
| A_23_P86421 | NCOA4 | 17 |
| A_23_P86424 | NCOA4 | 17 |
| A_23_P9056 | RB1CC1 | 17 |
| A_23_P9932 | PDCD4 | 17 |
| A_24_P100266 | ST13 | 17 |
| A_24_P102080 | RAB2A | 17 |
| A_24_P123190 | PLD1 | 17 |
| A_24_P135748 | GRTP1 | 17 |
| A_24_P137602 | CEP120 | 17 |
| A_24_P144314 | | 17 |
| A_24_P156922 | SCP2 | 17 |
| A_24_P16361 | | 17 |
| A_24_P167806 | IDH3A | 17 |
| A_24_P174346 | ATP5G3 | 17 |
| A_24_P181101 | TMEM135 | 17 |
| A_24_P200652 | C6ORF62 | 17 |
| A_24_P222872 | UGT1A6 | 17 |
| A_24_P226076 | METAP1 | 17 |
| A_24_P246591 | | 17 |
| A_24_P250815 | POF1B | 17 |
| A_24_P285522 | MAP4K3 | 17 |
| A_24_P328471 | PLEKHA3 | 17 |
| A_24_P329152 | CD2AP | 17 |
| A_24_P334378 | UGT2A3 | 17 |
| A_24_P340036 | RNF128 | 17 |
| A_24_P356592 | PRKAR1A | 17 |
| A_24_P374427 | ZDHHC21 | 17 |
| A_24_P374943 | CXADR | 17 |
| A_24_P387609 | ISCA1 | 17 |
| A_24_P392774 | HSD17B12 | 17 |
| A_24_P401392 | | 17 |
| A_24_P403168 | PRPF4B | 17 |
| A_24_P405552 | SERP1 | 17 |
| A_24_P418717 | BMPR1A | 17 |
| A_24_P518369 | | 17 |
| A_24_P541576 | | 17 |
| A_24_P548453 | CRYZ | 17 |
| A_24_P61864 | CCDC47 | 17 |
| A_24_P622697 | | 17 |
| A_24_P71904 | HPGD | 17 |
| A_24_P759477 | ITGB8 | 17 |
| A_24_P846755 | | 17 |
| A_24_P89987 | EPB41L4B | 17 |
| A_24_P917711 | PRKAB2 | 17 |
| A_24_P921897 | HOOK1 | 17 |
| A_24_P924816 | SLC2A13 | 17 |
| A_24_P933675 | HMGN1 | 17 |
| A_24_P940197 | PAPD5 | 17 |
| A_32_P102252 | VDAC1P | 17 |
| A_32_P125135 | | 17 |
| A_32_P163169 | VDAC1 | 17 |
| A_32_P191895 | | 17 |
| A_32_P193218 | LPP | 17 |
| A_32_P202703 | | 17 |
| A_32_P205637 | PARD6B | 17 |
| A_32_P211188 | PPARGC1B | 17 |
| A_32_P223859 | SMPDL3A | 17 |
| A_32_P224840 | AKTIP | 17 |
| A_32_P225355 | CPEB2 | 17 |
| A_32_P45009 | IDH1 | 17 |
| A_32_P69536 | | 17 |
| A_23_P104346 | PIP4K2A | 18 |
| A_23_P123478 | PDE7A | 18 |
| A_23_P13663 | FAM60A | 18 |
| A_23_P157679 | UTP23 | 18 |
| A_23_P158794 | RSRC1 | 18 |
| A_23_P207680 | ARL17B | 18 |
| A_23_P215009 | FAM65B | 18 |
| A_23_P382654 | ASCC3 | 18 |
| A_23_P391275 | RCAN3 | 18 |
| A_23_P60047 | TACC1 | 18 |
| A_23_P82474 | TWISTNB | 18 |
| A_24_P136807 | RFC1 | 18 |
| A_24_P145911 | TRA2B | 18 |
| A_24_P150665 | RUNDC2A | 18 |
| A_24_P16291 | | 18 |
| A_24_P187131 | FRYL | 18 |
| A_24_P212457 | GON4L | 18 |
| A_24_P214598 | PPM1K | 18 |
| A_24_P247596 | | 18 |
| A_24_P248251 | HLTF | 18 |
| A_24_P257579 | EPB41L4A | 18 |
| A_24_P298174 | CBX1 | 18 |
| A_24_P362850 | PBRM1 | 18 |
| A_24_P418294 | CEP97 | 18 |
| A_24_P418637 | MACF1 | 18 |
| A_24_P65941 | C21ORF96 | 18 |
| A_24_P865 | C6ORF204 | 18 |
| A_24_P911179 | ASPM | 18 |
| A_24_P919995 | | 18 |
| A_24_P927222 | RBM27 | 18 |
| A_24_P928281 | FGD2 | 18 |
| A_24_P932203 | MYST3 | 18 |
| A_32_P120604 | SNX29 | 18 |
| A_32_P127200 | | 18 |
| A_32_P180971 | LOC728323 | 18 |
| A_32_P227657 | | 18 |
| A_32_P54616 | KIAA1407 | 18 |
| A_32_P57057 | USP15 | 18 |
| A_32_P66020 | RUNDC2A | 18 |
| A_32_P71171 | | 18 |
| A_23_P104798 | IL18 | 19 |
| A_23_P120354 | ANKRD57 | 19 |
| A_23_P128613 | KDELC1 | 19 |
| A_23_P133036 | SLC34A2 | 19 |
| A_23_P136849 | | 19 |
| A_23_P156957 | NCOA7 | 19 |
| A_23_P170908 | NUBPL | 19 |
| A_23_P206661 | NQO1 | 19 |
| A_23_P212089 | NFKBIZ | 19 |
| A_23_P213562 | F2R | 19 |
| A_23_P3532 | LITAF | 19 |
| A_23_P46141 | CTSS | 19 |
| A_23_P51966 | | 19 |
| A_23_P76435 | GATC | 19 |
| A_24_P12438 | NCOA7 | 19 |
| A_24_P157087 | CASP8 | 19 |
| A_24_P214556 | | 19 |
| A_24_P225679 | IRS1 | 19 |
| A_24_P283221 | NAIP | 19 |
| A_24_P336853 | PNO1 | 19 |
| A_24_P65597 | | 19 |
| A_24_P936911 | | 19 |
| A_32_P103464 | | 19 |
| A_32_P136588 | | 19 |
| A_32_P140501 | | 19 |
| A_32_P177595 | | 19 |
| A_32_P68142 | | 19 |
| A_23_P104804 | ZBTB16 | 20 |
| A_23_P111206 | FKBP5 | 20 |
| A_23_P115726 | SLC16A9 | 20 |
| A_23_P138524 | CPXM2 | 20 |
| A_23_P155257 | FOXP1 | 20 |
| A_23_P155688 | SPINK2 | 20 |
| A_23_P210690 | TRIB3 | 20 |
| A_23_P251984 | PSPH | 20 |
| A_23_P253368 | HOXA10 | 20 |
| A_23_P29096 | PDE9A | 20 |
| A_23_P323180 | HOXD3 | 20 |
| A_23_P326913 | PRUNE2 | 20 |
| A_23_P363316 | HOXB5 | 20 |
| A_23_P39294 | PLAC2 | 20 |
| A_23_P403335 | EXPH5 | 20 |
| A_23_P500998 | HOXA9 | 20 |
| A_23_P53039 | LDHC | 20 |
| A_23_P55281 | HOXB7 | 20 |
| A_23_P6481 | TNRC6B | 20 |
| A_23_P70968 | HOXA7 | 20 |
| A_23_P93772 | HOXA5 | 20 |
| A_24_P168925 | CHRDL1 | 20 |
| A_24_P184692 | NKX2-1 | 20 |
| A_24_P213794 | CCRN4L | 20 |
| A_24_P321581 | SLC38A4 | 20 |
| A_24_P334208 | | 20 |
| A_24_P38081 | FKBP5 | 20 |
| A_24_P399220 | HOXB3 | 20 |
| A_24_P40626 | GREM2 | 20 |
| A_24_P500584 | XIST | 20 |
| A_24_P580248 | | 20 |
| A_24_P759584 | DIO3OS | 20 |
| A_24_P762886 | KIAA0485 | 20 |
| A_24_P77904 | HOXA10 | 20 |
| A_24_P922261 | SRGAP1 | 20 |
| A_24_P931859 | PDE9A | 20 |
| A_24_P934435 | | 20 |
| A_32_P101799 | | 20 |
| A_32_P115717 | | 20 |
| A_32_P126362 | | 20 |
| A_32_P143000 | FAM189A1 | 20 |
| A_32_P150269 | | 20 |
| A_32_P166653 | | 20 |
| A_32_P174285 | | 20 |
| A_32_P184727 | KPNB1 | 20 |
| A_32_P224727 | | 20 |
| A_32_P232035 | LOC100270746 | 20 |
| A_32_P30966 | | 20 |
| A_32_P3783 | | 20 |
| A_32_P43325 | | 20 |
| A_32_P45375 | | 20 |
| A_32_P627 | TAF9B | 20 |
| A_32_P76122 | | 20 |
| A_32_P77571 | | 20 |
| A_32_P78816 | PSPH | 20 |
| A_32_P84242 | FAM169A | 20 |
| A_23_P105012 | HRASLS2 | 21 |
| A_23_P105856 | SLC46A3 | 21 |
| A_23_P118065 | HSD17B2 | 21 |
| A_23_P128728 | ARG2 | 21 |
| A_23_P129144 | GCOM1 | 21 |
| A_23_P14515 | ACOT4 | 21 |
| A_23_P145711 | C7ORF10 | 21 |
| A_23_P155868 | PGRMC2 | 21 |
| A_23_P163408 | SCAPER | 21 |
| A_23_P202004 | PRTFDC1 | 21 |
| A_23_P205074 | SLC46A3 | 21 |
| A_23_P210060 | C2ORF88 | 21 |
| A_23_P233 | FMO5 | 21 |
| A_23_P251453 | HNF4G | 21 |
| A_23_P253301 | PFN2 | 21 |
| A_23_P27040 | TMEM98 | 21 |
| A_23_P326963 | C3ORF59 | 21 |
| A_23_P34478 | GIPC2 | 21 |
| A_23_P359655 | ZNF664 | 21 |
| A_23_P366983 | TRHDE | 21 |
| A_23_P375541 | LCORL | 21 |
| A_23_P390032 | TMEM20 | 21 |
| A_23_P400406 | ANKRD43 | 21 |
| A_23_P404536 | ENPP3 | 21 |
| A_23_P408271 | HSD17B11 | 21 |
| A_23_P418234 | PHLPP2 | 21 |
| A_23_P436284 | OSTBETA | 21 |
| A_23_P4536 | EPB41L3 | 21 |
| A_23_P47565 | LDHA | 21 |
| A_23_P499 | B3GALT2 | 21 |
| A_23_P502654 | SHMT1 | 21 |
| A_23_P51918 | SEC16B | 21 |
| A_23_P56933 | RTN4 | 21 |
| A_23_P60599 | UGT1A6 | 21 |
| A_23_P83098 | ALDH1A1 | 21 |
| A_23_P8311 | TTRAP | 21 |
| A_23_P93641 | AKR1B10 | 21 |
| A_23_P9875 | TESK2 | 21 |
| A_23_P993 | NR5A2 | 21 |
| A_24_P100301 | GIPC2 | 21 |
| A_24_P129341 | AKR1B10 | 21 |
| A_24_P170283 | | 21 |
| A_24_P174755 | SLC22A5 | 21 |
| A_24_P182182 | SLC25A5 | 21 |
| A_24_P182183 | SLC25A5 | 21 |
| A_24_P277875 | CHN2 | 21 |
| A_24_P296280 | FAM82A2 | 21 |
| A_24_P298228 | | 21 |
| A_24_P307126 | | 21 |
| A_24_P323682 | | 21 |
| A_24_P383704 | | 21 |
| A_24_P385732 | OSTALPHA | 21 |
| A_24_P396980 | PFN2 | 21 |
| A_24_P453970 | | 21 |
| A_24_P603890 | | 21 |
| A_24_P62530 | RHOU | 21 |
| A_24_P639679 | SNORD123 | 21 |
| A_24_P679796 | | 21 |
| A_24_P852601 | HNF4G | 21 |
| A_32_P101264 | TMEM20 | 21 |
| A_32_P123 | | 21 |
| A_32_P140153 | | 21 |
| A_32_P44568 | LDHA | 21 |
| A_32_P50670 | | 21 |
| A_23_P105368 | MGC16384 | 22 |
| A_23_P143926 | ULK4 | 22 |
| A_23_P209712 | SP100 | 22 |
| A_23_P255653 | TNFRSF10A | 22 |
| A_23_P320728 | | 22 |
| A_23_P359870 | | 22 |
| A_23_P3767 | NFAT5 | 22 |
| A_23_P394562 | | 22 |
| A_23_P396867 | HM13 | 22 |
| A_23_P502470 | IL6ST | 22 |
| A_23_P56938 | REL | 22 |
| A_23_P58579 | TRIM52 | 22 |
| A_23_P59836 | METTL2B | 22 |
| A_23_P70069 | | 22 |
| A_23_P80940 | PPAT | 22 |
| A_23_P82488 | | 22 |
| A_23_P84246 | | 22 |
| A_24_P118211 | | 22 |
| A_24_P136551 | NPLOC4 | 22 |
| A_24_P191207 | GABPB2 | 22 |
| A_24_P301954 | MGC16384 | 22 |
| A_24_P333341 | SCAMP4 | 22 |
| A_24_P391811 | DOCK10 | 22 |
| A_24_P400161 | C1ORF84 | 22 |
| A_24_P410554 | KIAA1659 | 22 |
| A_24_P450493 | | 22 |
| A_24_P462725 | | 22 |
| A_24_P50937 | LOC100128842 | 22 |
| A_24_P539226 | | 22 |
| A_24_P592871 | | 22 |
| A_24_P659415 | | 22 |
| A_24_P792130 | LOC100289574 | 22 |
| A_24_P848352 | | 22 |
| A_24_P912439 | AKNA | 22 |
| A_24_P919168 | | 22 |
| A_24_P919920 | ARV1 | 22 |
| A_24_P935682 | UNQ6228 | 22 |
| A_24_P937965 | RAB3GAP1 | 22 |
| A_24_P94054 | STK4 | 22 |
| A_32_P102627 | | 22 |
| A_32_P121794 | EXOSC6 | 22 |
| A_32_P149716 | | 22 |
| A_32_P169222 | | 22 |
| A_32_P177843 | | 22 |
| A_32_P192293 | | 22 |
| A_32_P26422 | | 22 |
| A_32_P34064 | | 22 |
| A_32_P38500 | | 22 |
| A_32_P40456 | | 22 |
| A_32_P74932 | | 22 |
| A_32_P84714 | | 22 |
| A_32_P99700 | | 22 |
| A_23_P106 | USP33 | 23 |
| A_23_P106602 | CRISPLD2 | 23 |
| A_23_P10699 | SNX12 | 23 |
| A_23_P126186 | DEGS1 | 23 |
| A_23_P126782 | F3 | 23 |
| A_23_P12950 | KBTBD4 | 23 |
| A_23_P135787 | GOLGB1 | 23 |
| A_23_P15857 | PPP4R1 | 23 |
| A_23_P168419 | MLL3 | 23 |
| A_23_P19936 | KDELR2 | 23 |
| A_23_P203841 | BAZ2A | 23 |
| A_23_P204581 | TXNRD1 | 23 |
| A_23_P205389 | MOAP1 | 23 |
| A_23_P21316 | PRUNE | 23 |
| A_23_P31844 | ATP6V1B2 | 23 |
| A_23_P342641 | SLC44A5 | 23 |
| A_23_P359131 | NIPA1 | 23 |
| A_23_P36445 | TMED2 | 23 |
| A_23_P36448 | TMED2 | 23 |
| A_23_P39590 | XDH | 23 |
| A_23_P424051 | LOC153328 | 23 |
| A_23_P51538 | GPA33 | 23 |
| A_23_P54597 | RSL1D1 | 23 |
| A_23_P60339 | C9ORF64 | 23 |
| A_23_P67913 | GMPPA | 23 |
| A_23_P70355 | SERPINB6 | 23 |
| A_23_P79259 | SH3BP4 | 23 |
| A_23_P83414 | PPP1CB | 23 |
| A_23_P91859 | TRANK1 | 23 |
| A_23_P96165 | C11ORF80 | 23 |
| A_23_P96641 | PRPS2 | 23 |
| A_23_P99249 | SUCLG2 | 23 |
| A_24_P100228 | XBP1 | 23 |
| A_24_P123012 | YWHAE | 23 |
| A_24_P13572 | DLST | 23 |
| A_24_P138091 | PTPN3 | 23 |
| A_24_P140171 | CRTAP | 23 |
| A_24_P152793 | | 23 |
| A_24_P15610 | | 23 |
| A_24_P169574 | | 23 |
| A_24_P174550 | RHOA | 23 |
| A_24_P190424 | RAB8A | 23 |
| A_24_P193570 | CNOT1 | 23 |
| A_24_P205364 | SHMT1 | 23 |
| A_24_P212539 | GALM | 23 |
| A_24_P229726 | | 23 |
| A_24_P230675 | SOCS2 | 23 |
| A_24_P247749 | RAB21 | 23 |
| A_24_P272561 | ZDHHC13 | 23 |
| A_24_P295745 | HSPA8 | 23 |
| A_24_P31235 | EIF5A | 23 |
| A_24_P322191 | CALCOCO2 | 23 |
| A_24_P328320 | GORASP2 | 23 |
| A_24_P332721 | | 23 |
| A_24_P333445 | MORF4L2 | 23 |
| A_24_P333479 | SLC39A14 | 23 |
| A_24_P33444 | YWHAE | 23 |
| A_24_P337380 | HNRNPH1 | 23 |
| A_24_P375609 | EIF5A | 23 |
| A_24_P378368 | MBTPS1 | 23 |
| A_24_P387839 | DEGS1 | 23 |
| A_24_P38930 | CTNND1 | 23 |
| A_24_P398585 | UNG | 23 |
| A_24_P402779 | PARP3 | 23 |
| A_24_P409816 | | 23 |
| A_24_P414999 | LAPTM4B | 23 |
| A_24_P42264 | LYZ | 23 |
| A_24_P470754 | | 23 |
| A_24_P59220 | POTEF | 23 |
| A_24_P684119 | LOC100130288 | 23 |
| A_24_P714134 | | 23 |
| A_24_P714707 | | 23 |
| A_24_P75979 | | 23 |
| A_24_P76210 | | 23 |
| A_24_P764598 | PTP4A2 | 23 |
| A_24_P80532 | CCNG2 | 23 |
| A_24_P830696 | | 23 |
| A_24_P881527 | CTNND1 | 23 |
| A_24_P89457 | CDKN1A | 23 |
| A_24_P94034 | USP22 | 23 |
| A_24_P98723 | LIN7C | 23 |
| A_32_P39216 | YWHAE | 23 |
| A_32_P77762 | | 23 |
| A_23_P10640 | ENPP7 | 24 |
| A_23_P111766 | | 24 |
| A_23_P112086 | DEFA5 | 24 |
| A_23_P114008 | TM4SF20 | 24 |
| A_23_P114423 | RGN | 24 |
| A_23_P116235 | MDK | 24 |
| A_23_P119936 | REG3A | 24 |
| A_23_P124300 | BCMO1 | 24 |
| A_23_P149019 | BAI2 | 24 |
| A_23_P15692 | GPR172B | 24 |
| A_23_P157793 | CA9 | 24 |
| A_23_P159325 | ANGPTL4 | 24 |
| A_23_P161171 | ASAH2 | 24 |
| A_23_P169503 | CEL | 24 |
| A_23_P170719 | | 24 |
| A_23_P20075 | NPC1L1 | 24 |
| A_23_P208126 | SERPINB5 | 24 |
| A_23_P218111 | SERPINA1 | 24 |
| A_23_P253896 | NPNT | 24 |
| A_23_P255345 | VNN1 | 24 |
| A_23_P30603 | DDO | 24 |
| A_23_P310274 | PRSS2 | 24 |
| A_23_P321949 | PLA2G2A | 24 |
| A_23_P380754 | PRSS1 | 24 |
| A_23_P395001 | SLC2A12 | 24 |
| A_23_P425681 | CCK | 24 |
| A_23_P428129 | CDKN1C | 24 |
| A_23_P428298 | UNC5CL | 24 |
| A_23_P45999 | FBXO2 | 24 |
| A_23_P500010 | KLK12 | 24 |
| A_23_P50146 | SIGLEC15 | 24 |
| A_23_P51217 | CLCA1 | 24 |
| A_23_P51690 | RHBG | 24 |
| A_23_P55828 | CCL25 | 24 |
| A_23_P62857 | | 24 |
| A_23_P71268 | AZGP1 | 24 |
| A_23_P79217 | LCT | 24 |
| A_23_P82886 | DEFA6 | 24 |
| A_23_P9232 | GCNT1 | 24 |
| A_23_P97339 | SLC16A4 | 24 |
| A_24_P103434 | UNC93A | 24 |
| A_24_P179244 | LOC100128979 | 24 |
| A_24_P253003 | WNT11 | 24 |
| A_24_P337700 | VNN1 | 24 |
| A_24_P363711 | DEFA6 | 24 |
| A_24_P385326 | PEAR1 | 24 |
| A_24_P49267 | | 24 |
| A_24_P526190 | LOC643201 | 24 |
| A_24_P53778 | ITLN2 | 24 |
| A_24_P58673 | REG4 | 24 |
| A_24_P833256 | | 24 |
| A_32_P170481 | LOC100240735 | 24 |
| A_32_P29408 | LOC100240735 | 24 |
| A_32_P65628 | REG3G | 24 |
| A_32_P94444 | PRSS2 | 24 |
| A_23_P106617 | WFDC1 | 25 |
| A_23_P109508 | NCF4 | 25 |
| A_23_P125977 | C1QC | 25 |
| A_23_P133474 | GPX3 | 25 |
| A_23_P137157 | RENBP | 25 |
| A_23_P141508 | CLEC10A | 25 |
| A_23_P147641 | TCEA2 | 25 |
| A_23_P14769 | FES | 25 |
| A_23_P151297 | TENC1 | 25 |
| A_23_P156890 | TCF21 | 25 |
| A_23_P163787 | MMP2 | 25 |
| A_23_P165848 | EMILIN1 | 25 |
| A_23_P17481 | SIGLEC1 | 25 |
| A_23_P1759 | AMICA1 | 25 |
| A_23_P200928 | NID1 | 25 |
| A_23_P211233 | COL6A2 | 25 |
| A_23_P217326 | FHL1 | 25 |
| A_23_P26771 | CD300C | 25 |
| A_23_P32500 | STAB1 | 25 |
| A_23_P332190 | IRF8 | 25 |
| A_23_P33643 | IL7R | 25 |
| A_23_P37702 | TPSAB1 | 25 |
| A_23_P39465 | BST2 | 25 |
| A_23_P433016 | FBLN1 | 25 |
| A_23_P5983 | PLTP | 25 |
| A_23_P70095 | CD74 | 25 |
| A_23_P87013 | TAGLN | 25 |
| A_23_P90710 | DES | 25 |
| A_23_P96568 | FLNA | 25 |
| A_24_P11315 | OLFML3 | 25 |
| A_24_P14634 | EMID1 | 25 |
| A_24_P15702 | | 25 |
| A_24_P215653 | CLEC14A | 25 |
| A_24_P222655 | C1QA | 25 |
| A_24_P243528 | HLA-DPA1 | 25 |
| A_24_P246626 | | 25 |
| A_24_P276628 | PPT1 | 25 |
| A_24_P326084 | HLA-DQA1 | 25 |
| A_24_P335025 | ENTPD1 | 25 |
| A_24_P402242 | COL3A1 | 25 |
| A_24_P46066 | CXCR6 | 25 |
| A_24_P79300 | CLDN5 | 25 |
| A_32_P24832 | OLFML3 | 25 |
| A_23_P108501 | EPHA4 | 26 |
| A_23_P114466 | TBL1Y | 26 |
| A_23_P127948 | ADM | 26 |
| A_23_P143935 | PIGZ | 26 |
| A_23_P160336 | LEFTY1 | 26 |
| A_23_P16225 | BEST2 | 26 |
| A_23_P162668 | CPM | 26 |
| A_23_P163336 | CA12 | 26 |
| A_23_P168916 | CA1 | 26 |
| A_23_P20363 | FAM164A | 26 |
| A_23_P207850 | TNS4 | 26 |
| A_23_P210465 | PI3 | 26 |
| A_23_P212779 | PARM1 | 26 |
| A_23_P217280 | NOX1 | 26 |
| A_23_P259071 | AREG | 26 |
| A_23_P28898 | PLCB4 | 26 |
| A_23_P30126 | FGFBP1 | 26 |
| A_23_P304921 | NOX1 | 26 |
| A_23_P3083 | | 26 |
| A_23_P312300 | SCGB2A1 | 26 |
| A_23_P316410 | NOX1 | 26 |
| A_23_P320216 | FAM55D | 26 |
| A_23_P372234 | CA12 | 26 |
| A_23_P396765 | PGM2L1 | 26 |
| A_23_P42257 | IER3 | 26 |
| A_23_P47790 | METTL1 | 26 |
| A_23_P571 | SLC2A1 | 26 |
| A_23_P70359 | C6ORF59 | 26 |
| A_23_P79398 | IL1R2 | 26 |
| A_23_P85250 | CD24 | 26 |
| A_23_P8913 | CA2 | 26 |
| A_24_P156734 | FLJ30430 | 26 |
| A_24_P191781 | PARM1 | 26 |
| A_24_P203630 | ANKRD36 | 26 |
| A_24_P254789 | COL14A1 | 26 |
| A_24_P280983 | HOXA11AS | 26 |
| A_24_P459522 | | 26 |
| A_24_P645914 | EFNA5 | 26 |
| A_24_P79403 | PF4 | 26 |
| A_24_P917819 | C21ORF99 | 26 |
| A_24_P928439 | | 26 |
| A_24_P938352 | CPM | 26 |
| A_24_P945113 | ACVRL1 | 26 |
| A_32_P170406 | TLN2 | 26 |
| A_32_P197367 | | 26 |
| A_32_P218025 | | 26 |
| A_32_P231250 | | 26 |
| A_32_P29551 | | 26 |
| A_32_P61298 | | 26 |
| A_23_P108514 | STK16 | 27 |
| A_23_P131074 | THEG | 27 |
| A_23_P162449 | SRGAP1 | 27 |
| A_23_P31858 | ST18 | 27 |
| A_23_P38757 | SLC14A1 | 27 |
| A_23_P501722 | TSPAN32 | 27 |
| A_23_P54736 | GNG13 | 27 |
| A_24_P111096 | PFKFB3 | 27 |
| A_24_P206604 | PFKFB3 | 27 |
| A_24_P253454 | RGMA | 27 |
| A_24_P324141 | MUC5B | 27 |
| A_24_P332081 | JAKMIP3 | 27 |
| A_24_P341000 | FLJ35379 | 27 |
| A_24_P375205 | MKL2 | 27 |
| A_24_P409182 | | 27 |
| A_24_P7584 | LY6G5C | 27 |
| A_24_P85258 | KIAA1751 | 27 |
| A_24_P874508 | | 27 |
| A_24_P928031 | | 27 |
| A_24_P935852 | | 27 |
| A_32_P128368 | | 27 |
| A_32_P133840 | TMCC2 | 27 |
| A_32_P151152 | | 27 |
| A_32_P185741 | | 27 |
| A_32_P60707 | | 27 |
| A_32_P7974 | TDRD10 | 27 |
| A_23_P108823 | OSBPL6 | 28 |
| A_23_P136116 | TMEM195 | 28 |
| A_23_P140009 | SLC10A2 | 28 |
| A_23_P146783 | NEB | 28 |
| A_23_P149998 | PBLD | 28 |
| A_23_P157371 | FAM3C | 28 |
| A_23_P159076 | SLC17A8 | 28 |
| A_23_P160406 | KCTD3 | 28 |
| A_23_P164436 | ASPA | 28 |
| A_23_P167276 | PAQR3 | 28 |
| A_23_P18672 | GBA3 | 28 |
| A_23_P20316 | CA3 | 28 |
| A_23_P210675 | SYCP2 | 28 |
| A_23_P211047 | BACH1 | 28 |
| A_23_P216468 | SLC1A1 | 28 |
| A_23_P21990 | SLC23A1 | 28 |
| A_23_P24004 | IFIT2 | 28 |
| A_23_P25069 | LOC728715 | 28 |
| A_23_P253012 | GRAMD1C | 28 |
| A_23_P256008 | ZDHHC11 | 28 |
| A_23_P302060 | IFNE | 28 |
| A_23_P308800 | GLS | 28 |
| A_23_P312901 | GPR112 | 28 |
| A_23_P31376 | LRRN3 | 28 |
| A_23_P326852 | CACNB2 | 28 |
| A_23_P336342 | | 28 |
| A_23_P354341 | CD160 | 28 |
| A_23_P356585 | HLF | 28 |
| A_23_P358917 | CYP3A7 | 28 |
| A_23_P385126 | DEPDC7 | 28 |
| A_23_P404606 | C5ORF41 | 28 |
| A_23_P420442 | SEMA6D | 28 |
| A_23_P43157 | MYBL1 | 28 |
| A_23_P434430 | ZNF439 | 28 |
| A_23_P44207 | ACOT12 | 28 |
| A_23_P500093 | RGS13 | 28 |
| A_23_P501193 | KCNJ16 | 28 |
| A_23_P52266 | IFIT1 | 28 |
| A_23_P71226 | SLC13A1 | 28 |
| A_23_P74900 | ESRRG | 28 |
| A_23_P8801 | CYP3A5 | 28 |
| A_23_P8961 | IL7 | 28 |
| A_23_P92928 | C6 | 28 |
| A_24_P105564 | PRKAB2 | 28 |
| A_24_P124647 | SLC17A8 | 28 |
| A_24_P153456 | ZDHHC11 | 28 |
| A_24_P294233 | GLS | 28 |
| A_24_P355057 | SLC13A1 | 28 |
| A_24_P373475 | ASPA | 28 |
| A_24_P38754 | CISD1 | 28 |
| A_24_P393571 | GDA | 28 |
| A_24_P511877 | | 28 |
| A_24_P63537 | ERAP1 | 28 |
| A_24_P636882 | STK17B | 28 |
| A_24_P671115 | | 28 |
| A_24_P747419 | | 28 |
| A_24_P80500 | BDH2 | 28 |
| A_24_P912228 | LOC100131053 | 28 |
| A_24_P934387 | SLC5A12 | 28 |
| A_32_P100475 | | 28 |
| A_32_P101689 | FAM3C | 28 |
| A_32_P107876 | FRAS1 | 28 |
| A_32_P112531 | | 28 |
| A_32_P115446 | | 28 |
| A_32_P117730 | | 28 |
| A_32_P119704 | | 28 |
| A_32_P121978 | | 28 |
| A_32_P131583 | | 28 |
| A_32_P137336 | | 28 |
| A_32_P146635 | | 28 |
| A_32_P158181 | | 28 |
| A_32_P158272 | MACC1 | 28 |
| A_32_P180435 | SPDYE1 | 28 |
| A_32_P183598 | | 28 |
| A_32_P184394 | TFEC | 28 |
| A_32_P194182 | | 28 |
| A_32_P201958 | FLVCR1 | 28 |
| A_32_P204239 | CDHR3 | 28 |
| A_32_P220762 | OSBPL6 | 28 |
| A_32_P227870 | SLC30A4 | 28 |
| A_32_P227921 | | 28 |
| A_32_P34138 | FAM25A | 28 |
| A_32_P40424 | | 28 |
| A_32_P66908 | CXXC4 | 28 |
| A_32_P72541 | | 28 |
| A_32_P879150 | | 28 |
| A_32_P9737 | | 28 |
| A_23_P11017 | | 29 |
| A_23_P117387 | MIA2 | 29 |
| A_23_P127789 | AHNAK | 29 |
| A_23_P146997 | CXORF15 | 29 |
| A_23_P147869 | KIAA1109 | 29 |
| A_23_P155197 | PTPLB | 29 |
| A_23_P203201 | DDX6 | 29 |
| A_23_P204810 | OSBPL8 | 29 |
| A_23_P205408 | MIA2 | 29 |
| A_23_P212179 | HRH1 | 29 |
| A_23_P218784 | DDX17 | 29 |
| A_23_P26358 | SMG1 | 29 |
| A_23_P300301 | JMJD1C | 29 |
| A_23_P339633 | CCDC75 | 29 |
| A_23_P351320 | CDC40 | 29 |
| A_23_P382302 | RIF1 | 29 |
| A_23_P382835 | P2RY1 | 29 |
| A_23_P383698 | LOC641298 | 29 |
| A_23_P390722 | TOR1AIP2 | 29 |
| A_23_P39137 | GRLF1 | 29 |
| A_23_P406131 | TMEM159 | 29 |
| A_23_P423074 | FAM169A | 29 |
| A_23_P42530 | DST | 29 |
| A_23_P425932 | VTI1A | 29 |
| A_23_P48387 | PDS5B | 29 |
| A_23_P7483 | BDP1 | 29 |
| A_23_P97795 | ACBD5 | 29 |
| A_24_P115287 | | 29 |
| A_24_P130865 | PHF8 | 29 |
| A_24_P136497 | DENND4A | 29 |
| A_24_P155761 | AKAP13 | 29 |
| A_24_P158946 | FGD4 | 29 |
| A_24_P162423 | | 29 |
| A_24_P171110 | HNRNPU | 29 |
| A_24_P178106 | YTHDC2 | 29 |
| A_24_P215407 | DDX6 | 29 |
| A_24_P238402 | ZNF644 | 29 |
| A_24_P256863 | THRAP3 | 29 |
| A_24_P26114 | OXSR1 | 29 |
| A_24_P264928 | BOD1L | 29 |
| A_24_P286412 | CRCP | 29 |
| A_24_P288979 | GNPTAB | 29 |
| A_24_P298224 | NCKAP5 | 29 |
| A_24_P302833 | APPL1 | 29 |
| A_24_P303052 | PPARGC1A | 29 |
| A_24_P346101 | PRELID2 | 29 |
| A_24_P362931 | SLC4A7 | 29 |
| A_24_P367666 | ASH1L | 29 |
| A_24_P402847 | CLCN3 | 29 |
| A_24_P410776 | DNAH7 | 29 |
| A_24_P50829 | TRPM7 | 29 |
| A_24_P529644 | H6PD | 29 |
| A_24_P60217 | SLC23A3 | 29 |
| A_24_P617818 | | 29 |
| A_24_P678741 | KIAA1671 | 29 |
| A_24_P678743 | KIAA1671 | 29 |
| A_24_P728115 | | 29 |
| A_24_P7565 | | 29 |
| A_24_P8098 | NSD1 | 29 |
| A_24_P816777 | LOC440288 | 29 |
| A_24_P84719 | | 29 |
| A_24_P889980 | PMS2L1 | 29 |
| A_24_P910490 | | 29 |
| A_24_P922858 | TMEM33 | 29 |
| A_24_P922969 | RALGAPA1 | 29 |
| A_24_P932016 | HUWE1 | 29 |
| A_24_P932308 | COQ9 | 29 |
| A_24_P933492 | ZDHHC21 | 29 |
| A_24_P93656 | CAND1 | 29 |
| A_24_P98948 | | 29 |
| A_32_P143516 | FAM178A | 29 |
| A_32_P156136 | NAA16 | 29 |
| A_32_P157228 | AHCTF1 | 29 |
| A_32_P157402 | SLC25A13 | 29 |
| A_32_P165297 | | 29 |
| A_32_P166480 | C9ORF93 | 29 |
| A_32_P182458 | | 29 |
| A_32_P185361 | | 29 |
| A_32_P215462 | | 29 |
| A_32_P231346 | | 29 |
| A_32_P233860 | YWHAZ | 29 |
| A_32_P309929 | CDK9 | 29 |
| A_32_P5040 | NOTCH2NL | 29 |
| A_32_P89087 | | 29 |
| A_23_P111811 | ING3 | 30 |
| A_23_P145424 | KIAA1009 | 30 |
| A_23_P151895 | CILP | 30 |
| A_23_P16806 | MGC10701 | 30 |
| A_23_P213166 | C4ORF21 | 30 |
| A_23_P216038 | PHF20L1 | 30 |
| A_23_P328621 | UBQLNL | 30 |
| A_23_P36865 | CEP290 | 30 |
| A_23_P381746 | ASXL3 | 30 |
| A_23_P405707 | BCOR | 30 |
| A_23_P89871 | ZNF415 | 30 |
| A_23_P90333 | ZNF404 | 30 |
| A_23_P96369 | CXORF57 | 30 |
| A_24_P23482 | CLYBL | 30 |
| A_24_P237389 | EIF1AX | 30 |
| A_24_P238525 | | 30 |
| A_24_P307665 | | 30 |
| A_24_P376139 | | 30 |
| A_24_P51118 | MTAP | 30 |
| A_24_P576219 | | 30 |
| A_24_P706983 | | 30 |
| A_24_P7974 | SLC26A6 | 30 |
| A_24_P916364 | STRBP | 30 |
| A_24_P916816 | SP100 | 30 |
| A_24_P928272 | | 30 |
| A_32_P107777 | MBD2 | 30 |
| A_32_P115277 | | 30 |
| A_32_P117186 | | 30 |
| A_32_P122579 | | 30 |
| A_32_P134290 | ZCCHC2 | 30 |
| A_32_P13991 | | 30 |
| A_32_P146844 | | 30 |
| A_32_P181271 | | 30 |
| A_32_P181826 | | 30 |
| A_32_P186921 | ZNF616 | 30 |
| A_32_P191735 | | 30 |
| A_32_P206549 | | 30 |
| A_32_P210038 | | 30 |
| A_32_P211141 | LYRM7 | 30 |
| A_32_P222060 | | 30 |
| A_32_P385 | | 30 |
| A_32_P40463 | NUDT9P1 | 30 |
| A_32_P48526 | | 30 |
| A_32_P486443 | ZNF500 | 30 |
| A_32_P517749 | RPS6KA3 | 30 |
| A_32_P61439 | | 30 |
| A_32_P73304 | | 30 |
| A_32_P80587 | | 30 |
| A_32_P81173 | USP34 | 30 |
| A_32_P81806 | LOC148203 | 30 |
| A_32_P87631 | | 30 |
| A_32_P90685 | | 30 |
| A_23_P112726 | SCN9A | 31 |
| A_23_P12514 | RHOC | 31 |
| A_23_P149946 | CDHR1 | 31 |
| A_23_P150018 | DUSP5 | 31 |
| A_23_P163238 | STRC | 31 |
| A_23_P167367 | PITX2 | 31 |
| A_23_P201551 | VAV3 | 31 |
| A_23_P20494 | NDRG1 | 31 |
| A_23_P213050 | HPGD | 31 |
| A_23_P214821 | EDN1 | 31 |
| A_23_P216556 | EPB41L4B | 31 |
| A_23_P217114 | ALAD | 31 |
| A_23_P250294 | ABHD5 | 31 |
| A_23_P258463 | PROM1 | 31 |
| A_23_P27795 | SPINT2 | 31 |
| A_23_P29257 | H1F0 | 31 |
| A_23_P305120 | ANKRD23 | 31 |
| A_23_P305759 | ABHD3 | 31 |
| A_23_P314805 | TMEM56 | 31 |
| A_23_P36187 | SYT8 | 31 |
| A_23_P370651 | FAM13A | 31 |
| A_23_P413923 | DMRTA1 | 31 |
| A_23_P47924 | PTPRR | 31 |
| A_23_P500501 | FGFR3 | 31 |
| A_23_P55448 | KRT12 | 31 |
| A_23_P59718 | SRI | 31 |
| A_23_P6433 | MB | 31 |
| A_23_P65629 | KCNK10 | 31 |
| A_23_P70566 | FKBPL | 31 |
| A_23_P71790 | MAMDC4 | 31 |
| A_23_P75973 | RNF121 | 31 |
| A_23_P85441 | IGSF9 | 31 |
| A_23_P9402 | CNTFR | 31 |
| A_24_P103004 | SLC20A1 | 31 |
| A_24_P1054 | NFKBIL2 | 31 |
| A_24_P106297 | AMACR | 31 |
| A_24_P151692 | POF1B | 31 |
| A_24_P167877 | LOC100132247 | 31 |
| A_24_P203696 | | 31 |
| A_24_P298495 | | 31 |
| A_24_P3005 | SCN9A | 31 |
| A_24_P316046 | LOC25845 | 31 |
| A_24_P419300 | LOC25845 | 31 |
| A_24_P488105 | ETNK1 | 31 |
| A_24_P912799 | SEMA5A | 31 |
| A_24_P930088 | LOC100286909 | 31 |
| A_24_P937029 | | 31 |
| A_32_P199998 | C10ORF75 | 31 |
| A_32_P217271 | | 31 |
| A_32_P230398 | | 31 |
| A_32_P34522 | | 31 |
| A_32_P49764 | LOC100292909 | 31 |
| A_32_P78285 | | 31 |
| A_23_P112957 | | 32 |
| A_23_P123596 | GLDC | 32 |
| A_23_P124632 | IGHA1 | 32 |
| A_23_P136026 | IGHA1 | 32 |
| A_23_P158868 | | 32 |
| A_23_P159163 | | 32 |
| A_23_P159435 | | 32 |
| A_23_P167168 | IGJ | 32 |
| A_23_P170830 | | 32 |
| A_23_P21249 | | 32 |
| A_23_P21260 | | 32 |
| A_23_P21800 | LOC440871 | 32 |
| A_23_P259763 | | 32 |
| A_23_P350782 | | 32 |
| A_23_P361654 | IGKC | 32 |
| A_23_P390206 | | 32 |
| A_23_P435390 | | 32 |
| A_23_P44053 | | 32 |
| A_23_P61042 | | 32 |
| A_23_P61068 | | 32 |
| A_23_P6362 | DERL3 | 32 |
| A_23_P73328 | | 32 |
| A_23_P84596 | MGC29506 | 32 |
| A_23_P84791 | | 32 |
| A_23_P96191 | | 32 |
| A_23_P9997 | | 32 |
| A_24_P100684 | | 32 |
| A_24_P101226 | | 32 |
| A_24_P101642 | LOC401847 | 32 |
| A_24_P110242 | | 32 |
| A_24_P110487 | | 32 |
| A_24_P144346 | LOC100289290 | 32 |
| A_24_P15388 | LOC100291190 | 32 |
| A_24_P15550 | | 32 |
| A_24_P16004 | | 32 |
| A_24_P161853 | LOC100132941 | 32 |
| A_24_P169713 | | 32 |
| A_24_P179107 | | 32 |
| A_24_P203886 | | 32 |
| A_24_P204374 | | 32 |
| A_24_P204574 | | 32 |
| A_24_P204727 | | 32 |
| A_24_P212024 | LOC100291464 | 32 |
| A_24_P229447 | | 32 |
| A_24_P239076 | IGLL1 | 32 |
| A_24_P24053 | | 32 |
| A_24_P241996 | | 32 |
| A_24_P263786 | | 32 |
| A_24_P272146 | IGKC | 32 |
| A_24_P298805 | | 32 |
| A_24_P306905 | IGKV2-24 | 32 |
| A_24_P307375 | | 32 |
| A_24_P315854 | LOC100292858 | 32 |
| A_24_P315941 | | 32 |
| A_24_P318990 | | 32 |
| A_24_P323298 | | 32 |
| A_24_P33341 | | 32 |
| A_24_P341126 | | 32 |
| A_24_P357847 | LOC440871 | 32 |
| A_24_P358321 | | 32 |
| A_24_P361816 | | 32 |
| A_24_P384119 | LOC100287372 | 32 |
| A_24_P384604 | | 32 |
| A_24_P413286 | | 32 |
| A_24_P417352 | IGHM | 32 |
| A_24_P465799 | | 32 |
| A_24_P472081 | | 32 |
| A_24_P484904 | | 32 |
| A_24_P488083 | | 32 |
| A_24_P490109 | | 32 |
| A_24_P494425 | | 32 |
| A_24_P510357 | LOC100293440 | 32 |
| A_24_P519504 | | 32 |
| A_24_P538459 | | 32 |
| A_24_P590547 | LOC652494 | 32 |
| A_24_P604784 | | 32 |
| A_24_P605563 | LOC100290481 | 32 |
| A_24_P608268 | | 32 |
| A_24_P626951 | | 32 |
| A_24_P639701 | | 32 |
| A_24_P66578 | LOC100287723 | 32 |
| A_24_P677559 | LOC100291682 | 32 |
| A_24_P702749 | | 32 |
| A_24_P750327 | | 32 |
| A_24_P76868 | | 32 |
| A_24_P813550 | LOC100292858 | 32 |
| A_24_P83102 | IGLL1 | 32 |
| A_24_P852001 | | 32 |
| A_24_P889462 | LOC652494 | 32 |
| A_24_P917316 | | 32 |
| A_24_P917492 | TTLL3 | 32 |
| A_24_P92683 | LOC646057 | 32 |
| A_24_P93523 | IGKV1D-8 | 32 |
| A_32_P132194 | | 32 |
| A_32_P148118 | LOC642838 | 32 |
| A_32_P148122 | LOC642838 | 32 |
| A_32_P157927 | | 32 |
| A_32_P159192 | LOC440871 | 32 |
| A_32_P39440 | LOC440871 | 32 |
| A_32_P43664 | | 32 |
| A_32_P51988 | LOC100290115 | 32 |
| A_32_P65022 | | 32 |
| A_32_P722809 | IGKV1-5 | 32 |
| A_32_P76137 | | 32 |
| A_23_P115022 | TMEM125 | 33 |
| A_23_P131825 | TNNC2 | 33 |
| A_23_P133216 | NLN | 33 |
| A_23_P140405 | FOXN3 | 33 |
| A_23_P163251 | PAQR5 | 33 |
| A_23_P164912 | LIN7B | 33 |
| A_23_P2041 | MICALCL | 33 |
| A_23_P301530 | ANK3 | 33 |
| A_23_P333218 | ERGIC1 | 33 |
| A_23_P33583 | DNAH7 | 33 |
| A_23_P427014 | CLDN8 | 33 |
| A_23_P49145 | ZG16 | 33 |
| A_23_P66948 | FAM59A | 33 |
| A_23_P69531 | KLB | 33 |
| A_23_P87049 | SORL1 | 33 |
| A_23_P97173 | HSD3B1 | 33 |
| A_23_P99253 | LIN7A | 33 |
| A_24_P115621 | EIF4EBP2 | 33 |
| A_24_P136471 | SLC14A2 | 33 |
| A_24_P366777 | NOTCH2NL | 33 |
| A_24_P42693 | CYP4F11 | 33 |
| A_24_P867201 | | 33 |
| A_24_P943393 | AHNAK | 33 |
| A_24_P944331 | PAQR5 | 33 |
| A_32_P122715 | | 33 |
| A_32_P13168 | | 33 |
| A_32_P223504 | | 33 |
| A_32_P34920 | FOXD1 | 33 |
| A_23_P115246 | FCN3 | 34 |
| A_23_P117602 | GZMB | 34 |
| A_23_P137097 | SLC16A2 | 34 |
| A_23_P13753 | NFE2 | 34 |
| A_23_P137856 | MUC1 | 34 |
| A_23_P150316 | MMP12 | 34 |
| A_23_P151851 | DUOX2 | 34 |
| A_23_P156687 | CFB | 34 |
| A_23_P157628 | DEFB4A | 34 |
| A_23_P157875 | FCN1 | 34 |
| A_23_P160920 | PDZK1IP1 | 34 |
| A_23_P161698 | MMP3 | 34 |
| A_23_P166109 | FLRT3 | 34 |
| A_23_P166408 | OSM | 34 |
| A_23_P1691 | MMP1 | 34 |
| A_23_P169437 | LCN2 | 34 |
| A_23_P17065 | CCL20 | 34 |
| A_23_P200728 | FCGR3A | 34 |
| A_23_P209995 | IL1RN | 34 |
| A_23_P214267 | GPR110 | 34 |
| A_23_P23048 | S100A9 | 34 |
| A_23_P256158 | ADRA2C | 34 |
| A_23_P306203 | SAA2 | 34 |
| A_23_P315364 | CXCL2 | 34 |
| A_23_P330561 | C19ORF59 | 34 |
| A_23_P340698 | MMP12 | 34 |
| A_23_P373017 | CCL3 | 34 |
| A_23_P38795 | FPR1 | 34 |
| A_23_P394304 | PDZK1IP1 | 34 |
| A_23_P40453 | CBR3 | 34 |
| A_23_P411296 | CEBPB | 34 |
| A_23_P434809 | S100A8 | 34 |
| A_23_P45751 | CLCA4 | 34 |
| A_23_P502464 | NOS2 | 34 |
| A_23_P503072 | CCL28 | 34 |
| A_23_P60627 | ALOX15B | 34 |
| A_23_P63390 | FCGR1B | 34 |
| A_23_P64721 | GPR109B | 34 |
| A_23_P74001 | S100A12 | 34 |
| A_23_P79518 | IL1B | 34 |
| A_23_P82651 | NPTX2 | 34 |
| A_23_P86599 | DMBT1 | 34 |
| A_23_P92860 | CCNO | 34 |
| A_24_P183150 | CXCL3 | 34 |
| A_24_P257416 | CXCL2 | 34 |
| A_24_P277367 | CXCL5 | 34 |
| A_24_P335092 | SAA1 | 34 |
| A_24_P70480 | CEACAM4 | 34 |
| A_24_P764690 | ZNF720 | 34 |
| A_24_P78531 | CLEC4E | 34 |
| A_24_P788878 | C2CD4B | 34 |
| A_32_P164916 | | 34 |
| A_32_P164917 | | 34 |
| A_32_P214011 | | 34 |
| A_32_P70158 | LILRB3 | 34 |
| A_32_P74942 | | 34 |
| A_23_P115902 | | 35 |
| A_23_P118135 | | 35 |
| A_23_P11922 | | 35 |
| A_23_P120973 | FAM118A | 35 |
| A_23_P124252 | CAMK1D | 35 |
| A_23_P161644 | RBM14 | 35 |
| A_23_P167121 | GABRA2 | 35 |
| A_23_P207476 | SPAG9 | 35 |
| A_23_P211643 | PPARA | 35 |
| A_23_P212869 | GABRA2 | 35 |
| A_23_P2293 | HELB | 35 |
| A_23_P2294 | HELB | 35 |
| A_23_P23234 | MGC5457 | 35 |
| A_23_P2901 | | 35 |
| A_23_P343237 | ANGEL2 | 35 |
| A_23_P346982 | DTWD2 | 35 |
| A_23_P348281 | C1ORF83 | 35 |
| A_23_P350895 | RABGAP1 | 35 |
| A_23_P351695 | SYNRG | 35 |
| A_23_P365119 | CCDC57 | 35 |
| A_23_P37317 | LOC100293413 | 35 |
| A_23_P387691 | DIP2B | 35 |
| A_23_P390621 | PACRGL | 35 |
| A_23_P400459 | RG9MTD3 | 35 |
| A_23_P402164 | LOC142937 | 35 |
| A_23_P403195 | MICAL3 | 35 |
| A_23_P412059 | ZRANB3 | 35 |
| A_23_P418477 | AKAP11 | 35 |
| A_23_P42036 | LYRM2 | 35 |
| A_23_P42042 | LYRM2 | 35 |
| A_23_P430051 | NGLY1 | 35 |
| A_23_P48416 | DIS3 | 35 |
| A_23_P49686 | | 35 |
| A_23_P55616 | SLC14A1 | 35 |
| A_23_P63057 | GON4L | 35 |
| A_23_P67312 | ZNF136 | 35 |
| A_23_P77468 | | 35 |
| A_23_P84272 | | 35 |
| A_23_P8754 | AASS | 35 |
| A_23_P88021 | GPR180 | 35 |
| A_23_P93818 | STAG3L1 | 35 |
| A_23_P9662 | IPP | 35 |
| A_23_P97517 | RALGPS2 | 35 |
| A_24_P111019 | | 35 |
| A_24_P127701 | LOC651856 | 35 |
| A_24_P136711 | | 35 |
| A_24_P145107 | | 35 |
| A_24_P145333 | | 35 |
| A_24_P162128 | | 35 |
| A_24_P176404 | ZFP90 | 35 |
| A_24_P176805 | ZNF264 | 35 |
| A_24_P186030 | PRKY | 35 |
| A_24_P187826 | PPIL4 | 35 |
| A_24_P194661 | FLJ38379 | 35 |
| A_24_P201381 | FGFBP3 | 35 |
| A_24_P239309 | | 35 |
| A_24_P24565 | | 35 |
| A_24_P253755 | PIGL | 35 |
| A_24_P268474 | | 35 |
| A_24_P27373 | PLDN | 35 |
| A_24_P287403 | MPHOSPH8 | 35 |
| A_24_P290609 | PDLIM2 | 35 |
| A_24_P29665 | CYCS | 35 |
| A_24_P305556 | MCM8 | 35 |
| A_24_P307854 | FBXL18 | 35 |
| A_24_P307974 | TAF8 | 35 |
| A_24_P310224 | ZNF347 | 35 |
| A_24_P316495 | | 35 |
| A_24_P321715 | | 35 |
| A_24_P359165 | SWAP70 | 35 |
| A_24_P378302 | C15ORF28 | 35 |
| A_24_P381379 | | 35 |
| A_24_P387321 | ZNF44 | 35 |
| A_24_P408341 | CRYZL1 | 35 |
| A_24_P418998 | | 35 |
| A_24_P419017 | | 35 |
| A_24_P453921 | LOC100129460 | 35 |
| A_24_P481534 | | 35 |
| A_24_P51279 | | 35 |
| A_24_P51375 | | 35 |
| A_24_P535380 | | 35 |
| A_24_P537188 | | 35 |
| A_24_P570454 | | 35 |
| A_24_P57170 | C19ORF55 | 35 |
| A_24_P593120 | | 35 |
| A_24_P59387 | C8ORF60 | 35 |
| A_24_P607195 | | 35 |
| A_24_P649829 | | 35 |
| A_24_P65098 | TMEM87A | 35 |
| A_24_P66337 | LCLAT1 | 35 |
| A_24_P670342 | | 35 |
| A_24_P697437 | | 35 |
| A_24_P718672 | | 35 |
| A_24_P722292 | | 35 |
| A_24_P732033 | | 35 |
| A_24_P737553 | | 35 |
| A_24_P744554 | | 35 |
| A_24_P799680 | | 35 |
| A_24_P829934 | | 35 |
| A_24_P830025 | FNTA | 35 |
| A_24_P8349 | ZFYVE16 | 35 |
| A_24_P855152 | | 35 |
| A_24_P856722 | | 35 |
| A_24_P876772 | | 35 |
| A_24_P88493 | | 35 |
| A_24_P910030 | LOC100289550 | 35 |
| A_24_P912372 | KIAA0754 | 35 |
| A_24_P921343 | SFRS2IP | 35 |
| A_24_P931974 | | 35 |
| A_24_P936171 | AGMAT | 35 |
| A_24_P937582 | | 35 |
| A_24_P941167 | APOL6 | 35 |
| A_24_P944063 | ZNF785 | 35 |
| A_24_P945165 | ACOT11 | 35 |
| A_32_P101002 | | 35 |
| A_32_P108226 | | 35 |
| A_32_P110086 | | 35 |
| A_32_P110178 | | 35 |
| A_32_P110433 | CYP20A1 | 35 |
| A_32_P113404 | | 35 |
| A_32_P116088 | | 35 |
| A_32_P118010 | | 35 |
| A_32_P118013 | | 35 |
| A_32_P127412 | | 35 |
| A_32_P129310 | | 35 |
| A_32_P132883 | | 35 |
| A_32_P134090 | | 35 |
| A_32_P139196 | MIR17HG | 35 |
| A_32_P140228 | | 35 |
| A_32_P1434 | | 35 |
| A_32_P146898 | | 35 |
| A_32_P150130 | PLEKHA2 | 35 |
| A_32_P1647 | | 35 |
| A_32_P179131 | | 35 |
| A_32_P181548 | | 35 |
| A_32_P185398 | | 35 |
| A_32_P190682 | | 35 |
| A_32_P194891 | | 35 |
| A_32_P195788 | | 35 |
| A_32_P197976 | | 35 |
| A_32_P203292 | | 35 |
| A_32_P203615 | | 35 |
| A_32_P206561 | | 35 |
| A_32_P206989 | | 35 |
| A_32_P209472 | MLL3 | 35 |
| A_32_P209909 | | 35 |
| A_32_P21255 | SLC30A4 | 35 |
| A_32_P215143 | LOC100288583 | 35 |
| A_32_P217051 | | 35 |
| A_32_P220161 | | 35 |
| A_32_P220233 | | 35 |
| A_32_P222872 | | 35 |
| A_32_P225472 | LOC389834 | 35 |
| A_32_P23096 | | 35 |
| A_32_P23209 | | 35 |
| A_32_P233713 | | 35 |
| A_32_P26738 | KIAA1143 | 35 |
| A_32_P27698 | SEPT14 | 35 |
| A_32_P28712 | | 35 |
| A_32_P30238 | | 35 |
| A_32_P30831 | | 35 |
| A_32_P32496 | FLJ44253 | 35 |
| A_32_P34167 | | 35 |
| A_32_P37143 | GAS2L3 | 35 |
| A_32_P38436 | | 35 |
| A_32_P39963 | EXOSC6 | 35 |
| A_32_P44808 | | 35 |
| A_32_P51688 | | 35 |
| A_32_P53884 | KIF1C | 35 |
| A_32_P53976 | | 35 |
| A_32_P55414 | | 35 |
| A_32_P5673 | FABP3 | 35 |
| A_32_P65303 | | 35 |
| A_32_P67447 | | 35 |
| A_32_P67577 | | 35 |
| A_32_P69475 | LNPEP | 35 |
| A_32_P71310 | | 35 |
| A_32_P72110 | PVR | 35 |
| A_32_P72822 | CCNB2 | 35 |
| A_32_P7308 | | 35 |
| A_32_P73580 | | 35 |
| A_32_P76576 | MALT1 | 35 |
| A_32_P83520 | | 35 |
| A_32_P89277 | | 35 |
| A_32_P97373 | | 35 |
| A_23_P124934 | IKZF1 | 36 |
| A_23_P139687 | ERP27 | 36 |
| A_23_P157495 | PPP3CC | 36 |
| A_23_P167017 | POPDC2 | 36 |
| A_23_P209347 | ANKRD44 | 36 |
| A_23_P218858 | ABI3BP | 36 |
| A_23_P255166 | ARHGAP15 | 36 |
| A_23_P302018 | TXK | 36 |
| A_23_P335920 | RPS6KA2 | 36 |
| A_23_P35916 | ATM | 36 |
| A_23_P35995 | ASAM | 36 |
| A_23_P378555 | PARP15 | 36 |
| A_23_P431252 | KBTBD8 | 36 |
| A_23_P45087 | ZNF107 | 36 |
| A_23_P64898 | KLRG1 | 36 |
| A_23_P75848 | GVIN1 | 36 |
| A_23_P82181 | QRSL1 | 36 |
| A_24_P109554 | CEP110 | 36 |
| A_24_P163477 | QRSL1 | 36 |
| A_24_P190007 | GP2 | 36 |
| A_24_P190631 | IKZF1 | 36 |
| A_24_P196878 | | 36 |
| A_24_P241183 | CLEC2D | 36 |
| A_24_P256674 | ARHGEF10 | 36 |
| A_24_P289260 | DACT2 | 36 |
| A_24_P291278 | LAX1 | 36 |
| A_24_P316234 | LOC643475 | 36 |
| A_24_P33982 | C17ORF60 | 36 |
| A_24_P360206 | PCDHA11 | 36 |
| A_24_P360529 | PDE7A | 36 |
| A_24_P374741 | FRMD4A | 36 |
| A_24_P477549 | LOC100289399 | 36 |
| A_24_P485105 | | 36 |
| A_24_P56887 | CRLF3 | 36 |
| A_24_P586264 | PPM1K | 36 |
| A_24_P608007 | | 36 |
| A_24_P76158 | DOCK11 | 36 |
| A_24_P769977 | | 36 |
| A_24_P772276 | LOC100292160 | 36 |
| A_24_P795215 | | 36 |
| A_24_P890536 | | 36 |
| A_24_P899020 | | 36 |
| A_24_P935026 | STK4 | 36 |
| A_24_P935330 | PRKCB | 36 |
| A_24_P943566 | PHACTR1 | 36 |
| A_24_P945262 | CARD11 | 36 |
| A_32_P118325 | | 36 |
| A_32_P119604 | | 36 |
| A_32_P138409 | | 36 |
| A_32_P144390 | | 36 |
| A_32_P152986 | | 36 |
| A_32_P162095 | | 36 |
| A_32_P163594 | | 36 |
| A_32_P180185 | | 36 |
| A_32_P219704 | | 36 |
| A_32_P30345 | | 36 |
| A_32_P37584 | | 36 |
| A_32_P406186 | | 36 |
| A_32_P47200 | | 36 |
| A_32_P92117 | | 36 |
| A_32_P96719 | SHCBP1 | 36 |
| A_23_P125107 | HLA-B | 37 |
| A_23_P125109 | | 37 |
| A_23_P133916 | C2 | 37 |
| A_23_P145264 | HLA-F | 37 |
| A_23_P145336 | LOC100294275 | 37 |
| A_23_P17837 | APOL1 | 37 |
| A_23_P250629 | PSMB8 | 37 |
| A_23_P259292 | C1QTNF5 | 37 |
| A_23_P31006 | HLA-DRB5 | 37 |
| A_23_P314024 | | 37 |
| A_23_P338113 | | 37 |
| A_23_P370707 | | 37 |
| A_23_P373126 | | 37 |
| A_23_P380857 | APOL4 | 37 |
| A_23_P408353 | HLA-A | 37 |
| A_23_P95917 | HLA-C | 37 |
| A_24_P326082 | HLA-E | 37 |
| A_24_P343233 | HLA-DRB1 | 37 |
| A_24_P370472 | HLA-DRB4 | 37 |
| A_24_P402222 | HLA-DRB3 | 37 |
| A_32_P162183 | C2 | 37 |
| A_32_P162187 | C2 | 37 |
| A_32_P205624 | SHC2 | 37 |
| A_23_P125748 | ZMAT1 | 38 |
| A_23_P150746 | DKFZP547G183 | 38 |
| A_23_P218584 | BCL11A | 38 |
| A_23_P30163 | FLJ13197 | 38 |
| A_23_P350879 | PRO2852 | 38 |
| A_23_P37111 | DICER1 | 38 |
| A_23_P375566 | STXBP4 | 38 |
| A_23_P404211 | FANCM | 38 |
| A_23_P40896 | SLC25A36 | 38 |
| A_23_P51082 | | 38 |
| A_23_P97123 | MGC12538 | 38 |
| A_24_P107918 | CBFB | 38 |
| A_24_P119381 | | 38 |
| A_24_P153576 | SHPRH | 38 |
| A_24_P188056 | PARP11 | 38 |
| A_24_P247910 | BDP1 | 38 |
| A_24_P461998 | | 38 |
| A_24_P497235 | | 38 |
| A_24_P50818 | GTF3C3 | 38 |
| A_24_P513669 | | 38 |
| A_24_P529786 | | 38 |
| A_24_P629883 | | 38 |
| A_24_P665513 | | 38 |
| A_24_P675731 | | 38 |
| A_24_P685241 | RFX3 | 38 |
| A_24_P693820 | | 38 |
| A_24_P787914 | | 38 |
| A_24_P813730 | | 38 |
| A_24_P915300 | SYDE2 | 38 |
| A_24_P918137 | | 38 |
| A_24_P920904 | C8ORF59 | 38 |
| A_24_P925211 | | 38 |
| A_24_P936393 | | 38 |
| A_24_P941708 | RUFY2 | 38 |
| A_24_P943283 | DENND1B | 38 |
| A_32_P105110 | | 38 |
| A_32_P110820 | | 38 |
| A_32_P111266 | | 38 |
| A_32_P121855 | | 38 |
| A_32_P123106 | PAPOLG | 38 |
| A_32_P128974 | | 38 |
| A_32_P131342 | | 38 |
| A_32_P133038 | | 38 |
| A_32_P142943 | | 38 |
| A_32_P144007 | | 38 |
| A_32_P167883 | | 38 |
| A_32_P174258 | | 38 |
| A_32_P185766 | | 38 |
| A_32_P195346 | | 38 |
| A_32_P19561 | | 38 |
| A_32_P198620 | | 38 |
| A_32_P203219 | MAP4K5 | 38 |
| A_32_P204565 | | 38 |
| A_32_P206391 | | 38 |
| A_32_P208039 | | 38 |
| A_32_P211048 | | 38 |
| A_32_P212802 | LOC442572 | 38 |
| A_32_P213615 | | 38 |
| A_32_P216015 | | 38 |
| A_32_P216122 | | 38 |
| A_32_P221641 | | 38 |
| A_32_P230537 | | 38 |
| A_32_P24295 | | 38 |
| A_32_P29442 | | 38 |
| A_32_P31827 | | 38 |
| A_32_P33213 | | 38 |
| A_32_P3342 | | 38 |
| A_32_P36835 | | 38 |
| A_32_P40145 | | 38 |
| A_32_P41153 | | 38 |
| A_32_P46495 | | 38 |
| A_32_P51707 | | 38 |
| A_32_P81324 | | 38 |
| A_32_P95015 | | 38 |
| A_32_P9931 | | 38 |
| A_23_P12620 | TNKS2 | 39 |
| A_23_P14708 | ZNF280D | 39 |
| A_23_P16006 | ZNF600 | 39 |
| A_23_P217098 | VPS13A | 39 |
| A_23_P256391 | GOLGA4 | 39 |
| A_23_P258037 | KDM3A | 39 |
| A_23_P309207 | ZNF577 | 39 |
| A_23_P324718 | SYNJ1 | 39 |
| A_23_P325887 | TBC1D8B | 39 |
| A_23_P327361 | DMXL2 | 39 |
| A_23_P397347 | MCM9 | 39 |
| A_23_P434929 | CEP120 | 39 |
| A_23_P78018 | ABCA5 | 39 |
| A_23_P81721 | SLC25A27 | 39 |
| A_23_P89422 | ABCA10 | 39 |
| A_23_P90542 | ZNF540 | 39 |
| A_24_P108863 | SCML1 | 39 |
| A_24_P141629 | FAM111A | 39 |
| A_24_P187706 | GOLGA4 | 39 |
| A_24_P194313 | C21ORF66 | 39 |
| A_24_P226198 | RAD50 | 39 |
| A_24_P226210 | CEP120 | 39 |
| A_24_P263144 | BMX | 39 |
| A_24_P290999 | NEAT1 | 39 |
| A_24_P303080 | MFSD8 | 39 |
| A_24_P366107 | DNA2 | 39 |
| A_24_P380679 | C7ORF53 | 39 |
| A_24_P538708 | | 39 |
| A_24_P566916 | NEAT1 | 39 |
| A_24_P566968 | | 39 |
| A_24_P82135 | SECISBP2L | 39 |
| A_24_P940725 | SFRS18 | 39 |
| A_24_P941787 | PRPF4B | 39 |
| A_32_P105397 | | 39 |
| A_32_P144326 | | 39 |
| A_32_P167705 | AGBL2 | 39 |
| A_32_P169679 | LCORL | 39 |
| A_32_P177040 | SPDYE5 | 39 |
| A_32_P190944 | ACVR1C | 39 |
| A_32_P198029 | | 39 |
| A_32_P219116 | CENPJ | 39 |
| A_32_P227648 | | 39 |
| A_32_P38228 | | 39 |
| A_32_P64668 | | 39 |
| A_32_P69783 | | 39 |
| A_23_P130764 | KCNJ14 | 40 |
| A_23_P139500 | BHLHE41 | 40 |
| A_23_P152992 | TMC8 | 40 |
| A_23_P204640 | NANOG | 40 |
| A_23_P339079 | ZNF573 | 40 |
| A_23_P346265 | GNPTAB | 40 |
| A_23_P347632 | MTSS1 | 40 |
| A_23_P359174 | LOC100130401 | 40 |
| A_23_P371410 | PRKACB | 40 |
| A_23_P373716 | ZNF563 | 40 |
| A_23_P3921 | FLJ11710 | 40 |
| A_23_P413634 | ZNF329 | 40 |
| A_23_P50320 | | 40 |
| A_23_P52846 | | 40 |
| A_23_P57393 | | 40 |
| A_23_P6561 | FLJ10213 | 40 |
| A_23_P66190 | VAC14 | 40 |
| A_24_P224998 | | 40 |
| A_24_P347447 | DAAM1 | 40 |
| A_24_P50972 | LOC100132727 | 40 |
| A_24_P592060 | | 40 |
| A_24_P606239 | | 40 |
| A_24_P662427 | | 40 |
| A_24_P665185 | | 40 |
| A_24_P67898 | MGEA5 | 40 |
| A_24_P686956 | | 40 |
| A_24_P693433 | | 40 |
| A_24_P85418 | | 40 |
| A_24_P911960 | | 40 |
| A_24_P923922 | RUNDC2B | 40 |
| A_24_P924697 | CDC42EP3 | 40 |
| A_24_P930391 | | 40 |
| A_32_P106315 | | 40 |
| A_32_P108554 | | 40 |
| A_32_P118220 | | 40 |
| A_32_P124392 | | 40 |
| A_32_P137819 | | 40 |
| A_32_P140475 | KIAA1377 | 40 |
| A_32_P142149 | | 40 |
| A_32_P146826 | | 40 |
| A_32_P156062 | | 40 |
| A_32_P158723 | | 40 |
| A_32_P159176 | | 40 |
| A_32_P164573 | | 40 |
| A_32_P16462 | | 40 |
| A_32_P179646 | | 40 |
| A_32_P179807 | | 40 |
| A_32_P185701 | | 40 |
| A_32_P187304 | | 40 |
| A_32_P191373 | | 40 |
| A_32_P19193 | | 40 |
| A_32_P193792 | | 40 |
| A_32_P198282 | | 40 |
| A_32_P20203 | | 40 |
| A_32_P209582 | UNQ6228 | 40 |
| A_32_P209770 | | 40 |
| A_32_P220523 | | 40 |
| A_32_P232704 | MTERF | 40 |
| A_32_P233834 | | 40 |
| A_32_P25243 | | 40 |
| A_32_P43878 | | 40 |
| A_32_P4581 | | 40 |
| A_32_P48615 | LOC100128108 | 40 |
| A_32_P53234 | | 40 |
| A_32_P55427 | | 40 |
| A_32_P58912 | | 40 |
| A_32_P6452 | | 40 |
| A_32_P67533 | L3MBTL3 | 40 |
| A_32_P71016 | | 40 |
| A_32_P73071 | | 40 |
| A_32_P88987 | | 40 |
| A_32_P95573 | | 40 |
| A_23_P137238 | KDM5D | 41 |
| A_23_P203558 | HBB | 41 |
| A_23_P259314 | RPS4Y1 | 41 |
| A_23_P26457 | HBA2 | 41 |
| A_23_P31816 | DEFA3 | 41 |
| A_23_P324384 | RPS4Y2 | 41 |
| A_23_P37856 | HBA1 | 41 |
| A_24_P142305 | HBA2 | 41 |
| A_24_P147849 | | 41 |
| A_24_P75190 | HBD | 41 |
| A_32_P47166 | | 41 |
| A_23_P142724 | RPL37A | 42 |
| A_23_P143906 | MLF1 | 42 |
| A_23_P216396 | EXOSC2 | 42 |
| A_23_P26713 | RPL23 | 42 |
| A_23_P34018 | RPL39 | 42 |
| A_23_P366376 | TDGF1 | 42 |
| A_23_P41917 | HOMER1 | 42 |
| A_23_P44956 | RPL35A | 42 |
| A_23_P53476 | LDHB | 42 |
| A_23_P8640 | GPER | 42 |
| A_23_P86838 | SLC36A4 | 42 |
| A_24_P105794 | RPL31 | 42 |
| A_24_P145787 | | 42 |
| A_24_P161463 | C20ORF199 | 42 |
| A_24_P16503 | CNPY3 | 42 |
| A_24_P392230 | | 42 |
| A_24_P40010 | RPS28 | 42 |
| A_24_P409681 | | 42 |
| A_24_P503492 | | 42 |
| A_24_P516728 | | 42 |
| A_24_P524452 | | 42 |
| A_24_P576445 | TBC1D8 | 42 |
| A_24_P7181 | | 42 |
| A_24_P754989 | | 42 |
| A_24_P845082 | | 42 |
| A_24_P881430 | | 42 |
| A_24_P924185 | | 42 |
| A_24_P93397 | LOC100288233 | 42 |
| A_32_P110243 | LOC729927 | 42 |
| A_32_P111658 | | 42 |
| A_32_P128399 | | 42 |
| A_32_P132337 | | 42 |
| A_32_P135601 | | 42 |
| A_32_P140268 | KCND3 | 42 |
| A_32_P145856 | | 42 |
| A_32_P147790 | | 42 |
| A_32_P151823 | LOC346887 | 42 |
| A_32_P153456 | | 42 |
| A_32_P158053 | | 42 |
| A_32_P160398 | | 42 |
| A_32_P161166 | | 42 |
| A_32_P171695 | CC2D2B | 42 |
| A_32_P191541 | | 42 |
| A_32_P202588 | | 42 |
| A_32_P205478 | | 42 |
| A_32_P218251 | | 42 |
| A_32_P220307 | RPL39 | 42 |
| A_32_P225129 | | 42 |
| A_32_P226768 | | 42 |
| A_32_P229365 | | 42 |
| A_32_P29395 | | 42 |
| A_32_P42104 | | 42 |
| A_32_P51781 | | 42 |
| A_32_P54260 | | 42 |
| A_32_P64263 | | 42 |
| A_32_P75867 | | 42 |
| A_32_P80016 | | 42 |
| A_32_P8857 | | 42 |
| A_32_P92814 | | 42 |
| A_32_P98348 | ZNF525 | 42 |
| A_23_P212968 | UGT2B11 | 43 |
| A_23_P501624 | UGT2B17 | 43 |
| A_23_P58407 | UGT2B15 | 43 |
| A_23_P7342 | UGT2B10 | 43 |
| A_24_P17691 | UGT2B17 | 43 |
| A_24_P521559 | UGT2B10 | 43 |
| A_24_P575267 | | 43 |
