# Supplementary material for: Host Genes Related to Paneth Cells and Xenobiotic Metabolism Are Associated with Shifts in Human Ileum-Associated Microbial Composition
Source: PLoS One. 2012 Jun 13;7(6):e30044. doi: 10.1371/journal.pone.0030044 (PMC3374611; doi:10.1371/journal.pone.0030044)
Supplement: Table S3 — Clusters obtained by after dimension reduction using SAM. The clusters are listed along with the percentage of variation explained by the 1st PC of the cluster, which is related to the compactness of the cluster. The bolded clusters are the clusters in which >40% of the gene-probes were concentrated in two of the 265 clusters obtained without prior dimension reduction. The clusters were considered enriched for genes that were differentially expressed in CD vs. Control, UC vs. Control or CD vs. UC if ≥50% of the genes with a correlation of ≥0.75% to the 1st PC of the cluster demonstrated a significant fold change (see Supplementary Table 1). Clusters were considered enriched for genes in an IPA canonical pathway if P<0.01 and at least 4 genes were in the pathway. In addition we listed our interpretation of the biological significance of the pathway. (DOCX) [file pone.0030044.s003.docx]

**Supplementary Table S3. Clusters obtained by after dimension reduction using SAM.** The clusters are listed along with the percentage of variation explained by the 1^st^ PC of the cluster, which is related to the compactness of the cluster. The bolded clusters are the clusters in which >40% of the gene-probes were concentrated in two of the 265 clusters obtained without prior dimension reduction. The clusters were considered enriched for genes that were differentially expressed in CD vs. Control, UC vs. Control or CD vs. UC if ≥ 50% of the genes with a correlation of ≥ 0.75% to the 1^st^ PC of the cluster demonstrated a significant fold change (see Supplementary Table 1). Clusters were considered enriched for genes in an IPA canonical pathway if P <0.01 and at least 4 genes were in the pathway. In addition we listed our interpretation of the biological significance of the pathway.

| Cluster/  (% 1^st^ PC) | CD vs. Control | UC vs. Control | CD vs. UC | Biological significance  IPA canonical pathways (P-value)  **^*^Assigned by authors** |
| --- | --- | --- | --- | --- |
| **1/ (68.4%)** | ↓ |  | ↓ | Altered T and B Cell Signaling in Rheumatoid Arthritis (6.9E-4)  B Cell Receptor Signaling (9.7E-3)  ***Formation of organized lymphoid structures** |
| **2/ (67.7%)** |  | ↓ |  | LPS/IL-1 Mediated Inhibition of RXR Function (8.9E-4)  Arachidonic Acid Metabolism (2.2E-3)  Xenobiotic Metabolism Signaling (4.4E-3)  ***Nuclear receptor signaling** |
| **3/ (70.3%)** |  |  | ↓ |  |
| **4/ (78.6%)** |  |  | ↑ | CDK5 Signaling (2.2E-4)  ILK Signaling (2.7E-3)  ERK/MAPK Signaling (2.9E-3)  Integrin Signaling (3.7E-3) |
| **5/ (67.6%)** |  | ↓ | ↑ |  |
| **6/ (73.1%)** |  |  | ↑ | Antigen Presentation Pathway (1.5E-5)  Galactose Metabolism (8.0E-5)  Allograft Rejection Signaling (2.8E-4)  Glycerolipid Metabolism (2.9E-4)  Autoimmune Thyroid Disease Signaling (3.5E-4)  Type I Diabetes Mellitus Signaling (4.5E-4)  Graft-versus-Host Disease Signaling (4.9E-4)  Neuroprotective Role of THOP1 in Alzheimer’s Disease (5.9E-4)  Pyruvate Metabolism (6.3E-4)  Glycerophospholipid Metabolism (1.3E-3)  Fructose and Mannose Metabolism (1.4E-3)  Fatty Acid Metabolism (2.9E-3)  Arachidonic Acid Metabolism (4.0E-3)  Calveolar-mediated Endocytosis Signaling (5.0E-3)  Phosopholipid Degradation (8.3E-3)  Virus Entry via Endocytotic Pathways (9.4E-3)  Crosstalk between Dendritic Cells and Natural Killer Cells (9.8E-3) |
| **7/ (60.5%)** |  | ↑ | ↓ |  |
| **8/ (48.6%)** | ↑ |  | ↑ |  |
| **9/ (45.0%)** |  | ↓ | ↑ |  |
| **10/ (51.8%)** | ↑ |  | ↑ |  |
| **11/ (59.8%)** | ↑ |  |  | Atherosclerosis Signaling (3.6E-7)  Hepatic Fibrosis/Hepatic Stellate Cell Activation (3.5E-5)  Dendritic Cell Metabolism (8.3E-4)  ***Fibrosis”** |
| **12/ (63.8%)** |  | ↓ |  |  |
| **13/ (81.3%)** |  | ↓ | ↑ | LPS/IL-1 Mediated Inhibition of RXR Function (7.5E-14)  Xenobiotic Metabolism Signaling (1.7E-13)  Metabolism of Xenobiotics by Cytochrome P450 (3.0E-13)  FXR/RXR Activation (1.2E-11)  PXR/RXR Activation (3.1E-11)  Aryl Hydrocarbon Receptor Signaling (2.2E-5)  NRF2-mediated Oxidative Stress Response (9.2E-5)  Glutathione Metabolism (1.3E-4)  Arginine and Proline Metabolism (3.6E-4)  Fatty Acid Metabolism (9.7E-4)  Tryptophan Metabolism (1.6E-3)  ***Nuclear receptor signaling** |
| 14/ (36.6%) | ↑ |  |  | IGF-1 Signaling (1.8E-4) |
| **15/ (52.5%)** |  | ↑ |  |  |
| **16/ (52.6%)** |  |  | ↓ |  |
| **17/ (73.5%)** |  |  | ↑ |  |
| **18/ (69.3%)** |  |  | ↓ |  |
| **19/ (42.8%)** |  | ↑ |  |  |
| 20/ (24.2%) | ↓ |  | ↓ |  |
| **21/ (66.6%)** |  | ↓ | ↑ |  |
| **22/ (65.1%)** |  |  | ↓ |  |
| **23/ (62.5%)** |  |  | ↑ |  |
| **24/ (54.0%)** | ↑ |  | ↑ | Sphingolipid Metabolism (6.0E-5)  ***Paneth cell markers** |
| **25/ (58.3%)** | ↑ |  | ↑ |  |
| **26/ (58.3%)** |  | ↑ | ↓ |  |
| **27/ (76.9%)** | ↓ | ↓ |  |  |
| **28/ (64.0%)** |  | ↓ |  |  |
| **29/ (63.3%)** | ↓ |  |  |  |
| **30/ (51.5%)** | ↓ |  |  |  |
| 31/ (33.7%) |  | ↓ |  |  |
| **32/ (85.6%)** | ↑ |  | ↑ | Primary Immunodeficiency Signaling (1.5E-8)  ***Immunoglobulins** |
| **33/ (45.3%)** | ↓ |  |  |  |
| **34/ (48.0%)** | ↑ | ↑ |  | Role of Macrophages, Fibroblasts, and Endothelial Cells in Rheumatoid Arthritis (4.9E-7)  IL-17 Signaling (3.9E-6)  Glucocorticoid Receptor Signaling (1.4E-5)  Atherosclerosis Signaling (1.5E-5)  Acute Phase Response Signaling (1.7E-5)  HIF1α Signaling (3.2E-4)  Systemic Lupus Erythematosus Signaling (5.4E-4)  Dendritic Cell Maturation (1.2E-3)  Colorectal Cancer Metastasis Signaling (7.0E-3) |
| **35/(55.8%)** |  |  | ↓ |  |
| 36/ (57.3%) |  |  | ↓ |  |
| **37/ (65.9%)** |  |  | ↑ | Antigen Presentation Pathway (6.42E-24)  Crosstalk between Dendritic Cells and Natural Killer Cells (6.83E-17)  Allograft Rejection Signaling (2E-15)  Autoimmune Thyroid Disease Signaling (3.14E-15)  Graft-versus-Host Disease Signaling (5.88E-15)  Type I Diabetes Mellitus Signaling (7.75E-12)  Dendritic Cell Maturation (7.92E-11)  Neuroprotective Role of THOP1 In Alzheimer’s Disese (5.87E-10)  Systemic Lupus Erthematosus Signaling (1.19E-7)  Communication between Innate and Adaptive Immune Cells (7,1E-7)  Protein Ubiquitination Pathway (4.4E-5)  ***MHC molecules** |
| **38/ (64.8%)** | ↓ |  | ↓ |  |
| **39/ (63.8%)** |  | ↓ |  |  |
| **40/ (52.4%)** | ↓ |  | ↓ |  |
| **41/ (57.2%)** |  | ↑ | ↓ | ***Adult hemoglobin genes** |
| **42/ (52.3%)** |  |  | ↓ |  |
| **43/ (93.6%)** |  | ↓ | ↑ | Retinol Metabolism (2.5E-11)  Pentose and Glucuronate Interconversions (3.7E-11)  Androgen and Estrogen Metabolism (6.9E-10)  Starch and Sucrose Metabolism (8.2E-10)  Metabolism of Xenobiotics by Cytochrome P450 (1.6E-9)  Xenobiotic Metabolism Signaling (1.1E-7)  ***UDP-glucuronyltransferases** |
